# Probing sociodemographic influence on code-switching and language choice in Quebec with geolocation of tweets

**DOI:** 10.3389/fpsyg.2023.1137038

**Published:** 2023-05-02

**Authors:** Olga Kellert

**Affiliations:** Department of Romance Linguistics, University of Göttingen, Göttingen, Germany

**Keywords:** language contact, code-switching, bilingualism, Quebec, geolocation, Twitter

## Abstract

This paper investigates the influence of the relative size of speech communities on language use in multilingual regions and cities. Due to peoples’ everyday mobility inside a city, it is still unclear whether the size of a population matters for language use on a sub-city scale. By testing the correlation between the size of a population and language use on various spatial scales, this study will contribute to a better understanding of the extent to which sociodemographic factors influence language use. The present study investigates two particular phenomena that are common to multilingual speakers, namely language mixing or Code-Switching and using multiple languages without mixing. Demographic information from a Canadian census will make predictions about the intensity of Code-Switching and language use by multilinguals in cities of Quebec and neighborhoods of Montreal. Geolocated tweets will be used to identify where these linguistic phenomena occur the most and the least. My results show that the intensity of Code-Switching and the use of English by bilinguals is influenced by the size of anglophone and francophone populations on various spatial scales such as the city level, land use level (city center vs. periphery of Montreal), and large urban zones on the sub-city level, namely the western and eastern urban zones of Montreal. However, the correlation between population figures and language use is difficult to measure and evaluate on a much smaller sub-urban scale such as the city block scale due to factors such as population figures missing from the census and people’s mobility. A qualitative evaluation of language use on a small spatial scale seems to suggest that other social influences such as the location context or topic of discussion are much more important predictors for language use than population figures. Methods will be suggested for testing this hypothesis in future research. I conclude that geographic space can provide us information about the relation between language use in multilingual cities and sociodemographic factors such as a speech community’s size and that social media is a valuable alternative data source for sociolinguistic research that offers new insights into the mechanisms of language use such as Code-Switching.

## 1. Introduction

Code-Switching or the mixing of multiple languages in a single conversation (abbreviated as CS) is a very well-known phenomenon of language contact. Examples (1) and (2) show CS within a sentence between English and French from Cook (1991) and a tweet from Montreal. In both sentences, the switch occurs from French into English (henceforth CS-Engl.):

1. J’ai acheté *an american car*. “I bought an American car” (Cook, 1991).
2. Nouveau café *in my hood*. “New café in my hood” (Twitter).

CS can also occur from English into French as shown in the following tweet in (3) from a bilingual photographer from Montreal (henceforth CS-French):

1. So this guy is 1 TODAY!! Happy birthday C.! *Je ne savais pas quoi faire*. Soft and bright is what we needed. *Donc une vieille porte a fait l’affaire*.
  “I did not know what to do. […]. An old door did the trick.”

Bilingual or multilingual speakers can use two or more languages without necessarily using Code-Switching, as shown by examples (4) and (5) from the same bilingual user. This particular user is from Montreal and has posted 23 tweets in French and 22 in English.

1. Notre devoir de citoyen est. fait! Allez voter, oubliez pas. @ xxxxx^1^ High School.
2. Last year I got to see the mtlalouettes for the first time and promised myself to come back with my son (mtlalouettes is a football team from Montreal).

The influence factors on Code-Switching (CS) as in (1–3) and on Language Choice of Bilinguals (LCB) as in (4) and (5) have been investigated from various perspectives, such as the structural (Poplack, 1980; Cook, 1991; Myers-Scotton, 2002), social (Schweda, 1980; Poplack, 1985; Fishman, 2000; Bullock and Toribio, 2009; Gardner-Chloros, 2009; Holmes and Wilson, 2013; Valenti, 2014), cognitive (Müller, 2017; Kremin et al., 2021, among others), and discourse perspectives (Gumperz and Dell, 1972; Konidaris, 2004; Auer, 2007; Auer and Eastman, 2010). One important factor influencing CS and LCB is the relative size of speech communities (Schweda, 1980; Poplack, 1985), alongside other factors such as age, addressee, topic of discussion, and language attitude (see Berisso Genemo, 2022 for various factors). CS from French into English, for instance, has been shown to be more prominent in the city of Ottawa than in the city of Hull (Poplack, 1985). This difference has been explained by the difference in the size of the anglophone population (Poplack, 1985). Ottawa has a larger anglophone community than Hull. An intuitive explanation for this correlation is that the larger anglophone community in a city like Ottawa results in a higher amount of language use in English. This can affect the speakers of French from the same city in their language use by being exposed to a large amount of English use. Previous research has shown that frequency strongly affects language production (Unsworth, 2016, among others).

Schweda (1980) has made an observation similar to that of Poplack (1985) with respect to LCB. Bilingual speakers adapt to the local environment by choosing the language that is more often used in a given city or area. Bilinguals thus tend to use English more often than French in a city where more English than French is used (Schweda, 1980). The intuitive explanation of this effect is that multilinguals adapt their language use to their local environment in a way similar to how they adapt their language use to their addressees (Konidaris, 2004).

However, there are studies on LCB and CS that seem to contradict this intuitive correlation between the size of a language community and language use such as CS and LCB. Lamarre et al. (2002) study shows that despite the Canadian census data from 2011 predicting more LCB (English) in the west of the island of Montreal than in the east (Statistics Canada, 2011) because it shows a relatively higher number of anglophone speakers in the west than in the east (see Timiou, 2014 for visualization of Statistics Canada, 2011), language use does not depend entirely on the geographic distribution of the population (Lamarre et al., 2002). Bilinguals from Montreal use both languages independent of their location, especially in informal contexts such as on the street and in coffee houses (Lamarre et al., 2002). This is unexpected given the results from previous studies based on different methodologies ranging from tweet analysis to picture analysis of street signs, which show a clear geographic separation of languages in Montreal, indicating more English in the west and more French in the east (Bouchard, 2000; Laur, 2003; Termote, 2003; Mocanu et al., 2013; Leimgruber and Fernández-Mallat, 2021). However, the latter studies did not investigate language use by *bilinguals* as the authors in Lamarre et al. (2002) study did, which might explain the difference in the results. One possible explanation for the conflicting results in previous studies such as that of Poplack (1985) and of Lamarre et al. (2002) is that they are based on few location points and/or few bilingual speakers, which is very likely related to the challenge of data collection. CS, for instance, is a spontaneous phenomenon and is more often used in informal contexts. CS is almost never used in legal documents or other highly formal contexts. In order to collect natural language data with CS, natural language needs to be collected in authentic and informal communication contexts. This requirement excludes the use of many linguistic corpora that contain news articles, linguistic questionnaires, or survey-based methods. The latter two methods are based on asking a selected group of people about their language behavior in a specific context, mainly “at home” (Bouchard, 2000; Laur, 2003; Statistics Canada, 2011). People cannot be asked under what circumstances they code-switch and how often they do it, as CS often occurs spontaneously and speakers are not always aware of when they are code-switching. For this reason, a different methodology and a different data source are needed to test correlations between the size of the population of a particular city or neighborhood and CS or LCB from the same location.

The present study aims at clarifying the influence of the population size on CS and LCB by using tweets from Twitter associated with location information and user IDs that are necessary for identifying bilinguals and for measuring the intensity of CS and LCB per geographic area. This information will provide answers concerning whether the intensity correlates with the size of the speech communities in a given location. The size of a speech community will be taken from population data published by Statistics Canada (2011).

*General Hypothesis*: CS and LCB correlate with the size of speech communities (Poplack, 1985).

The outline of the paper is as follows. Section 2 expands on the General Hypothesis with several more operational hypotheses in more detail by looking at predictions from Statistics Canada (2011). Section 3 presents the data source and the methodology to test the detailed hypotheses from Section 2. Section 4 shows the results, and section 5 discusses the results and future research plans.

## 2. Hypotheses

**Figure 1:**
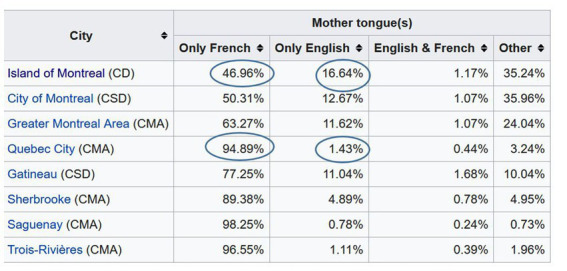
Census Profile from Statistics Canada (2011) showing relative numbers of French (see column “Only French”) and English speaking communities (see column “Only English”). My emphasis of differences between numbers of speech communities.

According to Figure 1 from Statistics Canada (2011), there is a much higher percentage of anglophones on the island of Montreal and in the city of Gatineau (a city on the border to Ontario), than in the city of Quebec.

*Hypothesis 1:* CS into English should be higher on the island of Montreal and in the city of Gatineau than in the city of Quebec.

According to Figure 1, the anglophone population is larger on the island of Montreal than in Greater Montreal (GM), which includes the island and the surrounding area. According to Statistics Canada (2011), visualized by Timiou (2014) on a geographic map, the anglophone population on the island of Montreal is larger in the western part of the city than in the eastern part. The population differences (+/−Greater Montreal) and urban zones (+/−western part of the island) should have an effect on language use.

*Hypothesis 2:* Bilinguals use more English on the island of Montreal than in Greater Montreal.

*Hypothesis 3:* Bilinguals use more English in the western part than in the eastern part of the island.

*Hypothesis 4:* There is more CS into English on the island of Montreal than in Greater Montreal.

*Hypothesis 5:* CS into English is higher in the western part than in the eastern part and CS into French is higher in the eastern part than in the western part.

These five hypotheses will be tested in section 4 after the section Data and methodology.

## 3. Data and methodology

### 3.1. Data

This paper uses Twitter as a data source to test Hypotheses 1–5 listed in the previous section. Twitter is characterized as a “microblogging platform,” with the prefix “micro-” referring to the brevity of the posts. The platform allows registered users to distribute short messages (tweets). Tweets, unlike WhatsApp messages, are not private but public, which means that everybody can read them.

**Figure 2:**
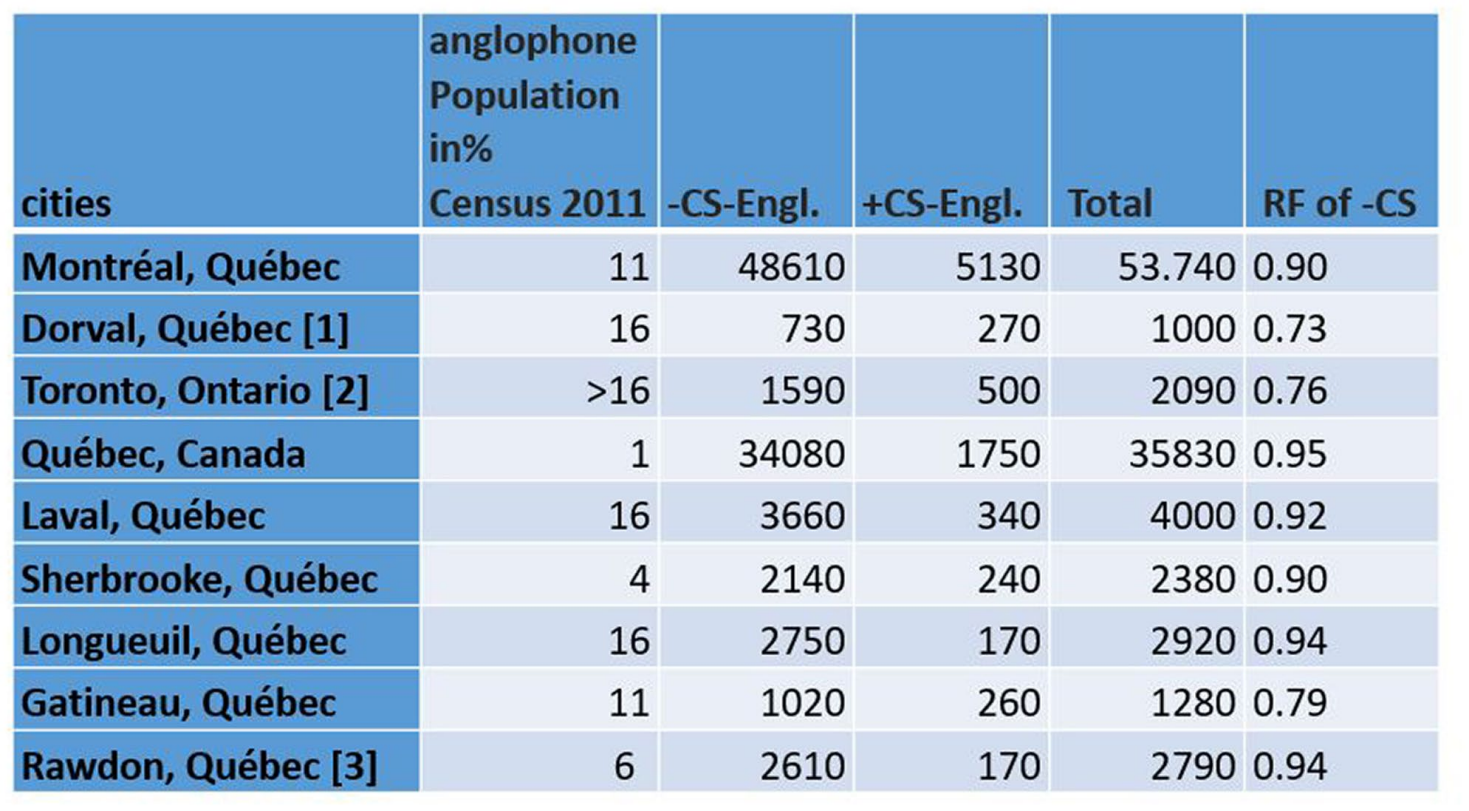
Number of Tweets with and without CS in cities extracted by “city name” from Twitter that have a higher number than 150 Tweets for +CS. Right most column: Relative Frequency calculation of –CS: – CS/Total. [1] Dorval has not been studied as a separated city from the island of Montreal according to Canadian census 2011. This is why I use the same number of population as the island of Montreal. [2] https://www.ontario.ca/document/2016-census-highlights/fact-sheet-6-mother-tongue-and-language. [3] https://www12.statcan.gc.ca/census-recensement/2021/as-sa/fogs-spg/page.cfm?dguid=2021A00052462037&lang=F&topic=1.

**Figure 3:**
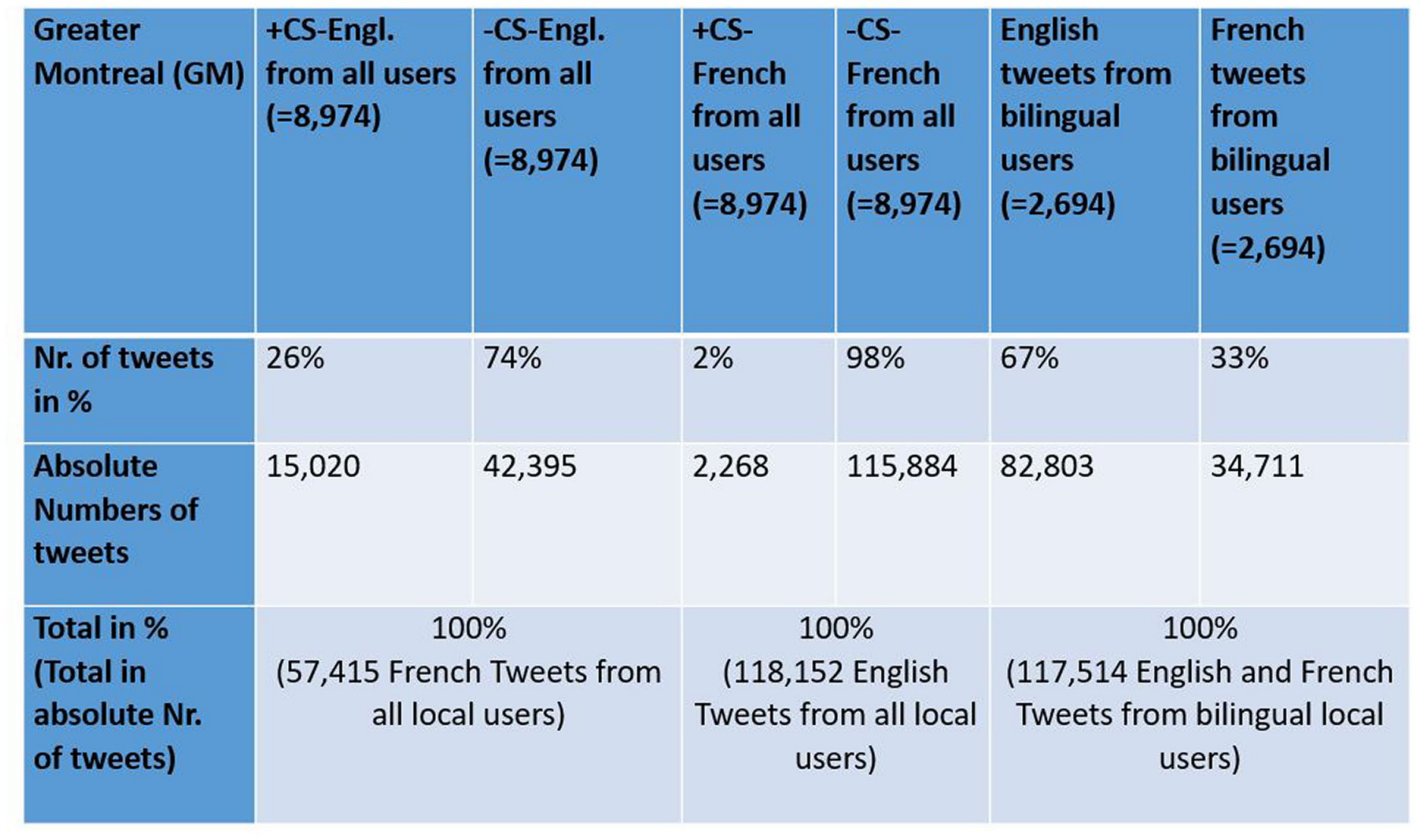
Distribution of Language use [+/−CS-Engl., +/−CS-French, LCB (English), and LCB (French)] in GM.

One of the great advantages of using social media platforms like Twitter as a data source for linguistic analysis is the large amount of speech data and corresponding meta-data, such as geolocation data and user information (Mocanu et al., 2013; Gonçalves and Sánchez, 2014; Levy et al., 2018, among others), which I will present in more detail below. Just to provide some numbers that illustrate the size of the data set I used in this study: I analyzed more than 100,000 geolocated tweets from bilinguals from the city of Montreal to study their language choices in space and almost 9,000 bilingual and non-bilingual Twitter users from Montreal (for detailed numbers, see Figures 2, 3, which will be commented in the corresponding sections). This is a scale quite different from that of most sociolinguistic studies conducted in Montreal, which typically analyze no more than a handful of speakers from the city (Lamarre et al., 2002; Konidaris, 2004, among others). Another advantage of using Twitter for the study of CS and LCB is that many text messages are written in various contexts such as coffee bars, restaurants, streets, work and at home (Kruspe et al., 2021) and that many tweets represent informal speech (Scheffler et al., 2022). Indeed, Twitter has been used to extract CS such as Spanish–English Code-Switching in the United States (Mendels et al., 2018).

The data used in this study are from a tweet corpus collected from November 2017 through March 2021 (Kellert, 2022). I used the language tag “lang” == “fr” for French and “lang” == “en” as defined by Twitter to find all French tweets and all English tweets in my corpus from particular locations, as will be defined in section 3.2.

### 3.2. Methodology

#### 3.2.1. Defining code-switching

Code-Switching into English (henceforth simply CS-Engl.) is defined as the use of English words in French tweets such as the tweet *Bye le frette et la neige* “Bye, ice cold and snow.” Code-Switching into French (CS-French) is defined as the use of French words in English tweets such as the tweet *Thanksgiving could not have been better*… *Merci Chérie* (….Thank you, darling.).

Note that the position of the English words used in French tweets and French words in English tweets was not considered in the present study (for structural aspects of CS, see Poplack, 1980; Cook, 1991; Myers-Scotton, 2002).

In order to calculate the number of tweets with and without Code-Switching (+/–CS), a list of English and French words that have the same meaning or grammatical function, such as French *anniversaire* vs. English *birthday* or English *not* vs. French *pas*, was created.

The list of French and English words contains around 150 word pairs from different semantic domains that occur very frequently in tweets: including greetings, goodbyes, and wishes (see Kellert, 2022 for the identification of the most frequent semantic domains on Twitter), which can be found as a supported file (see Supplementary File 1, henceforth “my list”).

This procedure of creating lists of lexical word pairs in order to be able to match their frequency in geographic space is a classic procedure in dialectology that investigates lexical distribution in space (Gonçalves and Sánchez, 2014; Grieve et al., 2019). Such a list has the advantage of controlling the spatial distribution of lexical items that have the same meaning [e.g., *Bye le frette et la neige* (+CS-Engl.) vs. *Au revoir le frette et la neige* “Bye, ice cold and snow!” (–CS-Engl.)]. In addition, the advantage of using a list of manually selected word pairs is that we can exclude lexical borrowings from English that have been assimilated into the Canadian French lexicon, such as *le sandwich* “the sandwich” or *cool* “cool/great” and that do not represent synchronic CS. Other (automatic) methods of CS identification as well as various types of CS such as more conventionalized and more spontaneous CS are reserved for future research (see section 5).

#### 3.2.2. City information

I used the city information encoded by the tag “city name” in the meta-data associated with the tweets to calculate the relative proportions of +/–CS-Engl. per city and visualize the use of +/–CS-Engl. on maps and compare cities where +/–CS-Engl. is more intense. If Hypothesis 1 in Section 2 is correct, the cities with the strongest CS-Engl. will fall out according to the relative size of the anglophone and francophone speech communities.

#### 3.2.3. Geolocation information

I used the *precise* geolocation of the tweets, that is, the location of the user when posting a tweet message to compare the amount of CS and LCB (French vs. English) per urban zone in Greater Montreal. Example (2), repeated here, is an example of a text message with geolocation information:

1. Nouveau café in my hood. “New café in my hood.”

The exact location of the user when sending the text message can be mapped on a geographic map like Google Maps using coordinates such as 45.523081 (latitude), −73.588132 (longitude), extracted from the tweets by using “geo coordinates.” In this case, the message was sent from *Le Saint Louis café* in Montreal.

By means of exact addresses or geolocation data, we can visualize the proportion of CS and LCB (French vs. English) on a geographic map showing Montreal and compare urban zones where CS-Engl. and CS-French and LCB are more intense (Mocanu et al., 2013; Gonçalves and Sánchez, 2014; Levy et al., 2018; Grieve et al., 2019, among others). This procedure will allow us to test Hypotheses 2–5. If these hypotheses are correct, CS and LCB will vary according to the eastern and western part of Montreal’s island and to +/−Greater Montreal, which correlate with differences in the size of the anglophone and francophone speech communities.

#### 3.2.4. Defining language choice of “bilingual” users

I defined bilingual users as those who tweet in both English and French, that is, with at least one tweet in English and at least one tweet in French in the area of Greater Montreal. In order to find bilingual users, I used user ID encoded by integers in the meta-data of tweets (“user id”).

#### 3.2.5. Calculation of relative frequencies and visualization on maps

A substantial part of the methodology in this section such as calculation of relative frequencies and visualization on maps is based on Kellert and Matlis (2022). I calculated the relative frequency of French tweets with English words (+CS-Engl.) and French tweets with French equivalents (–CS-Engl.) per city. I created city corpora by using the city information expressed as “city name” in the tweets’ meta-data.

In addition, I calculated +/−CS-Engl. and +/−CS-French as well as LCB (English and French) per urban zone or location in Greater Montreal. For the latter calculation, I used the geographic extent of Greater Montreal ([−74.031218, −73.284148, 45.833152, 45.323716]) to find tweets that were posted from the area covering this extent by using the geolocation information. I binned the geographic extent of Greater Montreal into 50 × 50 equal bins, which generated 2,500 bins for Greater Montreal. The size of a single bin corresponds more or less to the size of a city block (Kellert and Matlis, 2022). The absolute frequency counts of +/−CS-Engl. and +/−CS-French as well as LCB (English and French) per urban zone or location in Greater Montreal can be found as Supplementary Files 2–4.

We can refer to each such cluster of data—i.e., all data pertaining to one city or to one urban zone—as a “container” or “bin.” I used a particular calculation method that identifies the bins with the largest differences for +CS or –CS (see differential distribution, Kellert and Matlis, 2022).

Differential distribution compares the geographical shape of a distribution of one linguistic variant (e.g., tweets with English words or +CS-Engl.) with the geographical distribution of another linguistic variant (e.g., tweets with French words with the same meaning or –CS-Engl.). If the geographical shapes of two distributions (+CS-Engl. and –CS-Engl.) overlap, they are the same, which means there is no difference in the distributions. Ultimately, what the Differential Distribution does is measure the difference between distributions per bin. The following mathematical definition of the differential distribution is a modified version of that found in Kellert and Matlis (2022).

We first define normalized tweet distributions by: $f_{i,j}^{T}\equiv c_{i,j}^{T}/N^{T}$ and $f_{i,j}^{R}\equiv c_{i,j}^{R}/N^{R}$, where $N^{T}\equiv \underset{i}{\sum }\underset{j}{\sum }c_{i,j}^{T}$ and $N^{R}\equiv \underset{i}{\sum }\underset{j}{\sum }c_{i,j}^{R}$ are the total number of tweets in the target (+CS) and reference (–CS) distributions, respectively. The quantities $f_{i,j}^{T}$ and $f_{i,j}^{R}$ represent the fraction of tweets in the (i,j)th bin for the target and reference cases, respectively. The comparison between the two distributions is then done by calculating the difference in the tweet fraction per bin: $\Delta f_{i,j}\equiv f_{i,j}^{T}-f_{i,j}^{R}$, which is referred to as “differential distribution” in Kellert and Matlis (2022). This quantity can be interpreted as follows: bins with positive values of $\Delta f_{i,j}$ over-represent the target tweets, while negative values under-represent them, relative to the reference tweet distribution. Since bins with equal representation of tweets have $\Delta f_{i,j}=0$, independently of the total numbers of each variant, small variations in degree of representation can be resolved. This metric does not require special treatment for bins with zero counts, and results in larger values of $\Delta f_{i,j}$ for larger variations, even if either of the tweet counts are zero. As a result, noise associated with low-count bins is suppressed. A consequence of the normalization is that the sum of the distribution differences is exactly zero, $\underset{i}{\sum }\underset{j}{\sum }\Delta f_{i,j}=0$, so that for any two distributions, the contributions from each will be equal and all bins will be identically zero ($\Delta f_{i,j}=0$) for two distributions of exactly the same shape but a different total number of tweets.

Let us go through the mathematical calculation using a hypothetical example. Assume that we have five cities with a total sum of 80 tweets with French words (–CS-Engl.) and a total of 240 tweets with English words (+CS-Engl.) from my list of word pairs. Let us further assume that a particular city x has 10 tweets with French words (–CS-Engl.) and 30 with English words (+CS-Engl.). Applying the calculation of differential distribution above, we get Δ = 30/240–10/80 = 0. The result of zero means that city x is not prominent for +CS-Engl. or –CS-Engl., as the difference between +CS-Engl. and –CS-Engl. is zero. If a city has a higher value, it is more prominent for +CS-Engl., and if it has a negative value, it is more prominent for –CS-Engl. As will be shown in section 4.1, this calculation is especially susceptible to overemphasizing differences in distributions, where differences calculated by simple relative frequencies (that is, *x* number of observations divided by the sum of all observations) show only a small variation.

I visualized the results from the calculation of differential distribution on geographic maps by marking the location in red if the difference of CS-Engl. was positive, that is, if there were more tweets with English words in comparison to all other locations. Otherwise, I marked the location in blue (see visualization technique in Kellert and Matlis, 2022). I marked location in purple, if CS-French was more prominent than the absence of CS-French. The size of the circle corresponds to the size of the delta. The larger the red circle, the more positive the delta of CS-Engl., and the larger the blue circle, the more negative the delta. The same case applies for CS-French, that is, the larger the purple circle, the higher the intensity of CS-French. For the visualization, I used Cartopy (see Cartopy v0.11.2. Met Office, 2014), which is an open source, that is, freely available and modifiable, software that maps coordinates to Open Street Maps, which is also freely available. All figures in this document were produced by using the base map and data from OpenStreetMap and OpenStreetMap Foundation under the Open Database License.

#### 3.2.6. Preprocessing/prefiltering

Montreal is a popular tourist destination from which tourists from France might tweet in French and tourists from various other countries might tweet in English. Tourists might thus influence the statistics.

In order to check how much tourists influence the differential distribution of LCB in Montreal, I performed an experiment. I defined local users as those who created their profile in Montreal, as encoded by “user location” on Twitter. I assumed that the user location very likely represents the “place of residence” of the Twitter user (see Kellert and Matlis, 2022). I checked this assumption on a random set of Twitter profiles by looking at other cues that might provide evidence of the user’s origin, such as the profile description and the content of the tweets. Let us consider an example of a user with an account from Montreal. The user says on her profile that she is a *Passionate Montrealer*, and furthermore she says she *tweets in English and French*, which can also be seen by the Code-Switching in the profile description, where she says she is a *Creative mind* and then code-switches into French: *joueuse de tennis* “tennis player.” By using user IDs, I was able to identify tweets from this user and to check whether she tweeted mostly from Montreal. I filtered out users who tweet from Montreal but who did not create their profile in that city, assuming that these users are temporarily in Montreal as tourists.

I then tested whether LCB or language choice of bilingual users was much different depending on local users or on all users (including tourists). As it turns out there is no difference in spatial distribution of tweet behavior between local and all bilingual users from Montreal (see section 4.2). Not only is there no difference in spatial distribution of tweets, there is also almost no difference in frequency numbers of LCB from local and all users (117,514 Tweets from local bilingual users vs. 121,109 Tweets from all bilingual users). This result suggests that the majority of bilingual users French-English are local users, which makes intuitively sense because someone who is tweeting in French and English in Montreal is very likely someone from a multilingual region or city like Montreal.

## 4. Results

### 4.1. Cities and population differences: Testing hypothesis 1

The results show that Hypothesis 1 is confirmed for most of the cities that show enough data for a statistical comparison.

**Figure 4:**
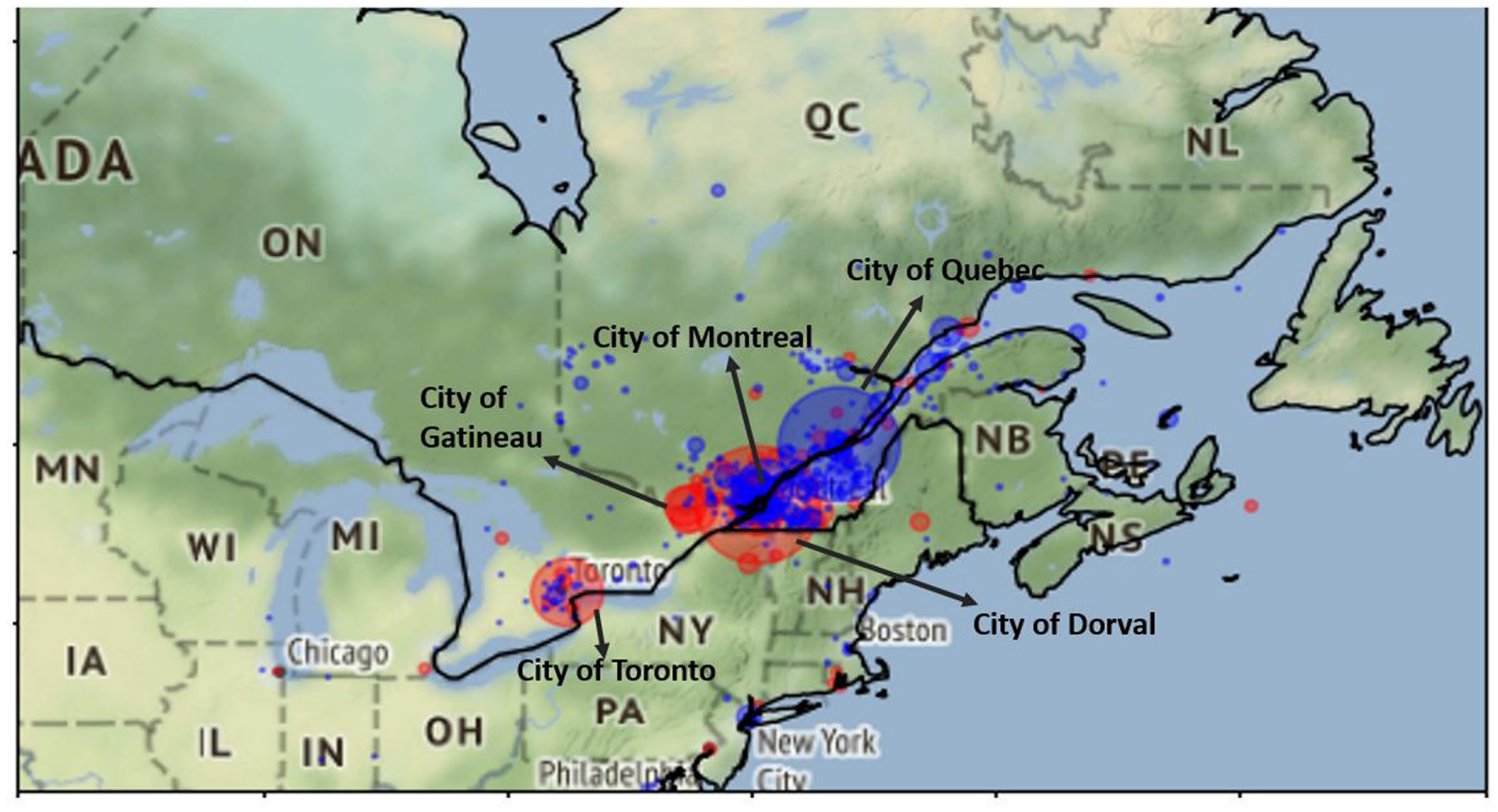
+/− Differential distribution of +CS-Engl. (red) und –CS-Engl. (blue) in French Tweets in Quebec and Ontario. City of Dorval is the most prominent city for +CS-Engl. in French tweets, followed by Toronto and the city of Gatineau. Base map and data from OpenStreetMap and OpenStreetMap Foundation under the Open Database License.

Figure 4 shows the differential distribution of CS-Engl. in the province of Quebec and its surroundings, such as the province of Ontario (see Figure 4). There are only a few cities that show clear differences, which are marked by easily identifiable big red and blue circles (nine cities in total). Many other cities marked as small blue or red points do not provide enough data points to be statistically relevant; that is, they show very few tweets with +/–CS-Engl. The statistical numbers for these nine cities are provided in Figure 2. The biggest red circle representing a city with the most prominent use of CS-Engl. in Figure 4 is Dorval, which is a small provincial city in the west of the island of Montreal. The second largest red circle is the city of Toronto, in the province of Ontario, and the third largest circle is the city of Gatineau at the border between the provinces Ontario and Quebec. The largest blue circle marking the highest absence of English words in French tweets (–CS-Engl.) corresponds to the city of Quebec. This is the city with the least CS compared to all other cities. Figure 2 also provides the relative frequencies of –CS-Engl. per city and the population numbers of anglophone communities in percentages published by Canadian Statistics (see Statistics Canada, 2011). Figure 2 shows that cities with the relative frequency (RF) of –CS-Engl. lower than 0.8 (<0.8) correspond to high percentages of anglophone inhabitants (over 10%). Cities with higher RF (>0.9) mostly correspond to lower anglophone population figures (under 10%). However, the city of Montreal shows comparatively less CS-Engl. than the city of Gatineau, which is less than expected from Hypothesis 1. In addition, the city of Montreal shows relatively more CS-Engl. than the city of Quebec, which is expected according to Hypothesis 1 according to both calculations: the differential distribution (see Figure 4) and the relative frequency calculation in Figure 2. However, the visualization in Figure 4 overemphasizes differences in CS-Engl. in cities; whereas the difference in RF between Montreal and the city of Quebec is a very low number (RF difference is 0.05 according to Figure 2).

To summarize, we observe a higher proportion of CS-Engl. in Dorval (a city on the island of Montreal) and cities on the border with Ontario, as well as in the city of Toronto in Ontario, than we do in the city of Quebec, which shows the least use of CS. The city of Montreal shows less use of CS than the city of Quebec, which is expected, but it shows less use of CS-Engl. than Gatineau, which is unexpected.

### 4.2. Influence of population differences in Montreal on LCB: Testing hypotheses 2 and 3

There are in total 8,974 local users in Montreal in my tweet corpus, 2,694 of whom (=almost 1/3) tweet in both languages. That is, there are 2,694 bilingual users, who posted 34,711 tweets in French and 82,803 in English (Figure 3). This result confirms Mocanu et al.’s (2013) observation that there is a general trend to write more tweets in English than in French in Montreal.

**Figure 5:**
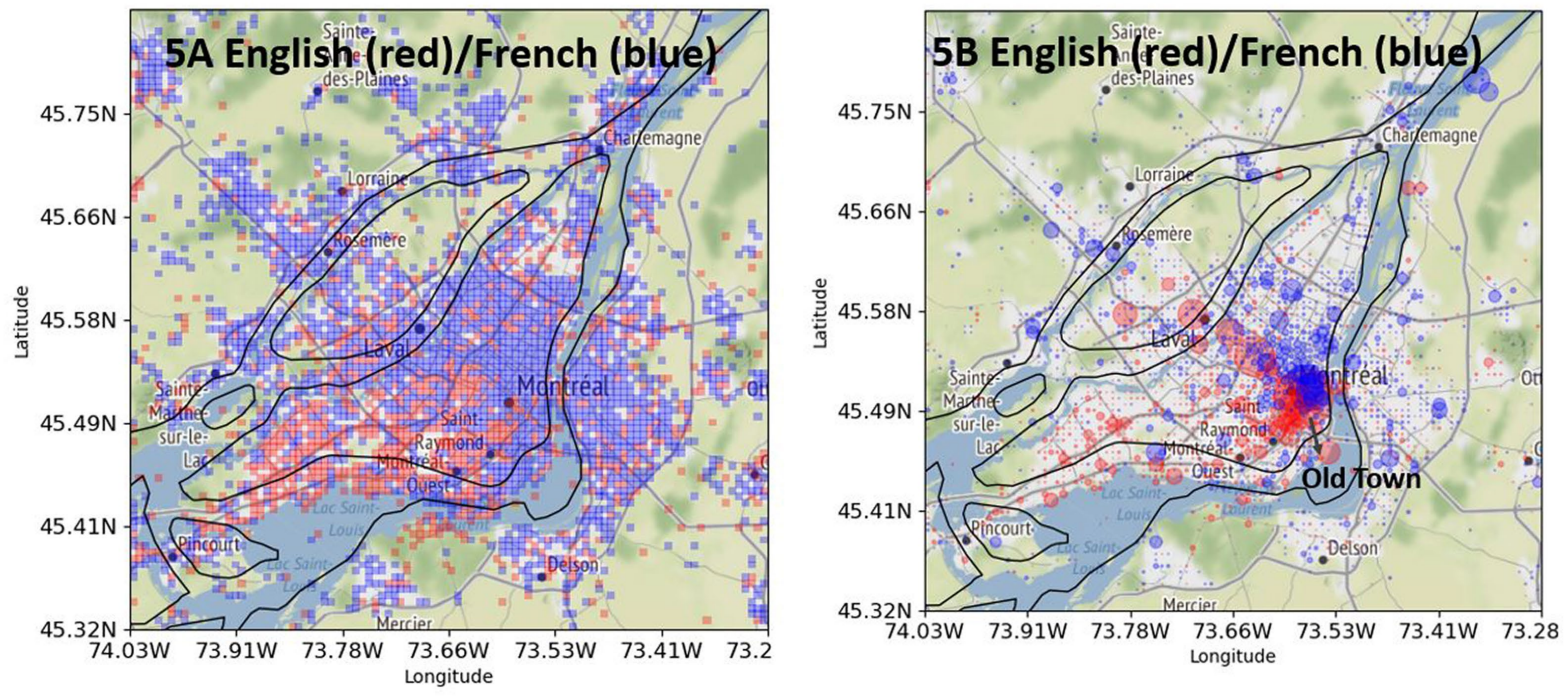
English tweets (red) and French tweets (blue) posted by “local” bilingual users in Greater Montreal. (**A**, left): without intensity. (**B**, right): with intensity. Base map and data from OpenStreetMap and OpenStreetMap Foundation under the Open Database License.

Figure 5 shows the differential distribution of English (red) and French tweets (blue) in Greater Montreal among bilingual users. Figure 5A shows the distribution in a square format, which emphasizes the geographic pattern of the distribution. The pattern is very clear: English is distributed more in the western part of the city, and French in the eastern part. Moreover, we see more blue than red outside the island of Montreal, which indicates that the difference between the island and the periphery plays a role in the distribution of LCB. These results confirm Hypothesis 2.

Figure 5B shows the same differential distribution as Figure 5A, but with an intensity marking, which shows where the intensity of English and French tweets is located the most. English tweets are very much concentrated in the old town of the city, and few big red circles are visible in the city of Laval. The intensity of a few big red circles helps us to evaluate the strength of the difference (Kellert and Matlis, 2022). In contrast to Figures 5A,B shows that the difference in the distribution of English tweets on the island and periphery is rather weak. This observation confirms the numbers from Figure 1 from Statistics Canada (2011), which show an approximate difference of 5% in the Anglophone population inside and outside the island of Montreal (see 16.64% vs. 11.62% in +/− Greater Montreal).

**Figure 6:**
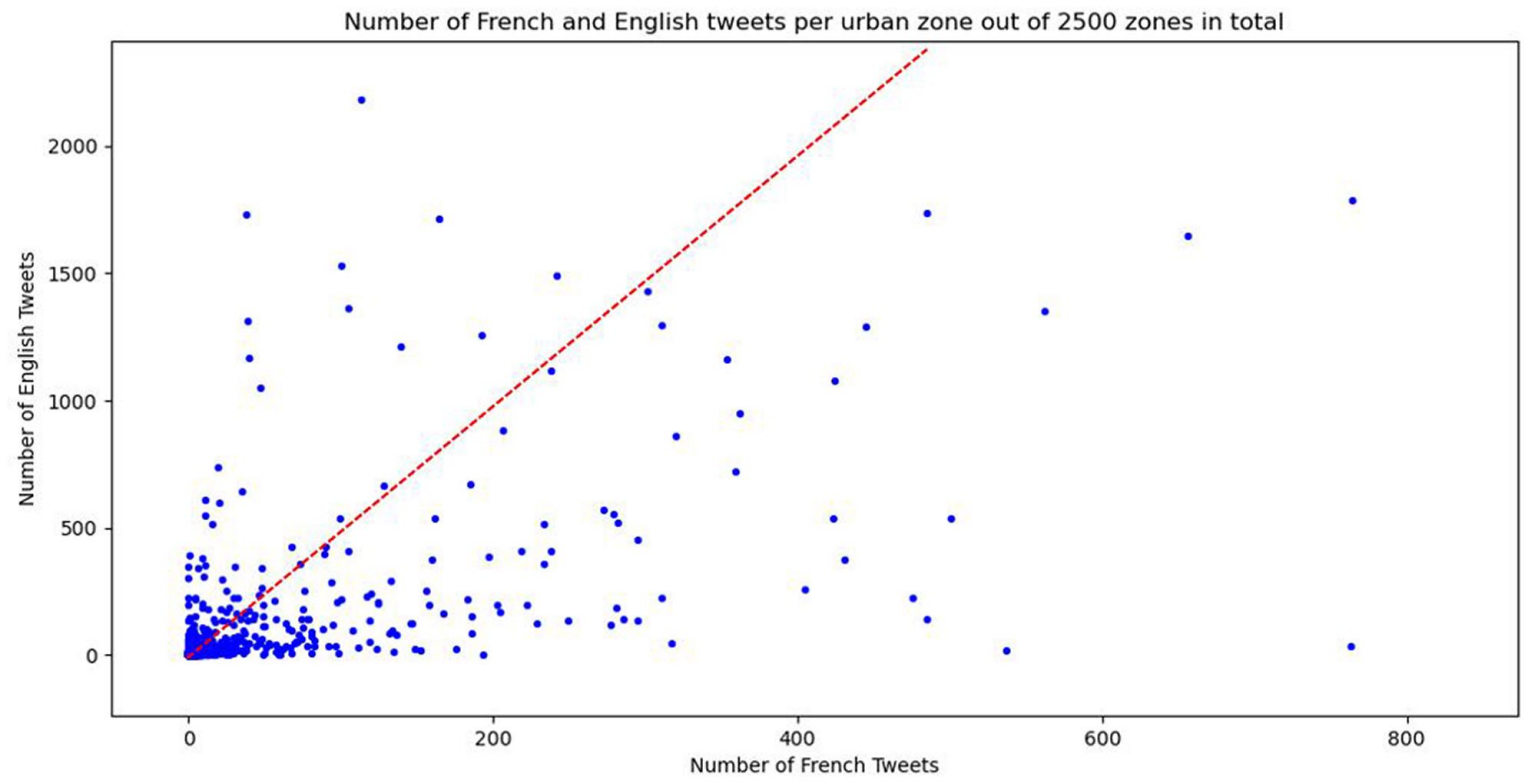
Frequencies of English (y-axis) and French Tweets (x-axis) per urban zone in GM. Red dashed line corresponds to a correlation line. The points following the correlation line represent locations with no difference in language choice.

Figure 6 shows the underlying data of Figure 5, representing the distribution of frequencies of English tweets (y-axis) and French tweets (x-axis) in Greater Montreal per bin (2,500 bins in total). The most important point of the distribution in Figure 6 is that not all points are distributed along the linear correlation line in red, which would suggest that each location in Greater Montreal has the same relative number of English and French tweets. If the data points followed the correlation line that would indicate that the location does not matter for LCB, contrary to Hypotheses 2 and 3 in section 2. What we instead see from the plot in Figure 6 is that some data points do follow the correlation line, but some do not. There are visible data points representing locations that show strong preferences for English, which are distributed close to the y-axis, and that show strong preferences for French, distributed close to the x-axis. These locations are the ones that show the biggest red or blue circles in Figure 5B. However, some locations do not show any difference in the relative frequency of English and French tweets, which means that they are not relevant for LCB.

**Figure 7:**
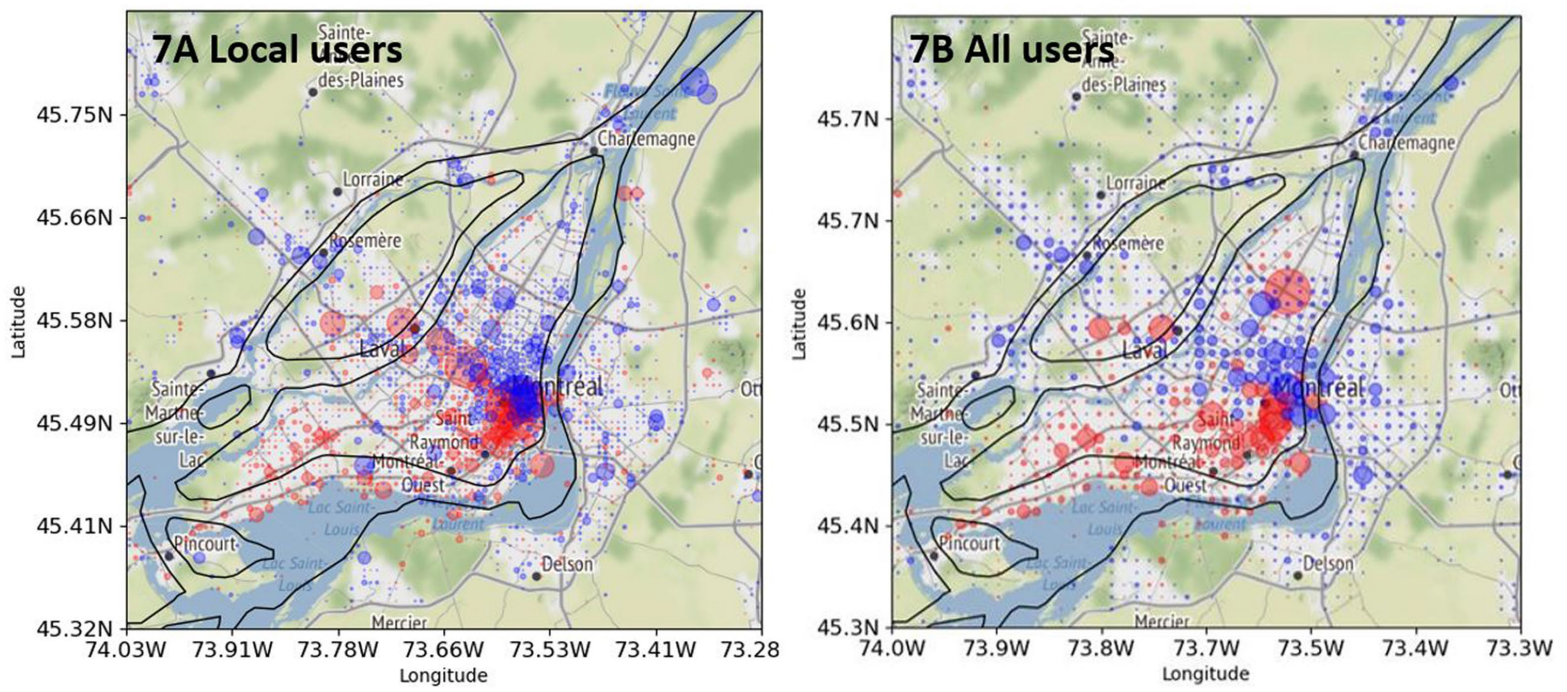
Comparison of English tweets (red) and French tweets (blue) posted by bilingual local and all users in Greater Montreal. (**A**, left): “local” bilingual users (see also Figure 5B). (**B**, right): “all” bilingual users. Base map and data from OpenStreetMap and OpenStreetMap Foundation under the Open Database License.

Finally, Figure 7 compares two distributions of English and French tweets. Figure 7A shows the distribution of tweets produced by local bilingual users (repeated from Figure 5B), whereas Figure 7B shows the distribution of tweets produced by all bilingual users without filtering out non-local bilingual users (see section 3.2.6 on preprocessing and filtering). The pattern in Figure 7B is almost the same as that in Figure 7A, which indicates that non-local bilingual users do not change significantly the spatial pattern of tweet distribution.

To summarize the results, some locations do show a division of the city into two linguistic zones among bilinguals, with French in the eastern part of the island and English in the western part, as well as more English on the island than on the periphery. However, some other locations do not show any difference in the distribution of French and English tweets. The latter observation is probably the effect that Lamarre et al. (2002) observed in their study on the basis of bilingual Montrealers using a different method.

### 4.3. Influence of population differences in Montreal on CS: Testing hypotheses 4 and 5

This section shows that CS depends on the location in Montreal, which confirms the hypotheses 4 and 5, but the strength of the pattern is rather weak.

**Figure 8:**
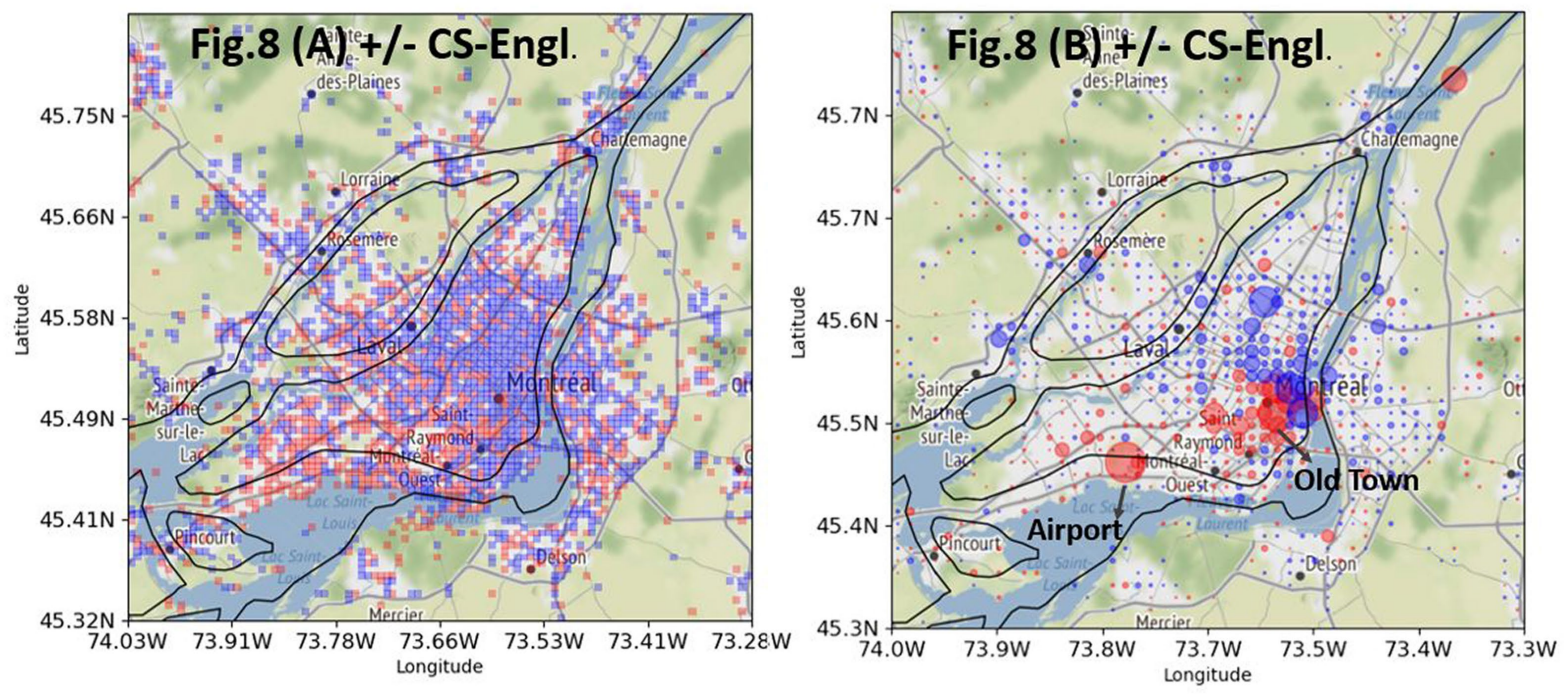
Differential distribution of +CS-Engl. (red) and –CS-Engl. (blue) in Greater Montreal. (**A**, left): without intensity. (**B**, right): with intensity. Base map and data from OpenStreetMap and OpenStreetMap Foundation under the Open Database License.

There is a total of 57,415 georeferenced French tweets from Greater Montreal from local users that contain one of the words from my list (see Figure 3). 15,020 tweets contain English words from my list (+CS-Engl.), and 42,395 tweets contain French words from my list (–CS-Engl.; see Figure 3). Despite this large number of tweets with English words or + CS-Engl., the tweets are not distributed everywhere on the island of Montreal and the city’s periphery, as Figure 8 shows. The square format in Figure 8A shows clearly a spatial pattern. CS-Engl. is heavily concentrated in the western part of the city, as shown by the color red. Consequently, CS-Engl. is mostly used in the area with the larger anglophone population according to Statistics Canada (2011).

Figure 8B shows the same distribution of CS as in Figure 8A, but with an intensity marker. The biggest red circles are located at the airport and in the old town. The question is why these contexts are especially prominent for English words in French tweets. One possible explanation is that at the airport, a particularly large number of goodbyes and greetings are expressed in English. Indeed, it is noticeable that many fixed or idiomatic expressions in English are used in goodbyes like *Bye, Here we come! It’s gonna be fun!*, as shown in examples (6–8).

1. Départ pour des petites vacances avec ma France!!! La Floride here We come!!! Après ça va aller vite…busy busy…
  “Departure with my France for a few days of vacation. Florida, here we come. Later, it will be very fast. Busy busy.”
2. 🛣 on the road -. 🚙 | It’s road trip time! On part pour quelques jours à Québec! 💙 It’s gonna be fun! 🤗. 📸 | ou….
  “on the road -. 🚙 | It’s road trip time! We’re going to Quebec for a few days! 💙 It’s gonna be fun!. 📸| or…”
3. “Bye le frette et la neige!,” “Bye ice cold and snow!”

This type of CS-Engl. corresponds to Poplack’s (1980) notion of “emblematic CS”; that is, CS at the airport is mainly used in connection with discourse units, particles, and word fillers, such as *bye, well, you know*, whereas the main message is written in French.

**Figure 9:**
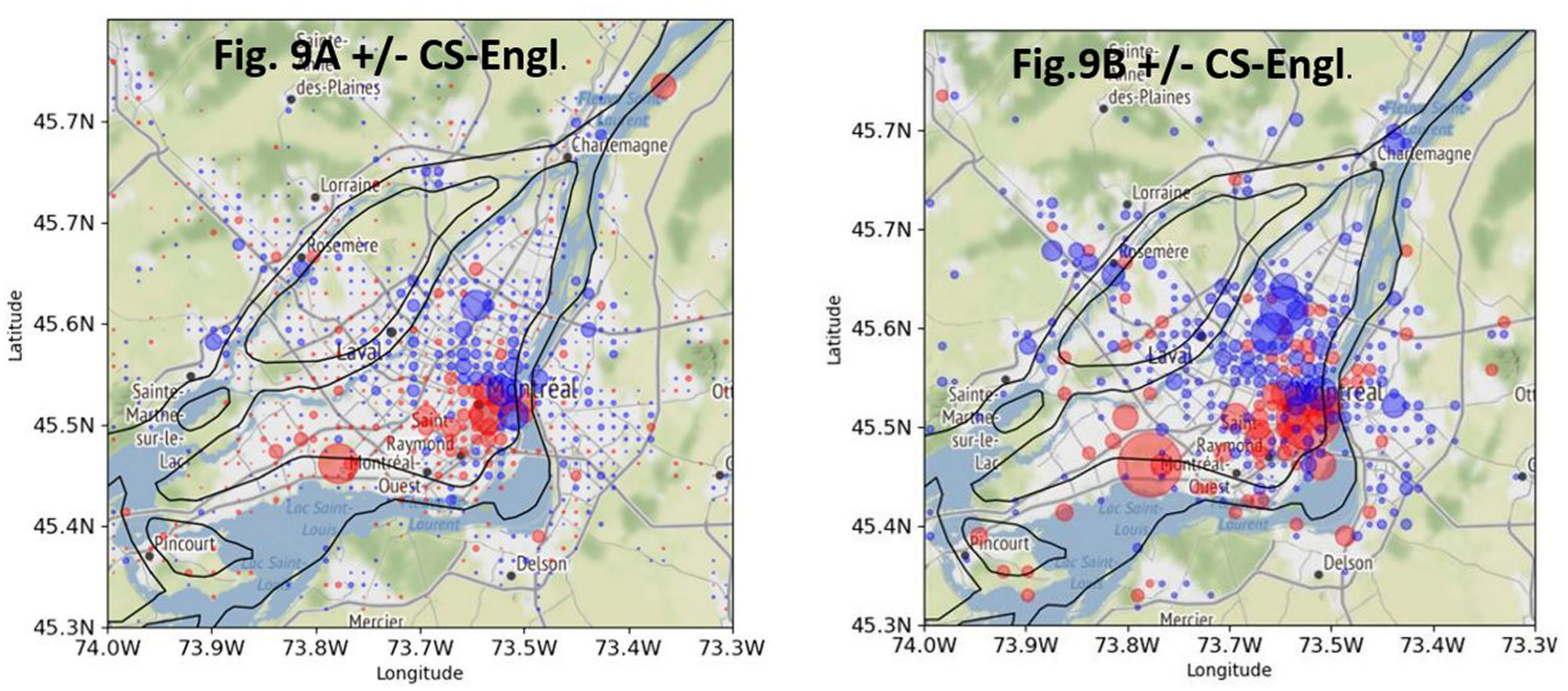
Comparison of +/−CS-Engl. in Greater Montreal. (**A**, left): full list. (**B**, right): self-referring items. Base map and data from OpenStreetMap and OpenStreetMap Foundation under the Open Database License.

In order to determine how stable the spatial pattern of CS-Engl. is in Montreal as shown in Figure 8B, I used a subset of the word pairs from my list that only contains self-referring expressions such as *my* or *mine* in English and corresponding words in French. Figure 9 shows a comparison between a tweet distribution of +/−CS-Engl. based on a full list of word pairs (see Figure 9A, repeated from Figure 8B) and a tweet distribution based on a subset of word pairs from my list (see Figure 9B). The comparison shows that the spatial pattern of +CS-Engl. and –CS-Engl. is still the same for a subset of word pairs from my list, that is, with more CS in the west than in the east. Actually, the pattern is even stronger in Figure 9B than in Figure 9A, which means that when users from Montreal talk about themselves, they use English words even more in the west and French words in the east compared to other words with no self-reference.

**Figure 10:**
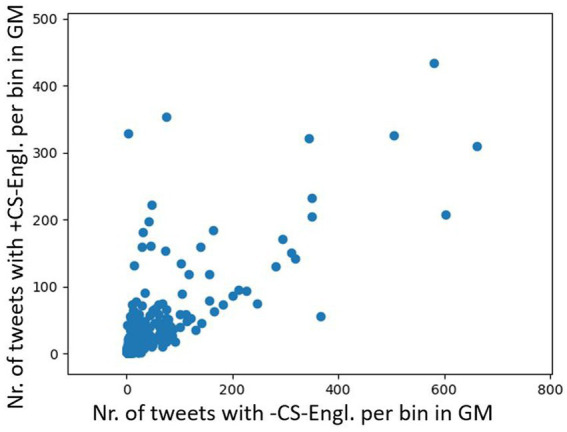
+/−CS-Engl. per urban location in GM.

**Figure 11:**
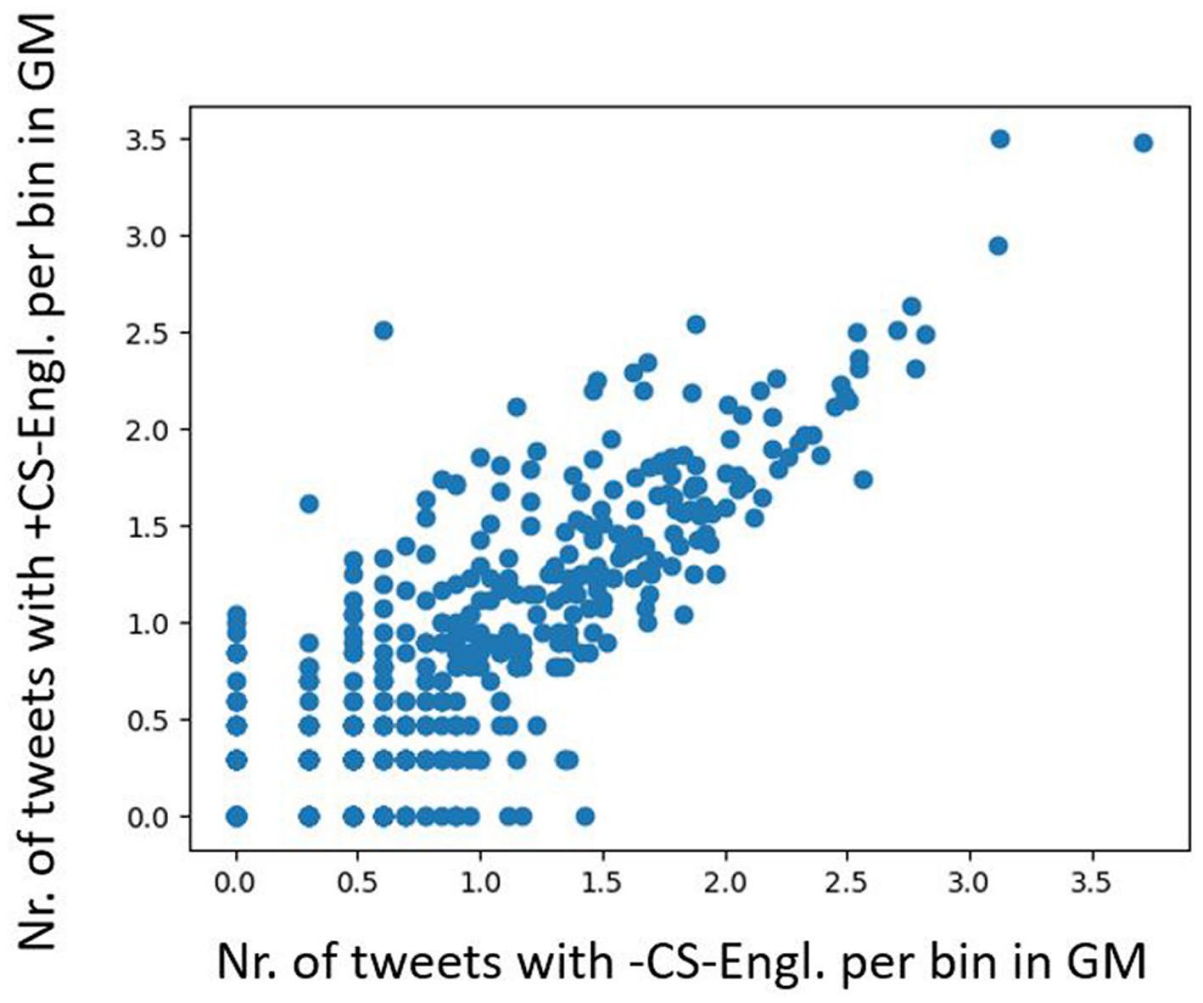
Base-10 logarithm on +/−CS per bin in GM.

**Figure 12:**
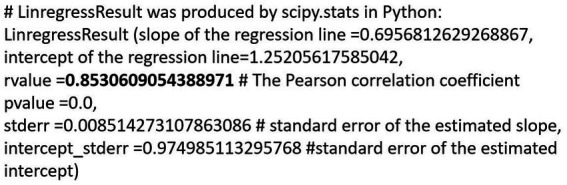
Statistics of +/−CS per bin in GM.

Figure 10 shows a plot with frequency distribution of tweet counts (+CS-Engl. and –CS-Engl. on a full list of word pairs). It shows similar results as with tweet distribution of LCB in Figure 6, that is, some locations do not show any difference for CS-Engl., whereas some do. As the numbers are much smaller for CS than for LCB, I used a base-10 logarithm on the data (Figure 11), which makes the pattern in Figure 10 more visible for small numbers. Figure 11 illustrates that higher tweet frequencies show a higher linear correlation in the distribution of tweets with and without CS-Engl. This means that locations from which a high number of tweets is posted do not show a huge difference in +/−CS-Engl. To test this observation statistically, I calculated the Pearson correlation coefficient (see Figure 12). The Pearson correlation coefficient value is rather high (>0.8). This means that, overall, the location does not strongly influence the distribution of +/–CS-Engl. in Greater Montreal, especially with higher frequency numbers.

How should we interpret this result, which seems to contradict the clear spatial pattern of +/-CS-Engl. in Figure 8A? The spatial pattern is there, but the signal or the effect is weak (see Kellert and Matlis, 2022 for a detailed discussion of the difference between the presence of a spatial pattern and the signal or the strength of the pattern).

**Figure 13:**
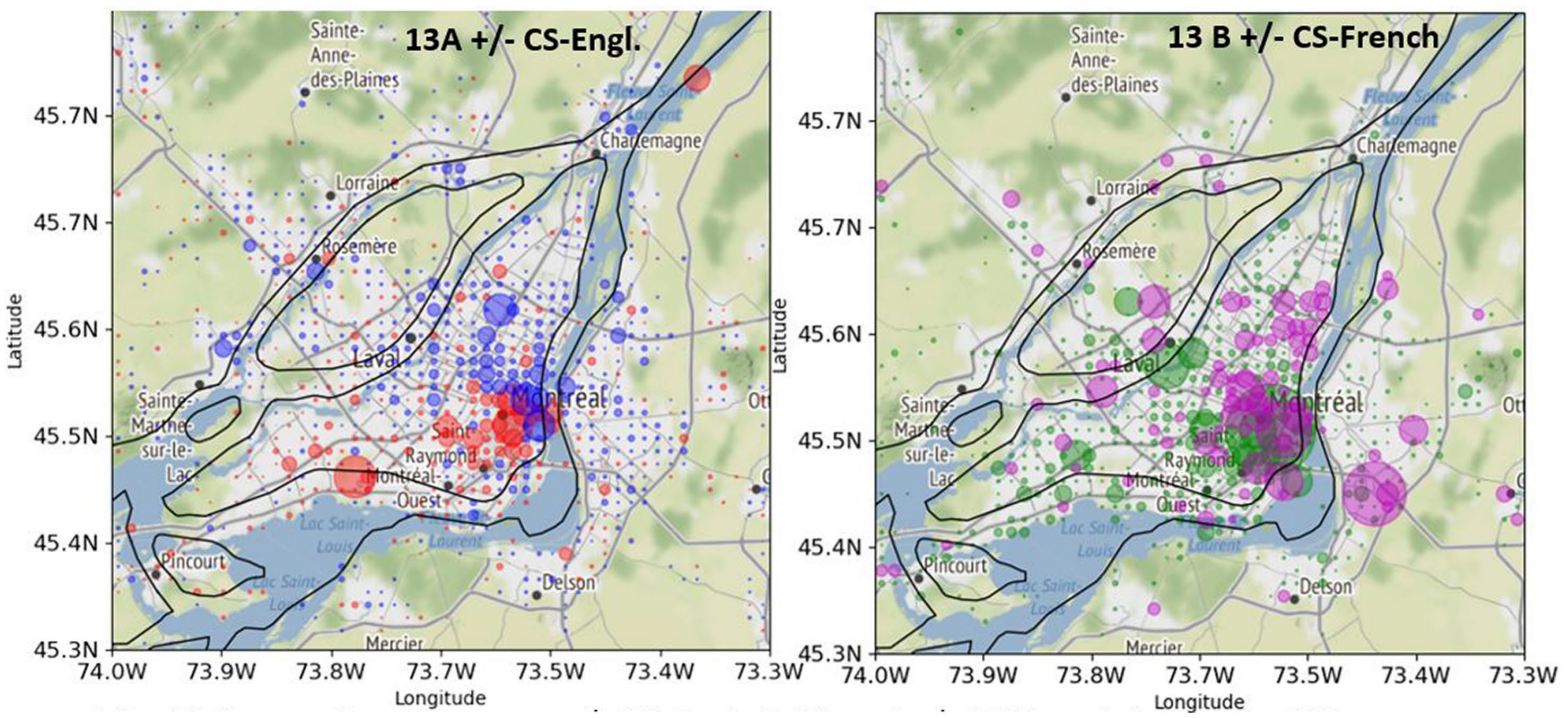
Comparison between +/−CS-Engl. (left) and +/−CS-French (right) in GM. (**A**, left): +CS-Engl. (red) and –CS-Engl. (blue). (**B**, right) + CS-French (purple) and –CS-French (green). Base map and data from OpenStreetMap and OpenStreetMap Foundation under the Open Database License.

Figure 13 shows a comparison between the spatial distribution of CS-Engl. in French Tweets (Figure 13A) and CS-French in English Tweets in Greater Montreal (Figure 13B). As there is comparatively few tweets with CS-French in English tweets (see Figure 3 for exact numbers), there are only few circles showing where CS-French is used comparatively more than the absence of CS-French in Greater Montreal. The few visible circles showing CS-French (in purple) are clearly concentrated in the east of the island of Montreal and outside of the island. The spatial pattern of Code-Switching (CS-Engl. and CS-French) shows that CS depends on location as predicted by the General Hypothesis. However, the strength of the pattern is rather weak.

## 5. Discussion and future research plans

One of the major contributions of this paper was to test the General Hypothesis, repeated here below, which has led to conflicting results in previous studies (Poplack, 1985 vs. Lamarre et al., 2002):

*General Hypothesis*: CS and LCB correlate with the size of speech communities (Poplack, 1985).

The results of CS-Engl. in cities seem to show a trend confirming the General Hypothesis when comparing entire cities (see Hypothesis 1). However, not all cities have yielded enough data to be able to confirm the General Hypothesis due to low numbers of tweets. This result can be improved in the future. One possible improvement is to extend my word-pair list with additional word pairs or to use a completely different approach for measuring CS that does not rely on manually crafted word-pair lists (Mendels et al., 2018, among others). In the future, it will be necessary to test the automatic language classification of tweets and/or of words to improve the identification of CS and LCB. In this paper, I relied on the language classification performed by Twitter. Twitter’s language classifier from 2015 labels English Tweets with 99% precision (see Twitter website^2^). However, Twitter also mentions that minor languages and tweets with mixed languages such as English and French have a lower degree of precision (Mendels et al., 2018).

One important issue related to the identification of CS concerns the definition of CS. In this paper, as well as in many other computational linguistic approaches to CS (Mendels et al., 2018 for an overview), CS is defined very broadly as mixing of languages and thus also includes any kind of mixing such as formulaic expressions or “conventionalized” CS and language translations used in the same tweet such as *We are open! Nous sommes ouverts!* In linguistics, however, formulaic expressions are distinguished from “proper,” “genuine” or “spontaneous” CS (Poplack, 1980, among others). I will address this issue in detail in future research.

Another important result of this study is the contribution to testing the General Hypothesis at the level of Greater Montreal (island of Montreal and periphery) and on the sub-city level (east and west of the island) using precise geolocation information. On this level of spatial granularity, the results are mixed. Looking at geographic patterns of CS and LCB in Figures 5A, 8A, there is a trend of spatial division as predicted by the General Hypothesis or more precisely by Hypotheses 2–5. However, by looking into the statistical numbers and testing frequency correlations per bin, the effect of spatial pattern visible in Figures 5A, 8A is rather weak. Most locations do not show a big difference in frequency distributions of LCB (English and French) and of CS (+/–CS), especially in locations with higher frequency numbers. In the future, I will use methods to test spatial correlation in the data to see whether locations with preferences for English are clustered together (see Anselin, 1995 for Local Moran’s I).

This study has shown that there is considerably more English tweets than French tweets and considerably more Code-Switching in French tweets than in English tweets in Greater Montreal (see Figure 3). This observation suggests that language use on Twitter is not entirely predicted by population numbers of speech communities as shown in Figure 1 from Canadian statistics. The higher numbers of tweet counts in English in Figure 3 strongly indicate that English is the more dominant language on Twitter, which can be related to various factors such as English being used as *Lingua Franca* in social media (Laitinen and Lundberg, 2020). The observation of spatial patterns of CS-Engl. in cities of Quebec and spatial patterns of LCB and CS in Greater Montreal indicates that despite the dominance of English in the counts of tweets, the geographic context also influences language use to some extent, more strongly at city than sub-city level. This implies that the mechanisms of the digital and non-digital language contact are not the same.

In the future, I will test the influence of social contexts as defined by buildings or location contexts on CS and LCB. For instance, the airport has been shown to play an important role for +CS-Engl. (Figure 8B), but the airport is not the most important context for English tweets posted by bilinguals (Figure 5B). Instead, bilinguals tweet slightly more in French than in English at the airport. However, in order to compare similar contexts such as all coffee bars or airports requires information about the social use of buildings and urban districts. Lamarre et al. (2002) notice a difference in language use in formal/informal contexts by observing bilinguals’ language use as they move through the city. One could test the consequence of the difference between formal and informal contexts and CS or LCB in the future on the basis of tweets’ content. Formal and informal contexts usually correlate with different topics of discussion. Formal contexts often contain information of interest to the general public such as information about vaccination or elections. Informal contexts more often contain personal information of interest to particular user groups or people such as information about personal events or things. This hypothesis will be tested in the future on methods tailored for a topic analysis of tweets.

**Figure 14:**
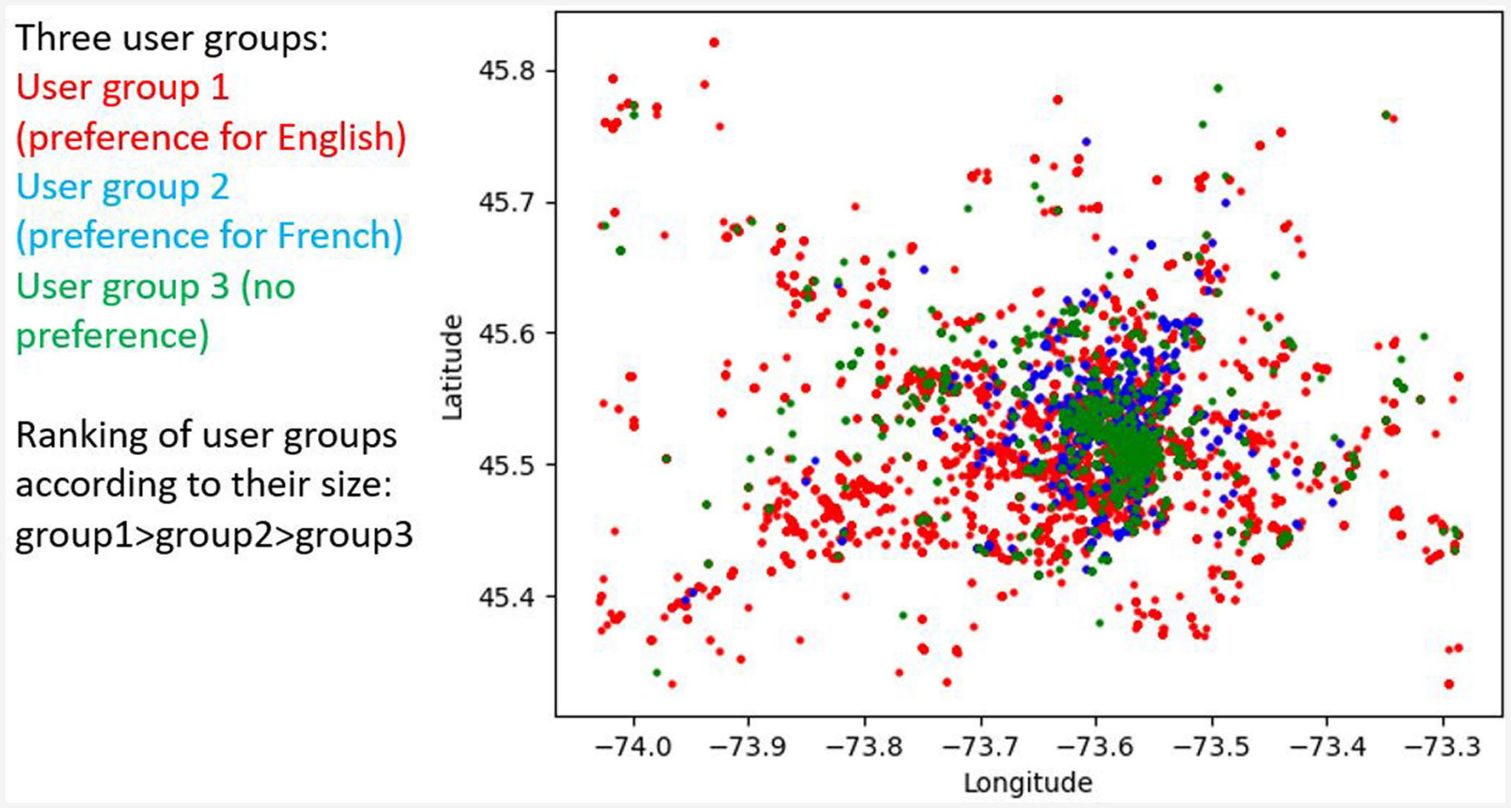
Distribution of English and French tweets per bilingual user group in GM. Tweets of bilingual users with preference for English (>90% tweets in English) in red, with preference for French in blue and with no preference in green.

Another topic that needs to be explored in the future is user variation with respect to CS and LCB. User IDs can be used to find all tweets produced by the same user and then to sort users according to the intensity of Code-Switching and/or their language preferences. This method allows us to find users who are resistant to CS or who use CS very frequently. The geolocation of tweets produced by a specific user group, such as a user group with a high preference for English or French or users without a language preference, allows us to classify these user groups with respect to the locations they visit. Figure 14 shows an analysis that explores user IDs to classify user groups according to their language preferences and to classify users by their visited location. The results show that users with the highest preference for English are dispersed throughout the city (marked by red points), whereas users with no language preference are concentrated in the city center (marked by green points). One tentative explanation for the concentration of users without a language preference is the assumption that the majority of users from the city center are entrepreneurs of some kind, who tweet in both languages to address as many clients as possible, including both French and English speakers. This hypothesis predicts that tweets from this user group should more often correspond to translations or paraphrases of the same content in different languages. This prediction can be tested by looking at temporal features of the tweets, assuming that they will very likely be posted at a similar time and by analyzing the content of the tweets. These methods will allow us to study ways of describing users and to contribute to sociolinguistic studies (Labov, 2006).

Another question that needs to be addressed in the future is how the factor “time” influences the distribution of language use. The present study measures the distribution of language use in a particular period, namely on the basis of tweets from 2017 to 2021. It is possible that users from Montreal pass their time in different locations depending on the period (year or day). However, if we compare the language distribution of bilinguals in Greater Montreal performed on the dataset used in this study with the language distribution of all users in Montreal performed by Mocanu et al. (2013) on an earlier dataset, we see a similar effect: English is used in the west and French is used in the east. This result suggests that the temporal difference in years does not matter much for language choice. In order to confirm this conclusion, though, other periods need to be considered.

Finally, more research needs to be done to investigate the extent to which bilingual users on Twitter represent bilingual speakers outside Twitter. It is also important to note that users allowing their geolocation to be seen publicly represent only a small proportion of all users on Twitter (Kruspe et al., 2021). It is thus quite possible that the location differences observed in this study are only representative for a particular user group on Twitter. In order to address this issue, various methods have been suggested in the research on Twitter (Kruspe et al., 2021 for an overview). The representativity of Twitter users allowing their geolocation to be visible will be investigated in the future.

To sum up, this study has shown that sociodemographic factors measured by a speech community’s size tend to influence CS and LCB on various spatial scales—the city and sub-city scales—but to a different extent. Geolocated tweets offer the possibility of studying the influence of location on language behavior. In order to maximize the benefit of social media data for sociolinguistic research, more techniques are needed to be able to analyze large quantities of data. This includes location classification by their social use and classification of tweets by user, topic, and language.

## Author’s note

The methodology of this article is based on OK’s previous work in Kellert and Matlis (2022).

## Data availability statement

The original contributions presented in the study are included in the article/Supplementary material, further inquiries can be directed to the corresponding author.

## Author contributions

OK: data collection, formulation of the hypotheses, design of testing the hypotheses, discussion of results, and article writing and editing.

## Funding

OK acknowledges the support given by the Open Access Publication Funds of the Göttingen University and by the German Research Foundation (DFG) (Grant number: 468416293).

## Conflict of interest

The author declares that the research was conducted in the absence of any commercial or financial relationships that could be construed as a potential conflict of interest.

## Publisher’s note

All claims expressed in this article are solely those of the authors and do not necessarily represent those of their affiliated organizations, or those of the publisher, the editors and the reviewers. Any product that may be evaluated in this article, or claim that may be made by its manufacturer, is not guaranteed or endorsed by the publisher.

## Footnotes

^1^I have anonymized names and place names that could be linked to an individual person.

^2^ https://blog.twitter.com/engineering/en_us/a/2015/evaluating-language-identification-performance
