# Supplementary material for: Probing sociodemographic influence on code-switching and language choice in Quebec with geolocation of tweets
Source: Front Psychol. 2023 May 2;14:1137038. doi: 10.3389/fpsyg.2023.1137038 (PMC10187760; doi:10.3389/fpsyg.2023.1137038)
Supplement: Supplementary file 1 [file Data_Sheet_1.PDF]

English words= [" is ", ' not ', ' and ', "with", 'day', "this", "time", ' our ', 'love', 'night', 'Happy', 'happy', 'food', 'like', 'new', 'dinner', 'winter', 'day', "Day", 'music', 'party', 'What', 'foodie', 'snow', 'here', 'Time', 'morning', 'appointment', ' gang ', 'Friday', 'but', 'week ', ' us ', 'game', 'life', 'city', 'great', 'sky', 'friends', 'car', 'coffee', 'ever', 'have', 'No ', ' no ', ' long ', 'Last', ' fall ', 'Kingdom', ' see ', 'first', ' can ', 'coming', 'ready', ' cat ', ' come ', 'Hello', 'off', 'spring', ' hot ', ' old ', ' soccer ', ' am ', ' visit ', 'Another', ' end ', ' vacation', ' dog ', 'delicious', 'Here', 'sweet', 'holiday', 'Lunch', 'evening', 'Hey', 'Check', 'Bye', ' hood ', 'soon', 'Today', 'Tonight', ' cute ', 'until', 'girl', 'big', 'than', 'Sales', 'bye', 'moving', 'need', ' ice ', 'Morning', 'available', ' event ', 'always', 'make', ' too ', 'movie', 'tree', 'clouds', ' kids ', 'Family', 'feet', ' pm ', 'weather', 'cheese', 'white', 'mood', 'room', ' real ', 'vegetable', ' take ', 'District', ' his ', 'these', 'happyness', 'very', 'going', 'Always', ' been ', 'around', 'drink', 'year', 'know', 'thank', 'afternoon', 'when', 'foodies', 'boys', 'water', 'outdoors', 'puppy', 'hours', 'Thursday', 'check', 'colors', 'chill', 'a lot', 'thing', 'potato', 'nothing', 'away', ' bit ', 'News', 'building', 'want', 'proud', ' she ', 'looking', 'without', 'together', 'friend', 'french', 'cake', 'Opening', 'blessed', 'Make', 'never', ' must ', 'babies', "baby", 'heart', ' eat ', 'gold', 'orders', 'meal', 'child', 'please', 'Stay', 'early', 'yeah', 'They', 'really', ' join ', 'cookies', 'Cookies', 'strong', "Enjoy", 'months', 'meet', "Meet", 'please']

French words= [' est ', ' pas ', ' et ', ' avec', ' il ', ' jour ', 'ceci', 'temps', ' notre', 'amour', 'nuit', 'dîner', 'heureux', 'heureuse', 'nourriture', 'comme ', 'nouveau', 'nouvelle', 'hiver', 'journée', 'debout', 'musique', 'fête', 'Quoi', 'manger', 'neige', ' ici ', 'temps', ' visite ', 'rencontre', 'groupe', 'vendredi', 'mais', 'semaine', ' nous ', 'jouet ', ' vie ', 'ville ', " ciel ", " amis ", " voiture ", " choisi ", " café ", " jamais ", " avoir ", " Non ", " non ", " longue ", " dernier ", " automne ", " royaume ", " voir ", " premier ", " peut ", " venir ", " prêt ", " chat ", " venir ", " bonjour ", " printemps ", " chaud ", "Vieux", "matin", 'visite', 'autre', ' fin ', 'vacances', 'chien', 'délicieux', ' ici ', 'doux', 'vacances', 'déjeuner', 'soirée', 'salut', 'Salut', 'chèque', 'au revoir', 'capot', 'bientôt', 'aujourd''hui', 'ce soir, mignon, jusqu''à..', 'fille', 'grand', 'plus', 'savoir', 'revoir', 'bouger', 'besoin', 'matin', 'disponible', 'evénement', 'toujours', 'faire', 'aussi', 'cinéman', 'arbre', 'nuages', 'enfants', 'famille', 'plaisir', 'pieds', 'après-midi', 'température', 'fromage', ' blanc', "humeur', 'chambre', 'réel', 'légumes', 'prendre', 'quartier', ' son ', ' ces ', 'bonheur', 'très', 'aller', 'toujours', 'être', 'autour', 'boisson', 'année', 'savoir', 'merci', 'après-midi', 'quand', 'gourmands', 'garçons', 'eau ', 'extérieur', chiot', 'heures', 'jeudi', 'contrôle', 'couleurs', 'froid', 'beaucoup', 'chose', 'patate', 'rien', ' loin ', ' peu ', 'nouvelles', 'bâtiment', 'vouloir', 'fier', 'elle', 'regarde', ' sans ', 'ensemble', ' ami ', ' amie ', 'français', 'gâteau', 'ouverture', béni ", " faire ", " jamais ", " doit ", " bébés ", " bébé ", " cœur ", " manger ", " commandes ", " repas ", " enfant ", " s'il vous plaît "]
