# Supplementary material for: Probing sociodemographic influence on code-switching and language choice in Quebec with geolocation of tweets
Source: Front Psychol. 2023 May 2;14:1137038. doi: 10.3389/fpsyg.2023.1137038 (PMC10187760; doi:10.3389/fpsyg.2023.1137038)
Supplement: Supplementary file 2 [file Data_Sheet_2.pdf]

Data_LCB_per bin in GM (longitude, latitude, sum, English tweets, French tweets)
[(-74.0237473, 45.32881036, 0, 0, 0), (-74.0088059, 45.32881036, 0, 0, 0), (-73.9938645, 45.32881036, 0, 0, 0), (-73.9789231, 45.32881036, 0, 0, 0), (-73.9639817, 45.32881036, 4, 1, 3), (-73.94904030000001, 45.32881036, 0, 0, 0), (-73.93409890000001, 45.32881036, 0, 0, 0), (-73.91915750000001, 45.32881036, 0, 0, 0), (-73.90421610000001, 45.32881036, 0, 0, 0), (-73.88927470000002, 45.32881036, 0, 0, 0), (-73.87433330000002, 45.32881036, 0, 0, 0), (-73.85939190000002, 45.32881036, 0, 0, 0), (-73.84445050000002, 45.32881036, 0, 0, 0), (-73.82950910000002, 45.32881036, 0, 0, 0), (-73.81456770000003, 45.32881036, 3, 1, 2), (-73.79962630000003, 45.32881036, 0, 0, 0), (-73.78468490000003, 45.32881036, 0, 0, 0), (-73.76974350000003, 45.32881036, 0, 0, 0), (-73.75480210000003, 45.32881036, 0, 0, 0), (-73.73986070000004, 45.32881036, 0, 0, 0), (-73.72491930000004, 45.32881036, 0, 0, 0), (-73.70997790000004, 45.32881036, 0, 0, 0), (-73.69503650000004, 45.32881036, 0, 0, 0), (-73.68009510000005, 45.32881036, 0, 0, 0), (-73.66515370000005, 45.32881036, 0, 0, 0), (-73.65021230000005, 45.32881036, 0, 0, 0), (-73.63527090000005, 45.32881036, 0, 0, 0), (-73.62032950000005, 45.32881036, 0, 0, 0), (-73.60538810000006, 45.32881036, 0, 0, 0), (-73.59044670000006, 45.32881036, 0, 0, 0), (-73.57550530000006, 45.32881036, 0, 0, 0), (-73.56056390000006, 45.32881036, 0, 0, 0), (-73.54562250000006, 45.32881036, 0, 0, 0), (-73.53068110000007, 45.32881036, 0, 0, 0), (-73.51573970000007, 45.32881036, 0, 0, 0), (-73.50079830000007, 45.32881036, 0, 0, 0), (-73.48585690000007, 45.32881036, 0, 0, 0), (-73.47091550000007, 45.32881036, 0, 0, 0), (-73.45597410000008, 45.32881036, 0, 0, 0), (-73.44103270000008, 45.32881036, 0, 0, 0), (-73.42609130000008, 45.32881036, 0, 0, 0), (-73.41114990000008, 45.32881036, 0, 0, 0), (-73.39620850000009, 45.32881036, 0, 0, 0), (-73.38126710000009, 45.32881036, 0, 0, 0), (-73.36632570000009, 45.32881036, 0, 0, 0), (-73.35138430000009, 45.32881036, 0, 0, 0), (-73.3364429000001, 45.32881036, 0, 0, 0), (-73.3215015000001, 45.32881036, 0, 0, 0), (-73.3065601000001, 45.32881036, 0, 0, 0), (-73.2916187000001, 45.32881036, 11, 0, 11), (-74.0237473, 45.33899908, 0, 0, 0), (-74.0088059, 45.33899908, 0, 0, 0), (-73.9938645, 45.33899908, 0, 0, 0), (-73.9789231, 45.33899908, 2, 0, 2), (-73.9639817, 45.33899908, 1, 0, 1), (-73.94904030000001, 45.33899908, 0, 0, 0), (-73.93409890000001, 45.33899908, 0, 0, 0), (-73.91915750000001, 45.33899908, 0, 0, 0), (-73.90421610000001, 45.33899908, 0, 0, 0), (-73.88927470000002, 45.33899908, 0, 0, 0), (-73.87433330000002, 45.33899908, 0, 0, 0), (-73.85939190000002, 45.33899908, 0, 0, 0), (-73.84445050000002, 45.33899908, 0, 0, 0), (-73.82950910000002, 45.33899908, 0, 0, 0), (-73.81456770000003, 45.33899908, 0, 0, 0), (-73.79962630000003, 45.33899908, 0, 0, 0), (-73.78468490000003, 45.33899908, 0, 0, 0), (-73.76974350000003, 45.33899908, 4, 0, 4), (-73.75480210000003, 45.33899908, 0, 0, 0), (-73.73986070000004, 45.33899908, 0, 0, 0), (-73.72491930000004, 45.33899908, 3, 1, 2), (-73.70997790000004, 45.33899908, 0, 0, 0), (-73.69503650000004, 45.33899908, 0, 0, 0), (-73.68009510000005, 45.33899908, 0, 0, 0), (-73.66515370000005, 45.33899908, 0, 0, 0), (-73.65021230000005, 45.33899908, 0, 0, 0), (-73.63527090000005, 45.33899908, 0, 0, 0), (-73.62032950000005, 45.33899908, 0, 0, 0), (-73.60538810000006, 45.33899908, 0, 0, 0), (-73.59044670000006, 45.33899908, 0, 0, 0), (-73.57550530000006, 45.33899908, 0, 0, 0), (-73.56056390000006, 45.33899908, 0, 0, 0), (-73.54562250000006, 45.33899908, 0, 0, 0), (-73.53068110000007, 45.33899908, 0, 0, 0), (-73.51573970000007, 45.33899908, 1, 0, 1), (-73.50079830000007, 45.33899908, 0, 0, 0), (-73.48585690000007, 45.33899908, 0, 0, 0), (-73.47091550000007, 45.33899908, 0, 0, 0), (-73.45597410000008, 45.33899908, 0, 0, 0), (-73.44103270000008, 45.33899908, 0, 0, 0), (-73.42609130000008, 45.33899908, 0, 0, 0), (-73.41114990000008, 45.33899908, 0, 0, 0), (-73.39620850000009, 45.33899908, 0, 0, 0), (-73.38126710000009, 45.33899908, 0, 0, 0), (-73.36632570000009, 45.33899908, 0, 0, 0), (-73.35138430000009, 45.33899908, 0, 0, 0), (-73.3364429000001, 45.33899908, 0, 0, 0), (-73.3215015000001, 45.33899908, 0, 0, 0), (-73.3065601000001, 45.33899908, 1, 0, 1), (-73.2916187000001, 45.33899908, 1, 1, 0), (-74.0237473, 45.3491878, 0, 0, 0), (-74.0088059, 45.3491878, 0, 0, 0), (-73.9938645, 45.3491878,

0, 0, 0), (-73.9789231, 45.3491878, 0, 0, 0), (-73.9639817, 45.3491878, 0, 0, 0), (-73.94904030000001, 45.3491878, 0, 0, 0), (-73.93409890000001, 45.3491878, 0, 0, 0), (-73.91915750000001, 45.3491878, 0, 0, 0), (-73.90421610000001, 45.3491878, 30, 18, 12), (-73.88927470000002, 45.3491878, 0, 0, 0), (-73.87433330000002, 45.3491878, 0, 0, 0), (-73.85939190000002, 45.3491878, 0, 0, 0), (-73.84445050000002, 45.3491878, 0, 0, 0), (-73.82950910000002, 45.3491878, 0, 0, 0), (-73.81456770000003, 45.3491878, 0, 0, 0), (-73.79962630000003, 45.3491878, 1, 1, 0), (-73.78468490000003, 45.3491878, 0, 0, 0), (-73.76974350000003, 45.3491878, 3, 3, 0), (-73.75480210000003, 45.3491878, 0, 0, 0), (-73.73986070000004, 45.3491878, 1, 0, 1), (-73.72491930000004, 45.3491878, 2, 0, 2), (-73.70997790000004, 45.3491878, 1, 0, 1), (-73.69503650000004, 45.3491878, 2, 2, 0), (-73.68009510000005, 45.3491878, 0, 0, 0), (-73.66515370000005, 45.3491878, 0, 0, 0), (-73.65021230000005, 45.3491878, 0, 0, 0), (-73.63527090000005, 45.3491878, 0, 0, 0), (-73.62032950000005, 45.3491878, 0, 0, 0), (-73.60538810000006, 45.3491878, 0, 0, 0), (-73.59044670000006, 45.3491878, 0, 0, 0), (-73.57550530000006, 45.3491878, 0, 0, 0), (-73.56056390000006, 45.3491878, 0, 0, 0), (-73.54562250000006, 45.3491878, 0, 0, 0), (-73.53068110000007, 45.3491878, 0, 0, 0), (-73.51573970000007, 45.3491878, 0, 0, 0), (-73.50079830000007, 45.3491878, 0, 0, 0), (-73.48585690000007, 45.3491878, 0, 0, 0), (-73.47091550000007, 45.3491878, 7, 6, 1), (-73.45597410000008, 45.3491878, 0, 0, 0), (-73.44103270000008, 45.3491878, 0, 0, 0), (-73.42609130000008, 45.3491878, 0, 0, 0), (-73.41114990000008, 45.3491878, 0, 0, 0), (-73.39620850000009, 45.3491878, 0, 0, 0), (-73.38126710000009, 45.3491878, 0, 0, 0), (-73.36632570000009, 45.3491878, 0, 0, 0), (-73.35138430000009, 45.3491878, 0, 0, 0), (-73.3364429000001, 45.3491878, 0, 0, 0), (-73.3215015000001, 45.3491878, 0, 0, 0), (-73.3065601000001, 45.3491878, 2, 2, 0), (-73.2916187000001, 45.3491878, 2, 2, 0), (-74.0237473, 45.359376520000005, 0, 0, 0), (-74.0088059, 45.359376520000005, 0, 0, 0), (-73.9938645, 45.359376520000005, 0, 0, 0), (-73.9789231, 45.359376520000005, 0, 0, 0), (-73.9639817, 45.359376520000005, 0, 0, 0), (-73.94904030000001, 45.359376520000005, 0, 0, 0), (-73.93409890000001, 45.359376520000005, 1, 1, 0), (-73.91915750000001, 45.359376520000005, 22, 5, 17), (-73.90421610000001, 45.359376520000005, 1, 0, 1), (-73.88927470000002, 45.359376520000005, 0, 0, 0), (-73.87433330000002, 45.359376520000005, 0, 0, 0), (-73.85939190000002, 45.359376520000005, 0, 0, 0), (-73.84445050000002, 45.359376520000005, 0, 0, 0), (-73.82950910000002, 45.359376520000005, 0, 0, 0), (-73.81456770000003, 45.359376520000005, 3, 3, 0), (-73.79962630000003, 45.359376520000005, 0, 0, 0), (-73.78468490000003, 45.359376520000005, 0, 0, 0), (-73.76974350000003, 45.359376520000005, 0, 0, 0), (-73.75480210000003, 45.359376520000005, 76, 25, 51), (-73.73986070000004, 45.359376520000005, 9, 9, 0), (-73.72491930000004, 45.359376520000005, 17, 7, 10), (-73.70997790000004, 45.359376520000005, 8, 0, 8), (-73.69503650000004, 45.359376520000005, 0, 0, 0), (-73.68009510000005, 45.359376520000005, 0, 0, 0), (-73.66515370000005, 45.359376520000005, 0, 0, 0), (-73.65021230000005, 45.359376520000005, 0, 0, 0), (-73.63527090000005, 45.359376520000005, 0, 0, 0), (-73.62032950000005, 45.359376520000005, 0, 0, 0), (-73.60538810000006, 45.359376520000005, 0, 0, 0), (-73.59044670000006, 45.359376520000005, 0, 0, 0), (-73.57550530000006, 45.359376520000005, 0, 0, 0), (-73.56056390000006, 45.359376520000005, 0, 0, 0), (-73.54562250000006, 45.359376520000005, 0, 0, 0), (-73.53068110000007, 45.359376520000005, 0, 0, 0), (-73.51573970000007, 45.359376520000005, 0, 0, 0), (-73.50079830000007, 45.359376520000005, 0, 0, 0), (-73.48585690000007, 45.359376520000005, 0, 0, 0), (-73.47091550000007, 45.359376520000005, 0, 0, 0), (-73.45597410000008, 45.359376520000005, 0, 0, 0), (-73.44103270000008, 45.359376520000005, 0, 0, 0), (-73.42609130000008, 45.359376520000005, 0, 0, 0), (-73.41114990000008, 45.359376520000005, 0, 0, 0), (-73.39620850000009,

45.359376520000005, 0, 0, 0), (-73.38126710000009, 45.359376520000005, 0, 0, 0), (-73.36632570000009, 45.359376520000005, 0, 0, 0), (-73.35138430000009, 45.359376520000005, 0, 0, 0), (-73.3364429000001, 45.359376520000005, 0, 0, 0), (-73.3215015000001, 45.359376520000005, 0, 0, 0), (-73.3065601000001, 45.359376520000005, 2, 1, 1), (-73.2916187000001, 45.359376520000005, 4, 4, 0), (-74.0237473, 45.36956524000001, 2, 2, 0), (-74.0088059, 45.36956524000001, 0, 0, 0), (-73.9938645, 45.36956524000001, 1, 0, 1), (-73.9789231, 45.36956524000001, 96, 11, 85), (-73.9639817, 45.36956524000001, 0, 0, 0), (-73.94904030000001, 45.36956524000001, 0, 0, 0), (-73.93409890000001, 45.36956524000001, 15, 13, 2), (-73.91915750000001, 45.36956524000001, 0, 0, 0), (-73.90421610000001, 45.36956524000001, 1, 1, 0), (-73.88927470000002, 45.36956524000001, 0, 0, 0), (-73.87433330000002, 45.36956524000001, 3, 2, 1), (-73.85939190000002, 45.36956524000001, 4, 0, 4), (-73.84445050000002, 45.36956524000001, 3, 0, 3), (-73.82950910000002, 45.36956524000001, 0, 0, 0), (-73.81456770000003, 45.36956524000001, 0, 0, 0), (-73.79962630000003, 45.36956524000001, 0, 0, 0), (-73.78468490000003, 45.36956524000001, 0, 0, 0), (-73.76974350000003, 45.36956524000001, 0, 0, 0), (-73.75480210000003, 45.36956524000001, 4, 3, 1), (-73.73986070000004, 45.36956524000001, 1, 0, 1), (-73.72491930000004, 45.36956524000001, 0, 0, 0), (-73.70997790000004, 45.36956524000001, 1, 0, 1), (-73.69503650000004, 45.36956524000001, 0, 0, 0), (-73.68009510000005, 45.36956524000001, 0, 0, 0), (-73.66515370000005, 45.36956524000001, 0, 0, 0), (-73.65021230000005, 45.36956524000001, 1, 0, 1), (-73.63527090000005, 45.36956524000001, 0, 0, 0), (-73.62032950000005, 45.36956524000001, 0, 0, 0), (-73.60538810000006, 45.36956524000001, 0, 0, 0), (-73.59044670000006, 45.36956524000001, 0, 0, 0), (-73.57550530000006, 45.36956524000001, 49, 28, 21), (-73.56056390000006, 45.36956524000001, 34, 26, 8), (-73.54562250000006, 45.36956524000001, 109, 2, 107), (-73.53068110000007, 45.36956524000001, 2, 0, 2), (-73.51573970000007, 45.36956524000001, 4, 0, 4), (-73.50079830000007, 45.36956524000001, 1, 0, 1), (-73.48585690000007, 45.36956524000001, 0, 0, 0), (-73.47091550000007, 45.36956524000001, 0, 0, 0), (-73.45597410000008, 45.36956524000001, 0, 0, 0), (-73.44103270000008, 45.36956524000001, 0, 0, 0), (-73.42609130000008, 45.36956524000001, 0, 0, 0), (-73.41114990000008, 45.36956524000001, 0, 0, 0), (-73.39620850000009, 45.36956524000001, 0, 0, 0), (-73.38126710000009, 45.36956524000001, 0, 0, 0), (-73.36632570000009, 45.36956524000001, 0, 0, 0), (-73.35138430000009, 45.36956524000001, 0, 0, 0), (-73.3364429000001, 45.36956524000001, 0, 0, 0), (-73.3215015000001, 45.36956524000001, 0, 0, 0), (-73.3065601000001, 45.36956524000001, 0, 0, 0), (-73.2916187000001, 45.36956524000001, 0, 0, 0), (-74.0237473, 45.37975396000001, 53, 16, 37), (-74.0088059, 45.37975396000001, 35, 1, 34), (-73.9938645, 45.37975396000001, 9, 2, 7), (-73.9789231, 45.37975396000001, 3, 3, 0), (-73.9639817, 45.37975396000001, 0, 0, 0), (-73.94904030000001, 45.37975396000001, 7, 0, 7), (-73.93409890000001, 45.37975396000001, 0, 0, 0), (-73.91915750000001, 45.37975396000001, 0, 0, 0), (-73.90421610000001, 45.37975396000001, 0, 0, 0), (-73.88927470000002, 45.37975396000001, 0, 0, 0), (-73.87433330000002, 45.37975396000001, 0, 0, 0), (-73.85939190000002, 45.37975396000001, 0, 0, 0), (-73.84445050000002, 45.37975396000001, 0, 0, 0), (-73.82950910000002, 45.37975396000001, 0, 0, 0), (-73.81456770000003, 45.37975396000001, 0, 0, 0), (-73.79962630000003, 45.37975396000001, 0, 0, 0), (-73.78468490000003, 45.37975396000001, 0, 0, 0), (-73.76974350000003, 45.37975396000001, 3, 1, 2), (-73.75480210000003, 45.37975396000001, 173, 149, 24), (-73.73986070000004, 45.37975396000001, 1, 0, 1), (-73.72491930000004, 45.37975396000001, 0, 0, 0), (-73.70997790000004, 45.37975396000001, 16, 2, 14), (-73.69503650000004, 45.37975396000001, 0, 0, 0), (-73.68009510000005, 45.37975396000001, 0, 0, 0), (-73.66515370000005, 45.37975396000001, 0, 0, 0), (-73.65021230000005, 45.37975396000001, 0, 0, 0), (-73.63527090000005, 45.37975396000001, 0, 0, 0), (-73.62032950000005, 45.37975396000001, 0, 0, 0), (-73.60538810000006, 45.37975396000001, 1, 0, 1),

(-73.59044670000006, 45.37975396000001, 1, 1, 0), (-73.57550530000006, 45.37975396000001, 1, 0, 1), (-73.56056390000006, 45.37975396000001, 35, 14, 21), (-73.54562250000006, 45.37975396000001, 15, 4, 11), (-73.53068110000007, 45.37975396000001, 4, 1, 3), (-73.51573970000007, 45.37975396000001, 192, 13, 179), (-73.50079830000007, 45.37975396000001, 7, 2, 5), (-73.48585690000007, 45.37975396000001, 2, 0, 2), (-73.47091550000007, 45.37975396000001, 0, 0, 0), (-73.45597410000008, 45.37975396000001, 0, 0, 0), (-73.44103270000008, 45.37975396000001, 0, 0, 0), (-73.42609130000008, 45.37975396000001, 0, 0, 0), (-73.41114990000008, 45.37975396000001, 0, 0, 0), (-73.39620850000009, 45.37975396000001, 0, 0, 0), (-73.38126710000009, 45.37975396000001, 0, 0, 0), (-73.36632570000009, 45.37975396000001, 0, 0, 0), (-73.35138430000009, 45.37975396000001, 0, 0, 0), (-73.3364429000001, 45.37975396000001, 0, 0, 0), (-73.3215015000001, 45.37975396000001, 0, 0, 0), (-73.3065601000001, 45.37975396000001, 0, 0, 0), (-73.2916187000001, 45.37975396000001, 0, 0, 0), (-74.0237473, 45.38994268000001, 31, 7, 24), (-74.0088059, 45.38994268000001, 8, 2, 6), (-73.9938645, 45.38994268000001, 2, 0, 2), (-73.9789231, 45.38994268000001, 16, 7, 9), (-73.9639817, 45.38994268000001, 32, 2, 30), (-73.94904030000001, 45.38994268000001, 12, 0, 12), (-73.93409890000001, 45.38994268000001, 1, 0, 1), (-73.91915750000001, 45.38994268000001, 0, 0, 0), (-73.90421610000001, 45.38994268000001, 11, 8, 3), (-73.88927470000002, 45.38994268000001, 0, 0, 0), (-73.87433330000002, 45.38994268000001, 0, 0, 0), (-73.85939190000002, 45.38994268000001, 0, 0, 0), (-73.84445050000002, 45.38994268000001, 0, 0, 0), (-73.82950910000002, 45.38994268000001, 0, 0, 0), (-73.81456770000003, 45.38994268000001, 0, 0, 0), (-73.79962630000003, 45.38994268000001, 0, 0, 0), (-73.78468490000003, 45.38994268000001, 0, 0, 0), (-73.76974350000003, 45.38994268000001, 5, 5, 0), (-73.75480210000003, 45.38994268000001, 12, 8, 4), (-73.73986070000004, 45.38994268000001, 0, 0, 0), (-73.72491930000004, 45.38994268000001, 0, 0, 0), (-73.70997790000004, 45.38994268000001, 0, 0, 0), (-73.69503650000004, 45.38994268000001, 2, 0, 2), (-73.68009510000005, 45.38994268000001, 0, 0, 0), (-73.66515370000005, 45.38994268000001, 1, 0, 1), (-73.65021230000005, 45.38994268000001, 0, 0, 0), (-73.63527090000005, 45.38994268000001, 0, 0, 0), (-73.62032950000005, 45.38994268000001, 0, 0, 0), (-73.60538810000006, 45.38994268000001, 0, 0, 0), (-73.59044670000006, 45.38994268000001, 1, 0, 1), (-73.57550530000006, 45.38994268000001, 3, 2, 1), (-73.56056390000006, 45.38994268000001, 5, 0, 5), (-73.54562250000006, 45.38994268000001, 37, 2, 35), (-73.53068110000007, 45.38994268000001, 0, 0, 0), (-73.51573970000007, 45.38994268000001, 1, 0, 1), (-73.50079830000007, 45.38994268000001, 3, 1, 2), (-73.48585690000007, 45.38994268000001, 3, 3, 0), (-73.47091550000007, 45.38994268000001, 1, 1, 0), (-73.45597410000008, 45.38994268000001, 0, 0, 0), (-73.44103270000008, 45.38994268000001, 0, 0, 0), (-73.42609130000008, 45.38994268000001, 0, 0, 0), (-73.41114990000008, 45.38994268000001, 0, 0, 0), (-73.39620850000009, 45.38994268000001, 0, 0, 0), (-73.38126710000009, 45.38994268000001, 0, 0, 0), (-73.36632570000009, 45.38994268000001, 0, 0, 0), (-73.35138430000009, 45.38994268000001, 0, 0, 0), (-73.3364429000001, 45.38994268000001, 0, 0, 0), (-73.3215015000001, 45.38994268000001, 0, 0, 0), (-73.3065601000001, 45.38994268000001, 0, 0, 0), (-73.2916187000001, 45.38994268000001, 0, 0, 0), (-74.0237473, 45.400131400000014, 12, 2, 10), (-74.0088059, 45.400131400000014, 0, 0, 0), (-73.9938645, 45.400131400000014, 0, 0, 0), (-73.9789231, 45.400131400000014, 2, 2, 0), (-73.9639817, 45.400131400000014, 65, 15, 50), (-73.94904030000001, 45.400131400000014, 197, 57, 140), (-73.93409890000001, 45.400131400000014, 11, 4, 7), (-73.91915750000001, 45.400131400000014, 10, 5, 5), (-73.90421610000001, 45.400131400000014, 0, 0, 0), (-73.88927470000002, 45.400131400000014, 0, 0, 0), (-73.87433330000002, 45.400131400000014, 0, 0, 0), (-73.85939190000002, 45.400131400000014, 0, 0, 0), (-73.84445050000002, 45.400131400000014, 0, 0, 0), (-73.82950910000002, 45.400131400000014, 0, 0, 0), (-73.81456770000003, 45.400131400000014, 8, 3, 5),

(-73.79962630000003, 45.400131400000014, 0, 0, 0), (-73.78468490000003, 45.400131400000014, 0, 0, 0), (-73.76974350000003, 45.400131400000014, 0, 0, 0), (-73.75480210000003, 45.400131400000014, 0, 0, 0), (-73.73986070000004, 45.400131400000014, 0, 0, 0), (-73.72491930000004, 45.400131400000014, 0, 0, 0), (-73.70997790000004, 45.400131400000014, 0, 0, 0), (-73.69503650000004, 45.400131400000014, 3, 0, 3), (-73.68009510000005, 45.400131400000014, 64, 0, 64), (-73.66515370000005, 45.400131400000014, 1, 0, 1), (-73.65021230000005, 45.400131400000014, 0, 0, 0), (-73.63527090000005, 45.400131400000014, 1, 0, 1), (-73.62032950000005, 45.400131400000014, 1, 0, 1), (-73.60538810000006, 45.400131400000014, 0, 0, 0), (-73.59044670000006, 45.400131400000014, 17, 9, 8), (-73.57550530000006, 45.400131400000014, 1, 1, 0), (-73.56056390000006, 45.400131400000014, 229, 4, 225), (-73.54562250000006, 45.400131400000014, 6, 4, 2), (-73.53068110000007, 45.400131400000014, 5, 2, 3), (-73.51573970000007, 45.400131400000014, 118, 43, 75), (-73.50079830000007, 45.400131400000014, 70, 8, 62), (-73.48585690000007, 45.400131400000014, 2, 0, 2), (-73.47091550000007, 45.400131400000014, 7, 6, 1), (-73.45597410000008, 45.400131400000014, 0, 0, 0), (-73.44103270000008, 45.400131400000014, 0, 0, 0), (-73.42609130000008, 45.400131400000014, 0, 0, 0), (-73.41114990000008, 45.400131400000014, 0, 0, 0), (-73.39620850000009, 45.400131400000014, 0, 0, 0), (-73.38126710000009, 45.400131400000014, 0, 0, 0), (-73.36632570000009, 45.400131400000014, 0, 0, 0), (-73.35138430000009, 45.400131400000014, 0, 0, 0), (-73.3364429000001, 45.400131400000014, 0, 0, 0), (-73.3215015000001, 45.400131400000014, 0, 0, 0), (-73.3065601000001, 45.400131400000014, 0, 0, 0), (-73.2916187000001, 45.400131400000014, 0, 0, 0), (-74.0237473, 45.410320120000016, 3, 0, 3), (-74.0088059, 45.410320120000016, 5, 3, 2), (-73.9938645, 45.410320120000016, 0, 0, 0), (-73.9789231, 45.410320120000016, 0, 0, 0), (-73.9639817, 45.410320120000016, 11, 3, 8), (-73.94904030000001, 45.410320120000016, 64, 8, 56), (-73.93409890000001, 45.410320120000016, 24, 0, 24), (-73.91915750000001, 45.410320120000016, 20, 2, 18), (-73.90421610000001, 45.410320120000016, 1, 0, 1), (-73.88927470000002, 45.410320120000016, 2, 1, 1), (-73.87433330000002, 45.410320120000016, 0, 0, 0), (-73.85939190000002, 45.410320120000016, 0, 0, 0), (-73.84445050000002, 45.410320120000016, 0, 0, 0), (-73.82950910000002, 45.410320120000016, 0, 0, 0), (-73.81456770000003, 45.410320120000016, 0, 0, 0), (-73.79962630000003, 45.410320120000016, 0, 0, 0), (-73.78468490000003, 45.410320120000016, 0, 0, 0), (-73.76974350000003, 45.410320120000016, 0, 0, 0), (-73.75480210000003, 45.410320120000016, 0, 0, 0), (-73.73986070000004, 45.410320120000016, 0, 0, 0), (-73.72491930000004, 45.410320120000016, 0, 0, 0), (-73.70997790000004, 45.410320120000016, 8, 0, 8), (-73.69503650000004, 45.410320120000016, 1, 0, 1), (-73.68009510000005, 45.410320120000016, 16, 2, 14), (-73.66515370000005, 45.410320120000016, 10, 0, 10), (-73.65021230000005, 45.410320120000016, 0, 0, 0), (-73.63527090000005, 45.410320120000016, 1, 0, 1), (-73.62032950000005, 45.410320120000016, 0, 0, 0), (-73.60538810000006, 45.410320120000016, 0, 0, 0), (-73.59044670000006, 45.410320120000016, 0, 0, 0), (-73.57550530000006, 45.410320120000016, 5, 4, 1), (-73.56056390000006, 45.410320120000016, 1, 1, 0), (-73.54562250000006, 45.410320120000016, 0, 0, 0), (-73.53068110000007, 45.410320120000016, 0, 0, 0), (-73.51573970000007, 45.410320120000016, 0, 0, 0), (-73.50079830000007, 45.410320120000016, 2, 1, 1), (-73.48585690000007, 45.410320120000016, 2, 2, 0), (-73.47091550000007, 45.410320120000016, 12, 6, 6), (-73.45597410000008, 45.410320120000016, 0, 0, 0), (-73.44103270000008, 45.410320120000016, 0, 0, 0), (-73.42609130000008, 45.410320120000016, 0, 0, 0), (-73.41114990000008, 45.410320120000016, 0, 0, 0), (-73.39620850000009, 45.410320120000016, 0, 0, 0), (-73.38126710000009,

45.410320120000016, 0, 0, 0), (-73.36632570000009, 45.410320120000016, 0, 0, 0), (-73.35138430000009, 45.410320120000016, 0, 0, 0), (-73.3364429000001, 45.410320120000016, 0, 0, 0), (-73.3215015000001, 45.410320120000016, 0, 0, 0), (-73.3065601000001, 45.410320120000016, 9, 4, 5), (-73.2916187000001, 45.410320120000016, 1, 0, 1), (-74.0237473, 45.42050884000002, 14, 4, 10), (-74.0088059, 45.42050884000002, 16, 9, 7), (-73.9938645, 45.42050884000002, 0, 0, 0), (-73.9789231, 45.42050884000002, 0, 0, 0), (-73.9639817, 45.42050884000002, 0, 0, 0), (-73.94904030000001, 45.42050884000002, 0, 0, 0), (-73.93409890000001, 45.42050884000002, 15, 1, 14), (-73.91915750000001, 45.42050884000002, 115, 12, 103), (-73.90421610000001, 45.42050884000002, 1, 1, 0), (-73.88927470000002, 45.42050884000002, 68, 1, 67), (-73.87433330000002, 45.42050884000002, 392, 9, 383), (-73.85939190000002, 45.42050884000002, 96, 1, 95), (-73.84445050000002, 45.42050884000002, 0, 0, 0), (-73.82950910000002, 45.42050884000002, 1, 0, 1), (-73.81456770000003, 45.42050884000002, 0, 0, 0), (-73.79962630000003, 45.42050884000002, 0, 0, 0), (-73.78468490000003, 45.42050884000002, 0, 0, 0), (-73.76974350000003, 45.42050884000002, 0, 0, 0), (-73.75480210000003, 45.42050884000002, 0, 0, 0), (-73.73986070000004, 45.42050884000002, 0, 0, 0), (-73.72491930000004, 45.42050884000002, 0, 0, 0), (-73.70997790000004, 45.42050884000002, 0, 0, 0), (-73.69503650000004, 45.42050884000002, 0, 0, 0), (-73.68009510000005, 45.42050884000002, 0, 0, 0), (-73.66515370000005, 45.42050884000002, 0, 0, 0), (-73.65021230000005, 45.42050884000002, 309, 48, 261), (-73.63527090000005, 45.42050884000002, 69, 3, 66), (-73.62032950000005, 45.42050884000002, 7, 5, 2), (-73.60538810000006, 45.42050884000002, 11, 4, 7), (-73.59044670000006, 45.42050884000002, 0, 0, 0), (-73.57550530000006, 45.42050884000002, 0, 0, 0), (-73.56056390000006, 45.42050884000002, 0, 0, 0), (-73.54562250000006, 45.42050884000002, 0, 0, 0), (-73.53068110000007, 45.42050884000002, 0, 0, 0), (-73.51573970000007, 45.42050884000002, 0, 0, 0), (-73.50079830000007, 45.42050884000002, 16, 6, 10), (-73.48585690000007, 45.42050884000002, 121, 37, 84), (-73.47091550000007, 45.42050884000002, 9, 4, 5), (-73.45597410000008, 45.42050884000002, 0, 0, 0), (-73.44103270000008, 45.42050884000002, 0, 0, 0), (-73.42609130000008, 45.42050884000002, 0, 0, 0), (-73.41114990000008, 45.42050884000002, 0, 0, 0), (-73.39620850000009, 45.42050884000002, 0, 0, 0), (-73.38126710000009, 45.42050884000002, 0, 0, 0), (-73.36632570000009, 45.42050884000002, 0, 0, 0), (-73.35138430000009, 45.42050884000002, 0, 0, 0), (-73.3364429000001, 45.42050884000002, 0, 0, 0), (-73.3215015000001, 45.42050884000002, 0, 0, 0), (-73.3065601000001, 45.42050884000002, 0, 0, 0), (-73.2916187000001, 45.42050884000002, 0, 0, 0), (-74.0237473, 45.43069756000002, 0, 0, 0), (-74.0088059, 45.43069756000002, 0, 0, 0), (-73.9938645, 45.43069756000002, 0, 0, 0), (-73.9789231, 45.43069756000002, 0, 0, 0), (-73.9639817, 45.43069756000002, 3, 2, 1), (-73.94904030000001, 45.43069756000002, 1, 0, 1), (-73.93409890000001, 45.43069756000002, 7, 0, 7), (-73.91915750000001, 45.43069756000002, 2, 0, 2), (-73.90421610000001, 45.43069756000002, 1, 1, 0), (-73.88927470000002, 45.43069756000002, 3, 0, 3), (-73.87433330000002, 45.43069756000002, 1876, 164, 1712), (-73.85939190000002, 45.43069756000002, 10, 0, 10), (-73.84445050000002, 45.43069756000002, 23, 1, 22), (-73.82950910000002, 45.43069756000002, 118, 9, 109), (-73.81456770000003, 45.43069756000002, 37, 3, 34), (-73.79962630000003, 45.43069756000002, 0, 0, 0), (-73.78468490000003, 45.43069756000002, 0, 0, 0), (-73.76974350000003, 45.43069756000002, 0, 0, 0), (-73.75480210000003, 45.43069756000002, 105, 10, 95), (-73.73986070000004, 45.43069756000002, 0, 0, 0), (-73.72491930000004, 45.43069756000002, 0, 0, 0), (-73.70997790000004, 45.43069756000002, 1, 0, 1), (-73.69503650000004, 45.43069756000002, 35, 14, 21), (-73.68009510000005, 45.43069756000002, 141, 82, 59), (-73.66515370000005, 45.43069756000002, 77, 31, 46), (-73.65021230000005, 45.43069756000002, 25, 12, 13), (-73.63527090000005, 45.43069756000002, 212, 40, 172), (-73.62032950000005, 45.43069756000002, 327, 124, 203), (-73.60538810000006, 45.43069756000002, 26, 6, 20),

(-73.59044670000006, 45.43069756000002, 145, 17, 128), (-73.57550530000006, 45.43069756000002, 1, 0, 1), (-73.56056390000006, 45.43069756000002, 0, 0, 0), (-73.54562250000006, 45.43069756000002, 0, 0, 0), (-73.53068110000007, 45.43069756000002, 0, 0, 0), (-73.51573970000007, 45.43069756000002, 0, 0, 0), (-73.50079830000007, 45.43069756000002, 0, 0, 0), (-73.48585690000007, 45.43069756000002, 4, 2, 2), (-73.47091550000007, 45.43069756000002, 4, 2, 2), (-73.45597410000008, 45.43069756000002, 349, 0, 349), (-73.44103270000008, 45.43069756000002, 8, 1, 7), (-73.42609130000008, 45.43069756000002, 0, 0, 0), (-73.41114990000008, 45.43069756000002, 0, 0, 0), (-73.39620850000009, 45.43069756000002, 0, 0, 0), (-73.38126710000009, 45.43069756000002, 0, 0, 0), (-73.36632570000009, 45.43069756000002, 0, 0, 0), (-73.35138430000009, 45.43069756000002, 0, 0, 0), (-73.3364429000001, 45.43069756000002, 0, 0, 0), (-73.3215015000001, 45.43069756000002, 2, 0, 2), (-73.3065601000001, 45.43069756000002, 22, 7, 15), (-73.2916187000001, 45.43069756000002, 34, 17, 17), (-74.0237473, 45.44088628000002, 0, 0, 0), (-74.0088059, 45.44088628000002, 0, 0, 0), (-73.9938645, 45.44088628000002, 0, 0, 0), (-73.9789231, 45.44088628000002, 0, 0, 0), (-73.9639817, 45.44088628000002, 0, 0, 0), (-73.94904030000001, 45.44088628000002, 0, 0, 0), (-73.93409890000001, 45.44088628000002, 0, 0, 0), (-73.91915750000001, 45.44088628000002, 2, 2, 0), (-73.90421610000001, 45.44088628000002, 0, 0, 0), (-73.88927470000002, 45.44088628000002, 26, 6, 20), (-73.87433330000002, 45.44088628000002, 1, 0, 1), (-73.85939190000002, 45.44088628000002, 6, 0, 6), (-73.84445050000002, 45.44088628000002, 0, 0, 0), (-73.82950910000002, 45.44088628000002, 5, 1, 4), (-73.81456770000003, 45.44088628000002, 79, 30, 49), (-73.79962630000003, 45.44088628000002, 9, 1, 8), (-73.78468490000003, 45.44088628000002, 2, 0, 2), (-73.76974350000003, 45.44088628000002, 59, 0, 59), (-73.75480210000003, 45.44088628000002, 39, 7, 32), (-73.73986070000004, 45.44088628000002, 103, 14, 89), (-73.72491930000004, 45.44088628000002, 621, 20, 601), (-73.70997790000004, 45.44088628000002, 9, 5, 4), (-73.69503650000004, 45.44088628000002, 1450, 192, 1258), (-73.68009510000005, 45.44088628000002, 92, 24, 68), (-73.66515370000005, 45.44088628000002, 22, 9, 13), (-73.65021230000005, 45.44088628000002, 19, 12, 7), (-73.63527090000005, 45.44088628000002, 123, 3, 120), (-73.62032950000005, 45.44088628000002, 30, 10, 20), (-73.60538810000006, 45.44088628000002, 75, 38, 37), (-73.59044670000006, 45.44088628000002, 2, 0, 2), (-73.57550530000006, 45.44088628000002, 6, 4, 2), (-73.56056390000006, 45.44088628000002, 2, 2, 0), (-73.54562250000006, 45.44088628000002, 0, 0, 0), (-73.53068110000007, 45.44088628000002, 0, 0, 0), (-73.51573970000007, 45.44088628000002, 67, 6, 61), (-73.50079830000007, 45.44088628000002, 2, 2, 0), (-73.48585690000007, 45.44088628000002, 0, 0, 0), (-73.47091550000007, 45.44088628000002, 25, 21, 4), (-73.45597410000008, 45.44088628000002, 4, 0, 4), (-73.44103270000008, 45.44088628000002, 257, 75, 182), (-73.42609130000008, 45.44088628000002, 16, 5, 11), (-73.41114990000008, 45.44088628000002, 0, 0, 0), (-73.39620850000009, 45.44088628000002, 0, 0, 0), (-73.38126710000009, 45.44088628000002, 0, 0, 0), (-73.36632570000009, 45.44088628000002, 0, 0, 0), (-73.35138430000009, 45.44088628000002, 0, 0, 0), (-73.3364429000001, 45.44088628000002, 0, 0, 0), (-73.3215015000001, 45.44088628000002, 0, 0, 0), (-73.3065601000001, 45.44088628000002, 1, 1, 0), (-73.2916187000001, 45.44088628000002, 194, 9, 185), (-74.0237473, 45.451075000000024, 4, 0, 4), (-74.0088059, 45.451075000000024, 0, 0, 0), (-73.9938645, 45.451075000000024, 3, 0, 3), (-73.9789231, 45.451075000000024, 0, 0, 0), (-73.9639817, 45.451075000000024, 0, 0, 0), (-73.94904030000001, 45.451075000000024, 0, 0, 0), (-73.93409890000001, 45.451075000000024, 2, 1, 1), (-73.91915750000001, 45.451075000000024, 0, 0, 0), (-73.90421610000001, 45.451075000000024, 0, 0, 0), (-73.88927470000002, 45.451075000000024, 10, 0, 10), (-73.87433330000002, 45.451075000000024, 0, 0, 0), (-73.85939190000002, 45.451075000000024, 619, 11, 608), (-73.84445050000002, 45.451075000000024, 45, 0, 45), (-73.82950910000002,

45.451075000000024, 11, 2, 9), (-73.81456770000003, 45.451075000000024, 375, 30, 345), (-73.79962630000003, 45.451075000000024, 20, 9, 11), (-73.78468490000003, 45.451075000000024, 55, 25, 30), (-73.76974350000003, 45.451075000000024, 4, 0, 4), (-73.75480210000003, 45.451075000000024, 1350, 39, 1311), (-73.73986070000004, 45.451075000000024, 44, 6, 38), (-73.72491930000004, 45.451075000000024, 3, 0, 3), (-73.70997790000004, 45.451075000000024, 38, 1, 37), (-73.69503650000004, 45.451075000000024, 81, 5, 76), (-73.68009510000005, 45.451075000000024, 223, 0, 223), (-73.66515370000005, 45.451075000000024, 3, 0, 3), (-73.65021230000005, 45.451075000000024, 173, 81, 92), (-73.63527090000005, 45.451075000000024, 24, 12, 12), (-73.62032950000005, 45.451075000000024, 46, 11, 35), (-73.60538810000006, 45.451075000000024, 199, 49, 150), (-73.59044670000006, 45.451075000000024, 332, 167, 165), (-73.57550530000006, 45.451075000000024, 402, 183, 219), (-73.56056390000006, 45.451075000000024, 219, 132, 87), (-73.54562250000006, 45.451075000000024, 40, 9, 31), (-73.53068110000007, 45.451075000000024, 0, 0, 0), (-73.51573970000007, 45.451075000000024, 0, 0, 0), (-73.50079830000007, 45.451075000000024, 2, 1, 1), (-73.48585690000007, 45.451075000000024, 70, 23, 47), (-73.47091550000007, 45.451075000000024, 330, 76, 254), (-73.45597410000008, 45.451075000000024, 2297, 113, 2184), (-73.44103270000008, 45.451075000000024, 379, 94, 285), (-73.42609130000008, 45.451075000000024, 14, 12, 2), (-73.41114990000008, 45.451075000000024, 0, 0, 0), (-73.39620850000009, 45.451075000000024, 0, 0, 0), (-73.38126710000009, 45.451075000000024, 16, 0, 16), (-73.36632570000009, 45.451075000000024, 0, 0, 0), (-73.35138430000009, 45.451075000000024, 1, 1, 0), (-73.3364429000001, 45.451075000000024, 76, 10, 66), (-73.3215015000001, 45.451075000000024, 0, 0, 0), (-73.3065601000001, 45.451075000000024, 177, 34, 143), (-73.2916187000001, 45.451075000000024, 32, 10, 22), (-74.0237473, 45.46126372000003, 0, 0, 0), (-74.0088059, 45.46126372000003, 0, 0, 0), (-73.9938645, 45.46126372000003, 0, 0, 0), (-73.9789231, 45.46126372000003, 0, 0, 0), (-73.9639817, 45.46126372000003, 0, 0, 0), (-73.94904030000001, 45.46126372000003, 3, 2, 1), (-73.93409890000001, 45.46126372000003, 4, 3, 1), (-73.91915750000001, 45.46126372000003, 1, 1, 0), (-73.90421610000001, 45.46126372000003, 0, 0, 0), (-73.88927470000002, 45.46126372000003, 7, 1, 6), (-73.87433330000002, 45.46126372000003, 10, 1, 9), (-73.85939190000002, 45.46126372000003, 26, 1, 25), (-73.84445050000002, 45.46126372000003, 1, 0, 1), (-73.82950910000002, 45.46126372000003, 280, 25, 255), (-73.81456770000003, 45.46126372000003, 17, 5, 12), (-73.79962630000003, 45.46126372000003, 4, 0, 4), (-73.78468490000003, 45.46126372000003, 6, 3, 3), (-73.76974350000003, 45.46126372000003, 2, 0, 2), (-73.75480210000003, 45.46126372000003, 700, 475, 225), (-73.73986070000004, 45.46126372000003, 95, 54, 41), (-73.72491930000004, 45.46126372000003, 18, 2, 16), (-73.70997790000004, 45.46126372000003, 305, 0, 305), (-73.69503650000004, 45.46126372000003, 0, 0, 0), (-73.68009510000005, 45.46126372000003, 2, 0, 2), (-73.66515370000005, 45.46126372000003, 48, 1, 47), (-73.65021230000005, 45.46126372000003, 7, 0, 7), (-73.63527090000005, 45.46126372000003, 192, 25, 167), (-73.62032950000005, 45.46126372000003, 320, 10, 310), (-73.60538810000006, 45.46126372000003, 34, 11, 23), (-73.59044670000006, 45.46126372000003, 165, 66, 99), (-73.57550530000006, 45.46126372000003, 98, 29, 69), (-73.56056390000006, 45.46126372000003, 627, 218, 409), (-73.54562250000006, 45.46126372000003, 1735, 242, 1493), (-73.53068110000007, 45.46126372000003, 0, 0, 0), (-73.51573970000007, 45.46126372000003, 0, 0, 0), (-73.50079830000007, 45.46126372000003, 3, 2, 1), (-73.48585690000007, 45.46126372000003, 14, 10, 4), (-73.47091550000007, 45.46126372000003, 35, 8, 27), (-73.45597410000008, 45.46126372000003, 4, 0, 4), (-73.44103270000008, 45.46126372000003, 4, 0, 4), (-73.42609130000008, 45.46126372000003, 7, 0, 7), (-73.41114990000008, 45.46126372000003, 0, 0, 0), (-73.39620850000009, 45.46126372000003, 1, 1, 0), (-73.38126710000009, 45.46126372000003, 0, 0, 0),

(-73.36632570000009, 45.46126372000003, 0, 0, 0), (-73.35138430000009, 45.46126372000003, 10, 5, 5), (-73.3364429000001, 45.46126372000003, 46, 11, 35), (-73.3215015000001, 45.46126372000003, 2, 2, 0), (-73.3065601000001, 45.46126372000003, 8, 0, 8), (-73.2916187000001, 45.46126372000003, 4, 0, 4), (-74.0237473, 45.47145244000003, 0, 0, 0), (-74.0088059, 45.47145244000003, 0, 0, 0), (-73.9938645, 45.47145244000003, 0, 0, 0), (-73.9789231, 45.47145244000003, 0, 0, 0), (-73.9639817, 45.47145244000003, 0, 0, 0), (-73.94904030000001, 45.47145244000003, 0, 0, 0), (-73.93409890000001, 45.47145244000003, 19, 4, 15), (-73.91915750000001, 45.47145244000003, 1, 1, 0), (-73.90421610000001, 45.47145244000003, 1, 0, 1), (-73.88927470000002, 45.47145244000003, 318, 100, 218), (-73.87433330000002, 45.47145244000003, 19, 2, 17), (-73.85939190000002, 45.47145244000003, 14, 0, 14), (-73.84445050000002, 45.47145244000003, 5, 2, 3), (-73.82950910000002, 45.47145244000003, 431, 73, 358), (-73.81456770000003, 45.47145244000003, 10, 0, 10), (-73.79962630000003, 45.47145244000003, 1469, 105, 1364), (-73.78468490000003, 45.47145244000003, 269, 56, 213), (-73.76974350000003, 45.47145244000003, 6, 1, 5), (-73.75480210000003, 45.47145244000003, 0, 0, 0), (-73.73986070000004, 45.47145244000003, 10, 0, 10), (-73.72491930000004, 45.47145244000003, 2, 0, 2), (-73.70997790000004, 45.47145244000003, 9, 0, 9), (-73.69503650000004, 45.47145244000003, 1, 1, 0), (-73.68009510000005, 45.47145244000003, 54, 3, 51), (-73.66515370000005, 45.47145244000003, 626, 485, 141), (-73.65021230000005, 45.47145244000003, 518, 90, 428), (-73.63527090000005, 45.47145244000003, 45, 10, 35), (-73.62032950000005, 45.47145244000003, 793, 128, 665), (-73.60538810000006, 45.47145244000003, 332, 124, 208), (-73.59044670000006, 45.47145244000003, 697, 162, 535), (-73.57550530000006, 45.47145244000003, 362, 120, 242), (-73.56056390000006, 45.47145244000003, 101, 34, 67), (-73.54562250000006, 45.47145244000003, 39, 13, 26), (-73.53068110000007, 45.47145244000003, 5, 1, 4), (-73.51573970000007, 45.47145244000003, 25, 8, 17), (-73.50079830000007, 45.47145244000003, 3, 2, 1), (-73.48585690000007, 45.47145244000003, 11, 1, 10), (-73.47091550000007, 45.47145244000003, 158, 16, 142), (-73.45597410000008, 45.47145244000003, 4, 0, 4), (-73.44103270000008, 45.47145244000003, 10, 1, 9), (-73.42609130000008, 45.47145244000003, 2, 0, 2), (-73.41114990000008, 45.47145244000003, 0, 0, 0), (-73.39620850000009, 45.47145244000003, 2, 1, 1), (-73.38126710000009, 45.47145244000003, 0, 0, 0), (-73.36632570000009, 45.47145244000003, 0, 0, 0), (-73.35138430000009, 45.47145244000003, 0, 0, 0), (-73.3364429000001, 45.47145244000003, 0, 0, 0), (-73.3215015000001, 45.47145244000003, 0, 0, 0), (-73.3065601000001, 45.47145244000003, 0, 0, 0), (-73.2916187000001, 45.47145244000003, 0, 0, 0), (-74.0237473, 45.48164116000003, 9, 1, 8), (-74.0088059, 45.48164116000003, 0, 0, 0), (-73.9938645, 45.48164116000003, 0, 0, 0), (-73.9789231, 45.48164116000003, 0, 0, 0), (-73.9639817, 45.48164116000003, 0, 0, 0), (-73.94904030000001, 45.48164116000003, 0, 0, 0), (-73.93409890000001, 45.48164116000003, 0, 0, 0), (-73.91915750000001, 45.48164116000003, 6, 1, 5), (-73.90421610000001, 45.48164116000003, 84, 62, 22), (-73.88927470000002, 45.48164116000003, 0, 0, 0), (-73.87433330000002, 45.48164116000003, 59, 15, 44), (-73.85939190000002, 45.48164116000003, 15, 3, 12), (-73.84445050000002, 45.48164116000003, 12, 3, 9), (-73.82950910000002, 45.48164116000003, 5, 5, 0), (-73.81456770000003, 45.48164116000003, 101, 14, 87), (-73.79962630000003, 45.48164116000003, 1178, 320, 858), (-73.78468490000003, 45.48164116000003, 366, 11, 355), (-73.76974350000003, 45.48164116000003, 1, 0, 1), (-73.75480210000003, 45.48164116000003, 7, 4, 3), (-73.73986070000004, 45.48164116000003, 1, 0, 1), (-73.72491930000004, 45.48164116000003, 6, 1, 5), (-73.70997790000004, 45.48164116000003, 194, 0, 194), (-73.69503650000004, 45.48164116000003, 68, 8, 60), (-73.68009510000005, 45.48164116000003, 0, 0, 0), (-73.66515370000005, 45.48164116000003, 6, 0, 6), (-73.65021230000005, 45.48164116000003, 29, 2, 27), (-73.63527090000005, 45.48164116000003, 387, 48, 339), (-73.62032950000005, 45.48164116000003, 749, 233, 516),

(-73.60538810000006, 45.48164116000003, 1097, 47, 1050), (-73.59044670000006, 45.48164116000003, 1352, 139, 1213), (-73.57550530000006, 45.48164116000003, 1312, 362, 950), (-73.56056390000006, 45.48164116000003, 1090, 206, 884), (-73.54562250000006, 45.48164116000003, 70, 26, 44), (-73.53068110000007, 45.48164116000003, 0, 0, 0), (-73.51573970000007, 45.48164116000003, 0, 0, 0), (-73.50079830000007, 45.48164116000003, 3, 1, 2), (-73.48585690000007, 45.48164116000003, 64, 8, 56), (-73.47091550000007, 45.48164116000003, 21, 12, 9), (-73.45597410000008, 45.48164116000003, 22, 8, 14), (-73.44103270000008, 45.48164116000003, 2, 1, 1), (-73.42609130000008, 45.48164116000003, 1, 0, 1), (-73.41114990000008, 45.48164116000003, 9, 1, 8), (-73.39620850000009, 45.48164116000003, 17, 2, 15), (-73.38126710000009, 45.48164116000003, 215, 27, 188), (-73.36632570000009, 45.48164116000003, 0, 0, 0), (-73.35138430000009, 45.48164116000003, 0, 0, 0), (-73.3364429000001, 45.48164116000003, 0, 0, 0), (-73.3215015000001, 45.48164116000003, 0, 0, 0), (-73.3065601000001, 45.48164116000003, 0, 0, 0), (-73.2916187000001, 45.48164116000003, 0, 0, 0), (-74.0237473, 45.49182988000003, 12, 5, 7), (-74.0088059, 45.49182988000003, 0, 0, 0), (-73.9938645, 45.49182988000003, 0, 0, 0), (-73.9789231, 45.49182988000003, 1, 1, 0), (-73.9639817, 45.49182988000003, 0, 0, 0), (-73.94904030000001, 45.49182988000003, 0, 0, 0), (-73.93409890000001, 45.49182988000003, 0, 0, 0), (-73.91915750000001, 45.49182988000003, 0, 0, 0), (-73.90421610000001, 45.49182988000003, 8, 0, 8), (-73.88927470000002, 45.49182988000003, 0, 0, 0), (-73.87433330000002, 45.49182988000003, 31, 20, 11), (-73.85939190000002, 45.49182988000003, 4, 0, 4), (-73.84445050000002, 45.49182988000003, 75, 12, 63), (-73.82950910000002, 45.49182988000003, 8, 0, 8), (-73.81456770000003, 45.49182988000003, 1355, 238, 1117), (-73.79962630000003, 45.49182988000003, 32, 5, 27), (-73.78468490000003, 45.49182988000003, 1, 0, 1), (-73.76974350000003, 45.49182988000003, 4, 0, 4), (-73.75480210000003, 45.49182988000003, 48, 20, 28), (-73.73986070000004, 45.49182988000003, 57, 29, 28), (-73.72491930000004, 45.49182988000003, 19, 0, 19), (-73.70997790000004, 45.49182988000003, 73, 13, 60), (-73.69503650000004, 45.49182988000003, 41, 18, 23), (-73.68009510000005, 45.49182988000003, 75, 8, 67), (-73.66515370000005, 45.49182988000003, 758, 19, 739), (-73.65021230000005, 45.49182988000003, 590, 233, 357), (-73.63527090000005, 45.49182988000003, 245, 49, 196), (-73.62032950000005, 45.49182988000003, 833, 279, 554), (-73.60538810000006, 45.49182988000003, 200, 37, 163), (-73.59044670000006, 45.49182988000003, 635, 99, 536), (-73.57550530000006, 45.49182988000003, 3906, 908, 2998), (-73.56056390000006, 45.49182988000003, 843, 272, 571), (-73.54562250000006, 45.49182988000003, 33, 17, 16), (-73.53068110000007, 45.49182988000003, 14, 2, 12), (-73.51573970000007, 45.49182988000003, 3, 2, 1), (-73.50079830000007, 45.49182988000003, 99, 3, 96), (-73.48585690000007, 45.49182988000003, 18, 1, 17), (-73.47091550000007, 45.49182988000003, 45, 5, 40), (-73.45597410000008, 45.49182988000003, 16, 3, 13), (-73.44103270000008, 45.49182988000003, 3, 2, 1), (-73.42609130000008, 45.49182988000003, 3, 2, 1), (-73.41114990000008, 45.49182988000003, 36, 10, 26), (-73.39620850000009, 45.49182988000003, 11, 3, 8), (-73.38126710000009, 45.49182988000003, 72, 14, 58), (-73.36632570000009, 45.49182988000003, 0, 0, 0), (-73.35138430000009, 45.49182988000003, 2, 0, 2), (-73.3364429000001, 45.49182988000003, 0, 0, 0), (-73.3215015000001, 45.49182988000003, 0, 0, 0), (-73.3065601000001, 45.49182988000003, 0, 0, 0), (-73.2916187000001, 45.49182988000003, 0, 0, 0), (-74.0237473, 45.502018600000035, 0, 0, 0), (-74.0088059, 45.502018600000035, 0, 0, 0), (-73.9938645, 45.502018600000035, 0, 0, 0), (-73.9789231, 45.502018600000035, 2, 1, 1), (-73.9639817, 45.502018600000035, 44, 6, 38), (-73.94904030000001, 45.502018600000035, 0, 0, 0), (-73.93409890000001, 45.502018600000035, 0, 0, 0), (-73.91915750000001, 45.502018600000035, 0, 0, 0), (-73.90421610000001, 45.502018600000035, 17, 8, 9), (-73.88927470000002, 45.502018600000035, 0, 0, 0), (-73.87433330000002, 45.502018600000035, 1, 0, 1),

(-73.85939190000002, 45.502018600000035, 4, 3, 1), (-73.84445050000002, 45.502018600000035, 16, 9, 7), (-73.82950910000002, 45.502018600000035, 47, 0, 47), (-73.81456770000003, 45.502018600000035, 9, 0, 9), (-73.79962630000003, 45.502018600000035, 12, 4, 8), (-73.78468490000003, 45.502018600000035, 20, 6, 14), (-73.76974350000003, 45.502018600000035, 16, 2, 14), (-73.75480210000003, 45.502018600000035, 679, 35, 644), (-73.73986070000004, 45.502018600000035, 1, 0, 1), (-73.72491930000004, 45.502018600000035, 34, 3, 31), (-73.70997790000004, 45.502018600000035, 131, 36, 95), (-73.69503650000004, 45.502018600000035, 22, 2, 20), (-73.68009510000005, 45.502018600000035, 22, 5, 17), (-73.66515370000005, 45.502018600000035, 99, 14, 85), (-73.65021230000005, 45.502018600000035, 2220, 485, 1735), (-73.63527090000005, 45.502018600000035, 165, 50, 115), (-73.62032950000005, 45.502018600000035, 469, 281, 188), (-73.60538810000006, 45.502018600000035, 373, 204, 169), (-73.59044670000006, 45.502018600000035, 5385, 1457, 3928), (-73.57550530000006, 45.502018600000035, 41159, 11364, 29795), (-73.56056390000006, 45.502018600000035, 36697, 5636, 31061), (-73.54562250000006, 45.502018600000035, 960, 423, 537), (-73.53068110000007, 45.502018600000035, 418, 222, 196), (-73.51573970000007, 45.502018600000035, 402, 203, 199), (-73.50079830000007, 45.502018600000035, 21, 11, 10), (-73.48585690000007, 45.502018600000035, 11, 8, 3), (-73.47091550000007, 45.502018600000035, 5, 3, 2), (-73.45597410000008, 45.502018600000035, 0, 0, 0), (-73.44103270000008, 45.502018600000035, 91, 3, 88), (-73.42609130000008, 45.502018600000035, 42, 4, 38), (-73.41114990000008, 45.502018600000035, 857, 185, 672), (-73.39620850000009, 45.502018600000035, 0, 0, 0), (-73.38126710000009, 45.502018600000035, 106, 12, 94), (-73.36632570000009, 45.502018600000035, 0, 0, 0), (-73.35138430000009, 45.502018600000035, 0, 0, 0), (-73.3364429000001, 45.502018600000035, 0, 0, 0), (-73.3215015000001, 45.502018600000035, 0, 0, 0), (-73.3065601000001, 45.502018600000035, 0, 0, 0), (-73.2916187000001, 45.502018600000035, 1, 1, 0), (-74.0237473, 45.51220732000004, 0, 0, 0), (-74.0088059, 45.51220732000004, 0, 0, 0), (-73.9938645, 45.51220732000004, 3, 2, 1), (-73.9789231, 45.51220732000004, 1, 0, 1), (-73.9639817, 45.51220732000004, 0, 0, 0), (-73.94904030000001, 45.51220732000004, 0, 0, 0), (-73.93409890000001, 45.51220732000004, 0, 0, 0), (-73.91915750000001, 45.51220732000004, 0, 0, 0), (-73.90421610000001, 45.51220732000004, 0, 0, 0), (-73.88927470000002, 45.51220732000004, 7, 5, 2), (-73.87433330000002, 45.51220732000004, 0, 0, 0), (-73.85939190000002, 45.51220732000004, 0, 0, 0), (-73.84445050000002, 45.51220732000004, 1, 1, 0), (-73.82950910000002, 45.51220732000004, 5, 4, 1), (-73.81456770000003, 45.51220732000004, 33, 4, 29), (-73.79962630000003, 45.51220732000004, 1, 0, 1), (-73.78468490000003, 45.51220732000004, 3, 0, 3), (-73.76974350000003, 45.51220732000004, 0, 0, 0), (-73.75480210000003, 45.51220732000004, 1, 0, 1), (-73.73986070000004, 45.51220732000004, 11, 1, 10), (-73.72491930000004, 45.51220732000004, 20, 6, 14), (-73.70997790000004, 45.51220732000004, 107, 32, 75), (-73.69503650000004, 45.51220732000004, 210, 9, 201), (-73.68009510000005, 45.51220732000004, 192, 31, 161), (-73.66515370000005, 45.51220732000004, 349, 117, 232), (-73.65021230000005, 45.51220732000004, 513, 105, 408), (-73.63527090000005, 45.51220732000004, 67, 12, 55), (-73.62032950000005, 45.51220732000004, 1630, 100, 1530), (-73.60538810000006, 45.51220732000004, 139, 74, 65), (-73.59044670000006, 45.51220732000004, 3234, 293, 2941), (-73.57550530000006, 45.51220732000004, 2304, 656, 1648), (-73.56056390000006, 45.51220732000004, 8273, 3185, 5088), (-73.54562250000006, 45.51220732000004, 801, 282, 519), (-73.53068110000007, 45.51220732000004, 411, 156, 255), (-73.51573970000007, 45.51220732000004, 50, 5, 45), (-73.50079830000007, 45.51220732000004, 11, 2, 9), (-73.48585690000007, 45.51220732000004, 6, 3, 3), (-73.47091550000007, 45.51220732000004, 9, 8, 1), (-73.45597410000008, 45.51220732000004, 88, 81, 7), (-73.44103270000008, 45.51220732000004, 8, 3, 5), (-73.42609130000008, 45.51220732000004, 19, 11, 8), (-73.41114990000008,

45.51220732000004, 2, 1, 1), (-73.39620850000009, 45.51220732000004, 2, 1, 1), (-73.38126710000009, 45.51220732000004, 10, 4, 6), (-73.36632570000009, 45.51220732000004, 0, 0, 0), (-73.35138430000009, 45.51220732000004, 0, 0, 0), (-73.3364429000001, 45.51220732000004, 0, 0, 0), (-73.3215015000001, 45.51220732000004, 0, 0, 0), (-73.3065601000001, 45.51220732000004, 0, 0, 0), (-73.2916187000001, 45.51220732000004, 0, 0, 0), (-74.0237473, 45.52239604000004, 0, 0, 0), (-74.0088059, 45.52239604000004, 0, 0, 0), (-73.9938645, 45.52239604000004, 2, 1, 1), (-73.9789231, 45.52239604000004, 0, 0, 0), (-73.9639817, 45.52239604000004, 0, 0, 0), (-73.94904030000001, 45.52239604000004, 1, 0, 1), (-73.93409890000001, 45.52239604000004, 1, 1, 0), (-73.91915750000001, 45.52239604000004, 0, 0, 0), (-73.90421610000001, 45.52239604000004, 0, 0, 0), (-73.88927470000002, 45.52239604000004, 0, 0, 0), (-73.87433330000002, 45.52239604000004, 4, 2, 2), (-73.85939190000002, 45.52239604000004, 19, 15, 4), (-73.84445050000002, 45.52239604000004, 8, 1, 7), (-73.82950910000002, 45.52239604000004, 0, 0, 0), (-73.81456770000003, 45.52239604000004, 1, 1, 0), (-73.79962630000003, 45.52239604000004, 8, 2, 6), (-73.78468490000003, 45.52239604000004, 21, 9, 12), (-73.76974350000003, 45.52239604000004, 3, 0, 3), (-73.75480210000003, 45.52239604000004, 4, 3, 1), (-73.73986070000004, 45.52239604000004, 25, 6, 19), (-73.72491930000004, 45.52239604000004, 22, 10, 12), (-73.70997790000004, 45.52239604000004, 71, 26, 45), (-73.69503650000004, 45.52239604000004, 50, 9, 41), (-73.68009510000005, 45.52239604000004, 157, 22, 135), (-73.66515370000005, 45.52239604000004, 200, 176, 24), (-73.65021230000005, 45.52239604000004, 11, 3, 8), (-73.63527090000005, 45.52239604000004, 8, 6, 2), (-73.62032950000005, 45.52239604000004, 644, 238, 406), (-73.60538810000006, 45.52239604000004, 1500, 424, 1076), (-73.59044670000006, 45.52239604000004, 2844, 1125, 1719), (-73.57550530000006, 45.52239604000004, 3710, 1736, 1974), (-73.56056390000006, 45.52239604000004, 2756, 1441, 1315), (-73.54562250000006, 45.52239604000004, 804, 431, 373), (-73.53068110000007, 45.52239604000004, 165, 68, 97), (-73.51573970000007, 45.52239604000004, 47, 31, 16), (-73.50079830000007, 45.52239604000004, 31, 12, 19), (-73.48585690000007, 45.52239604000004, 4, 2, 2), (-73.47091550000007, 45.52239604000004, 354, 158, 196), (-73.45597410000008, 45.52239604000004, 15, 5, 10), (-73.44103270000008, 45.52239604000004, 0, 0, 0), (-73.42609130000008, 45.52239604000004, 1, 0, 1), (-73.41114990000008, 45.52239604000004, 0, 0, 0), (-73.39620850000009, 45.52239604000004, 10, 6, 4), (-73.38126710000009, 45.52239604000004, 0, 0, 0), (-73.36632570000009, 45.52239604000004, 13, 1, 12), (-73.35138430000009, 45.52239604000004, 8, 1, 7), (-73.3364429000001, 45.52239604000004, 201, 21, 180), (-73.3215015000001, 45.52239604000004, 0, 0, 0), (-73.3065601000001, 45.52239604000004, 1, 1, 0), (-73.2916187000001, 45.52239604000004, 10, 6, 4), (-74.0237473, 45.53258476000004, 0, 0, 0), (-74.0088059, 45.53258476000004, 2, 2, 0), (-73.9938645, 45.53258476000004, 35, 25, 10), (-73.9789231, 45.53258476000004, 0, 0, 0), (-73.9639817, 45.53258476000004, 0, 0, 0), (-73.94904030000001, 45.53258476000004, 0, 0, 0), (-73.93409890000001, 45.53258476000004, 16, 9, 7), (-73.91915750000001, 45.53258476000004, 0, 0, 0), (-73.90421610000001, 45.53258476000004, 0, 0, 0), (-73.88927470000002, 45.53258476000004, 13, 3, 10), (-73.87433330000002, 45.53258476000004, 4, 2, 2), (-73.85939190000002, 45.53258476000004, 1, 1, 0), (-73.84445050000002, 45.53258476000004, 1, 1, 0), (-73.82950910000002, 45.53258476000004, 1, 0, 1), (-73.81456770000003, 45.53258476000004, 27, 18, 9), (-73.79962630000003, 45.53258476000004, 10, 4, 6), (-73.78468490000003, 45.53258476000004, 36, 12, 24), (-73.76974350000003, 45.53258476000004, 56, 1, 55), (-73.75480210000003, 45.53258476000004, 10, 4, 6), (-73.73986070000004, 45.53258476000004, 48, 9, 39), (-73.72491930000004, 45.53258476000004, 30, 21, 9), (-73.70997790000004, 45.53258476000004, 87, 40, 47), (-73.69503650000004, 45.53258476000004, 4, 2, 2), (-73.68009510000005, 45.53258476000004, 19, 6, 13), (-73.66515370000005, 45.53258476000004, 80, 51, 29), (-73.65021230000005, 45.53258476000004, 484, 89, 395),

(-73.63527090000005, 45.53258476000004, 125, 71, 54), (-73.62032950000005, 45.53258476000004, 2042, 1059, 983), (-73.60538810000006, 45.53258476000004, 2553, 764, 1789), (-73.59044670000006, 45.53258476000004, 355, 229, 126), (-73.57550530000006, 45.53258476000004, 425, 285, 140), (-73.56056390000006, 45.53258476000004, 229, 134, 95), (-73.54562250000006, 45.53258476000004, 89, 36, 53), (-73.53068110000007, 45.53258476000004, 0, 0, 0), (-73.51573970000007, 45.53258476000004, 1515, 353, 1162), (-73.50079830000007, 45.53258476000004, 11, 9, 2), (-73.48585690000007, 45.53258476000004, 51, 24, 27), (-73.47091550000007, 45.53258476000004, 4, 1, 3), (-73.45597410000008, 45.53258476000004, 2, 1, 1), (-73.44103270000008, 45.53258476000004, 85, 25, 60), (-73.42609130000008, 45.53258476000004, 3, 0, 3), (-73.41114990000008, 45.53258476000004, 2, 2, 0), (-73.39620850000009, 45.53258476000004, 0, 0, 0), (-73.38126710000009, 45.53258476000004, 0, 0, 0), (-73.36632570000009, 45.53258476000004, 0, 0, 0), (-73.35138430000009, 45.53258476000004, 255, 32, 223), (-73.3364429000001, 45.53258476000004, 6, 1, 5), (-73.3215015000001, 45.53258476000004, 0, 0, 0), (-73.3065601000001, 45.53258476000004, 1, 1, 0), (-73.2916187000001, 45.53258476000004, 0, 0, 0), (-74.0237473, 45.542773480000044, 2, 2, 0), (-74.0088059, 45.542773480000044, 8, 5, 3), (-73.9938645, 45.542773480000044, 0, 0, 0), (-73.9789231, 45.542773480000044, 0, 0, 0), (-73.9639817, 45.542773480000044, 0, 0, 0), (-73.94904030000001, 45.542773480000044, 0, 0, 0), (-73.93409890000001, 45.542773480000044, 0, 0, 0), (-73.91915750000001, 45.542773480000044, 6, 2, 4), (-73.90421610000001, 45.542773480000044, 4, 2, 2), (-73.88927470000002, 45.542773480000044, 11, 5, 6), (-73.87433330000002, 45.542773480000044, 31, 18, 13), (-73.85939190000002, 45.542773480000044, 9, 4, 5), (-73.84445050000002, 45.542773480000044, 0, 0, 0), (-73.82950910000002, 45.542773480000044, 0, 0, 0), (-73.81456770000003, 45.542773480000044, 1, 0, 1), (-73.79962630000003, 45.542773480000044, 0, 0, 0), (-73.78468490000003, 45.542773480000044, 5, 0, 5), (-73.76974350000003, 45.542773480000044, 28, 8, 20), (-73.75480210000003, 45.542773480000044, 61, 20, 41), (-73.73986070000004, 45.542773480000044, 1, 1, 0), (-73.72491930000004, 45.542773480000044, 11, 5, 6), (-73.70997790000004, 45.542773480000044, 2, 1, 1), (-73.69503650000004, 45.542773480000044, 11, 6, 5), (-73.68009510000005, 45.542773480000044, 13, 8, 5), (-73.66515370000005, 45.542773480000044, 212, 95, 117), (-73.65021230000005, 45.542773480000044, 82, 24, 58), (-73.63527090000005, 45.542773480000044, 4198, 426, 3772), (-73.62032950000005, 45.542773480000044, 535, 160, 375), (-73.60538810000006, 45.542773480000044, 1078, 359, 719), (-73.59044670000006, 45.542773480000044, 397, 277, 120), (-73.57550530000006, 45.542773480000044, 269, 186, 83), (-73.56056390000006, 45.542773480000044, 116, 69, 47), (-73.54562250000006, 45.542773480000044, 533, 311, 222), (-73.53068110000007, 45.542773480000044, 57, 50, 7), (-73.51573970000007, 45.542773480000044, 16, 10, 6), (-73.50079830000007, 45.542773480000044, 9, 6, 3), (-73.48585690000007, 45.542773480000044, 19, 11, 8), (-73.47091550000007, 45.542773480000044, 107, 17, 90), (-73.45597410000008, 45.542773480000044, 7, 4, 3), (-73.44103270000008, 45.542773480000044, 3, 0, 3), (-73.42609130000008, 45.542773480000044, 4, 3, 1), (-73.41114990000008, 45.542773480000044, 1, 1, 0), (-73.39620850000009, 45.542773480000044, 0, 0, 0), (-73.38126710000009, 45.542773480000044, 0, 0, 0), (-73.36632570000009, 45.542773480000044, 1, 0, 1), (-73.35138430000009, 45.542773480000044, 0, 0, 0), (-73.3364429000001, 45.542773480000044, 61, 14, 47), (-73.3215015000001, 45.542773480000044, 0, 0, 0), (-73.3065601000001, 45.542773480000044, 0, 0, 0), (-73.2916187000001, 45.542773480000044, 0, 0, 0), (-74.0237473, 45.552962200000046, 10, 3, 7), (-74.0088059, 45.552962200000046, 0, 0, 0), (-73.9938645, 45.552962200000046, 0, 0, 0), (-73.9789231, 45.552962200000046, 0, 0, 0), (-73.9639817, 45.552962200000046, 0, 0, 0), (-73.94904030000001, 45.552962200000046, 0, 0, 0), (-73.93409890000001, 45.552962200000046, 0, 0, 0), (-73.91915750000001,

45.552962200000046, 0, 0, 0), (-73.90421610000001, 45.552962200000046, 2, 0, 2), (-73.88927470000002, 45.552962200000046, 4, 3, 1), (-73.87433330000002, 45.552962200000046, 7, 1, 6), (-73.85939190000002, 45.552962200000046, 0, 0, 0), (-73.84445050000002, 45.552962200000046, 134, 0, 134), (-73.82950910000002, 45.552962200000046, 0, 0, 0), (-73.81456770000003, 45.552962200000046, 1, 0, 1), (-73.79962630000003, 45.552962200000046, 0, 0, 0), (-73.78468490000003, 45.552962200000046, 57, 2, 55), (-73.76974350000003, 45.552962200000046, 23, 3, 20), (-73.75480210000003, 45.552962200000046, 53, 33, 20), (-73.73986070000004, 45.552962200000046, 72, 29, 43), (-73.72491930000004, 45.552962200000046, 304, 97, 207), (-73.70997790000004, 45.552962200000046, 61, 60, 1), (-73.69503650000004, 45.552962200000046, 1609, 311, 1298), (-73.68009510000005, 45.552962200000046, 3, 2, 1), (-73.66515370000005, 45.552962200000046, 95, 57, 38), (-73.65021230000005, 45.552962200000046, 43, 26, 17), (-73.63527090000005, 45.552962200000046, 190, 64, 126), (-73.62032950000005, 45.552962200000046, 86, 60, 26), (-73.60538810000006, 45.552962200000046, 747, 295, 452), (-73.59044670000006, 45.552962200000046, 169, 152, 17), (-73.57550530000006, 45.552962200000046, 218, 136, 82), (-73.56056390000006, 45.552962200000046, 340, 186, 154), (-73.54562250000006, 45.552962200000046, 662, 405, 257), (-73.53068110000007, 45.552962200000046, 129, 96, 33), (-73.51573970000007, 45.552962200000046, 0, 0, 0), (-73.50079830000007, 45.552962200000046, 4, 3, 1), (-73.48585690000007, 45.552962200000046, 21, 12, 9), (-73.47091550000007, 45.552962200000046, 1, 1, 0), (-73.45597410000008, 45.552962200000046, 2, 0, 2), (-73.44103270000008, 45.552962200000046, 0, 0, 0), (-73.42609130000008, 45.552962200000046, 2, 0, 2), (-73.41114990000008, 45.552962200000046, 0, 0, 0), (-73.39620850000009, 45.552962200000046, 0, 0, 0), (-73.38126710000009, 45.552962200000046, 0, 0, 0), (-73.36632570000009, 45.552962200000046, 0, 0, 0), (-73.35138430000009, 45.552962200000046, 1, 0, 1), (-73.3364429000001, 45.552962200000046, 0, 0, 0), (-73.3215015000001, 45.552962200000046, 30, 11, 19), (-73.3065601000001, 45.552962200000046, 0, 0, 0), (-73.2916187000001, 45.552962200000046, 1, 0, 1), (-74.0237473, 45.56315092000005, 0, 0, 0), (-74.0088059, 45.56315092000005, 41, 11, 30), (-73.9938645, 45.56315092000005, 0, 0, 0), (-73.9789231, 45.56315092000005, 6, 3, 3), (-73.9639817, 45.56315092000005, 0, 0, 0), (-73.94904030000001, 45.56315092000005, 0, 0, 0), (-73.93409890000001, 45.56315092000005, 0, 0, 0), (-73.91915750000001, 45.56315092000005, 11, 2, 9), (-73.90421610000001, 45.56315092000005, 94, 47, 47), (-73.88927470000002, 45.56315092000005, 200, 43, 157), (-73.87433330000002, 45.56315092000005, 0, 0, 0), (-73.85939190000002, 45.56315092000005, 3, 3, 0), (-73.84445050000002, 45.56315092000005, 8, 4, 4), (-73.82950910000002, 45.56315092000005, 0, 0, 0), (-73.81456770000003, 45.56315092000005, 4, 0, 4), (-73.79962630000003, 45.56315092000005, 12, 8, 4), (-73.78468490000003, 45.56315092000005, 66, 30, 36), (-73.76974350000003, 45.56315092000005, 24, 6, 18), (-73.75480210000003, 45.56315092000005, 253, 29, 224), (-73.73986070000004, 45.56315092000005, 269, 146, 123), (-73.72491930000004, 45.56315092000005, 182, 75, 107), (-73.70997790000004, 45.56315092000005, 19, 11, 8), (-73.69503650000004, 45.56315092000005, 70, 57, 13), (-73.68009510000005, 45.56315092000005, 25, 12, 13), (-73.66515370000005, 45.56315092000005, 3079, 576, 2503), (-73.65021230000005, 45.56315092000005, 111, 78, 33), (-73.63527090000005, 45.56315092000005, 86, 38, 48), (-73.62032950000005, 45.56315092000005, 123, 70, 53), (-73.60538810000006, 45.56315092000005, 100, 46, 54), (-73.59044670000006, 45.56315092000005, 60, 37, 23), (-73.57550530000006, 45.56315092000005, 191, 88, 103), (-73.56056390000006, 45.56315092000005, 99, 64, 35), (-73.54562250000006, 45.56315092000005, 386, 249, 137), (-73.53068110000007, 45.56315092000005, 19, 9, 10), (-73.51573970000007, 45.56315092000005, 3, 0, 3), (-73.50079830000007, 45.56315092000005, 20, 0, 20), (-73.48585690000007, 45.56315092000005, 36, 24, 12), (-73.47091550000007,

45.56315092000005, 22, 12, 10), (-73.4559741000008, 45.56315092000005, 4, 1, 3), (-73.44103270000008, 45.56315092000005, 2, 1, 1), (-73.42609130000008, 45.56315092000005, 89, 3, 86), (-73.41114990000008, 45.56315092000005, 8, 1, 7), (-73.39620850000009, 45.56315092000005, 0, 0, 0), (-73.38126710000009, 45.56315092000005, 3, 3, 0), (-73.36632570000009, 45.56315092000005, 0, 0, 0), (-73.35138430000009, 45.56315092000005, 0, 0, 0), (-73.3364429000001, 45.56315092000005, 22, 8, 14), (-73.3215015000001, 45.56315092000005, 0, 0, 0), (-73.3065601000001, 45.56315092000005, 0, 0, 0), (-73.2916187000001, 45.56315092000005, 25, 5, 20), (-74.0237473, 45.57333964000005, 0, 0, 0), (-74.0088059, 45.57333964000005, 0, 0, 0), (-73.9938645, 45.57333964000005, 0, 0, 0), (-73.9789231, 45.57333964000005, 0, 0, 0), (-73.9639817, 45.57333964000005, 28, 1, 27), (-73.94904030000001, 45.57333964000005, 0, 0, 0), (-73.93409890000001, 45.57333964000005, 0, 0, 0), (-73.91915750000001, 45.57333964000005, 2, 1, 1), (-73.90421610000001, 45.57333964000005, 12, 6, 6), (-73.88927470000002, 45.57333964000005, 13, 10, 3), (-73.87433330000002, 45.57333964000005, 4, 4, 0), (-73.85939190000002, 45.57333964000005, 2, 1, 1), (-73.84445050000002, 45.57333964000005, 0, 0, 0), (-73.82950910000002, 45.57333964000005, 1, 0, 1), (-73.81456770000003, 45.57333964000005, 48, 31, 17), (-73.79962630000003, 45.57333964000005, 1, 1, 0), (-73.78468490000003, 45.57333964000005, 1205, 40, 1165), (-73.76974350000003, 45.57333964000005, 11, 3, 8), (-73.75480210000003, 45.57333964000005, 424, 133, 291), (-73.73986070000004, 45.57333964000005, 16, 12, 4), (-73.72491930000004, 45.57333964000005, 54, 14, 40), (-73.70997790000004, 45.57333964000005, 1772, 38, 1734), (-73.69503650000004, 45.57333964000005, 32, 19, 13), (-73.68009510000005, 45.57333964000005, 53, 35, 18), (-73.66515370000005, 45.57333964000005, 51, 26, 25), (-73.65021230000005, 45.57333964000005, 23, 18, 5), (-73.63527090000005, 45.57333964000005, 44, 9, 35), (-73.62032950000005, 45.57333964000005, 11, 6, 5), (-73.60538810000006, 45.57333964000005, 1036, 500, 536), (-73.59044670000006, 45.57333964000005, 156, 26, 130), (-73.57550530000006, 45.57333964000005, 23, 16, 7), (-73.56056390000006, 45.57333964000005, 21, 8, 13), (-73.54562250000006, 45.57333964000005, 41, 22, 19), (-73.53068110000007, 45.57333964000005, 429, 295, 134), (-73.51573970000007, 45.57333964000005, 0, 0, 0), (-73.50079830000007, 45.57333964000005, 0, 0, 0), (-73.48585690000007, 45.57333964000005, 9, 4, 5), (-73.47091550000007, 45.57333964000005, 17, 0, 17), (-73.4559741000008, 45.57333964000005, 105, 98, 7), (-73.44103270000008, 45.57333964000005, 56, 20, 36), (-73.42609130000008, 45.57333964000005, 1, 0, 1), (-73.41114990000008, 45.57333964000005, 36, 1, 35), (-73.39620850000009, 45.57333964000005, 15, 3, 12), (-73.38126710000009, 45.57333964000005, 1, 0, 1), (-73.36632570000009, 45.57333964000005, 0, 0, 0), (-73.35138430000009, 45.57333964000005, 0, 0, 0), (-73.3364429000001, 45.57333964000005, 7, 6, 1), (-73.3215015000001, 45.57333964000005, 21, 7, 14), (-73.3065601000001, 45.57333964000005, 0, 0, 0), (-73.2916187000001, 45.57333964000005, 0, 0, 0), (-74.0237473, 45.58352836000005, 0, 0, 0), (-74.0088059, 45.58352836000005, 0, 0, 0), (-73.9938645, 45.58352836000005, 0, 0, 0), (-73.9789231, 45.58352836000005, 12, 7, 5), (-73.9639817, 45.58352836000005, 1, 0, 1), (-73.94904030000001, 45.58352836000005, 0, 0, 0), (-73.93409890000001, 45.58352836000005, 2, 1, 1), (-73.91915750000001, 45.58352836000005, 0, 0, 0), (-73.90421610000001, 45.58352836000005, 0, 0, 0), (-73.88927470000002, 45.58352836000005, 0, 0, 0), (-73.87433330000002, 45.58352836000005, 20, 12, 8), (-73.85939190000002, 45.58352836000005, 1, 1, 0), (-73.84445050000002, 45.58352836000005, 0, 0, 0), (-73.82950910000002, 45.58352836000005, 0, 0, 0), (-73.81456770000003, 45.58352836000005, 2, 2, 0), (-73.79962630000003, 45.58352836000005, 9, 6, 3), (-73.78468490000003, 45.58352836000005, 15, 13, 2), (-73.76974350000003, 45.58352836000005, 8, 5, 3), (-73.75480210000003, 45.58352836000005, 9, 1, 8), (-73.73986070000004, 45.58352836000005, 9, 3, 6), (-73.72491930000004, 45.58352836000005, 3, 2, 1), (-73.70997790000004, 45.58352836000005, 29, 15, 14), (-73.69503650000004,

45.58352836000005, 3, 2, 1), (-73.68009510000005, 45.58352836000005, 1, 1, 0), (-73.66515370000005, 45.58352836000005, 3, 3, 0), (-73.65021230000005, 45.58352836000005, 39, 15, 24), (-73.63527090000005, 45.58352836000005, 152, 81, 71), (-73.62032950000005, 45.58352836000005, 7, 4, 3), (-73.60538810000006, 45.58352836000005, 216, 74, 142), (-73.59044670000006, 45.58352836000005, 91, 31, 60), (-73.57550530000006, 45.58352836000005, 17, 10, 7), (-73.56056390000006, 45.58352836000005, 19, 10, 9), (-73.54562250000006, 45.58352836000005, 83, 14, 69), (-73.53068110000007, 45.58352836000005, 22, 13, 9), (-73.51573970000007, 45.58352836000005, 1, 0, 1), (-73.50079830000007, 45.58352836000005, 0, 0, 0), (-73.48585690000007, 45.58352836000005, 2, 0, 2), (-73.47091550000007, 45.58352836000005, 2, 1, 1), (-73.45597410000008, 45.58352836000005, 1, 0, 1), (-73.44103270000008, 45.58352836000005, 8, 2, 6), (-73.42609130000008, 45.58352836000005, 0, 0, 0), (-73.41114990000008, 45.58352836000005, 0, 0, 0), (-73.39620850000009, 45.58352836000005, 0, 0, 0), (-73.38126710000009, 45.58352836000005, 0, 0, 0), (-73.36632570000009, 45.58352836000005, 0, 0, 0), (-73.35138430000009, 45.58352836000005, 2, 2, 0), (-73.3364429000001, 45.58352836000005, 2, 1, 1), (-73.3215015000001, 45.58352836000005, 3, 2, 1), (-73.3065601000001, 45.58352836000005, 0, 0, 0), (-73.2916187000001, 45.58352836000005, 0, 0, 0), (-74.0237473, 45.593717080000054, 0, 0, 0), (-74.0088059, 45.593717080000054, 0, 0, 0), (-73.9938645, 45.593717080000054, 0, 0, 0), (-73.9789231, 45.593717080000054, 0, 0, 0), (-73.9639817, 45.593717080000054, 0, 0, 0), (-73.94904030000001, 45.593717080000054, 1, 0, 1), (-73.93409890000001, 45.593717080000054, 2, 1, 1), (-73.91915750000001, 45.593717080000054, 0, 0, 0), (-73.90421610000001, 45.593717080000054, 0, 0, 0), (-73.88927470000002, 45.593717080000054, 0, 0, 0), (-73.87433330000002, 45.593717080000054, 0, 0, 0), (-73.85939190000002, 45.593717080000054, 0, 0, 0), (-73.84445050000002, 45.593717080000054, 0, 0, 0), (-73.82950910000002, 45.593717080000054, 0, 0, 0), (-73.81456770000003, 45.593717080000054, 0, 0, 0), (-73.79962630000003, 45.593717080000054, 7, 6, 1), (-73.78468490000003, 45.593717080000054, 21, 5, 16), (-73.76974350000003, 45.593717080000054, 1, 0, 1), (-73.75480210000003, 45.593717080000054, 3, 1, 2), (-73.73986070000004, 45.593717080000054, 4, 1, 3), (-73.72491930000004, 45.593717080000054, 8, 8, 0), (-73.70997790000004, 45.593717080000054, 0, 0, 0), (-73.69503650000004, 45.593717080000054, 32, 8, 24), (-73.68009510000005, 45.593717080000054, 4, 0, 4), (-73.66515370000005, 45.593717080000054, 144, 123, 21), (-73.65021230000005, 45.593717080000054, 33, 22, 11), (-73.63527090000005, 45.593717080000054, 25, 9, 16), (-73.62032950000005, 45.593717080000054, 3, 1, 2), (-73.60538810000006, 45.593717080000054, 9, 3, 6), (-73.59044670000006, 45.593717080000054, 553, 537, 16), (-73.57550530000006, 45.593717080000054, 205, 108, 97), (-73.56056390000006, 45.593717080000054, 11, 8, 3), (-73.54562250000006, 45.593717080000054, 42, 17, 25), (-73.53068110000007, 45.593717080000054, 31, 14, 17), (-73.51573970000007, 45.593717080000054, 19, 9, 10), (-73.50079830000007, 45.593717080000054, 0, 0, 0), (-73.48585690000007, 45.593717080000054, 0, 0, 0), (-73.47091550000007, 45.593717080000054, 5, 4, 1), (-73.45597410000008, 45.593717080000054, 0, 0, 0), (-73.44103270000008, 45.593717080000054, 583, 197, 386), (-73.42609130000008, 45.593717080000054, 20, 16, 4), (-73.41114990000008, 45.593717080000054, 0, 0, 0), (-73.39620850000009, 45.593717080000054, 0, 0, 0), (-73.38126710000009, 45.593717080000054, 0, 0, 0), (-73.36632570000009, 45.593717080000054, 0, 0, 0), (-73.35138430000009, 45.593717080000054, 1, 1, 0), (-73.3364429000001, 45.593717080000054, 84, 38, 46), (-73.3215015000001, 45.593717080000054, 11, 5, 6), (-73.3065601000001, 45.593717080000054, 0, 0, 0), (-73.2916187000001, 45.593717080000054, 0, 0, 0), (-74.0237473, 45.60390580000006, 0, 0, 0), (-74.0088059, 45.60390580000006, 0, 0, 0), (-73.9938645, 45.60390580000006, 0, 0, 0), (-73.9789231, 45.60390580000006, 0, 0, 0), (-73.9639817,

45.60390580000006, 0, 0, 0), (-73.94904030000001, 45.60390580000006, 0, 0, 0), (-73.93409890000001, 45.60390580000006, 0, 0, 0), (-73.91915750000001, 45.60390580000006, 0, 0, 0), (-73.90421610000001, 45.60390580000006, 0, 0, 0), (-73.88927470000002, 45.60390580000006, 0, 0, 0), (-73.87433330000002, 45.60390580000006, 0, 0, 0), (-73.85939190000002, 45.60390580000006, 0, 0, 0), (-73.84445050000002, 45.60390580000006, 0, 0, 0), (-73.82950910000002, 45.60390580000006, 0, 0, 0), (-73.81456770000003, 45.60390580000006, 1, 1, 0), (-73.79962630000003, 45.60390580000006, 22, 18, 4), (-73.78468490000003, 45.60390580000006, 5, 4, 1), (-73.76974350000003, 45.60390580000006, 2, 2, 0), (-73.75480210000003, 45.60390580000006, 0, 0, 0), (-73.73986070000004, 45.60390580000006, 1912, 562, 1350), (-73.72491930000004, 45.60390580000006, 21, 3, 18), (-73.70997790000004, 45.60390580000006, 1730, 301, 1429), (-73.69503650000004, 45.60390580000006, 3, 3, 0), (-73.68009510000005, 45.60390580000006, 28, 27, 1), (-73.66515370000005, 45.60390580000006, 55, 26, 29), (-73.65021230000005, 45.60390580000006, 8, 6, 2), (-73.63527090000005, 45.60390580000006, 118, 82, 36), (-73.62032950000005, 45.60390580000006, 363, 317, 46), (-73.60538810000006, 45.60390580000006, 48, 20, 28), (-73.59044670000006, 45.60390580000006, 28, 13, 15), (-73.57550530000006, 45.60390580000006, 17, 9, 8), (-73.56056390000006, 45.60390580000006, 320, 22, 298), (-73.54562250000006, 45.60390580000006, 26, 14, 12), (-73.53068110000007, 45.60390580000006, 32, 15, 17), (-73.51573970000007, 45.60390580000006, 138, 111, 27), (-73.50079830000007, 45.60390580000006, 0, 0, 0), (-73.48585690000007, 45.60390580000006, 0, 0, 0), (-73.47091550000007, 45.60390580000006, 4, 1, 3), (-73.45597410000008, 45.60390580000006, 125, 92, 33), (-73.44103270000008, 45.60390580000006, 1, 1, 0), (-73.42609130000008, 45.60390580000006, 0, 0, 0), (-73.41114990000008, 45.60390580000006, 0, 0, 0), (-73.39620850000009, 45.60390580000006, 0, 0, 0), (-73.38126710000009, 45.60390580000006, 0, 0, 0), (-73.36632570000009, 45.60390580000006, 0, 0, 0), (-73.35138430000009, 45.60390580000006, 64, 14, 50), (-73.3364429000001, 45.60390580000006, 1, 1, 0), (-73.3215015000001, 45.60390580000006, 0, 0, 0), (-73.3065601000001, 45.60390580000006, 3, 0, 3), (-73.2916187000001, 45.60390580000006, 0, 0, 0), (-74.0237473, 45.61409452000006, 0, 0, 0), (-74.0088059, 45.61409452000006, 0, 0, 0), (-73.9938645, 45.61409452000006, 1, 0, 1), (-73.9789231, 45.61409452000006, 0, 0, 0), (-73.9639817, 45.61409452000006, 0, 0, 0), (-73.94904030000001, 45.61409452000006, 0, 0, 0), (-73.93409890000001, 45.61409452000006, 0, 0, 0), (-73.91915750000001, 45.61409452000006, 0, 0, 0), (-73.90421610000001, 45.61409452000006, 0, 0, 0), (-73.88927470000002, 45.61409452000006, 0, 0, 0), (-73.87433330000002, 45.61409452000006, 0, 0, 0), (-73.85939190000002, 45.61409452000006, 1, 0, 1), (-73.84445050000002, 45.61409452000006, 217, 79, 138), (-73.82950910000002, 45.61409452000006, 72, 21, 51), (-73.81456770000003, 45.61409452000006, 0, 0, 0), (-73.79962630000003, 45.61409452000006, 1, 0, 1), (-73.78468490000003, 45.61409452000006, 271, 147, 124), (-73.76974350000003, 45.61409452000006, 0, 0, 0), (-73.75480210000003, 45.61409452000006, 10, 5, 5), (-73.73986070000004, 45.61409452000006, 38, 4, 34), (-73.72491930000004, 45.61409452000006, 4, 1, 3), (-73.70997790000004, 45.61409452000006, 2, 2, 0), (-73.69503650000004, 45.61409452000006, 16, 1, 15), (-73.68009510000005, 45.61409452000006, 20, 15, 5), (-73.66515370000005, 45.61409452000006, 59, 59, 0), (-73.65021230000005, 45.61409452000006, 26, 21, 5), (-73.63527090000005, 45.61409452000006, 4, 3, 1), (-73.62032950000005, 45.61409452000006, 36, 29, 7), (-73.60538810000006, 45.61409452000006, 152, 72, 80), (-73.59044670000006, 45.61409452000006, 176, 39, 137), (-73.57550530000006, 45.61409452000006, 346, 6, 340), (-73.56056390000006, 45.61409452000006, 55, 16, 39), (-73.54562250000006, 45.61409452000006, 6, 4, 2), (-73.53068110000007, 45.61409452000006, 14, 14, 0), (-73.51573970000007, 45.61409452000006, 532, 15, 517), (-73.50079830000007, 45.61409452000006, 2, 1, 1), (-73.48585690000007, 45.61409452000006, 0, 0, 0),

(-73.47091550000007, 45.61409452000006, 28, 25, 3), (-73.45597410000008, 45.61409452000006, 5, 4, 1), (-73.44103270000008, 45.61409452000006, 5, 1, 4), (-73.42609130000008, 45.61409452000006, 0, 0, 0), (-73.41114990000008, 45.61409452000006, 0, 0, 0), (-73.39620850000009, 45.61409452000006, 0, 0, 0), (-73.38126710000009, 45.61409452000006, 0, 0, 0), (-73.36632570000009, 45.61409452000006, 0, 0, 0), (-73.35138430000009, 45.61409452000006, 0, 0, 0), (-73.3364429000001, 45.61409452000006, 0, 0, 0), (-73.3215015000001, 45.61409452000006, 0, 0, 0), (-73.3065601000001, 45.61409452000006, 0, 0, 0), (-73.2916187000001, 45.61409452000006, 0, 0, 0), (-74.0237473, 45.62428324000006, 0, 0, 0), (-74.0088059, 45.62428324000006, 0, 0, 0), (-73.9938645, 45.62428324000006, 0, 0, 0), (-73.9789231, 45.62428324000006, 0, 0, 0), (-73.9639817, 45.62428324000006, 0, 0, 0), (-73.94904030000001, 45.62428324000006, 0, 0, 0), (-73.93409890000001, 45.62428324000006, 0, 0, 0), (-73.91915750000001, 45.62428324000006, 0, 0, 0), (-73.90421610000001, 45.62428324000006, 0, 0, 0), (-73.88927470000002, 45.62428324000006, 0, 0, 0), (-73.87433330000002, 45.62428324000006, 0, 0, 0), (-73.85939190000002, 45.62428324000006, 82, 0, 82), (-73.84445050000002, 45.62428324000006, 56, 38, 18), (-73.82950910000002, 45.62428324000006, 3, 3, 0), (-73.81456770000003, 45.62428324000006, 3, 2, 1), (-73.79962630000003, 45.62428324000006, 44, 10, 34), (-73.78468490000003, 45.62428324000006, 0, 0, 0), (-73.76974350000003, 45.62428324000006, 4, 2, 2), (-73.75480210000003, 45.62428324000006, 7, 5, 2), (-73.73986070000004, 45.62428324000006, 2, 2, 0), (-73.72491930000004, 45.62428324000006, 1, 0, 1), (-73.70997790000004, 45.62428324000006, 0, 0, 0), (-73.69503650000004, 45.62428324000006, 1, 0, 1), (-73.68009510000005, 45.62428324000006, 7, 0, 7), (-73.66515370000005, 45.62428324000006, 0, 0, 0), (-73.65021230000005, 45.62428324000006, 0, 0, 0), (-73.63527090000005, 45.62428324000006, 1, 0, 1), (-73.62032950000005, 45.62428324000006, 27, 23, 4), (-73.60538810000006, 45.62428324000006, 64, 3, 61), (-73.59044670000006, 45.62428324000006, 162, 49, 113), (-73.57550530000006, 45.62428324000006, 3, 0, 3), (-73.56056390000006, 45.62428324000006, 10, 3, 7), (-73.54562250000006, 45.62428324000006, 220, 4, 216), (-73.53068110000007, 45.62428324000006, 0, 0, 0), (-73.51573970000007, 45.62428324000006, 0, 0, 0), (-73.50079830000007, 45.62428324000006, 0, 0, 0), (-73.48585690000007, 45.62428324000006, 0, 0, 0), (-73.47091550000007, 45.62428324000006, 0, 0, 0), (-73.45597410000008, 45.62428324000006, 0, 0, 0), (-73.44103270000008, 45.62428324000006, 37, 0, 37), (-73.42609130000008, 45.62428324000006, 0, 0, 0), (-73.41114990000008, 45.62428324000006, 0, 0, 0), (-73.39620850000009, 45.62428324000006, 0, 0, 0), (-73.38126710000009, 45.62428324000006, 0, 0, 0), (-73.36632570000009, 45.62428324000006, 0, 0, 0), (-73.35138430000009, 45.62428324000006, 0, 0, 0), (-73.3364429000001, 45.62428324000006, 0, 0, 0), (-73.3215015000001, 45.62428324000006, 0, 0, 0), (-73.3065601000001, 45.62428324000006, 0, 0, 0), (-73.2916187000001, 45.62428324000006, 0, 0, 0), (-74.0237473, 45.63447196000006, 0, 0, 0), (-74.0088059, 45.63447196000006, 0, 0, 0), (-73.9938645, 45.63447196000006, 0, 0, 0), (-73.9789231, 45.63447196000006, 0, 0, 0), (-73.9639817, 45.63447196000006, 0, 0, 0), (-73.94904030000001, 45.63447196000006, 0, 0, 0), (-73.93409890000001, 45.63447196000006, 0, 0, 0), (-73.91915750000001, 45.63447196000006, 0, 0, 0), (-73.90421610000001, 45.63447196000006, 1, 0, 1), (-73.88927470000002, 45.63447196000006, 0, 0, 0), (-73.87433330000002, 45.63447196000006, 4, 2, 2), (-73.85939190000002, 45.63447196000006, 74, 22, 52), (-73.84445050000002, 45.63447196000006, 24, 19, 5), (-73.82950910000002, 45.63447196000006, 2, 1, 1), (-73.81456770000003, 45.63447196000006, 36, 26, 10), (-73.79962630000003, 45.63447196000006, 5, 2, 3), (-73.78468490000003, 45.63447196000006, 221, 78, 143), (-73.76974350000003, 45.63447196000006, 1, 1, 0), (-73.75480210000003, 45.63447196000006, 73, 68, 5), (-73.73986070000004, 45.63447196000006, 0, 0, 0), (-73.72491930000004, 45.63447196000006, 0, 0, 0), (-73.70997790000004, 45.63447196000006, 0, 0, 0),

(-73.69503650000004, 45.63447196000006, 0, 0, 0), (-73.68009510000005, 45.63447196000006, 0, 0, 0), (-73.66515370000005, 45.63447196000006, 2, 1, 1), (-73.65021230000005, 45.63447196000006, 5, 1, 4), (-73.63527090000005, 45.63447196000006, 0, 0, 0), (-73.62032950000005, 45.63447196000006, 1, 0, 1), (-73.60538810000006, 45.63447196000006, 3, 2, 1), (-73.59044670000006, 45.63447196000006, 57, 21, 36), (-73.57550530000006, 45.63447196000006, 0, 0, 0), (-73.56056390000006, 45.63447196000006, 1, 0, 1), (-73.54562250000006, 45.63447196000006, 148, 1, 147), (-73.53068110000007, 45.63447196000006, 1, 0, 1), (-73.51573970000007, 45.63447196000006, 6, 4, 2), (-73.50079830000007, 45.63447196000006, 493, 68, 425), (-73.48585690000007, 45.63447196000006, 11, 4, 7), (-73.47091550000007, 45.63447196000006, 0, 0, 0), (-73.45597410000008, 45.63447196000006, 0, 0, 0), (-73.44103270000008, 45.63447196000006, 0, 0, 0), (-73.42609130000008, 45.63447196000006, 0, 0, 0), (-73.41114990000008, 45.63447196000006, 0, 0, 0), (-73.39620850000009, 45.63447196000006, 3, 3, 0), (-73.38126710000009, 45.63447196000006, 16, 15, 1), (-73.36632570000009, 45.63447196000006, 0, 0, 0), (-73.35138430000009, 45.63447196000006, 0, 0, 0), (-73.3364429000001, 45.63447196000006, 0, 0, 0), (-73.3215015000001, 45.63447196000006, 0, 0, 0), (-73.3065601000001, 45.63447196000006, 1, 1, 0), (-73.2916187000001, 45.63447196000006, 0, 0, 0), (-74.0237473, 45.644660680000065, 0, 0, 0), (-74.0088059, 45.644660680000065, 0, 0, 0), (-73.9938645, 45.644660680000065, 0, 0, 0), (-73.9789231, 45.644660680000065, 0, 0, 0), (-73.9639817, 45.644660680000065, 0, 0, 0), (-73.94904030000001, 45.644660680000065, 1, 1, 0), (-73.93409890000001, 45.644660680000065, 0, 0, 0), (-73.91915750000001, 45.644660680000065, 0, 0, 0), (-73.90421610000001, 45.644660680000065, 1, 0, 1), (-73.88927470000002, 45.644660680000065, 0, 0, 0), (-73.87433330000002, 45.644660680000065, 13, 1, 12), (-73.85939190000002, 45.644660680000065, 10, 2, 8), (-73.84445050000002, 45.644660680000065, 100, 53, 47), (-73.82950910000002, 45.644660680000065, 4, 3, 1), (-73.81456770000003, 45.644660680000065, 0, 0, 0), (-73.79962630000003, 45.644660680000065, 1, 1, 0), (-73.78468490000003, 45.644660680000065, 3, 1, 2), (-73.76974350000003, 45.644660680000065, 3, 0, 3), (-73.75480210000003, 45.644660680000065, 3, 1, 2), (-73.73986070000004, 45.644660680000065, 1, 1, 0), (-73.72491930000004, 45.644660680000065, 2, 1, 1), (-73.70997790000004, 45.644660680000065, 0, 0, 0), (-73.69503650000004, 45.644660680000065, 0, 0, 0), (-73.68009510000005, 45.644660680000065, 0, 0, 0), (-73.66515370000005, 45.644660680000065, 0, 0, 0), (-73.65021230000005, 45.644660680000065, 1, 1, 0), (-73.63527090000005, 45.644660680000065, 0, 0, 0), (-73.62032950000005, 45.644660680000065, 0, 0, 0), (-73.60538810000006, 45.644660680000065, 0, 0, 0), (-73.59044670000006, 45.644660680000065, 6, 1, 5), (-73.57550530000006, 45.644660680000065, 19, 3, 16), (-73.56056390000006, 45.644660680000065, 1, 1, 0), (-73.54562250000006, 45.644660680000065, 2, 0, 2), (-73.53068110000007, 45.644660680000065, 0, 0, 0), (-73.51573970000007, 45.644660680000065, 5, 2, 3), (-73.50079830000007, 45.644660680000065, 36, 16, 20), (-73.48585690000007, 45.644660680000065, 8, 5, 3), (-73.47091550000007, 45.644660680000065, 0, 0, 0), (-73.45597410000008, 45.644660680000065, 0, 0, 0), (-73.44103270000008, 45.644660680000065, 2, 2, 0), (-73.42609130000008, 45.644660680000065, 0, 0, 0), (-73.41114990000008, 45.644660680000065, 0, 0, 0), (-73.39620850000009, 45.644660680000065, 0, 0, 0), (-73.38126710000009, 45.644660680000065, 0, 0, 0), (-73.36632570000009, 45.644660680000065, 0, 0, 0), (-73.35138430000009, 45.644660680000065, 0, 0, 0), (-73.3364429000001, 45.644660680000065, 0, 0, 0), (-73.3215015000001, 45.644660680000065, 0, 0, 0), (-73.3065601000001, 45.644660680000065, 0, 0, 0), (-73.2916187000001, 45.644660680000065, 0, 0, 0), (-74.0237473, 45.65484940000007, 0, 0, 0), (-74.0088059, 45.65484940000007, 0, 0, 0), (-73.9938645, 45.65484940000007, 0, 0, 0),

(-73.9789231, 45.65484940000007, 0, 0, 0), (-73.9639817, 45.65484940000007, 0, 0, 0), (-73.94904030000001, 45.65484940000007, 0, 0, 0), (-73.93409890000001, 45.65484940000007, 0, 0, 0), (-73.91915750000001, 45.65484940000007, 0, 0, 0), (-73.90421610000001, 45.65484940000007, 8, 5, 3), (-73.88927470000002, 45.65484940000007, 0, 0, 0), (-73.87433330000002, 45.65484940000007, 0, 0, 0), (-73.85939190000002, 45.65484940000007, 12, 1, 11), (-73.84445050000002, 45.65484940000007, 0, 0, 0), (-73.82950910000002, 45.65484940000007, 0, 0, 0), (-73.81456770000003, 45.65484940000007, 1, 0, 1), (-73.79962630000003, 45.65484940000007, 19, 6, 13), (-73.78468490000003, 45.65484940000007, 5, 3, 2), (-73.76974350000003, 45.65484940000007, 24, 7, 17), (-73.75480210000003, 45.65484940000007, 0, 0, 0), (-73.73986070000004, 45.65484940000007, 0, 0, 0), (-73.72491930000004, 45.65484940000007, 0, 0, 0), (-73.70997790000004, 45.65484940000007, 0, 0, 0), (-73.69503650000004, 45.65484940000007, 0, 0, 0), (-73.68009510000005, 45.65484940000007, 0, 0, 0), (-73.66515370000005, 45.65484940000007, 0, 0, 0), (-73.65021230000005, 45.65484940000007, 0, 0, 0), (-73.63527090000005, 45.65484940000007, 0, 0, 0), (-73.62032950000005, 45.65484940000007, 0, 0, 0), (-73.60538810000006, 45.65484940000007, 3, 2, 1), (-73.59044670000006, 45.65484940000007, 1, 1, 0), (-73.57550530000006, 45.65484940000007, 27, 2, 25), (-73.56056390000006, 45.65484940000007, 17, 12, 5), (-73.54562250000006, 45.65484940000007, 1, 0, 1), (-73.53068110000007, 45.65484940000007, 156, 4, 152), (-73.51573970000007, 45.65484940000007, 171, 119, 52), (-73.50079830000007, 45.65484940000007, 83, 4, 79), (-73.48585690000007, 45.65484940000007, 8, 7, 1), (-73.47091550000007, 45.65484940000007, 0, 0, 0), (-73.45597410000008, 45.65484940000007, 0, 0, 0), (-73.44103270000008, 45.65484940000007, 2, 2, 0), (-73.42609130000008, 45.65484940000007, 0, 0, 0), (-73.41114990000008, 45.65484940000007, 1, 0, 1), (-73.39620850000009, 45.65484940000007, 0, 0, 0), (-73.38126710000009, 45.65484940000007, 0, 0, 0), (-73.36632570000009, 45.65484940000007, 0, 0, 0), (-73.35138430000009, 45.65484940000007, 0, 0, 0), (-73.3364429000001, 45.65484940000007, 0, 0, 0), (-73.3215015000001, 45.65484940000007, 0, 0, 0), (-73.3065601000001, 45.65484940000007, 8, 7, 1), (-73.2916187000001, 45.65484940000007, 0, 0, 0), (-74.0237473, 45.66503812000007, 0, 0, 0), (-74.0088059, 45.66503812000007, 26, 7, 19), (-73.9938645, 45.66503812000007, 0, 0, 0), (-73.9789231, 45.66503812000007, 0, 0, 0), (-73.9639817, 45.66503812000007, 0, 0, 0), (-73.94904030000001, 45.66503812000007, 0, 0, 0), (-73.93409890000001, 45.66503812000007, 1, 1, 0), (-73.91915750000001, 45.66503812000007, 1, 1, 0), (-73.90421610000001, 45.66503812000007, 0, 0, 0), (-73.88927470000002, 45.66503812000007, 6, 6, 0), (-73.87433330000002, 45.66503812000007, 1736, 445, 1291), (-73.85939190000002, 45.66503812000007, 390, 1, 389), (-73.84445050000002, 45.66503812000007, 2, 2, 0), (-73.82950910000002, 45.66503812000007, 0, 0, 0), (-73.81456770000003, 45.66503812000007, 10, 10, 0), (-73.79962630000003, 45.66503812000007, 0, 0, 0), (-73.78468490000003, 45.66503812000007, 2, 0, 2), (-73.76974350000003, 45.66503812000007, 0, 0, 0), (-73.75480210000003, 45.66503812000007, 12, 3, 9), (-73.73986070000004, 45.66503812000007, 1, 1, 0), (-73.72491930000004, 45.66503812000007, 0, 0, 0), (-73.70997790000004, 45.66503812000007, 3, 2, 1), (-73.69503650000004, 45.66503812000007, 0, 0, 0), (-73.68009510000005, 45.66503812000007, 0, 0, 0), (-73.66515370000005, 45.66503812000007, 0, 0, 0), (-73.65021230000005, 45.66503812000007, 0, 0, 0), (-73.63527090000005, 45.66503812000007, 2, 2, 0), (-73.62032950000005, 45.66503812000007, 0, 0, 0), (-73.60538810000006, 45.66503812000007, 0, 0, 0), (-73.59044670000006, 45.66503812000007, 16, 10, 6), (-73.57550530000006, 45.66503812000007, 1, 1, 0), (-73.56056390000006, 45.66503812000007, 180, 42, 138), (-73.54562250000006, 45.66503812000007, 9, 5, 4), (-73.53068110000007, 45.66503812000007, 1, 0, 1), (-73.51573970000007, 45.66503812000007, 3, 2, 1), (-73.50079830000007, 45.66503812000007, 27, 20, 7), (-73.48585690000007, 45.66503812000007, 0, 0, 0), (-73.47091550000007, 45.66503812000007, 0, 0, 0), (-73.45597410000008, 45.66503812000007,

0, 0, 0), (-73.44103270000008, 45.66503812000007, 0, 0, 0), (-73.42609130000008, 45.66503812000007, 2, 1, 1), (-73.41114990000008, 45.66503812000007, 0, 0, 0), (-73.39620850000009, 45.66503812000007, 0, 0, 0), (-73.38126710000009, 45.66503812000007, 0, 0, 0), (-73.36632570000009, 45.66503812000007, 0, 0, 0), (-73.35138430000009, 45.66503812000007, 0, 0, 0), (-73.3364429000001, 45.66503812000007, 0, 0, 0), (-73.3215015000001, 45.66503812000007, 0, 0, 0), (-73.3065601000001, 45.66503812000007, 0, 0, 0), (-73.2916187000001, 45.66503812000007, 0, 0, 0), (-74.0237473, 45.67522684000007, 0, 0, 0), (-74.0088059, 45.67522684000007, 0, 0, 0), (-73.9938645, 45.67522684000007, 0, 0, 0), (-73.9789231, 45.67522684000007, 8, 8, 0), (-73.9639817, 45.67522684000007, 0, 0, 0), (-73.94904030000001, 45.67522684000007, 0, 0, 0), (-73.93409890000001, 45.67522684000007, 1, 1, 0), (-73.91915750000001, 45.67522684000007, 16, 3, 13), (-73.90421610000001, 45.67522684000007, 10, 0, 10), (-73.88927470000002, 45.67522684000007, 0, 0, 0), (-73.87433330000002, 45.67522684000007, 7, 3, 4), (-73.85939190000002, 45.67522684000007, 0, 0, 0), (-73.84445050000002, 45.67522684000007, 0, 0, 0), (-73.82950910000002, 45.67522684000007, 0, 0, 0), (-73.81456770000003, 45.67522684000007, 0, 0, 0), (-73.79962630000003, 45.67522684000007, 0, 0, 0), (-73.78468490000003, 45.67522684000007, 2, 2, 0), (-73.76974350000003, 45.67522684000007, 13, 3, 10), (-73.75480210000003, 45.67522684000007, 1, 0, 1), (-73.73986070000004, 45.67522684000007, 0, 0, 0), (-73.72491930000004, 45.67522684000007, 2, 2, 0), (-73.70997790000004, 45.67522684000007, 0, 0, 0), (-73.69503650000004, 45.67522684000007, 0, 0, 0), (-73.68009510000005, 45.67522684000007, 0, 0, 0), (-73.66515370000005, 45.67522684000007, 0, 0, 0), (-73.65021230000005, 45.67522684000007, 0, 0, 0), (-73.63527090000005, 45.67522684000007, 0, 0, 0), (-73.62032950000005, 45.67522684000007, 0, 0, 0), (-73.60538810000006, 45.67522684000007, 2, 0, 2), (-73.59044670000006, 45.67522684000007, 1, 0, 1), (-73.57550530000006, 45.67522684000007, 2, 2, 0), (-73.56056390000006, 45.67522684000007, 0, 0, 0), (-73.54562250000006, 45.67522684000007, 0, 0, 0), (-73.53068110000007, 45.67522684000007, 0, 0, 0), (-73.51573970000007, 45.67522684000007, 0, 0, 0), (-73.50079830000007, 45.67522684000007, 52, 49, 3), (-73.48585690000007, 45.67522684000007, 2, 2, 0), (-73.47091550000007, 45.67522684000007, 0, 0, 0), (-73.45597410000008, 45.67522684000007, 0, 0, 0), (-73.44103270000008, 45.67522684000007, 1, 1, 0), (-73.42609130000008, 45.67522684000007, 2, 1, 1), (-73.41114990000008, 45.67522684000007, 0, 0, 0), (-73.39620850000009, 45.67522684000007, 0, 0, 0), (-73.38126710000009, 45.67522684000007, 0, 0, 0), (-73.36632570000009, 45.67522684000007, 0, 0, 0), (-73.35138430000009, 45.67522684000007, 0, 0, 0), (-73.3364429000001, 45.67522684000007, 0, 0, 0), (-73.3215015000001, 45.67522684000007, 0, 0, 0), (-73.3065601000001, 45.67522684000007, 0, 0, 0), (-73.2916187000001, 45.67522684000007, 0, 0, 0), (-74.0237473, 45.685415560000074, 25, 2, 23), (-74.0088059, 45.685415560000074, 0, 0, 0), (-73.9938645, 45.685415560000074, 0, 0, 0), (-73.9789231, 45.685415560000074, 0, 0, 0), (-73.9639817, 45.685415560000074, 0, 0, 0), (-73.94904030000001, 45.685415560000074, 0, 0, 0), (-73.93409890000001, 45.685415560000074, 0, 0, 0), (-73.91915750000001, 45.685415560000074, 2, 2, 0), (-73.90421610000001, 45.685415560000074, 2, 0, 2), (-73.88927470000002, 45.685415560000074, 1, 1, 0), (-73.87433330000002, 45.685415560000074, 94, 67, 27), (-73.85939190000002, 45.685415560000074, 0, 0, 0), (-73.84445050000002, 45.685415560000074, 0, 0, 0), (-73.82950910000002, 45.685415560000074, 0, 0, 0), (-73.81456770000003, 45.685415560000074, 1, 1, 0), (-73.79962630000003, 45.685415560000074, 0, 0, 0), (-73.78468490000003, 45.685415560000074, 0, 0, 0), (-73.76974350000003, 45.685415560000074, 0, 0, 0), (-73.75480210000003, 45.685415560000074, 0, 0, 0), (-73.73986070000004, 45.685415560000074, 0, 0, 0), (-73.72491930000004, 45.685415560000074, 0, 0, 0), (-73.70997790000004, 45.685415560000074, 0, 0, 0), (-73.69503650000004, 45.685415560000074, 0, 0, 0), (-73.68009510000005, 45.685415560000074, 0, 0, 0),

(-73.66515370000005, 45.685415560000074, 0, 0, 0), (-73.65021230000005, 45.685415560000074, 2, 2, 0), (-73.63527090000005, 45.685415560000074, 13, 7, 6), (-73.62032950000005, 45.685415560000074, 0, 0, 0), (-73.60538810000006, 45.685415560000074, 0, 0, 0), (-73.59044670000006, 45.685415560000074, 0, 0, 0), (-73.57550530000006, 45.685415560000074, 0, 0, 0), (-73.56056390000006, 45.685415560000074, 0, 0, 0), (-73.54562250000006, 45.685415560000074, 0, 0, 0), (-73.53068110000007, 45.685415560000074, 1, 1, 0), (-73.51573970000007, 45.685415560000074, 9, 8, 1), (-73.50079830000007, 45.685415560000074, 6, 5, 1), (-73.48585690000007, 45.685415560000074, 0, 0, 0), (-73.47091550000007, 45.685415560000074, 0, 0, 0), (-73.45597410000008, 45.685415560000074, 0, 0, 0), (-73.44103270000008, 45.685415560000074, 46, 25, 21), (-73.42609130000008, 45.685415560000074, 22, 14, 8), (-73.41114990000008, 45.685415560000074, 0, 0, 0), (-73.39620850000009, 45.685415560000074, 0, 0, 0), (-73.38126710000009, 45.685415560000074, 0, 0, 0), (-73.36632570000009, 45.685415560000074, 0, 0, 0), (-73.35138430000009, 45.685415560000074, 0, 0, 0), (-73.3364429000001, 45.685415560000074, 0, 0, 0), (-73.3215015000001, 45.685415560000074, 0, 0, 0), (-73.3065601000001, 45.685415560000074, 0, 0, 0), (-73.2916187000001, 45.685415560000074, 0, 0, 0), (-74.0237473, 45.695604280000076, 4, 0, 4), (-74.0088059, 45.695604280000076, 0, 0, 0), (-73.9938645, 45.695604280000076, 0, 0, 0), (-73.9789231, 45.695604280000076, 0, 0, 0), (-73.9639817, 45.695604280000076, 0, 0, 0), (-73.94904030000001, 45.695604280000076, 0, 0, 0), (-73.93409890000001, 45.695604280000076, 0, 0, 0), (-73.91915750000001, 45.695604280000076, 0, 0, 0), (-73.90421610000001, 45.695604280000076, 0, 0, 0), (-73.88927470000002, 45.695604280000076, 0, 0, 0), (-73.87433330000002, 45.695604280000076, 0, 0, 0), (-73.85939190000002, 45.695604280000076, 0, 0, 0), (-73.84445050000002, 45.695604280000076, 0, 0, 0), (-73.82950910000002, 45.695604280000076, 0, 0, 0), (-73.81456770000003, 45.695604280000076, 1, 0, 1), (-73.79962630000003, 45.695604280000076, 0, 0, 0), (-73.78468490000003, 45.695604280000076, 0, 0, 0), (-73.76974350000003, 45.695604280000076, 0, 0, 0), (-73.75480210000003, 45.695604280000076, 0, 0, 0), (-73.73986070000004, 45.695604280000076, 0, 0, 0), (-73.72491930000004, 45.695604280000076, 0, 0, 0), (-73.70997790000004, 45.695604280000076, 19, 14, 5), (-73.69503650000004, 45.695604280000076, 0, 0, 0), (-73.68009510000005, 45.695604280000076, 0, 0, 0), (-73.66515370000005, 45.695604280000076, 0, 0, 0), (-73.65021230000005, 45.695604280000076, 6, 4, 2), (-73.63527090000005, 45.695604280000076, 79, 44, 35), (-73.62032950000005, 45.695604280000076, 0, 0, 0), (-73.60538810000006, 45.695604280000076, 138, 1, 137), (-73.59044670000006, 45.695604280000076, 0, 0, 0), (-73.57550530000006, 45.695604280000076, 0, 0, 0), (-73.56056390000006, 45.695604280000076, 0, 0, 0), (-73.54562250000006, 45.695604280000076, 0, 0, 0), (-73.53068110000007, 45.695604280000076, 1, 1, 0), (-73.51573970000007, 45.695604280000076, 2, 0, 2), (-73.50079830000007, 45.695604280000076, 0, 0, 0), (-73.48585690000007, 45.695604280000076, 2, 1, 1), (-73.47091550000007, 45.695604280000076, 0, 0, 0), (-73.45597410000008, 45.695604280000076, 0, 0, 0), (-73.44103270000008, 45.695604280000076, 1, 1, 0), (-73.42609130000008, 45.695604280000076, 0, 0, 0), (-73.41114990000008, 45.695604280000076, 0, 0, 0), (-73.39620850000009, 45.695604280000076, 0, 0, 0), (-73.38126710000009, 45.695604280000076, 0, 0, 0), (-73.36632570000009, 45.695604280000076, 0, 0, 0), (-73.35138430000009, 45.695604280000076, 0, 0, 0), (-73.3364429000001, 45.695604280000076, 0, 0, 0), (-73.3215015000001, 45.695604280000076, 0, 0, 0), (-73.3065601000001, 45.695604280000076, 0, 0, 0), (-73.2916187000001, 45.695604280000076, 0, 0, 0), (-74.0237473, 45.70579300000008, 0, 0, 0), (-74.0088059, 45.70579300000008, 0, 0, 0), (-73.9938645, 45.70579300000008, 0, 0, 0), (-73.9789231, 45.70579300000008, 0, 0, 0), (-73.9639817,

45.70579300000008, 0, 0, 0), (-73.94904030000001, 45.70579300000008, 0, 0, 0), (-73.93409890000001, 45.70579300000008, 0, 0, 0), (-73.91915750000001, 45.70579300000008, 0, 0, 0), (-73.90421610000001, 45.70579300000008, 0, 0, 0), (-73.88927470000002, 45.70579300000008, 0, 0, 0), (-73.87433330000002, 45.70579300000008, 0, 0, 0), (-73.85939190000002, 45.70579300000008, 0, 0, 0), (-73.84445050000002, 45.70579300000008, 0, 0, 0), (-73.82950910000002, 45.70579300000008, 0, 0, 0), (-73.81456770000003, 45.70579300000008, 0, 0, 0), (-73.79962630000003, 45.70579300000008, 0, 0, 0), (-73.78468490000003, 45.70579300000008, 0, 0, 0), (-73.76974350000003, 45.70579300000008, 0, 0, 0), (-73.75480210000003, 45.70579300000008, 0, 0, 0), (-73.73986070000004, 45.70579300000008, 0, 0, 0), (-73.72491930000004, 45.70579300000008, 0, 0, 0), (-73.70997790000004, 45.70579300000008, 0, 0, 0), (-73.69503650000004, 45.70579300000008, 0, 0, 0), (-73.68009510000005, 45.70579300000008, 0, 0, 0), (-73.66515370000005, 45.70579300000008, 5, 4, 1), (-73.65021230000005, 45.70579300000008, 146, 135, 11), (-73.63527090000005, 45.70579300000008, 2, 0, 2), (-73.62032950000005, 45.70579300000008, 1, 0, 1), (-73.60538810000006, 45.70579300000008, 0, 0, 0), (-73.59044670000006, 45.70579300000008, 0, 0, 0), (-73.57550530000006, 45.70579300000008, 0, 0, 0), (-73.56056390000006, 45.70579300000008, 0, 0, 0), (-73.54562250000006, 45.70579300000008, 0, 0, 0), (-73.53068110000007, 45.70579300000008, 0, 0, 0), (-73.51573970000007, 45.70579300000008, 3, 1, 2), (-73.50079830000007, 45.70579300000008, 0, 0, 0), (-73.48585690000007, 45.70579300000008, 0, 0, 0), (-73.47091550000007, 45.70579300000008, 5, 3, 2), (-73.45597410000008, 45.70579300000008, 0, 0, 0), (-73.44103270000008, 45.70579300000008, 0, 0, 0), (-73.42609130000008, 45.70579300000008, 0, 0, 0), (-73.41114990000008, 45.70579300000008, 0, 0, 0), (-73.39620850000009, 45.70579300000008, 0, 0, 0), (-73.38126710000009, 45.70579300000008, 0, 0, 0), (-73.36632570000009, 45.70579300000008, 0, 0, 0), (-73.35138430000009, 45.70579300000008, 0, 0, 0), (-73.3364429000001, 45.70579300000008, 0, 0, 0), (-73.3215015000001, 45.70579300000008, 0, 0, 0), (-73.3065601000001, 45.70579300000008, 0, 0, 0), (-73.2916187000001, 45.70579300000008, 0, 0, 0), (-74.0237473, 45.71598172000008, 0, 0, 0), (-74.0088059, 45.71598172000008, 0, 0, 0), (-73.9938645, 45.71598172000008, 0, 0, 0), (-73.9789231, 45.71598172000008, 0, 0, 0), (-73.9639817, 45.71598172000008, 0, 0, 0), (-73.94904030000001, 45.71598172000008, 0, 0, 0), (-73.93409890000001, 45.71598172000008, 0, 0, 0), (-73.91915750000001, 45.71598172000008, 0, 0, 0), (-73.90421610000001, 45.71598172000008, 1, 1, 0), (-73.88927470000002, 45.71598172000008, 0, 0, 0), (-73.87433330000002, 45.71598172000008, 0, 0, 0), (-73.85939190000002, 45.71598172000008, 0, 0, 0), (-73.84445050000002, 45.71598172000008, 0, 0, 0), (-73.82950910000002, 45.71598172000008, 0, 0, 0), (-73.81456770000003, 45.71598172000008, 0, 0, 0), (-73.79962630000003, 45.71598172000008, 0, 0, 0), (-73.78468490000003, 45.71598172000008, 0, 0, 0), (-73.76974350000003, 45.71598172000008, 0, 0, 0), (-73.75480210000003, 45.71598172000008, 0, 0, 0), (-73.73986070000004, 45.71598172000008, 0, 0, 0), (-73.72491930000004, 45.71598172000008, 0, 0, 0), (-73.70997790000004, 45.71598172000008, 28, 14, 14), (-73.69503650000004, 45.71598172000008, 4, 0, 4), (-73.68009510000005, 45.71598172000008, 0, 0, 0), (-73.66515370000005, 45.71598172000008, 1, 1, 0), (-73.65021230000005, 45.71598172000008, 3, 2, 1), (-73.63527090000005, 45.71598172000008, 0, 0, 0), (-73.62032950000005, 45.71598172000008, 0, 0, 0), (-73.60538810000006, 45.71598172000008, 2, 0, 2), (-73.59044670000006, 45.71598172000008, 0, 0, 0), (-73.57550530000006, 45.71598172000008, 0, 0, 0), (-73.56056390000006, 45.71598172000008, 0, 0, 0), (-73.54562250000006, 45.71598172000008, 46, 26, 20), (-73.53068110000007, 45.71598172000008, 0, 0, 0), (-73.51573970000007, 45.71598172000008, 75, 14, 61), (-73.50079830000007, 45.71598172000008, 4, 1, 3), (-73.48585690000007, 45.71598172000008, 10, 3, 7), (-73.47091550000007, 45.71598172000008, 7, 3, 4), (-73.4559741000008, 45.71598172000008, 0, 0, 0), (-73.44103270000008, 45.71598172000008, 0, 0, 0),

(-73.42609130000008, 45.71598172000008, 0, 0, 0), (-73.41114990000008, 45.71598172000008, 0, 0, 0), (-73.39620850000009, 45.71598172000008, 0, 0, 0), (-73.38126710000009, 45.71598172000008, 0, 0, 0), (-73.36632570000009, 45.71598172000008, 0, 0, 0), (-73.35138430000009, 45.71598172000008, 0, 0, 0), (-73.3364429000001, 45.71598172000008, 0, 0, 0), (-73.3215015000001, 45.71598172000008, 0, 0, 0), (-73.3065601000001, 45.71598172000008, 0, 0, 0), (-73.2916187000001, 45.71598172000008, 0, 0, 0), (-74.0237473, 45.72617044000008, 0, 0, 0), (-74.0088059, 45.72617044000008, 0, 0, 0), (-73.9938645, 45.72617044000008, 0, 0, 0), (-73.9789231, 45.72617044000008, 0, 0, 0), (-73.9639817, 45.72617044000008, 0, 0, 0), (-73.94904030000001, 45.72617044000008, 0, 0, 0), (-73.93409890000001, 45.72617044000008, 0, 0, 0), (-73.91915750000001, 45.72617044000008, 0, 0, 0), (-73.90421610000001, 45.72617044000008, 0, 0, 0), (-73.88927470000002, 45.72617044000008, 0, 0, 0), (-73.87433330000002, 45.72617044000008, 0, 0, 0), (-73.85939190000002, 45.72617044000008, 0, 0, 0), (-73.84445050000002, 45.72617044000008, 0, 0, 0), (-73.82950910000002, 45.72617044000008, 9, 7, 2), (-73.81456770000003, 45.72617044000008, 0, 0, 0), (-73.79962630000003, 45.72617044000008, 0, 0, 0), (-73.78468490000003, 45.72617044000008, 0, 0, 0), (-73.76974350000003, 45.72617044000008, 0, 0, 0), (-73.75480210000003, 45.72617044000008, 0, 0, 0), (-73.73986070000004, 45.72617044000008, 0, 0, 0), (-73.72491930000004, 45.72617044000008, 0, 0, 0), (-73.70997790000004, 45.72617044000008, 0, 0, 0), (-73.69503650000004, 45.72617044000008, 2, 1, 1), (-73.68009510000005, 45.72617044000008, 1, 0, 1), (-73.66515370000005, 45.72617044000008, 0, 0, 0), (-73.65021230000005, 45.72617044000008, 0, 0, 0), (-73.63527090000005, 45.72617044000008, 3, 2, 1), (-73.62032950000005, 45.72617044000008, 59, 0, 59), (-73.60538810000006, 45.72617044000008, 0, 0, 0), (-73.59044670000006, 45.72617044000008, 0, 0, 0), (-73.57550530000006, 45.72617044000008, 0, 0, 0), (-73.56056390000006, 45.72617044000008, 0, 0, 0), (-73.54562250000006, 45.72617044000008, 0, 0, 0), (-73.53068110000007, 45.72617044000008, 0, 0, 0), (-73.51573970000007, 45.72617044000008, 1, 0, 1), (-73.50079830000007, 45.72617044000008, 2, 2, 0), (-73.48585690000007, 45.72617044000008, 1, 1, 0), (-73.47091550000007, 45.72617044000008, 5, 2, 3), (-73.45597410000008, 45.72617044000008, 5, 1, 4), (-73.44103270000008, 45.72617044000008, 0, 0, 0), (-73.42609130000008, 45.72617044000008, 0, 0, 0), (-73.41114990000008, 45.72617044000008, 0, 0, 0), (-73.39620850000009, 45.72617044000008, 0, 0, 0), (-73.38126710000009, 45.72617044000008, 0, 0, 0), (-73.36632570000009, 45.72617044000008, 0, 0, 0), (-73.35138430000009, 45.72617044000008, 0, 0, 0), (-73.3364429000001, 45.72617044000008, 0, 0, 0), (-73.3215015000001, 45.72617044000008, 0, 0, 0), (-73.3065601000001, 45.72617044000008, 0, 0, 0), (-73.2916187000001, 45.72617044000008, 0, 0, 0), (-74.0237473, 45.736359160000085, 0, 0, 0), (-74.0088059, 45.736359160000085, 0, 0, 0), (-73.9938645, 45.736359160000085, 0, 0, 0), (-73.9789231, 45.736359160000085, 0, 0, 0), (-73.9639817, 45.736359160000085, 0, 0, 0), (-73.94904030000001, 45.736359160000085, 0, 0, 0), (-73.93409890000001, 45.736359160000085, 0, 0, 0), (-73.91915750000001, 45.736359160000085, 0, 0, 0), (-73.90421610000001, 45.736359160000085, 0, 0, 0), (-73.88927470000002, 45.736359160000085, 0, 0, 0), (-73.87433330000002, 45.736359160000085, 0, 0, 0), (-73.85939190000002, 45.736359160000085, 0, 0, 0), (-73.84445050000002, 45.736359160000085, 0, 0, 0), (-73.82950910000002, 45.736359160000085, 0, 0, 0), (-73.81456770000003, 45.736359160000085, 0, 0, 0), (-73.79962630000003, 45.736359160000085, 0, 0, 0), (-73.78468490000003, 45.736359160000085, 0, 0, 0), (-73.76974350000003, 45.736359160000085, 0, 0, 0), (-73.75480210000003, 45.736359160000085, 0, 0, 0), (-73.73986070000004, 45.736359160000085, 0, 0, 0), (-73.72491930000004, 45.736359160000085, 9, 6, 3), (-73.70997790000004, 45.736359160000085, 0, 0, 0), (-73.69503650000004, 45.736359160000085, 0, 0, 0), (-73.68009510000005, 45.736359160000085, 2, 1, 1), (-73.66515370000005,

45.736359160000085, 0, 0, 0), (-73.65021230000005, 45.736359160000085, 280, 46, 234), (-73.63527090000005, 45.736359160000085, 0, 0, 0), (-73.62032950000005, 45.736359160000085, 28, 0, 28), (-73.60538810000006, 45.736359160000085, 2, 2, 0), (-73.59044670000006, 45.736359160000085, 0, 0, 0), (-73.57550530000006, 45.736359160000085, 0, 0, 0), (-73.56056390000006, 45.736359160000085, 0, 0, 0), (-73.54562250000006, 45.736359160000085, 0, 0, 0), (-73.53068110000007, 45.736359160000085, 0, 0, 0), (-73.51573970000007, 45.736359160000085, 0, 0, 0), (-73.50079830000007, 45.736359160000085, 0, 0, 0), (-73.48585690000007, 45.736359160000085, 0, 0, 0), (-73.47091550000007, 45.736359160000085, 2, 1, 1), (-73.45597410000008, 45.736359160000085, 112, 48, 64), (-73.44103270000008, 45.736359160000085, 19, 17, 2), (-73.42609130000008, 45.736359160000085, 0, 0, 0), (-73.41114990000008, 45.736359160000085, 0, 0, 0), (-73.39620850000009, 45.736359160000085, 0, 0, 0), (-73.38126710000009, 45.736359160000085, 0, 0, 0), (-73.36632570000009, 45.736359160000085, 0, 0, 0), (-73.35138430000009, 45.736359160000085, 0, 0, 0), (-73.3364429000001, 45.736359160000085, 0, 0, 0), (-73.3215015000001, 45.736359160000085, 0, 0, 0), (-73.3065601000001, 45.736359160000085, 0, 0, 0), (-73.2916187000001, 45.736359160000085, 0, 0, 0), (-74.0237473, 45.74654788000009, 0, 0, 0), (-74.0088059, 45.74654788000009, 0, 0, 0), (-73.9938645, 45.74654788000009, 3, 3, 0), (-73.9789231, 45.74654788000009, 0, 0, 0), (-73.9639817, 45.74654788000009, 0, 0, 0), (-73.94904030000001, 45.74654788000009, 0, 0, 0), (-73.93409890000001, 45.74654788000009, 0, 0, 0), (-73.91915750000001, 45.74654788000009, 0, 0, 0), (-73.90421610000001, 45.74654788000009, 0, 0, 0), (-73.88927470000002, 45.74654788000009, 0, 0, 0), (-73.87433330000002, 45.74654788000009, 0, 0, 0), (-73.85939190000002, 45.74654788000009, 0, 0, 0), (-73.84445050000002, 45.74654788000009, 0, 0, 0), (-73.82950910000002, 45.74654788000009, 0, 0, 0), (-73.81456770000003, 45.74654788000009, 0, 0, 0), (-73.79962630000003, 45.74654788000009, 0, 0, 0), (-73.78468490000003, 45.74654788000009, 0, 0, 0), (-73.76974350000003, 45.74654788000009, 0, 0, 0), (-73.75480210000003, 45.74654788000009, 0, 0, 0), (-73.73986070000004, 45.74654788000009, 0, 0, 0), (-73.72491930000004, 45.74654788000009, 18, 11, 7), (-73.70997790000004, 45.74654788000009, 0, 0, 0), (-73.69503650000004, 45.74654788000009, 0, 0, 0), (-73.68009510000005, 45.74654788000009, 0, 0, 0), (-73.66515370000005, 45.74654788000009, 0, 0, 0), (-73.65021230000005, 45.74654788000009, 0, 0, 0), (-73.63527090000005, 45.74654788000009, 0, 0, 0), (-73.62032950000005, 45.74654788000009, 4, 3, 1), (-73.60538810000006, 45.74654788000009, 32, 19, 13), (-73.59044670000006, 45.74654788000009, 0, 0, 0), (-73.57550530000006, 45.74654788000009, 1, 0, 1), (-73.56056390000006, 45.74654788000009, 0, 0, 0), (-73.54562250000006, 45.74654788000009, 0, 0, 0), (-73.53068110000007, 45.74654788000009, 0, 0, 0), (-73.51573970000007, 45.74654788000009, 0, 0, 0), (-73.50079830000007, 45.74654788000009, 0, 0, 0), (-73.48585690000007, 45.74654788000009, 0, 0, 0), (-73.47091550000007, 45.74654788000009, 3, 1, 2), (-73.45597410000008, 45.74654788000009, 16, 6, 10), (-73.44103270000008, 45.74654788000009, 3, 2, 1), (-73.42609130000008, 45.74654788000009, 0, 0, 0), (-73.41114990000008, 45.74654788000009, 0, 0, 0), (-73.39620850000009, 45.74654788000009, 0, 0, 0), (-73.38126710000009, 45.74654788000009, 0, 0, 0), (-73.36632570000009, 45.74654788000009, 0, 0, 0), (-73.35138430000009, 45.74654788000009, 0, 0, 0), (-73.3364429000001, 45.74654788000009, 0, 0, 0), (-73.3215015000001, 45.74654788000009, 0, 0, 0), (-73.3065601000001, 45.74654788000009, 0, 0, 0), (-73.2916187000001, 45.74654788000009, 0, 0, 0), (-74.0237473, 45.75673660000009, 55, 4, 51), (-74.0088059, 45.75673660000009, 19, 0, 19), (-73.9938645, 45.75673660000009, 1, 1, 0), (-73.9789231, 45.75673660000009, 0, 0, 0), (-73.9639817, 45.75673660000009, 0, 0, 0), (-73.94904030000001, 45.75673660000009, 0, 0, 0), (-73.93409890000001, 45.75673660000009, 0, 0, 0), (-73.91915750000001, 45.75673660000009, 5, 2, 3), (-73.90421610000001, 45.75673660000009,

0, 0, 0), (-73.88927470000002, 45.75673660000009, 0, 0, 0), (-73.87433330000002, 45.75673660000009, 0, 0, 0), (-73.85939190000002, 45.75673660000009, 0, 0, 0), (-73.84445050000002, 45.75673660000009, 0, 0, 0), (-73.82950910000002, 45.75673660000009, 0, 0, 0), (-73.81456770000003, 45.75673660000009, 0, 0, 0), (-73.79962630000003, 45.75673660000009, 0, 0, 0), (-73.78468490000003, 45.75673660000009, 1, 1, 0), (-73.76974350000003, 45.75673660000009, 0, 0, 0), (-73.75480210000003, 45.75673660000009, 0, 0, 0), (-73.73986070000004, 45.75673660000009, 0, 0, 0), (-73.72491930000004, 45.75673660000009, 0, 0, 0), (-73.70997790000004, 45.75673660000009, 0, 0, 0), (-73.69503650000004, 45.75673660000009, 0, 0, 0), (-73.68009510000005, 45.75673660000009, 0, 0, 0), (-73.66515370000005, 45.75673660000009, 0, 0, 0), (-73.65021230000005, 45.75673660000009, 0, 0, 0), (-73.63527090000005, 45.75673660000009, 0, 0, 0), (-73.62032950000005, 45.75673660000009, 0, 0, 0), (-73.60538810000006, 45.75673660000009, 0, 0, 0), (-73.59044670000006, 45.75673660000009, 0, 0, 0), (-73.57550530000006, 45.75673660000009, 0, 0, 0), (-73.56056390000006, 45.75673660000009, 0, 0, 0), (-73.54562250000006, 45.75673660000009, 0, 0, 0), (-73.53068110000007, 45.75673660000009, 0, 0, 0), (-73.51573970000007, 45.75673660000009, 1, 1, 0), (-73.50079830000007, 45.75673660000009, 1, 1, 0), (-73.48585690000007, 45.75673660000009, 3, 0, 3), (-73.47091550000007, 45.75673660000009, 20, 2, 18), (-73.45597410000008, 45.75673660000009, 0, 0, 0), (-73.44103270000008, 45.75673660000009, 148, 29, 119), (-73.42609130000008, 45.75673660000009, 0, 0, 0), (-73.41114990000008, 45.75673660000009, 0, 0, 0), (-73.39620850000009, 45.75673660000009, 0, 0, 0), (-73.38126710000009, 45.75673660000009, 0, 0, 0), (-73.36632570000009, 45.75673660000009, 0, 0, 0), (-73.35138430000009, 45.75673660000009, 0, 0, 0), (-73.3364429000001, 45.75673660000009, 0, 0, 0), (-73.3215015000001, 45.75673660000009, 0, 0, 0), (-73.3065601000001, 45.75673660000009, 0, 0, 0), (-73.2916187000001, 45.75673660000009, 0, 0, 0), (-74.0237473, 45.76692532000009, 2, 1, 1), (-74.0088059, 45.76692532000009, 2, 0, 2), (-73.9938645, 45.76692532000009, 19, 17, 2), (-73.9789231, 45.76692532000009, 9, 2, 7), (-73.9639817, 45.76692532000009, 0, 0, 0), (-73.94904030000001, 45.76692532000009, 0, 0, 0), (-73.93409890000001, 45.76692532000009, 0, 0, 0), (-73.91915750000001, 45.76692532000009, 0, 0, 0), (-73.90421610000001, 45.76692532000009, 0, 0, 0), (-73.88927470000002, 45.76692532000009, 0, 0, 0), (-73.87433330000002, 45.76692532000009, 0, 0, 0), (-73.85939190000002, 45.76692532000009, 0, 0, 0), (-73.84445050000002, 45.76692532000009, 0, 0, 0), (-73.82950910000002, 45.76692532000009, 0, 0, 0), (-73.81456770000003, 45.76692532000009, 2, 1, 1), (-73.79962630000003, 45.76692532000009, 1, 0, 1), (-73.78468490000003, 45.76692532000009, 0, 0, 0), (-73.76974350000003, 45.76692532000009, 0, 0, 0), (-73.75480210000003, 45.76692532000009, 0, 0, 0), (-73.73986070000004, 45.76692532000009, 1, 1, 0), (-73.72491930000004, 45.76692532000009, 0, 0, 0), (-73.70997790000004, 45.76692532000009, 0, 0, 0), (-73.69503650000004, 45.76692532000009, 0, 0, 0), (-73.68009510000005, 45.76692532000009, 0, 0, 0), (-73.66515370000005, 45.76692532000009, 1, 0, 1), (-73.65021230000005, 45.76692532000009, 0, 0, 0), (-73.63527090000005, 45.76692532000009, 0, 0, 0), (-73.62032950000005, 45.76692532000009, 0, 0, 0), (-73.60538810000006, 45.76692532000009, 0, 0, 0), (-73.59044670000006, 45.76692532000009, 0, 0, 0), (-73.57550530000006, 45.76692532000009, 0, 0, 0), (-73.56056390000006, 45.76692532000009, 0, 0, 0), (-73.54562250000006, 45.76692532000009, 0, 0, 0), (-73.53068110000007, 45.76692532000009, 0, 0, 0), (-73.51573970000007, 45.76692532000009, 0, 0, 0), (-73.50079830000007, 45.76692532000009, 0, 0, 0), (-73.48585690000007, 45.76692532000009, 2, 2, 0), (-73.47091550000007, 45.76692532000009, 3, 3, 0), (-73.45597410000008, 45.76692532000009, 0, 0, 0), (-73.44103270000008, 45.76692532000009, 1, 1, 0), (-73.42609130000008, 45.76692532000009, 1, 1, 0), (-73.41114990000008, 45.76692532000009, 0, 0, 0), (-73.39620850000009, 45.76692532000009, 0, 0, 0), (-73.38126710000009, 45.76692532000009, 0, 0, 0), (-73.36632570000009,

45.76692532000009, 2, 2, 0), (-73.35138430000009, 45.76692532000009, 196, 193, 3), (-73.3364429000001, 45.76692532000009, 1, 1, 0), (-73.3215015000001, 45.76692532000009, 0, 0, 0), (-73.3065601000001, 45.76692532000009, 0, 0, 0), (-73.2916187000001, 45.76692532000009, 0, 0, 0), (-74.0237473, 45.77711404000009, 2, 0, 2), (-74.0088059, 45.77711404000009, 253, 119, 134), (-73.9938645, 45.77711404000009, 44, 36, 8), (-73.9789231, 45.77711404000009, 1, 1, 0), (-73.9639817, 45.77711404000009, 0, 0, 0), (-73.94904030000001, 45.77711404000009, 0, 0, 0), (-73.93409890000001, 45.77711404000009, 0, 0, 0), (-73.91915750000001, 45.77711404000009, 0, 0, 0), (-73.90421610000001, 45.77711404000009, 0, 0, 0), (-73.88927470000002, 45.77711404000009, 0, 0, 0), (-73.87433330000002, 45.77711404000009, 0, 0, 0), (-73.85939190000002, 45.77711404000009, 0, 0, 0), (-73.84445050000002, 45.77711404000009, 0, 0, 0), (-73.82950910000002, 45.77711404000009, 0, 0, 0), (-73.81456770000003, 45.77711404000009, 0, 0, 0), (-73.79962630000003, 45.77711404000009, 0, 0, 0), (-73.78468490000003, 45.77711404000009, 0, 0, 0), (-73.76974350000003, 45.77711404000009, 0, 0, 0), (-73.75480210000003, 45.77711404000009, 0, 0, 0), (-73.73986070000004, 45.77711404000009, 2, 2, 0), (-73.72491930000004, 45.77711404000009, 0, 0, 0), (-73.70997790000004, 45.77711404000009, 0, 0, 0), (-73.69503650000004, 45.77711404000009, 0, 0, 0), (-73.68009510000005, 45.77711404000009, 0, 0, 0), (-73.66515370000005, 45.77711404000009, 0, 0, 0), (-73.65021230000005, 45.77711404000009, 0, 0, 0), (-73.63527090000005, 45.77711404000009, 71, 15, 56), (-73.62032950000005, 45.77711404000009, 0, 0, 0), (-73.60538810000006, 45.77711404000009, 0, 0, 0), (-73.59044670000006, 45.77711404000009, 0, 0, 0), (-73.57550530000006, 45.77711404000009, 0, 0, 0), (-73.56056390000006, 45.77711404000009, 0, 0, 0), (-73.54562250000006, 45.77711404000009, 0, 0, 0), (-73.53068110000007, 45.77711404000009, 0, 0, 0), (-73.51573970000007, 45.77711404000009, 0, 0, 0), (-73.50079830000007, 45.77711404000009, 0, 0, 0), (-73.48585690000007, 45.77711404000009, 0, 0, 0), (-73.47091550000007, 45.77711404000009, 0, 0, 0), (-73.45597410000008, 45.77711404000009, 0, 0, 0), (-73.44103270000008, 45.77711404000009, 0, 0, 0), (-73.42609130000008, 45.77711404000009, 0, 0, 0), (-73.41114990000008, 45.77711404000009, 0, 0, 0), (-73.39620850000009, 45.77711404000009, 0, 0, 0), (-73.38126710000009, 45.77711404000009, 0, 0, 0), (-73.36632570000009, 45.77711404000009, 795, 763, 32), (-73.35138430000009, 45.77711404000009, 6, 4, 2), (-73.3364429000001, 45.77711404000009, 0, 0, 0), (-73.3215015000001, 45.77711404000009, 0, 0, 0), (-73.3065601000001, 45.77711404000009, 0, 0, 0), (-73.2916187000001, 45.77711404000009, 0, 0, 0), (-74.0237473, 45.787302760000095, 0, 0, 0), (-74.0088059, 45.787302760000095, 0, 0, 0), (-73.9938645, 45.787302760000095, 7, 2, 5), (-73.9789231, 45.787302760000095, 1, 0, 1), (-73.9639817, 45.787302760000095, 0, 0, 0), (-73.94904030000001, 45.787302760000095, 0, 0, 0), (-73.93409890000001, 45.787302760000095, 2, 1, 1), (-73.91915750000001, 45.787302760000095, 0, 0, 0), (-73.90421610000001, 45.787302760000095, 0, 0, 0), (-73.88927470000002, 45.787302760000095, 0, 0, 0), (-73.87433330000002, 45.787302760000095, 0, 0, 0), (-73.85939190000002, 45.787302760000095, 0, 0, 0), (-73.84445050000002, 45.787302760000095, 0, 0, 0), (-73.82950910000002, 45.787302760000095, 0, 0, 0), (-73.81456770000003, 45.787302760000095, 0, 0, 0), (-73.79962630000003, 45.787302760000095, 0, 0, 0), (-73.78468490000003, 45.787302760000095, 0, 0, 0), (-73.76974350000003, 45.787302760000095, 0, 0, 0), (-73.75480210000003, 45.787302760000095, 0, 0, 0), (-73.73986070000004, 45.787302760000095, 0, 0, 0), (-73.72491930000004, 45.787302760000095, 0, 0, 0), (-73.70997790000004, 45.787302760000095, 0, 0, 0), (-73.69503650000004, 45.787302760000095, 0, 0, 0), (-73.68009510000005, 45.787302760000095, 0, 0, 0), (-73.66515370000005, 45.787302760000095, 0, 0, 0), (-73.65021230000005, 45.787302760000095, 0, 0, 0), (-73.63527090000005, 45.787302760000095, 0, 0, 0), (-73.62032950000005, 45.787302760000095, 0, 0, 0), (-73.60538810000006, 45.787302760000095, 0, 0, 0),

(-73.59044670000006, 45.787302760000095, 0, 0, 0), (-73.57550530000006, 45.787302760000095, 0, 0, 0), (-73.56056390000006, 45.787302760000095, 0, 0, 0), (-73.54562250000006, 45.787302760000095, 0, 0, 0), (-73.53068110000007, 45.787302760000095, 0, 0, 0), (-73.51573970000007, 45.787302760000095, 0, 0, 0), (-73.50079830000007, 45.787302760000095, 2, 2, 0), (-73.48585690000007, 45.787302760000095, 0, 0, 0), (-73.47091550000007, 45.787302760000095, 0, 0, 0), (-73.45597410000008, 45.787302760000095, 0, 0, 0), (-73.44103270000008, 45.787302760000095, 0, 0, 0), (-73.42609130000008, 45.787302760000095, 0, 0, 0), (-73.41114990000008, 45.787302760000095, 1, 1, 0), (-73.39620850000009, 45.787302760000095, 0, 0, 0), (-73.38126710000009, 45.787302760000095, 0, 0, 0), (-73.36632570000009, 45.787302760000095, 0, 0, 0), (-73.35138430000009, 45.787302760000095, 0, 0, 0), (-73.3364429000001, 45.787302760000095, 0, 0, 0), (-73.3215015000001, 45.787302760000095, 0, 0, 0), (-73.3065601000001, 45.787302760000095, 0, 0, 0), (-73.2916187000001, 45.787302760000095, 0, 0, 0), (-74.0237473, 45.7974914800001, 15, 6, 9), (-74.0088059, 45.7974914800001, 0, 0, 0), (-73.9938645, 45.7974914800001, 1, 1, 0), (-73.9789231, 45.7974914800001, 0, 0, 0), (-73.9639817, 45.7974914800001, 0, 0, 0), (-73.94904030000001, 45.7974914800001, 0, 0, 0), (-73.93409890000001, 45.7974914800001, 0, 0, 0), (-73.91915750000001, 45.7974914800001, 0, 0, 0), (-73.90421610000001, 45.7974914800001, 0, 0, 0), (-73.88927470000002, 45.7974914800001, 0, 0, 0), (-73.87433330000002, 45.7974914800001, 0, 0, 0), (-73.85939190000002, 45.7974914800001, 0, 0, 0), (-73.84445050000002, 45.7974914800001, 0, 0, 0), (-73.82950910000002, 45.7974914800001, 0, 0, 0), (-73.81456770000003, 45.7974914800001, 0, 0, 0), (-73.79962630000003, 45.7974914800001, 0, 0, 0), (-73.78468490000003, 45.7974914800001, 0, 0, 0), (-73.76974350000003, 45.7974914800001, 0, 0, 0), (-73.75480210000003, 45.7974914800001, 0, 0, 0), (-73.73986070000004, 45.7974914800001, 0, 0, 0), (-73.72491930000004, 45.7974914800001, 0, 0, 0), (-73.70997790000004, 45.7974914800001, 0, 0, 0), (-73.69503650000004, 45.7974914800001, 0, 0, 0), (-73.68009510000005, 45.7974914800001, 0, 0, 0), (-73.66515370000005, 45.7974914800001, 0, 0, 0), (-73.65021230000005, 45.7974914800001, 0, 0, 0), (-73.63527090000005, 45.7974914800001, 0, 0, 0), (-73.62032950000005, 45.7974914800001, 0, 0, 0), (-73.60538810000006, 45.7974914800001, 0, 0, 0), (-73.59044670000006, 45.7974914800001, 0, 0, 0), (-73.57550530000006, 45.7974914800001, 0, 0, 0), (-73.56056390000006, 45.7974914800001, 0, 0, 0), (-73.54562250000006, 45.7974914800001, 0, 0, 0), (-73.53068110000007, 45.7974914800001, 0, 0, 0), (-73.51573970000007, 45.7974914800001, 0, 0, 0), (-73.50079830000007, 45.7974914800001, 0, 0, 0), (-73.48585690000007, 45.7974914800001, 0, 0, 0), (-73.47091550000007, 45.7974914800001, 0, 0, 0), (-73.45597410000008, 45.7974914800001, 1, 1, 0), (-73.44103270000008, 45.7974914800001, 0, 0, 0), (-73.42609130000008, 45.7974914800001, 0, 0, 0), (-73.41114990000008, 45.7974914800001, 1, 1, 0), (-73.39620850000009, 45.7974914800001, 0, 0, 0), (-73.38126710000009, 45.7974914800001, 0, 0, 0), (-73.36632570000009, 45.7974914800001, 0, 0, 0), (-73.35138430000009, 45.7974914800001, 0, 0, 0), (-73.3364429000001, 45.7974914800001, 1, 0, 1), (-73.3215015000001, 45.7974914800001, 0, 0, 0), (-73.3065601000001, 45.7974914800001, 0, 0, 0), (-73.2916187000001, 45.7974914800001, 0, 0, 0), (-74.0237473, 45.8076802000001, 3, 1, 2), (-74.0088059, 45.8076802000001, 0, 0, 0), (-73.9938645, 45.8076802000001, 0, 0, 0), (-73.9789231, 45.8076802000001, 0, 0, 0), (-73.9639817, 45.8076802000001, 0, 0, 0), (-73.94904030000001, 45.8076802000001, 0, 0, 0), (-73.93409890000001, 45.8076802000001, 0, 0, 0), (-73.91915750000001, 45.8076802000001, 0, 0, 0), (-73.90421610000001, 45.8076802000001, 0, 0, 0), (-73.88927470000002, 45.8076802000001, 0, 0, 0), (-73.87433330000002, 45.8076802000001, 0, 0, 0), (-73.85939190000002, 45.8076802000001, 0, 0, 0), (-73.84445050000002, 45.8076802000001, 0, 0, 0), (-73.82950910000002, 45.8076802000001, 0, 0, 0), (-73.81456770000003, 45.8076802000001, 0, 0, 0),

(-73.79962630000003, 45.8076802000001, 0, 0, 0), (-73.78468490000003, 45.8076802000001, 0, 0, 0), (-73.76974350000003, 45.8076802000001, 0, 0, 0), (-73.75480210000003, 45.8076802000001, 0, 0, 0), (-73.73986070000004, 45.8076802000001, 0, 0, 0), (-73.72491930000004, 45.8076802000001, 0, 0, 0), (-73.70997790000004, 45.8076802000001, 0, 0, 0), (-73.69503650000004, 45.8076802000001, 0, 0, 0), (-73.68009510000005, 45.8076802000001, 0, 0, 0), (-73.66515370000005, 45.8076802000001, 0, 0, 0), (-73.65021230000005, 45.8076802000001, 0, 0, 0), (-73.63527090000005, 45.8076802000001, 0, 0, 0), (-73.62032950000005, 45.8076802000001, 0, 0, 0), (-73.60538810000006, 45.8076802000001, 0, 0, 0), (-73.59044670000006, 45.8076802000001, 0, 0, 0), (-73.57550530000006, 45.8076802000001, 0, 0, 0), (-73.56056390000006, 45.8076802000001, 0, 0, 0), (-73.54562250000006, 45.8076802000001, 0, 0, 0), (-73.53068110000007, 45.8076802000001, 0, 0, 0), (-73.51573970000007, 45.8076802000001, 0, 0, 0), (-73.50079830000007, 45.8076802000001, 0, 0, 0), (-73.48585690000007, 45.8076802000001, 0, 0, 0), (-73.47091550000007, 45.8076802000001, 0, 0, 0), (-73.45597410000008, 45.8076802000001, 0, 0, 0), (-73.44103270000008, 45.8076802000001, 0, 0, 0), (-73.42609130000008, 45.8076802000001, 0, 0, 0), (-73.41114990000008, 45.8076802000001, 0, 0, 0), (-73.39620850000009, 45.8076802000001, 0, 0, 0), (-73.38126710000009, 45.8076802000001, 0, 0, 0), (-73.36632570000009, 45.8076802000001, 0, 0, 0), (-73.35138430000009, 45.8076802000001, 0, 0, 0), (-73.3364429000001, 45.8076802000001, 0, 0, 0), (-73.3215015000001, 45.8076802000001, 0, 0, 0), (-73.3065601000001, 45.8076802000001, 0, 0, 0), (-73.2916187000001, 45.8076802000001, 0, 0, 0), (-74.0237473, 45.8178689200001, 0, 0, 0), (-74.0088059, 45.8178689200001, 0, 0, 0), (-73.9938645, 45.8178689200001, 0, 0, 0), (-73.9789231, 45.8178689200001, 0, 0, 0), (-73.9639817, 45.8178689200001, 0, 0, 0), (-73.94904030000001, 45.8178689200001, 0, 0, 0), (-73.93409890000001, 45.8178689200001, 560, 11, 549), (-73.91915750000001, 45.8178689200001, 0, 0, 0), (-73.90421610000001, 45.8178689200001, 6, 2, 4), (-73.88927470000002, 45.8178689200001, 0, 0, 0), (-73.87433330000002, 45.8178689200001, 0, 0, 0), (-73.85939190000002, 45.8178689200001, 0, 0, 0), (-73.84445050000002, 45.8178689200001, 0, 0, 0), (-73.82950910000002, 45.8178689200001, 0, 0, 0), (-73.81456770000003, 45.8178689200001, 0, 0, 0), (-73.79962630000003, 45.8178689200001, 0, 0, 0), (-73.78468490000003, 45.8178689200001, 0, 0, 0), (-73.76974350000003, 45.8178689200001, 0, 0, 0), (-73.75480210000003, 45.8178689200001, 0, 0, 0), (-73.73986070000004, 45.8178689200001, 0, 0, 0), (-73.72491930000004, 45.8178689200001, 0, 0, 0), (-73.70997790000004, 45.8178689200001, 0, 0, 0), (-73.69503650000004, 45.8178689200001, 0, 0, 0), (-73.68009510000005, 45.8178689200001, 0, 0, 0), (-73.66515370000005, 45.8178689200001, 0, 0, 0), (-73.65021230000005, 45.8178689200001, 0, 0, 0), (-73.63527090000005, 45.8178689200001, 0, 0, 0), (-73.62032950000005, 45.8178689200001, 0, 0, 0), (-73.60538810000006, 45.8178689200001, 0, 0, 0), (-73.59044670000006, 45.8178689200001, 0, 0, 0), (-73.57550530000006, 45.8178689200001, 0, 0, 0), (-73.56056390000006, 45.8178689200001, 0, 0, 0), (-73.54562250000006, 45.8178689200001, 0, 0, 0), (-73.53068110000007, 45.8178689200001, 0, 0, 0), (-73.51573970000007, 45.8178689200001, 0, 0, 0), (-73.50079830000007, 45.8178689200001, 0, 0, 0), (-73.48585690000007, 45.8178689200001, 0, 0, 0), (-73.47091550000007, 45.8178689200001, 0, 0, 0), (-73.45597410000008, 45.8178689200001, 0, 0, 0), (-73.44103270000008, 45.8178689200001, 2, 2, 0), (-73.42609130000008, 45.8178689200001, 2, 2, 0), (-73.41114990000008, 45.8178689200001, 0, 0, 0), (-73.39620850000009, 45.8178689200001, 0, 0, 0), (-73.38126710000009, 45.8178689200001, 0, 0, 0), (-73.36632570000009, 45.8178689200001, 0, 0, 0), (-73.35138430000009, 45.8178689200001, 0, 0, 0), (-73.3364429000001, 45.8178689200001, 0, 0, 0), (-73.3215015000001, 45.8178689200001, 0, 0, 0), (-73.3065601000001, 45.8178689200001, 0, 0, 0), (-73.2916187000001, 45.8178689200001, 0, 0, 0), (-74.0237473, 45.828057640000104, 0, 0, 0), (-74.0088059, 45.828057640000104, 0, 0, 0), (-73.9938645, 45.828057640000104, 0, 0, 0),

(-73.9789231, 45.828057640000104, 0, 0, 0), (-73.9639817, 45.828057640000104, 0, 0, 0), (-73.94904030000001, 45.828057640000104, 0, 0, 0), (-73.93409890000001, 45.828057640000104, 0, 0, 0), (-73.91915750000001, 45.828057640000104, 0, 0, 0), (-73.90421610000001, 45.828057640000104, 0, 0, 0), (-73.88927470000002, 45.828057640000104, 0, 0, 0), (-73.87433330000002, 45.828057640000104, 0, 0, 0), (-73.85939190000002, 45.828057640000104, 0, 0, 0), (-73.84445050000002, 45.828057640000104, 0, 0, 0), (-73.82950910000002, 45.828057640000104, 0, 0, 0), (-73.81456770000003, 45.828057640000104, 0, 0, 0), (-73.79962630000003, 45.828057640000104, 0, 0, 0), (-73.78468490000003, 45.828057640000104, 0, 0, 0), (-73.76974350000003, 45.828057640000104, 0, 0, 0), (-73.75480210000003, 45.828057640000104, 0, 0, 0), (-73.73986070000004, 45.828057640000104, 0, 0, 0), (-73.72491930000004, 45.828057640000104, 0, 0, 0), (-73.70997790000004, 45.828057640000104, 0, 0, 0), (-73.69503650000004, 45.828057640000104, 0, 0, 0), (-73.68009510000005, 45.828057640000104, 0, 0, 0), (-73.66515370000005, 45.828057640000104, 0, 0, 0), (-73.65021230000005, 45.828057640000104, 0, 0, 0), (-73.63527090000005, 45.828057640000104, 0, 0, 0), (-73.62032950000005, 45.828057640000104, 0, 0, 0), (-73.60538810000006, 45.828057640000104, 1, 0, 1), (-73.59044670000006, 45.828057640000104, 0, 0, 0), (-73.57550530000006, 45.828057640000104, 0, 0, 0), (-73.56056390000006, 45.828057640000104, 0, 0, 0), (-73.54562250000006, 45.828057640000104, 0, 0, 0), (-73.53068110000007, 45.828057640000104, 0, 0, 0), (-73.51573970000007, 45.828057640000104, 0, 0, 0), (-73.50079830000007, 45.828057640000104, 0, 0, 0), (-73.48585690000007, 45.828057640000104, 0, 0, 0), (-73.47091550000007, 45.828057640000104, 0, 0, 0), (-73.45597410000008, 45.828057640000104, 0, 0, 0), (-73.44103270000008, 45.828057640000104, 6, 5, 1), (-73.42609130000008, 45.828057640000104, 12, 12, 0), (-73.41114990000008, 45.828057640000104, 0, 0, 0), (-73.39620850000009, 45.828057640000104, 0, 0, 0), (-73.38126710000009, 45.828057640000104, 0, 0, 0), (-73.36632570000009, 45.828057640000104, 1, 0, 1), (-73.35138430000009, 45.828057640000104, 1, 1, 0), (-73.3364429000001, 45.828057640000104, 0, 0, 0), (-73.3215015000001, 45.828057640000104, 0, 0, 0), (-73.3065601000001, 45.828057640000104, 0, 0, 0), (-73.2916187000001, 45.828057640000104, 0, 0, 0)]
