# Supplementary material for: Probing sociodemographic influence on code-switching and language choice in Quebec with geolocation of tweets
Source: Front Psychol. 2023 May 2;14:1137038. doi: 10.3389/fpsyg.2023.1137038 (PMC10187760; doi:10.3389/fpsyg.2023.1137038)
Supplement: Supplementary file 3 [file Data_Sheet_3.PDF]

List of tuples: (coordinates, -CS-Engl., +CS-Engl.)
[(-74.0237473, 45.32881036, 0, 0), (-74.0088059, 45.32881036, 0, 0), (-73.9938645, 45.32881036, 0, 0), (-73.9789231, 45.32881036, 0, 0), (-73.9639817, 45.32881036, 0, 1), (-73.94904030000001, 45.32881036, 0, 0), (-73.93409890000001, 45.32881036, 0, 0), (-73.91915750000001, 45.32881036, 0, 0), (-73.90421610000001, 45.32881036, 0, 0), (-73.88927470000002, 45.32881036, 0, 0), (-73.87433330000002, 45.32881036, 0, 0), (-73.85939190000002, 45.32881036, 0, 0), (-73.84445050000002, 45.32881036, 0, 0), (-73.82950910000002, 45.32881036, 0, 0), (-73.81456770000003, 45.32881036, 0, 0), (-73.79962630000003, 45.32881036, 0, 0), (-73.78468490000003, 45.32881036, 0, 0), (-73.76974350000003, 45.32881036, 0, 0), (-73.75480210000003, 45.32881036, 0, 0), (-73.73986070000004, 45.32881036, 0, 0), (-73.72491930000004, 45.32881036, 0, 0), (-73.70997790000004, 45.32881036, 0, 0), (-73.69503650000004, 45.32881036, 0, 0), (-73.68009510000005, 45.32881036, 0, 0), (-73.66515370000005, 45.32881036, 0, 0), (-73.65021230000005, 45.32881036, 0, 0), (-73.63527090000005, 45.32881036, 0, 0), (-73.62032950000005, 45.32881036, 0, 0), (-73.60538810000006, 45.32881036, 0, 0), (-73.59044670000006, 45.32881036, 0, 0), (-73.57550530000006, 45.32881036, 0, 0), (-73.56056390000006, 45.32881036, 0, 0), (-73.54562250000006, 45.32881036, 0, 0), (-73.53068110000007, 45.32881036, 0, 0), (-73.51573970000007, 45.32881036, 0, 0), (-73.50079830000007, 45.32881036, 0, 0), (-73.48585690000007, 45.32881036, 0, 0), (-73.47091550000007, 45.32881036, 0, 0), (-73.45597410000008, 45.32881036, 0, 0), (-73.44103270000008, 45.32881036, 0, 0), (-73.42609130000008, 45.32881036, 0, 0), (-73.41114990000008, 45.32881036, 0, 0), (-73.39620850000009, 45.32881036, 0, 0), (-73.38126710000009, 45.32881036, 0, 0), (-73.36632570000009, 45.32881036, 0, 0), (-73.35138430000009, 45.32881036, 0, 0), (-73.3364429000001, 45.32881036, 0, 0), (-73.3215015000001, 45.32881036, 0, 0), (-73.3065601000001, 45.32881036, 0, 0), (-73.2916187000001, 45.32881036, 0, 0), (-74.0237473, 45.33899908, 0, 0), (-74.0088059, 45.33899908, 0, 0), (-73.9938645, 45.33899908, 0, 0), (-73.9789231, 45.33899908, 0, 0), (-73.9639817, 45.33899908, 0, 0), (-73.94904030000001, 45.33899908, 0, 0), (-73.93409890000001, 45.33899908, 0, 0), (-73.91915750000001, 45.33899908, 0, 0), (-73.90421610000001, 45.33899908, 0, 0), (-73.88927470000002, 45.33899908, 0, 0), (-73.87433330000002, 45.33899908, 0, 0), (-73.85939190000002, 45.33899908, 0, 0), (-73.84445050000002, 45.33899908, 0, 0), (-73.82950910000002, 45.33899908, 0, 0), (-73.81456770000003, 45.33899908, 0, 0), (-73.79962630000003, 45.33899908, 0, 0), (-73.78468490000003, 45.33899908, 0, 0), (-73.76974350000003, 45.33899908, 0, 0), (-73.75480210000003, 45.33899908, 0, 0), (-73.73986070000004, 45.33899908, 0, 0), (-73.72491930000004, 45.33899908, 0, 1), (-73.70997790000004, 45.33899908, 0, 0), (-73.69503650000004, 45.33899908, 0, 0), (-73.68009510000005, 45.33899908, 0, 0), (-73.66515370000005, 45.33899908, 0, 0), (-73.65021230000005, 45.33899908, 0, 0), (-73.63527090000005, 45.33899908, 0, 0), (-73.62032950000005, 45.33899908, 0, 0), (-73.60538810000006, 45.33899908, 0, 0), (-73.59044670000006, 45.33899908, 0, 0), (-73.57550530000006, 45.33899908, 0, 0), (-73.56056390000006, 45.33899908, 0, 0), (-73.54562250000006, 45.33899908, 0, 0), (-73.53068110000007, 45.33899908, 0, 0), (-73.51573970000007, 45.33899908, 0, 0), (-73.50079830000007, 45.33899908, 0, 0), (-73.48585690000007, 45.33899908, 0, 0), (-73.47091550000007, 45.33899908, 0, 0), (-73.45597410000008, 45.33899908, 0, 0), (-73.44103270000008, 45.33899908, 0, 0), (-73.42609130000008, 45.33899908, 0, 0), (-73.41114990000008, 45.33899908, 0, 0), (-73.39620850000009, 45.33899908, 0, 0), (-73.38126710000009, 45.33899908, 0, 0), (-73.36632570000009, 45.33899908, 0, 0), (-73.35138430000009, 45.33899908, 0, 0), (-73.3364429000001, 45.33899908, 0, 0), (-73.3215015000001, 45.33899908, 0, 0), (-73.3065601000001, 45.33899908, 0, 0), (-73.2916187000001, 45.33899908, 0, 0), (-74.0237473, 45.3491878, 0, 0), (-74.0088059, 45.3491878, 0, 0), (-73.9938645, 45.3491878, 0, 0), (-73.9789231, 45.3491878, 0, 0), (-73.9639817, 45.3491878, 0, 0), (-73.94904030000001,

45.3491878, 0, 0), (-73.93409890000001, 45.3491878, 0, 0), (-73.91915750000001, 45.3491878, 0, 0), (-73.90421610000001, 45.3491878, 2, 2), (-73.88927470000002, 45.3491878, 0, 0), (-73.87433330000002, 45.3491878, 0, 0), (-73.85939190000002, 45.3491878, 0, 0), (-73.84445050000002, 45.3491878, 0, 0), (-73.82950910000002, 45.3491878, 0, 0), (-73.81456770000003, 45.3491878, 0, 0), (-73.79962630000003, 45.3491878, 1, 0), (-73.78468490000003, 45.3491878, 0, 0), (-73.76974350000003, 45.3491878, 0, 2), (-73.75480210000003, 45.3491878, 0, 0), (-73.73986070000004, 45.3491878, 0, 0), (-73.72491930000004, 45.3491878, 0, 0), (-73.70997790000004, 45.3491878, 0, 0), (-73.69503650000004, 45.3491878, 2, 2), (-73.68009510000005, 45.3491878, 0, 0), (-73.66515370000005, 45.3491878, 0, 0), (-73.65021230000005, 45.3491878, 0, 0), (-73.63527090000005, 45.3491878, 0, 0), (-73.62032950000005, 45.3491878, 0, 0), (-73.60538810000006, 45.3491878, 0, 0), (-73.59044670000006, 45.3491878, 0, 0), (-73.57550530000006, 45.3491878, 0, 0), (-73.56056390000006, 45.3491878, 0, 0), (-73.54562250000006, 45.3491878, 0, 0), (-73.53068110000007, 45.3491878, 0, 0), (-73.51573970000007, 45.3491878, 0, 0), (-73.50079830000007, 45.3491878, 0, 0), (-73.48585690000007, 45.3491878, 0, 0), (-73.47091550000007, 45.3491878, 4, 5), (-73.45597410000008, 45.3491878, 0, 0), (-73.44103270000008, 45.3491878, 0, 0), (-73.42609130000008, 45.3491878, 0, 0), (-73.41114990000008, 45.3491878, 0, 0), (-73.39620850000009, 45.3491878, 0, 0), (-73.38126710000009, 45.3491878, 0, 0), (-73.36632570000009, 45.3491878, 0, 0), (-73.35138430000009, 45.3491878, 0, 0), (-73.3364429000001, 45.3491878, 0, 0), (-73.3215015000001, 45.3491878, 0, 0), (-73.3065601000001, 45.3491878, 2, 0), (-73.2916187000001, 45.3491878, 0, 0), (-74.0237473, 45.359376520000005, 0, 0), (-74.0088059, 45.359376520000005, 0, 0), (-73.9938645, 45.359376520000005, 0, 0), (-73.9789231, 45.359376520000005, 0, 0), (-73.9639817, 45.359376520000005, 0, 0), (-73.94904030000001, 45.359376520000005, 0, 0), (-73.93409890000001, 45.359376520000005, 1, 0), (-73.91915750000001, 45.359376520000005, 2, 0), (-73.90421610000001, 45.359376520000005, 0, 0), (-73.88927470000002, 45.359376520000005, 0, 0), (-73.87433330000002, 45.359376520000005, 0, 0), (-73.85939190000002, 45.359376520000005, 0, 0), (-73.84445050000002, 45.359376520000005, 0, 0), (-73.82950910000002, 45.359376520000005, 0, 0), (-73.81456770000003, 45.359376520000005, 3, 0), (-73.79962630000003, 45.359376520000005, 0, 0), (-73.78468490000003, 45.359376520000005, 0, 0), (-73.76974350000003, 45.359376520000005, 0, 0), (-73.75480210000003, 45.359376520000005, 3, 11), (-73.73986070000004, 45.359376520000005, 6, 4), (-73.72491930000004, 45.359376520000005, 2, 1), (-73.70997790000004, 45.359376520000005, 0, 0), (-73.69503650000004, 45.359376520000005, 0, 0), (-73.68009510000005, 45.359376520000005, 0, 0), (-73.66515370000005, 45.359376520000005, 0, 0), (-73.65021230000005, 45.359376520000005, 0, 0), (-73.63527090000005, 45.359376520000005, 0, 0), (-73.62032950000005, 45.359376520000005, 0, 0), (-73.60538810000006, 45.359376520000005, 0, 0), (-73.59044670000006, 45.359376520000005, 0, 0), (-73.57550530000006, 45.359376520000005, 0, 0), (-73.56056390000006, 45.359376520000005, 0, 0), (-73.54562250000006, 45.359376520000005, 0, 0), (-73.53068110000007, 45.359376520000005, 0, 0), (-73.51573970000007, 45.359376520000005, 0, 0), (-73.50079830000007, 45.359376520000005, 0, 0), (-73.48585690000007, 45.359376520000005, 0, 0), (-73.47091550000007, 45.359376520000005, 0, 0), (-73.45597410000008, 45.359376520000005, 0, 0), (-73.44103270000008, 45.359376520000005, 0, 0), (-73.42609130000008, 45.359376520000005, 0, 0), (-73.41114990000008, 45.359376520000005, 0, 0), (-73.39620850000009, 45.359376520000005, 0, 0), (-73.38126710000009, 45.359376520000005, 0, 0), (-73.36632570000009, 45.359376520000005, 0, 0), (-73.35138430000009, 45.359376520000005, 0, 0), (-73.3364429000001, 45.359376520000005, 0, 0), (-73.3215015000001, 45.359376520000005, 0, 0), (-73.3065601000001, 45.359376520000005, 0, 1), (-73.2916187000001, 45.359376520000005,

1, 0), (-74.0237473, 45.36956524000001, 0, 0), (-74.0088059, 45.36956524000001, 0, 0), (-73.9938645, 45.36956524000001, 0, 0), (-73.9789231, 45.36956524000001, 3, 4), (-73.9639817, 45.36956524000001, 0, 0), (-73.94904030000001, 45.36956524000001, 0, 0), (-73.93409890000001, 45.36956524000001, 0, 9), (-73.91915750000001, 45.36956524000001, 0, 0), (-73.90421610000001, 45.36956524000001, 1, 0), (-73.88927470000002, 45.36956524000001, 0, 0), (-73.87433330000002, 45.36956524000001, 0, 2), (-73.85939190000002, 45.36956524000001, 0, 0), (-73.84445050000002, 45.36956524000001, 0, 0), (-73.82950910000002, 45.36956524000001, 0, 0), (-73.81456770000003, 45.36956524000001, 0, 0), (-73.79962630000003, 45.36956524000001, 0, 0), (-73.78468490000003, 45.36956524000001, 0, 0), (-73.76974350000003, 45.36956524000001, 0, 0), (-73.75480210000003, 45.36956524000001, 0, 0), (-73.73986070000004, 45.36956524000001, 0, 0), (-73.72491930000004, 45.36956524000001, 0, 0), (-73.70997790000004, 45.36956524000001, 0, 0), (-73.69503650000004, 45.36956524000001, 0, 0), (-73.68009510000005, 45.36956524000001, 0, 0), (-73.66515370000005, 45.36956524000001, 0, 0), (-73.65021230000005, 45.36956524000001, 0, 0), (-73.63527090000005, 45.36956524000001, 0, 0), (-73.62032950000005, 45.36956524000001, 0, 0), (-73.60538810000006, 45.36956524000001, 0, 0), (-73.59044670000006, 45.36956524000001, 0, 0), (-73.57550530000006, 45.36956524000001, 12, 3), (-73.56056390000006, 45.36956524000001, 13, 9), (-73.54562250000006, 45.36956524000001, 0, 0), (-73.53068110000007, 45.36956524000001, 0, 0), (-73.51573970000007, 45.36956524000001, 0, 0), (-73.50079830000007, 45.36956524000001, 0, 0), (-73.48585690000007, 45.36956524000001, 0, 0), (-73.47091550000007, 45.36956524000001, 0, 0), (-73.45597410000008, 45.36956524000001, 0, 0), (-73.44103270000008, 45.36956524000001, 0, 0), (-73.42609130000008, 45.36956524000001, 0, 0), (-73.41114990000008, 45.36956524000001, 0, 0), (-73.39620850000009, 45.36956524000001, 0, 0), (-73.38126710000009, 45.36956524000001, 0, 0), (-73.36632570000009, 45.36956524000001, 0, 0), (-73.35138430000009, 45.36956524000001, 0, 0), (-73.3364429000001, 45.36956524000001, 0, 0), (-73.3215015000001, 45.36956524000001, 0, 0), (-73.3065601000001, 45.36956524000001, 0, 0), (-73.2916187000001, 45.36956524000001, 0, 0), (-74.0237473, 45.37975396000001, 3, 7), (-74.0088059, 45.37975396000001, 1, 0), (-73.9938645, 45.37975396000001, 0, 1), (-73.9789231, 45.37975396000001, 1, 1), (-73.9639817, 45.37975396000001, 0, 0), (-73.94904030000001, 45.37975396000001, 0, 0), (-73.93409890000001, 45.37975396000001, 0, 0), (-73.91915750000001, 45.37975396000001, 0, 0), (-73.90421610000001, 45.37975396000001, 0, 0), (-73.88927470000002, 45.37975396000001, 0, 0), (-73.87433330000002, 45.37975396000001, 0, 0), (-73.85939190000002, 45.37975396000001, 0, 0), (-73.84445050000002, 45.37975396000001, 0, 0), (-73.82950910000002, 45.37975396000001, 0, 0), (-73.81456770000003, 45.37975396000001, 0, 0), (-73.79962630000003, 45.37975396000001, 0, 0), (-73.78468490000003, 45.37975396000001, 0, 0), (-73.76974350000003, 45.37975396000001, 0, 1), (-73.75480210000003, 45.37975396000001, 4, 6), (-73.73986070000004, 45.37975396000001, 0, 0), (-73.72491930000004, 45.37975396000001, 0, 0), (-73.70997790000004, 45.37975396000001, 2, 2), (-73.69503650000004, 45.37975396000001, 0, 0), (-73.68009510000005, 45.37975396000001, 0, 0), (-73.66515370000005, 45.37975396000001, 0, 0), (-73.65021230000005, 45.37975396000001, 0, 0), (-73.63527090000005, 45.37975396000001, 0, 0), (-73.62032950000005, 45.37975396000001, 0, 0), (-73.60538810000006, 45.37975396000001, 0, 0), (-73.59044670000006, 45.37975396000001, 0, 0), (-73.57550530000006, 45.37975396000001, 0, 0), (-73.56056390000006, 45.37975396000001, 4, 9), (-73.54562250000006, 45.37975396000001, 1, 1), (-73.53068110000007, 45.37975396000001, 1, 0), (-73.51573970000007, 45.37975396000001, 1, 11), (-73.50079830000007, 45.37975396000001, 0, 2), (-73.48585690000007, 45.37975396000001, 0, 0), (-73.47091550000007, 45.37975396000001, 0, 0), (-73.45597410000008, 45.37975396000001, 0, 0), (-73.44103270000008, 45.37975396000001, 0, 0), (-73.42609130000008, 45.37975396000001, 0, 0),

(-73.41114990000008, 45.37975396000001, 0, 0), (-73.39620850000009, 45.37975396000001, 0, 0), (-73.38126710000009, 45.37975396000001, 0, 0), (-73.36632570000009, 45.37975396000001, 0, 0), (-73.35138430000009, 45.37975396000001, 0, 0), (-73.3364429000001, 45.37975396000001, 0, 0), (-73.3215015000001, 45.37975396000001, 0, 0), (-73.3065601000001, 45.37975396000001, 0, 0), (-73.2916187000001, 45.37975396000001, 0, 0), (-74.0237473, 45.38994268000001, 4, 2), (-74.0088059, 45.38994268000001, 0, 1), (-73.9938645, 45.38994268000001, 0, 0), (-73.9789231, 45.38994268000001, 0, 4), (-73.9639817, 45.38994268000001, 1, 1), (-73.94904030000001, 45.38994268000001, 0, 0), (-73.93409890000001, 45.38994268000001, 0, 0), (-73.91915750000001, 45.38994268000001, 0, 0), (-73.90421610000001, 45.38994268000001, 2, 5), (-73.88927470000002, 45.38994268000001, 0, 0), (-73.87433330000002, 45.38994268000001, 0, 0), (-73.85939190000002, 45.38994268000001, 0, 0), (-73.84445050000002, 45.38994268000001, 0, 0), (-73.82950910000002, 45.38994268000001, 0, 0), (-73.81456770000003, 45.38994268000001, 0, 0), (-73.79962630000003, 45.38994268000001, 0, 0), (-73.78468490000003, 45.38994268000001, 0, 0), (-73.76974350000003, 45.38994268000001, 5, 0), (-73.75480210000003, 45.38994268000001, 7, 2), (-73.73986070000004, 45.38994268000001, 0, 0), (-73.72491930000004, 45.38994268000001, 0, 0), (-73.70997790000004, 45.38994268000001, 0, 0), (-73.69503650000004, 45.38994268000001, 0, 0), (-73.68009510000005, 45.38994268000001, 0, 0), (-73.66515370000005, 45.38994268000001, 0, 0), (-73.65021230000005, 45.38994268000001, 0, 0), (-73.63527090000005, 45.38994268000001, 0, 0), (-73.62032950000005, 45.38994268000001, 0, 0), (-73.60538810000006, 45.38994268000001, 0, 0), (-73.59044670000006, 45.38994268000001, 0, 0), (-73.57550530000006, 45.38994268000001, 0, 0), (-73.56056390000006, 45.38994268000001, 0, 0), (-73.54562250000006, 45.38994268000001, 0, 0), (-73.53068110000007, 45.38994268000001, 0, 0), (-73.51573970000007, 45.38994268000001, 0, 0), (-73.50079830000007, 45.38994268000001, 1, 0), (-73.48585690000007, 45.38994268000001, 0, 2), (-73.47091550000007, 45.38994268000001, 1, 0), (-73.45597410000008, 45.38994268000001, 0, 0), (-73.44103270000008, 45.38994268000001, 0, 0), (-73.42609130000008, 45.38994268000001, 0, 0), (-73.41114990000008, 45.38994268000001, 0, 0), (-73.39620850000009, 45.38994268000001, 0, 0), (-73.38126710000009, 45.38994268000001, 0, 0), (-73.36632570000009, 45.38994268000001, 0, 0), (-73.35138430000009, 45.38994268000001, 0, 0), (-73.3364429000001, 45.38994268000001, 0, 0), (-73.3215015000001, 45.38994268000001, 0, 0), (-73.3065601000001, 45.38994268000001, 0, 0), (-73.2916187000001, 45.38994268000001, 0, 0), (-74.0237473, 45.400131400000014, 0, 0), (-74.0088059, 45.400131400000014, 0, 0), (-73.9938645, 45.400131400000014, 0, 0), (-73.9789231, 45.400131400000014, 0, 0), (-73.9639817, 45.400131400000014, 4, 6), (-73.94904030000001, 45.400131400000014, 7, 15), (-73.93409890000001, 45.400131400000014, 0, 1), (-73.91915750000001, 45.400131400000014, 0, 0), (-73.90421610000001, 45.400131400000014, 0, 0), (-73.88927470000002, 45.400131400000014, 0, 0), (-73.87433330000002, 45.400131400000014, 0, 0), (-73.85939190000002, 45.400131400000014, 0, 0), (-73.84445050000002, 45.400131400000014, 0, 0), (-73.82950910000002, 45.400131400000014, 0, 0), (-73.81456770000003, 45.400131400000014, 0, 0), (-73.79962630000003, 45.400131400000014, 0, 0), (-73.78468490000003, 45.400131400000014, 0, 0), (-73.76974350000003, 45.400131400000014, 0, 0), (-73.75480210000003, 45.400131400000014, 0, 0), (-73.73986070000004, 45.400131400000014, 0, 0), (-73.72491930000004, 45.400131400000014, 0, 0), (-73.70997790000004, 45.400131400000014, 0, 0), (-73.69503650000004, 45.400131400000014, 0, 0), (-73.68009510000005, 45.400131400000014, 0, 0), (-73.66515370000005, 45.400131400000014, 0, 0), (-73.65021230000005, 45.400131400000014, 0, 0), (-73.63527090000005, 45.400131400000014, 0, 0), (-73.62032950000005, 45.400131400000014, 0, 0), (-73.60538810000006, 45.400131400000014, 0, 0), (-73.59044670000006, 45.400131400000014, 2, 3), (-73.57550530000006, 45.400131400000014, 1, 0),

(-73.56056390000006, 45.400131400000014, 0, 3), (-73.54562250000006, 45.400131400000014, 4, 0), (-73.53068110000007, 45.400131400000014, 0, 1), (-73.51573970000007, 45.400131400000014, 2, 42), (-73.50079830000007, 45.400131400000014, 2, 2), (-73.48585690000007, 45.400131400000014, 0, 0), (-73.47091550000007, 45.400131400000014, 1, 2), (-73.45597410000008, 45.400131400000014, 0, 0), (-73.44103270000008, 45.400131400000014, 0, 0), (-73.42609130000008, 45.400131400000014, 0, 0), (-73.41114990000008, 45.400131400000014, 0, 0), (-73.39620850000009, 45.400131400000014, 0, 0), (-73.38126710000009, 45.400131400000014, 0, 0), (-73.36632570000009, 45.400131400000014, 0, 0), (-73.35138430000009, 45.400131400000014, 0, 0), (-73.3364429000001, 45.400131400000014, 0, 0), (-73.3215015000001, 45.400131400000014, 0, 0), (-73.3065601000001, 45.400131400000014, 0, 0), (-73.2916187000001, 45.400131400000014, 0, 0), (-74.0237473, 45.410320120000016, 0, 0), (-74.0088059, 45.410320120000016, 0, 1), (-73.9938645, 45.410320120000016, 0, 0), (-73.9789231, 45.410320120000016, 0, 0), (-73.9639817, 45.410320120000016, 2, 0), (-73.94904030000001, 45.410320120000016, 2, 0), (-73.93409890000001, 45.410320120000016, 0, 0), (-73.91915750000001, 45.410320120000016, 0, 0), (-73.90421610000001, 45.410320120000016, 0, 0), (-73.88927470000002, 45.410320120000016, 0, 0), (-73.87433330000002, 45.410320120000016, 0, 0), (-73.85939190000002, 45.410320120000016, 0, 0), (-73.84445050000002, 45.410320120000016, 0, 0), (-73.82950910000002, 45.410320120000016, 0, 0), (-73.81456770000003, 45.410320120000016, 0, 0), (-73.79962630000003, 45.410320120000016, 0, 0), (-73.78468490000003, 45.410320120000016, 0, 0), (-73.76974350000003, 45.410320120000016, 0, 0), (-73.75480210000003, 45.410320120000016, 0, 0), (-73.73986070000004, 45.410320120000016, 0, 0), (-73.72491930000004, 45.410320120000016, 0, 0), (-73.70997790000004, 45.410320120000016, 0, 0), (-73.69503650000004, 45.410320120000016, 0, 0), (-73.68009510000005, 45.410320120000016, 2, 1), (-73.66515370000005, 45.410320120000016, 0, 0), (-73.65021230000005, 45.410320120000016, 0, 0), (-73.63527090000005, 45.410320120000016, 0, 0), (-73.62032950000005, 45.410320120000016, 0, 0), (-73.60538810000006, 45.410320120000016, 0, 0), (-73.59044670000006, 45.410320120000016, 0, 0), (-73.57550530000006, 45.410320120000016, 0, 2), (-73.56056390000006, 45.410320120000016, 0, 1), (-73.54562250000006, 45.410320120000016, 0, 0), (-73.53068110000007, 45.410320120000016, 0, 0), (-73.51573970000007, 45.410320120000016, 0, 0), (-73.50079830000007, 45.410320120000016, 1, 0), (-73.48585690000007, 45.410320120000016, 0, 0), (-73.47091550000007, 45.410320120000016, 1, 1), (-73.45597410000008, 45.410320120000016, 0, 0), (-73.44103270000008, 45.410320120000016, 0, 0), (-73.42609130000008, 45.410320120000016, 0, 0), (-73.41114990000008, 45.410320120000016, 0, 0), (-73.39620850000009, 45.410320120000016, 0, 0), (-73.38126710000009, 45.410320120000016, 0, 0), (-73.36632570000009, 45.410320120000016, 0, 0), (-73.35138430000009, 45.410320120000016, 0, 0), (-73.3364429000001, 45.410320120000016, 0, 0), (-73.3215015000001, 45.410320120000016, 0, 0), (-73.3065601000001, 45.410320120000016, 0, 0), (-73.2916187000001, 45.410320120000016, 0, 0), (-74.0237473, 45.42050884000002, 3, 0), (-74.0088059, 45.42050884000002, 2, 6), (-73.9938645, 45.42050884000002, 0, 0), (-73.9789231, 45.42050884000002, 0, 0), (-73.9639817, 45.42050884000002, 0, 0), (-73.94904030000001, 45.42050884000002, 0, 0), (-73.93409890000001, 45.42050884000002, 0, 0), (-73.91915750000001, 45.42050884000002, 5, 2), (-73.90421610000001, 45.42050884000002, 1, 0), (-73.88927470000002, 45.42050884000002, 0, 0), (-73.87433330000002, 45.42050884000002, 2, 5), (-73.85939190000002, 45.42050884000002, 1, 1), (-73.84445050000002, 45.42050884000002, 0, 0), (-73.82950910000002, 45.42050884000002, 0, 0), (-73.81456770000003, 45.42050884000002, 0, 0), (-73.79962630000003, 45.42050884000002, 0, 0), (-73.78468490000003, 45.42050884000002, 0, 0), (-73.76974350000003, 45.42050884000002, 0, 0), (-73.75480210000003, 45.42050884000002, 0, 0), (-73.73986070000004, 45.42050884000002, 0, 0),

(-73.72491930000004, 45.42050884000002, 0, 0), (-73.70997790000004, 45.42050884000002, 0, 0), (-73.69503650000004, 45.42050884000002, 0, 0), (-73.68009510000005, 45.42050884000002, 0, 0), (-73.66515370000005, 45.42050884000002, 0, 0), (-73.65021230000005, 45.42050884000002, 10, 20), (-73.63527090000005, 45.42050884000002, 1, 1), (-73.62032950000005, 45.42050884000002, 2, 1), (-73.60538810000006, 45.42050884000002, 2, 0), (-73.59044670000006, 45.42050884000002, 0, 0), (-73.57550530000006, 45.42050884000002, 0, 0), (-73.56056390000006, 45.42050884000002, 0, 0), (-73.54562250000006, 45.42050884000002, 0, 0), (-73.53068110000007, 45.42050884000002, 0, 0), (-73.51573970000007, 45.42050884000002, 0, 0), (-73.50079830000007, 45.42050884000002, 2, 3), (-73.48585690000007, 45.42050884000002, 5, 25), (-73.47091550000007, 45.42050884000002, 1, 2), (-73.45597410000008, 45.42050884000002, 0, 0), (-73.44103270000008, 45.42050884000002, 0, 0), (-73.42609130000008, 45.42050884000002, 0, 0), (-73.41114990000008, 45.42050884000002, 0, 0), (-73.39620850000009, 45.42050884000002, 0, 0), (-73.38126710000009, 45.42050884000002, 0, 0), (-73.36632570000009, 45.42050884000002, 0, 0), (-73.35138430000009, 45.42050884000002, 0, 0), (-73.3364429000001, 45.42050884000002, 0, 0), (-73.3215015000001, 45.42050884000002, 0, 0), (-73.3065601000001, 45.42050884000002, 0, 0), (-73.2916187000001, 45.42050884000002, 0, 0), (-74.0237473, 45.43069756000002, 0, 0), (-74.0088059, 45.43069756000002, 0, 0), (-73.9938645, 45.43069756000002, 0, 0), (-73.9789231, 45.43069756000002, 0, 0), (-73.9639817, 45.43069756000002, 0, 0), (-73.94904030000001, 45.43069756000002, 0, 0), (-73.93409890000001, 45.43069756000002, 0, 0), (-73.91915750000001, 45.43069756000002, 0, 0), (-73.90421610000001, 45.43069756000002, 0, 0), (-73.88927470000002, 45.43069756000002, 0, 0), (-73.87433330000002, 45.43069756000002, 0, 125), (-73.85939190000002, 45.43069756000002, 0, 0), (-73.84445050000002, 45.43069756000002, 0, 0), (-73.82950910000002, 45.43069756000002, 2, 3), (-73.81456770000003, 45.43069756000002, 1, 1), (-73.79962630000003, 45.43069756000002, 0, 0), (-73.78468490000003, 45.43069756000002, 0, 0), (-73.76974350000003, 45.43069756000002, 0, 0), (-73.75480210000003, 45.43069756000002, 1, 1), (-73.73986070000004, 45.43069756000002, 0, 0), (-73.72491930000004, 45.43069756000002, 0, 0), (-73.70997790000004, 45.43069756000002, 0, 0), (-73.69503650000004, 45.43069756000002, 5, 2), (-73.68009510000005, 45.43069756000002, 30, 20), (-73.66515370000005, 45.43069756000002, 10, 8), (-73.65021230000005, 45.43069756000002, 1, 10), (-73.63527090000005, 45.43069756000002, 9, 11), (-73.62032950000005, 45.43069756000002, 86, 26), (-73.60538810000006, 45.43069756000002, 3, 2), (-73.59044670000006, 45.43069756000002, 7, 2), (-73.57550530000006, 45.43069756000002, 0, 0), (-73.56056390000006, 45.43069756000002, 0, 0), (-73.54562250000006, 45.43069756000002, 0, 0), (-73.53068110000007, 45.43069756000002, 0, 0), (-73.51573970000007, 45.43069756000002, 0, 0), (-73.50079830000007, 45.43069756000002, 0, 0), (-73.48585690000007, 45.43069756000002, 2, 0), (-73.47091550000007, 45.43069756000002, 1, 1), (-73.45597410000008, 45.43069756000002, 0, 0), (-73.44103270000008, 45.43069756000002, 0, 1), (-73.42609130000008, 45.43069756000002, 0, 0), (-73.41114990000008, 45.43069756000002, 0, 0), (-73.39620850000009, 45.43069756000002, 0, 0), (-73.38126710000009, 45.43069756000002, 0, 0), (-73.36632570000009, 45.43069756000002, 0, 0), (-73.35138430000009, 45.43069756000002, 0, 0), (-73.3364429000001, 45.43069756000002, 0, 0), (-73.3215015000001, 45.43069756000002, 0, 0), (-73.3065601000001, 45.43069756000002, 1, 0), (-73.2916187000001, 45.43069756000002, 7, 1), (-74.0237473, 45.44088628000002, 0, 0), (-74.0088059, 45.44088628000002, 0, 0), (-73.9938645, 45.44088628000002, 0, 0), (-73.9789231, 45.44088628000002, 0, 0), (-73.9639817, 45.44088628000002, 0, 0), (-73.94904030000001, 45.44088628000002, 0, 0), (-73.93409890000001, 45.44088628000002, 0, 0), (-73.91915750000001, 45.44088628000002, 1, 1), (-73.90421610000001, 45.44088628000002, 0, 0), (-73.88927470000002, 45.44088628000002, 0, 3), (-73.87433330000002, 45.44088628000002, 0, 0), (-73.85939190000002,

45.44088628000002, 0, 0), (-73.84445050000002, 45.44088628000002, 0, 0), (-73.82950910000002, 45.44088628000002, 1, 0), (-73.81456770000003, 45.44088628000002, 16, 14), (-73.79962630000003, 45.44088628000002, 0, 1), (-73.78468490000003, 45.44088628000002, 0, 0), (-73.76974350000003, 45.44088628000002, 0, 0), (-73.75480210000003, 45.44088628000002, 5, 2), (-73.73986070000004, 45.44088628000002, 1, 0), (-73.72491930000004, 45.44088628000002, 9, 9), (-73.70997790000004, 45.44088628000002, 3, 1), (-73.69503650000004, 45.44088628000002, 34, 90), (-73.68009510000005, 45.44088628000002, 10, 7), (-73.66515370000005, 45.44088628000002, 7, 4), (-73.65021230000005, 45.44088628000002, 0, 3), (-73.63527090000005, 45.44088628000002, 1, 3), (-73.62032950000005, 45.44088628000002, 0, 2), (-73.60538810000006, 45.44088628000002, 14, 6), (-73.59044670000006, 45.44088628000002, 0, 0), (-73.57550530000006, 45.44088628000002, 2, 0), (-73.56056390000006, 45.44088628000002, 0, 2), (-73.54562250000006, 45.44088628000002, 0, 0), (-73.53068110000007, 45.44088628000002, 0, 0), (-73.51573970000007, 45.44088628000002, 2, 1), (-73.50079830000007, 45.44088628000002, 0, 1), (-73.48585690000007, 45.44088628000002, 0, 0), (-73.47091550000007, 45.44088628000002, 15, 6), (-73.45597410000008, 45.44088628000002, 0, 0), (-73.44103270000008, 45.44088628000002, 35, 17), (-73.42609130000008, 45.44088628000002, 4, 1), (-73.41114990000008, 45.44088628000002, 0, 0), (-73.39620850000009, 45.44088628000002, 0, 0), (-73.38126710000009, 45.44088628000002, 0, 0), (-73.36632570000009, 45.44088628000002, 0, 0), (-73.35138430000009, 45.44088628000002, 0, 0), (-73.3364429000001, 45.44088628000002, 0, 0), (-73.3215015000001, 45.44088628000002, 0, 0), (-73.3065601000001, 45.44088628000002, 1, 0), (-73.2916187000001, 45.44088628000002, 1, 7), (-74.0237473, 45.451075000000024, 0, 0), (-74.0088059, 45.451075000000024, 0, 0), (-73.9938645, 45.451075000000024, 0, 0), (-73.9789231, 45.451075000000024, 0, 0), (-73.9639817, 45.451075000000024, 0, 0), (-73.94904030000001, 45.451075000000024, 0, 0), (-73.93409890000001, 45.451075000000024, 0, 1), (-73.91915750000001, 45.451075000000024, 0, 0), (-73.90421610000001, 45.451075000000024, 0, 0), (-73.88927470000002, 45.451075000000024, 0, 0), (-73.87433330000002, 45.451075000000024, 0, 0), (-73.85939190000002, 45.451075000000024, 3, 8), (-73.84445050000002, 45.451075000000024, 0, 0), (-73.82950910000002, 45.451075000000024, 0, 0), (-73.81456770000003, 45.451075000000024, 6, 13), (-73.79962630000003, 45.451075000000024, 3, 2), (-73.78468490000003, 45.451075000000024, 11, 8), (-73.76974350000003, 45.451075000000024, 0, 0), (-73.75480210000003, 45.451075000000024, 4, 22), (-73.73986070000004, 45.451075000000024, 0, 1), (-73.72491930000004, 45.451075000000024, 0, 0), (-73.70997790000004, 45.451075000000024, 0, 0), (-73.69503650000004, 45.451075000000024, 3, 2), (-73.68009510000005, 45.451075000000024, 0, 0), (-73.66515370000005, 45.451075000000024, 0, 0), (-73.65021230000005, 45.451075000000024, 10, 73), (-73.63527090000005, 45.451075000000024, 1, 9), (-73.62032950000005, 45.451075000000024, 1, 2), (-73.60538810000006, 45.451075000000024, 13, 16), (-73.59044670000006, 45.451075000000024, 24, 58), (-73.57550530000006, 45.451075000000024, 78, 51), (-73.56056390000006, 45.451075000000024, 63, 39), (-73.54562250000006, 45.451075000000024, 1, 1), (-73.53068110000007, 45.451075000000024, 0, 0), (-73.51573970000007, 45.451075000000024, 0, 0), (-73.50079830000007, 45.451075000000024, 1, 0), (-73.48585690000007, 45.451075000000024, 7, 8), (-73.47091550000007, 45.451075000000024, 37, 22), (-73.45597410000008, 45.451075000000024, 29, 71), (-73.44103270000008, 45.451075000000024, 62, 29), (-73.42609130000008, 45.451075000000024, 1, 0), (-73.41114990000008, 45.451075000000024, 0, 0), (-73.39620850000009, 45.451075000000024, 0, 0), (-73.38126710000009, 45.451075000000024, 0, 0), (-73.36632570000009, 45.451075000000024, 0, 0), (-73.35138430000009, 45.451075000000024, 0, 1), (-73.3364429000001, 45.451075000000024, 1, 1), (-73.3215015000001, 45.451075000000024, 0, 0), (-73.3065601000001, 45.451075000000024, 5,

15), (-73.2916187000001, 45.451075000000024, 1, 2), (-74.0237473, 45.46126372000003, 0, 0), (-74.0088059, 45.46126372000003, 0, 0), (-73.9938645, 45.46126372000003, 0, 0), (-73.9789231, 45.46126372000003, 0, 0), (-73.9639817, 45.46126372000003, 0, 0), (-73.94904030000001, 45.46126372000003, 2, 0), (-73.93409890000001, 45.46126372000003, 2, 0), (-73.91915750000001, 45.46126372000003, 0, 0), (-73.90421610000001, 45.46126372000003, 0, 0), (-73.88927470000002, 45.46126372000003, 0, 1), (-73.87433330000002, 45.46126372000003, 0, 1), (-73.85939190000002, 45.46126372000003, 1, 1), (-73.84445050000002, 45.46126372000003, 0, 0), (-73.82950910000002, 45.46126372000003, 4, 16), (-73.81456770000003, 45.46126372000003, 0, 0), (-73.79962630000003, 45.46126372000003, 0, 0), (-73.78468490000003, 45.46126372000003, 0, 0), (-73.76974350000003, 45.46126372000003, 0, 0), (-73.75480210000003, 45.46126372000003, 73, 154), (-73.73986070000004, 45.46126372000003, 13, 22), (-73.72491930000004, 45.46126372000003, 1, 1), (-73.70997790000004, 45.46126372000003, 0, 0), (-73.69503650000004, 45.46126372000003, 0, 0), (-73.68009510000005, 45.46126372000003, 0, 0), (-73.66515370000005, 45.46126372000003, 0, 1), (-73.65021230000005, 45.46126372000003, 0, 0), (-73.63527090000005, 45.46126372000003, 8, 6), (-73.62032950000005, 45.46126372000003, 7, 5), (-73.60538810000006, 45.46126372000003, 7, 0), (-73.59044670000006, 45.46126372000003, 30, 16), (-73.57550530000006, 45.46126372000003, 4, 12), (-73.56056390000006, 45.46126372000003, 88, 37), (-73.54562250000006, 45.46126372000003, 20, 20), (-73.53068110000007, 45.46126372000003, 0, 0), (-73.51573970000007, 45.46126372000003, 0, 0), (-73.50079830000007, 45.46126372000003, 0, 2), (-73.48585690000007, 45.46126372000003, 7, 2), (-73.47091550000007, 45.46126372000003, 4, 1), (-73.45597410000008, 45.46126372000003, 0, 0), (-73.44103270000008, 45.46126372000003, 0, 0), (-73.42609130000008, 45.46126372000003, 0, 0), (-73.41114990000008, 45.46126372000003, 0, 0), (-73.39620850000009, 45.46126372000003, 0, 0), (-73.38126710000009, 45.46126372000003, 0, 0), (-73.36632570000009, 45.46126372000003, 0, 0), (-73.35138430000009, 45.46126372000003, 2, 5), (-73.3364429000001, 45.46126372000003, 1, 1), (-73.3215015000001, 45.46126372000003, 0, 1), (-73.3065601000001, 45.46126372000003, 0, 0), (-73.2916187000001, 45.46126372000003, 0, 0), (-74.0237473, 45.47145244000003, 0, 0), (-74.0088059, 45.47145244000003, 0, 0), (-73.9938645, 45.47145244000003, 0, 0), (-73.9789231, 45.47145244000003, 0, 0), (-73.9639817, 45.47145244000003, 0, 0), (-73.94904030000001, 45.47145244000003, 0, 0), (-73.93409890000001, 45.47145244000003, 2, 0), (-73.91915750000001, 45.47145244000003, 0, 1), (-73.90421610000001, 45.47145244000003, 0, 0), (-73.88927470000002, 45.47145244000003, 8, 53), (-73.87433330000002, 45.47145244000003, 1, 0), (-73.85939190000002, 45.47145244000003, 0, 0), (-73.84445050000002, 45.47145244000003, 1, 0), (-73.82950910000002, 45.47145244000003, 8, 51), (-73.81456770000003, 45.47145244000003, 0, 0), (-73.79962630000003, 45.47145244000003, 12, 65), (-73.78468490000003, 45.47145244000003, 0, 42), (-73.76974350000003, 45.47145244000003, 1, 0), (-73.75480210000003, 45.47145244000003, 0, 0), (-73.73986070000004, 45.47145244000003, 0, 0), (-73.72491930000004, 45.47145244000003, 0, 0), (-73.70997790000004, 45.47145244000003, 0, 0), (-73.69503650000004, 45.47145244000003, 1, 0), (-73.68009510000005, 45.47145244000003, 0, 3), (-73.66515370000005, 45.47145244000003, 4, 329), (-73.65021230000005, 45.47145244000003, 50, 18), (-73.63527090000005, 45.47145244000003, 4, 3), (-73.62032950000005, 45.47145244000003, 53, 66), (-73.60538810000006, 45.47145244000003, 53, 46), (-73.59044670000006, 45.47145244000003, 60, 58), (-73.57550530000006, 45.47145244000003, 40, 27), (-73.56056390000006, 45.47145244000003, 22, 2), (-73.54562250000006, 45.47145244000003, 4, 2), (-73.53068110000007, 45.47145244000003, 0, 0), (-73.51573970000007, 45.47145244000003, 3, 1), (-73.50079830000007, 45.47145244000003, 0, 1), (-73.48585690000007, 45.47145244000003, 0, 0), (-73.47091550000007, 45.47145244000003, 5, 2), (-73.45597410000008, 45.47145244000003, 0, 0), (-73.44103270000008,

45.47145244000003, 0, 0), (-73.42609130000008, 45.47145244000003, 0, 0), (-73.41114990000008, 45.47145244000003, 0, 0), (-73.39620850000009, 45.47145244000003, 1, 0), (-73.38126710000009, 45.47145244000003, 0, 0), (-73.36632570000009, 45.47145244000003, 0, 0), (-73.35138430000009, 45.47145244000003, 0, 0), (-73.3364429000001, 45.47145244000003, 0, 0), (-73.3215015000001, 45.47145244000003, 0, 0), (-73.3065601000001, 45.47145244000003, 0, 0), (-73.2916187000001, 45.47145244000003, 0, 0), (-74.0237473, 45.48164116000003, 0, 0), (-74.0088059, 45.48164116000003, 0, 0), (-73.9938645, 45.48164116000003, 0, 0), (-73.9789231, 45.48164116000003, 0, 0), (-73.9639817, 45.48164116000003, 0, 0), (-73.94904030000001, 45.48164116000003, 0, 0), (-73.93409890000001, 45.48164116000003, 0, 0), (-73.91915750000001, 45.48164116000003, 0, 0), (-73.90421610000001, 45.48164116000003, 29, 27), (-73.88927470000002, 45.48164116000003, 0, 0), (-73.87433330000002, 45.48164116000003, 8, 8), (-73.85939190000002, 45.48164116000003, 1, 1), (-73.84445050000002, 45.48164116000003, 1, 2), (-73.82950910000002, 45.48164116000003, 4, 1), (-73.81456770000003, 45.48164116000003, 7, 0), (-73.79962630000003, 45.48164116000003, 26, 48), (-73.78468490000003, 45.48164116000003, 2, 6), (-73.76974350000003, 45.48164116000003, 0, 0), (-73.75480210000003, 45.48164116000003, 0, 1), (-73.73986070000004, 45.48164116000003, 0, 0), (-73.72491930000004, 45.48164116000003, 0, 0), (-73.70997790000004, 45.48164116000003, 0, 0), (-73.69503650000004, 45.48164116000003, 3, 3), (-73.68009510000005, 45.48164116000003, 0, 0), (-73.66515370000005, 45.48164116000003, 0, 0), (-73.65021230000005, 45.48164116000003, 0, 2), (-73.63527090000005, 45.48164116000003, 21, 8), (-73.62032950000005, 45.48164116000003, 49, 64), (-73.60538810000006, 45.48164116000003, 17, 14), (-73.59044670000006, 45.48164116000003, 42, 29), (-73.57550530000006, 45.48164116000003, 118, 119), (-73.56056390000006, 45.48164116000003, 35, 49), (-73.54562250000006, 45.48164116000003, 7, 10), (-73.53068110000007, 45.48164116000003, 0, 0), (-73.51573970000007, 45.48164116000003, 0, 0), (-73.50079830000007, 45.48164116000003, 0, 0), (-73.48585690000007, 45.48164116000003, 1, 7), (-73.47091550000007, 45.48164116000003, 5, 2), (-73.45597410000008, 45.48164116000003, 3, 2), (-73.44103270000008, 45.48164116000003, 1, 0), (-73.42609130000008, 45.48164116000003, 0, 0), (-73.41114990000008, 45.48164116000003, 0, 0), (-73.39620850000009, 45.48164116000003, 1, 1), (-73.38126710000009, 45.48164116000003, 13, 17), (-73.36632570000009, 45.48164116000003, 0, 0), (-73.35138430000009, 45.48164116000003, 0, 0), (-73.3364429000001, 45.48164116000003, 0, 0), (-73.3215015000001, 45.48164116000003, 0, 0), (-73.3065601000001, 45.48164116000003, 0, 0), (-73.2916187000001, 45.48164116000003, 0, 0), (-74.0237473, 45.49182988000003, 2, 2), (-74.0088059, 45.49182988000003, 0, 0), (-73.9938645, 45.49182988000003, 0, 0), (-73.9789231, 45.49182988000003, 0, 0), (-73.9639817, 45.49182988000003, 0, 0), (-73.94904030000001, 45.49182988000003, 0, 0), (-73.93409890000001, 45.49182988000003, 0, 0), (-73.91915750000001, 45.49182988000003, 0, 0), (-73.90421610000001, 45.49182988000003, 0, 0), (-73.88927470000002, 45.49182988000003, 0, 0), (-73.87433330000002, 45.49182988000003, 3, 13), (-73.85939190000002, 45.49182988000003, 0, 0), (-73.84445050000002, 45.49182988000003, 3, 2), (-73.82950910000002, 45.49182988000003, 0, 0), (-73.81456770000003, 45.49182988000003, 42, 197), (-73.79962630000003, 45.49182988000003, 0, 2), (-73.78468490000003, 45.49182988000003, 0, 0), (-73.76974350000003, 45.49182988000003, 0, 0), (-73.75480210000003, 45.49182988000003, 9, 11), (-73.73986070000004, 45.49182988000003, 17, 3), (-73.72491930000004, 45.49182988000003, 0, 0), (-73.70997790000004, 45.49182988000003, 1, 7), (-73.69503650000004, 45.49182988000003, 5, 2), (-73.68009510000005, 45.49182988000003, 1, 2), (-73.66515370000005, 45.49182988000003, 7, 8), (-73.65021230000005, 45.49182988000003, 102, 134), (-73.63527090000005, 45.49182988000003, 5, 3), (-73.62032950000005, 45.49182988000003, 60, 73), (-73.60538810000006, 45.49182988000003, 5, 7), (-73.59044670000006,

45.49182988000003, 32, 33), (-73.57550530000006, 45.49182988000003, 350, 232),
(-73.56056390000006, 45.49182988000003, 112, 58), (-73.54562250000006, 45.49182988000003,
9, 7), (-73.53068110000007, 45.49182988000003, 1, 1), (-73.51573970000007,
45.49182988000003, 1, 0), (-73.50079830000007, 45.49182988000003, 3, 1),
(-73.48585690000007, 45.49182988000003, 0, 0), (-73.47091550000007, 45.49182988000003, 3,
3), (-73.45597410000008, 45.49182988000003, 1, 0), (-73.44103270000008, 45.49182988000003,
2, 2), (-73.42609130000008, 45.49182988000003, 0, 0), (-73.41114990000008,
45.49182988000003, 3, 3), (-73.39620850000009, 45.49182988000003, 2, 0),
(-73.38126710000009, 45.49182988000003, 8, 7), (-73.36632570000009, 45.49182988000003, 0,
0), (-73.35138430000009, 45.49182988000003, 0, 0), (-73.3364429000001, 45.49182988000003,
0, 0), (-73.3215015000001, 45.49182988000003, 0, 0), (-73.3065601000001, 45.49182988000003,
0, 0), (-73.2916187000001, 45.49182988000003, 0, 0), (-74.0237473, 45.502018600000035, 0, 0),
(-74.0088059, 45.502018600000035, 0, 0), (-73.9938645, 45.502018600000035, 0, 0),
(-73.9789231, 45.502018600000035, 0, 0), (-73.9639817, 45.502018600000035, 2, 1),
(-73.94904030000001, 45.502018600000035, 0, 0), (-73.93409890000001, 45.502018600000035,
0, 0), (-73.91915750000001, 45.502018600000035, 0, 0), (-73.90421610000001,
45.502018600000035, 3, 2), (-73.88927470000002, 45.502018600000035, 0, 0),
(-73.87433330000002, 45.502018600000035, 0, 0), (-73.85939190000002, 45.502018600000035,
1, 1), (-73.84445050000002, 45.502018600000035, 2, 0), (-73.82950910000002,
45.502018600000035, 0, 0), (-73.81456770000003, 45.502018600000035, 0, 0),
(-73.79962630000003, 45.502018600000035, 2, 1), (-73.78468490000003, 45.502018600000035,
1, 1), (-73.76974350000003, 45.502018600000035, 1, 1), (-73.75480210000003,
45.502018600000035, 10, 27), (-73.73986070000004, 45.502018600000035, 0, 0),
(-73.72491930000004, 45.502018600000035, 0, 1), (-73.70997790000004, 45.502018600000035,
10, 6), (-73.69503650000004, 45.502018600000035, 1, 0), (-73.68009510000005,
45.502018600000035, 3, 2), (-73.66515370000005, 45.502018600000035, 4, 5),
(-73.65021230000005, 45.502018600000035, 163, 184), (-73.63527090000005,
45.502018600000035, 15, 8), (-73.62032950000005, 45.502018600000035, 82, 41),
(-73.60538810000006, 45.502018600000035, 43, 39), (-73.59044670000006,
45.502018600000035, 661, 310), (-73.57550530000006, 45.502018600000035, 5136, 3079),
(-73.56056390000006, 45.502018600000035, 1321, 3165), (-73.54562250000006,
45.502018600000035, 281, 130), (-73.53068110000007, 45.502018600000035, 68, 74),
(-73.51573970000007, 45.502018600000035, 58, 47), (-73.50079830000007,
45.502018600000035, 8, 1), (-73.48585690000007, 45.502018600000035, 2, 0),
(-73.47091550000007, 45.502018600000035, 0, 1), (-73.45597410000008, 45.502018600000035,
0, 0), (-73.44103270000008, 45.502018600000035, 0, 3), (-73.42609130000008,
45.502018600000035, 3, 1), (-73.41114990000008, 45.502018600000035, 14, 132),
(-73.39620850000009, 45.502018600000035, 0, 0), (-73.38126710000009, 45.502018600000035,
6, 2), (-73.36632570000009, 45.502018600000035, 0, 0), (-73.35138430000009,
45.502018600000035, 0, 0), (-73.3364429000001, 45.502018600000035, 0, 0),
(-73.3215015000001, 45.502018600000035, 0, 0), (-73.3065601000001, 45.502018600000035, 0,
0), (-73.2916187000001, 45.502018600000035, 1, 0), (-74.0237473, 45.51220732000004, 0, 0),
(-74.0088059, 45.51220732000004, 0, 0), (-73.9938645, 45.51220732000004, 1, 0), (-73.9789231,
45.51220732000004, 0, 0), (-73.9639817, 45.51220732000004, 0, 0), (-73.94904030000001,
45.51220732000004, 0, 0), (-73.93409890000001, 45.51220732000004, 0, 0),
(-73.91915750000001, 45.51220732000004, 0, 0), (-73.90421610000001, 45.51220732000004, 0,
0), (-73.88927470000002, 45.51220732000004, 1, 2), (-73.87433330000002, 45.51220732000004,
0, 0), (-73.85939190000002, 45.51220732000004, 0, 0), (-73.84445050000002,
45.51220732000004, 1, 0), (-73.82950910000002, 45.51220732000004, 1, 1),
(-73.81456770000003, 45.51220732000004, 1, 1), (-73.79962630000003, 45.51220732000004, 0,
0), (-73.78468490000003, 45.51220732000004, 0, 0), (-73.76974350000003, 45.51220732000004,

0, 0), (-73.75480210000003, 45.51220732000004, 0, 0), (-73.73986070000004, 45.51220732000004, 0, 0), (-73.72491930000004, 45.51220732000004, 5, 0), (-73.70997790000004, 45.51220732000004, 13, 9), (-73.69503650000004, 45.51220732000004, 6, 1), (-73.68009510000005, 45.51220732000004, 8, 10), (-73.66515370000005, 45.51220732000004, 29, 28), (-73.65021230000005, 45.51220732000004, 27, 33), (-73.63527090000005, 45.51220732000004, 5, 3), (-73.62032950000005, 45.51220732000004, 31, 39), (-73.60538810000006, 45.51220732000004, 23, 23), (-73.59044670000006, 45.51220732000004, 30, 181), (-73.57550530000006, 45.51220732000004, 295, 171), (-73.56056390000006, 45.51220732000004, 1296, 888), (-73.54562250000006, 45.51220732000004, 104, 89), (-73.53068110000007, 45.51220732000004, 43, 57), (-73.51573970000007, 45.51220732000004, 4, 1), (-73.50079830000007, 45.51220732000004, 0, 0), (-73.48585690000007, 45.51220732000004, 2, 1), (-73.47091550000007, 45.51220732000004, 3, 0), (-73.45597410000008, 45.51220732000004, 47, 19), (-73.44103270000008, 45.51220732000004, 2, 0), (-73.42609130000008, 45.51220732000004, 4, 3), (-73.41114990000008, 45.51220732000004, 0, 0), (-73.39620850000009, 45.51220732000004, 1, 0), (-73.38126710000009, 45.51220732000004, 1, 0), (-73.36632570000009, 45.51220732000004, 0, 0), (-73.35138430000009, 45.51220732000004, 0, 0), (-73.3364429000001, 45.51220732000004, 0, 0), (-73.3215015000001, 45.51220732000004, 0, 0), (-73.3065601000001, 45.51220732000004, 0, 0), (-73.2916187000001, 45.51220732000004, 0, 0), (-74.0237473, 45.52239604000004, 0, 0), (-74.0088059, 45.52239604000004, 0, 0), (-73.9938645, 45.52239604000004, 0, 0), (-73.9789231, 45.52239604000004, 0, 0), (-73.9639817, 45.52239604000004, 0, 0), (-73.94904030000001, 45.52239604000004, 0, 0), (-73.93409890000001, 45.52239604000004, 1, 1), (-73.91915750000001, 45.52239604000004, 0, 0), (-73.90421610000001, 45.52239604000004, 0, 0), (-73.88927470000002, 45.52239604000004, 0, 0), (-73.87433330000002, 45.52239604000004, 0, 0), (-73.85939190000002, 45.52239604000004, 1, 1), (-73.84445050000002, 45.52239604000004, 0, 1), (-73.82950910000002, 45.52239604000004, 0, 0), (-73.81456770000003, 45.52239604000004, 0, 0), (-73.79962630000003, 45.52239604000004, 1, 1), (-73.78468490000003, 45.52239604000004, 4, 3), (-73.76974350000003, 45.52239604000004, 0, 0), (-73.75480210000003, 45.52239604000004, 2, 2), (-73.73986070000004, 45.52239604000004, 3, 1), (-73.72491930000004, 45.52239604000004, 4, 4), (-73.70997790000004, 45.52239604000004, 10, 2), (-73.69503650000004, 45.52239604000004, 1, 0), (-73.68009510000005, 45.52239604000004, 1, 4), (-73.66515370000005, 45.52239604000004, 75, 18), (-73.65021230000005, 45.52239604000004, 1, 1), (-73.63527090000005, 45.52239604000004, 5, 1), (-73.62032950000005, 45.52239604000004, 131, 35), (-73.60538810000006, 45.52239604000004, 157, 118), (-73.59044670000006, 45.52239604000004, 505, 325), (-73.57550530000006, 45.52239604000004, 580, 433), (-73.56056390000006, 45.52239604000004, 602, 208), (-73.54562250000006, 45.52239604000004, 247, 74), (-73.53068110000007, 45.52239604000004, 20, 13), (-73.51573970000007, 45.52239604000004, 13, 3), (-73.50079830000007, 45.52239604000004, 8, 1), (-73.48585690000007, 45.52239604000004, 1, 0), (-73.47091550000007, 45.52239604000004, 71, 39), (-73.45597410000008, 45.52239604000004, 2, 1), (-73.44103270000008, 45.52239604000004, 0, 0), (-73.42609130000008, 45.52239604000004, 0, 0), (-73.41114990000008, 45.52239604000004, 0, 0), (-73.39620850000009, 45.52239604000004, 1, 4), (-73.38126710000009, 45.52239604000004, 0, 0), (-73.36632570000009, 45.52239604000004, 1, 1), (-73.35138430000009, 45.52239604000004, 0, 1), (-73.3364429000001, 45.52239604000004, 3, 18), (-73.3215015000001, 45.52239604000004, 0, 0), (-73.3065601000001, 45.52239604000004, 0, 0), (-73.2916187000001, 45.52239604000004, 3, 4), (-74.0237473, 45.53258476000004, 0, 0), (-74.0088059, 45.53258476000004, 2, 0), (-73.9938645, 45.53258476000004, 6, 8), (-73.9789231, 45.53258476000004, 0, 0), (-73.9639817, 45.53258476000004, 0, 0), (-73.94904030000001, 45.53258476000004, 0, 0), (-73.93409890000001, 45.53258476000004, 3, 0),

(-73.91915750000001, 45.53258476000004, 0, 0), (-73.90421610000001, 45.53258476000004, 0, 0), (-73.88927470000002, 45.53258476000004, 2, 0), (-73.87433330000002, 45.53258476000004, 0, 1), (-73.85939190000002, 45.53258476000004, 1, 0), (-73.84445050000002, 45.53258476000004, 0, 0), (-73.82950910000002, 45.53258476000004, 0, 0), (-73.81456770000003, 45.53258476000004, 7, 10), (-73.79962630000003, 45.53258476000004, 1, 1), (-73.78468490000003, 45.53258476000004, 6, 6), (-73.76974350000003, 45.53258476000004, 0, 0), (-73.75480210000003, 45.53258476000004, 2, 3), (-73.73986070000004, 45.53258476000004, 4, 0), (-73.72491930000004, 45.53258476000004, 15, 1), (-73.70997790000004, 45.53258476000004, 26, 7), (-73.69503650000004, 45.53258476000004, 2, 0), (-73.68009510000005, 45.53258476000004, 1, 4), (-73.66515370000005, 45.53258476000004, 28, 12), (-73.65021230000005, 45.53258476000004, 16, 32), (-73.63527090000005, 45.53258476000004, 32, 12), (-73.62032950000005, 45.53258476000004, 344, 321), (-73.60538810000006, 45.53258476000004, 350, 205), (-73.59044670000006, 45.53258476000004, 141, 45), (-73.57550530000006, 45.53258476000004, 114, 49), (-73.56056390000006, 45.53258476000004, 61, 20), (-73.54562250000006, 45.53258476000004, 20, 6), (-73.53068110000007, 45.53258476000004, 0, 0), (-73.51573970000007, 45.53258476000004, 140, 159), (-73.50079830000007, 45.53258476000004, 4, 1), (-73.48585690000007, 45.53258476000004, 18, 9), (-73.47091550000007, 45.53258476000004, 0, 0), (-73.45597410000008, 45.53258476000004, 0, 0), (-73.44103270000008, 45.53258476000004, 0, 1), (-73.42609130000008, 45.53258476000004, 0, 0), (-73.41114990000008, 45.53258476000004, 0, 0), (-73.39620850000009, 45.53258476000004, 0, 0), (-73.38126710000009, 45.53258476000004, 0, 0), (-73.36632570000009, 45.53258476000004, 0, 0), (-73.35138430000009, 45.53258476000004, 12, 15), (-73.3364429000001, 45.53258476000004, 1, 1), (-73.3215015000001, 45.53258476000004, 0, 0), (-73.3065601000001, 45.53258476000004, 1, 0), (-73.2916187000001, 45.53258476000004, 0, 0), (-74.0237473, 45.542773480000044, 1, 2), (-74.0088059, 45.542773480000044, 2, 0), (-73.9938645, 45.542773480000044, 0, 0), (-73.9789231, 45.542773480000044, 0, 0), (-73.9639817, 45.542773480000044, 0, 0), (-73.94904030000001, 45.542773480000044, 0, 0), (-73.93409890000001, 45.542773480000044, 0, 0), (-73.91915750000001, 45.542773480000044, 1, 0), (-73.90421610000001, 45.542773480000044, 0, 0), (-73.88927470000002, 45.542773480000044, 2, 1), (-73.87433330000002, 45.542773480000044, 7, 3), (-73.85939190000002, 45.542773480000044, 3, 0), (-73.84445050000002, 45.542773480000044, 0, 0), (-73.82950910000002, 45.542773480000044, 0, 0), (-73.81456770000003, 45.542773480000044, 0, 0), (-73.79962630000003, 45.542773480000044, 0, 0), (-73.78468490000003, 45.542773480000044, 0, 0), (-73.76974350000003, 45.542773480000044, 4, 1), (-73.75480210000003, 45.542773480000044, 6, 8), (-73.73986070000004, 45.542773480000044, 0, 0), (-73.72491930000004, 45.542773480000044, 4, 1), (-73.70997790000004, 45.542773480000044, 1, 0), (-73.69503650000004, 45.542773480000044, 2, 1), (-73.68009510000005, 45.542773480000044, 6, 0), (-73.66515370000005, 45.542773480000044, 47, 12), (-73.65021230000005, 45.542773480000044, 14, 6), (-73.63527090000005, 45.542773480000044, 156, 79), (-73.62032950000005, 45.542773480000044, 68, 37), (-73.60538810000006, 45.542773480000044, 183, 73), (-73.59044670000006, 45.542773480000044, 101, 59), (-73.57550530000006, 45.542773480000044, 79, 36), (-73.56056390000006, 45.542773480000044, 21, 9), (-73.54562250000006, 45.542773480000044, 166, 63), (-73.53068110000007, 45.542773480000044, 21, 9), (-73.51573970000007, 45.542773480000044, 4, 2), (-73.50079830000007, 45.542773480000044, 5, 2), (-73.48585690000007, 45.542773480000044, 3, 2), (-73.47091550000007, 45.542773480000044, 8, 3), (-73.45597410000008, 45.542773480000044, 0, 1), (-73.44103270000008, 45.542773480000044, 0, 0), (-73.42609130000008, 45.542773480000044, 1, 1), (-73.41114990000008, 45.542773480000044, 0, 0), (-73.39620850000009, 45.542773480000044, 0, 0), (-73.38126710000009,

45.542773480000044, 0, 0), (-73.36632570000009, 45.542773480000044, 0, 0), (-73.35138430000009, 45.542773480000044, 0, 0), (-73.3364429000001, 45.542773480000044, 6, 6), (-73.3215015000001, 45.542773480000044, 0, 0), (-73.3065601000001, 45.542773480000044, 0, 0), (-73.2916187000001, 45.542773480000044, 0, 0), (-74.0237473, 45.552962200000046, 2, 2), (-74.0088059, 45.552962200000046, 0, 0), (-73.9938645, 45.552962200000046, 0, 0), (-73.9789231, 45.552962200000046, 0, 0), (-73.9639817, 45.552962200000046, 0, 0), (-73.94904030000001, 45.552962200000046, 0, 0), (-73.93409890000001, 45.552962200000046, 0, 0), (-73.91915750000001, 45.552962200000046, 0, 0), (-73.90421610000001, 45.552962200000046, 0, 0), (-73.88927470000002, 45.552962200000046, 2, 0), (-73.87433330000002, 45.552962200000046, 0, 1), (-73.85939190000002, 45.552962200000046, 0, 0), (-73.84445050000002, 45.552962200000046, 0, 0), (-73.82950910000002, 45.552962200000046, 0, 0), (-73.81456770000003, 45.552962200000046, 0, 0), (-73.79962630000003, 45.552962200000046, 0, 0), (-73.78468490000003, 45.552962200000046, 0, 1), (-73.76974350000003, 45.552962200000046, 1, 0), (-73.75480210000003, 45.552962200000046, 11, 13), (-73.73986070000004, 45.552962200000046, 12, 7), (-73.72491930000004, 45.552962200000046, 43, 24), (-73.70997790000004, 45.552962200000046, 24, 11), (-73.69503650000004, 45.552962200000046, 7, 56), (-73.68009510000005, 45.552962200000046, 1, 0), (-73.66515370000005, 45.552962200000046, 22, 14), (-73.65021230000005, 45.552962200000046, 8, 6), (-73.63527090000005, 45.552962200000046, 31, 18), (-73.62032950000005, 45.552962200000046, 14, 6), (-73.60538810000006, 45.552962200000046, 211, 95), (-73.59044670000006, 45.552962200000046, 83, 29), (-73.57550530000006, 45.552962200000046, 36, 29), (-73.56056390000006, 45.552962200000046, 62, 45), (-73.54562250000006, 45.552962200000046, 201, 86), (-73.53068110000007, 45.552962200000046, 33, 8), (-73.51573970000007, 45.552962200000046, 0, 0), (-73.50079830000007, 45.552962200000046, 3, 1), (-73.48585690000007, 45.552962200000046, 3, 5), (-73.47091550000007, 45.552962200000046, 0, 0), (-73.45597410000008, 45.552962200000046, 0, 0), (-73.44103270000008, 45.552962200000046, 0, 0), (-73.42609130000008, 45.552962200000046, 0, 0), (-73.41114990000008, 45.552962200000046, 0, 0), (-73.39620850000009, 45.552962200000046, 0, 0), (-73.38126710000009, 45.552962200000046, 0, 0), (-73.36632570000009, 45.552962200000046, 0, 0), (-73.35138430000009, 45.552962200000046, 0, 0), (-73.3364429000001, 45.552962200000046, 0, 0), (-73.3215015000001, 45.552962200000046, 7, 5), (-73.3065601000001, 45.552962200000046, 0, 0), (-73.2916187000001, 45.552962200000046, 0, 0), (-74.0237473, 45.56315092000005, 0, 0), (-74.0088059, 45.56315092000005, 7, 4), (-73.9938645, 45.56315092000005, 0, 0), (-73.9789231, 45.56315092000005, 2, 0), (-73.9639817, 45.56315092000005, 0, 0), (-73.94904030000001, 45.56315092000005, 0, 0), (-73.93409890000001, 45.56315092000005, 0, 0), (-73.91915750000001, 45.56315092000005, 1, 0), (-73.90421610000001, 45.56315092000005, 19, 18), (-73.88927470000002, 45.56315092000005, 6, 35), (-73.87433330000002, 45.56315092000005, 0, 0), (-73.85939190000002, 45.56315092000005, 1, 0), (-73.84445050000002, 45.56315092000005, 3, 1), (-73.82950910000002, 45.56315092000005, 0, 0), (-73.81456770000003, 45.56315092000005, 0, 0), (-73.79962630000003, 45.56315092000005, 6, 3), (-73.78468490000003, 45.56315092000005, 17, 11), (-73.76974350000003, 45.56315092000005, 2, 0), (-73.75480210000003, 45.56315092000005, 22, 6), (-73.73986070000004, 45.56315092000005, 75, 52), (-73.72491930000004, 45.56315092000005, 25, 17), (-73.70997790000004, 45.56315092000005, 4, 0), (-73.69503650000004, 45.56315092000005, 23, 9), (-73.68009510000005, 45.56315092000005, 1, 5), (-73.66515370000005, 45.56315092000005, 320, 142), (-73.65021230000005, 45.56315092000005, 35, 17), (-73.63527090000005, 45.56315092000005, 2, 8), (-73.62032950000005, 45.56315092000005, 30, 15), (-73.60538810000006, 45.56315092000005, 23, 8), (-73.59044670000006, 45.56315092000005, 27, 1), (-73.57550530000006,

45.56315092000005, 38, 24), (-73.56056390000006, 45.56315092000005, 20, 18), (-73.54562250000006, 45.56315092000005, 100, 40), (-73.53068110000007, 45.56315092000005, 5, 3), (-73.51573970000007, 45.56315092000005, 0, 0), (-73.50079830000007, 45.56315092000005, 0, 0), (-73.48585690000007, 45.56315092000005, 1, 2), (-73.47091550000007, 45.56315092000005, 7, 4), (-73.45597410000008, 45.56315092000005, 0, 0), (-73.44103270000008, 45.56315092000005, 0, 0), (-73.42609130000008, 45.56315092000005, 0, 3), (-73.41114990000008, 45.56315092000005, 0, 1), (-73.39620850000009, 45.56315092000005, 0, 0), (-73.38126710000009, 45.56315092000005, 3, 2), (-73.36632570000009, 45.56315092000005, 0, 0), (-73.35138430000009, 45.56315092000005, 0, 0), (-73.3364429000001, 45.56315092000005, 5, 2), (-73.3215015000001, 45.56315092000005, 0, 0), (-73.3065601000001, 45.56315092000005, 0, 0), (-73.2916187000001, 45.56315092000005, 0, 4), (-74.0237473, 45.57333964000005, 0, 0), (-74.0088059, 45.57333964000005, 0, 0), (-73.9938645, 45.57333964000005, 0, 0), (-73.9789231, 45.57333964000005, 0, 0), (-73.9639817, 45.57333964000005, 1, 1), (-73.94904030000001, 45.57333964000005, 0, 0), (-73.93409890000001, 45.57333964000005, 0, 0), (-73.91915750000001, 45.57333964000005, 1, 0), (-73.90421610000001, 45.57333964000005, 5, 0), (-73.88927470000002, 45.57333964000005, 5, 1), (-73.87433330000002, 45.57333964000005, 3, 1), (-73.85939190000002, 45.57333964000005, 1, 0), (-73.84445050000002, 45.57333964000005, 0, 0), (-73.82950910000002, 45.57333964000005, 0, 0), (-73.81456770000003, 45.57333964000005, 21, 8), (-73.79962630000003, 45.57333964000005, 0, 0), (-73.78468490000003, 45.57333964000005, 23, 17), (-73.76974350000003, 45.57333964000005, 2, 0), (-73.75480210000003, 45.57333964000005, 55, 68), (-73.73986070000004, 45.57333964000005, 6, 2), (-73.72491930000004, 45.57333964000005, 10, 2), (-73.70997790000004, 45.57333964000005, 14, 14), (-73.69503650000004, 45.57333964000005, 6, 4), (-73.68009510000005, 45.57333964000005, 5, 3), (-73.66515370000005, 45.57333964000005, 8, 9), (-73.65021230000005, 45.57333964000005, 15, 7), (-73.63527090000005, 45.57333964000005, 4, 3), (-73.62032950000005, 45.57333964000005, 3, 1), (-73.60538810000006, 45.57333964000005, 227, 94), (-73.59044670000006, 45.57333964000005, 8, 16), (-73.57550530000006, 45.57333964000005, 7, 0), (-73.56056390000006, 45.57333964000005, 4, 1), (-73.54562250000006, 45.57333964000005, 13, 1), (-73.53068110000007, 45.57333964000005, 121, 53), (-73.51573970000007, 45.57333964000005, 0, 0), (-73.50079830000007, 45.57333964000005, 0, 0), (-73.48585690000007, 45.57333964000005, 0, 0), (-73.47091550000007, 45.57333964000005, 0, 0), (-73.45597410000008, 45.57333964000005, 91, 18), (-73.44103270000008, 45.57333964000005, 12, 7), (-73.42609130000008, 45.57333964000005, 0, 0), (-73.41114990000008, 45.57333964000005, 0, 1), (-73.39620850000009, 45.57333964000005, 3, 0), (-73.38126710000009, 45.57333964000005, 0, 0), (-73.36632570000009, 45.57333964000005, 0, 0), (-73.35138430000009, 45.57333964000005, 0, 0), (-73.3364429000001, 45.57333964000005, 1, 0), (-73.3215015000001, 45.57333964000005, 2, 0), (-73.3065601000001, 45.57333964000005, 0, 0), (-73.2916187000001, 45.57333964000005, 0, 0), (-74.0237473, 45.58352836000005, 0, 0), (-74.0088059, 45.58352836000005, 0, 0), (-73.9938645, 45.58352836000005, 0, 0), (-73.9789231, 45.58352836000005, 3, 3), (-73.9639817, 45.58352836000005, 0, 0), (-73.94904030000001, 45.58352836000005, 0, 0), (-73.93409890000001, 45.58352836000005, 1, 0), (-73.91915750000001, 45.58352836000005, 0, 0), (-73.90421610000001, 45.58352836000005, 0, 0), (-73.88927470000002, 45.58352836000005, 0, 0), (-73.87433330000002, 45.58352836000005, 8, 3), (-73.85939190000002, 45.58352836000005, 1, 0), (-73.84445050000002, 45.58352836000005, 0, 0), (-73.82950910000002, 45.58352836000005, 0, 0), (-73.81456770000003, 45.58352836000005, 2, 0), (-73.79962630000003, 45.58352836000005, 0, 2), (-73.78468490000003, 45.58352836000005, 5, 4), (-73.76974350000003, 45.58352836000005, 2, 2), (-73.75480210000003, 45.58352836000005, 1, 1), (-73.73986070000004, 45.58352836000005, 0, 2), (-73.72491930000004, 45.58352836000005, 1, 0), (-73.70997790000004, 45.58352836000005, 8,

3), (-73.69503650000004, 45.58352836000005, 1, 1), (-73.68009510000005, 45.58352836000005, 0, 0), (-73.66515370000005, 45.58352836000005, 0, 0), (-73.65021230000005, 45.58352836000005, 6, 2), (-73.63527090000005, 45.58352836000005, 6, 3), (-73.62032950000005, 45.58352836000005, 2, 0), (-73.60538810000006, 45.58352836000005, 22, 30), (-73.59044670000006, 45.58352836000005, 10, 13), (-73.57550530000006, 45.58352836000005, 2, 0), (-73.56056390000006, 45.58352836000005, 6, 3), (-73.54562250000006, 45.58352836000005, 9, 1), (-73.53068110000007, 45.58352836000005, 5, 1), (-73.51573970000007, 45.58352836000005, 0, 0), (-73.50079830000007, 45.58352836000005, 0, 0), (-73.48585690000007, 45.58352836000005, 0, 0), (-73.47091550000007, 45.58352836000005, 0, 0), (-73.45597410000008, 45.58352836000005, 0, 0), (-73.44103270000008, 45.58352836000005, 2, 1), (-73.42609130000008, 45.58352836000005, 0, 0), (-73.41114990000008, 45.58352836000005, 0, 0), (-73.39620850000009, 45.58352836000005, 0, 0), (-73.38126710000009, 45.58352836000005, 0, 0), (-73.36632570000009, 45.58352836000005, 0, 0), (-73.35138430000009, 45.58352836000005, 2, 0), (-73.3364429000001, 45.58352836000005, 0, 0), (-73.3215015000001, 45.58352836000005, 1, 0), (-73.3065601000001, 45.58352836000005, 0, 0), (-73.2916187000001, 45.58352836000005, 0, 0), (-74.0237473, 45.593717080000054, 0, 0), (-74.0088059, 45.593717080000054, 0, 0), (-73.9938645, 45.593717080000054, 0, 0), (-73.9789231, 45.593717080000054, 0, 0), (-73.9639817, 45.593717080000054, 0, 0), (-73.94904030000001, 45.593717080000054, 0, 0), (-73.93409890000001, 45.593717080000054, 0, 1), (-73.91915750000001, 45.593717080000054, 0, 0), (-73.90421610000001, 45.593717080000054, 0, 0), (-73.88927470000002, 45.593717080000054, 0, 0), (-73.87433330000002, 45.593717080000054, 0, 0), (-73.85939190000002, 45.593717080000054, 0, 0), (-73.84445050000002, 45.593717080000054, 0, 0), (-73.82950910000002, 45.593717080000054, 0, 0), (-73.81456770000003, 45.593717080000054, 0, 0), (-73.79962630000003, 45.593717080000054, 3, 0), (-73.78468490000003, 45.593717080000054, 0, 1), (-73.76974350000003, 45.593717080000054, 0, 0), (-73.75480210000003, 45.593717080000054, 0, 0), (-73.73986070000004, 45.593717080000054, 0, 0), (-73.72491930000004, 45.593717080000054, 0, 1), (-73.70997790000004, 45.593717080000054, 0, 0), (-73.69503650000004, 45.593717080000054, 5, 0), (-73.68009510000005, 45.593717080000054, 0, 0), (-73.66515370000005, 45.593717080000054, 68, 11), (-73.65021230000005, 45.593717080000054, 10, 0), (-73.63527090000005, 45.593717080000054, 3, 3), (-73.62032950000005, 45.593717080000054, 0, 0), (-73.60538810000006, 45.593717080000054, 3, 1), (-73.59044670000006, 45.593717080000054, 366, 55), (-73.57550530000006, 45.593717080000054, 49, 14), (-73.56056390000006, 45.593717080000054, 1, 3), (-73.54562250000006, 45.593717080000054, 7, 4), (-73.53068110000007, 45.593717080000054, 6, 2), (-73.51573970000007, 45.593717080000054, 4, 4), (-73.50079830000007, 45.593717080000054, 0, 0), (-73.48585690000007, 45.593717080000054, 0, 0), (-73.47091550000007, 45.593717080000054, 4, 0), (-73.45597410000008, 45.593717080000054, 0, 0), (-73.44103270000008, 45.593717080000054, 46, 160), (-73.42609130000008, 45.593717080000054, 8, 2), (-73.41114990000008, 45.593717080000054, 0, 0), (-73.39620850000009, 45.593717080000054, 0, 0), (-73.38126710000009, 45.593717080000054, 0, 0), (-73.36632570000009, 45.593717080000054, 0, 0), (-73.35138430000009, 45.593717080000054, 1, 1), (-73.3364429000001, 45.593717080000054, 28, 7), (-73.3215015000001, 45.593717080000054, 1, 0), (-73.3065601000001, 45.593717080000054, 0, 0), (-73.2916187000001, 45.593717080000054, 0, 0), (-74.0237473, 45.60390580000006, 0, 0), (-74.0088059, 45.60390580000006, 0, 0), (-73.9938645, 45.60390580000006, 0, 0), (-73.9789231, 45.60390580000006, 0, 0), (-73.9639817, 45.60390580000006, 0, 0), (-73.94904030000001, 45.60390580000006, 0, 0), (-73.93409890000001, 45.60390580000006, 0, 0), (-73.91915750000001, 45.60390580000006, 0, 0), (-73.90421610000001, 45.60390580000006, 0, 0), (-73.88927470000002, 45.60390580000006, 0, 0), (-73.87433330000002, 45.60390580000006, 0, 0), (-73.85939190000002,

45.60390580000006, 0, 0), (-73.84445050000002, 45.60390580000006, 0, 0), (-73.82950910000002, 45.60390580000006, 0, 0), (-73.81456770000003, 45.60390580000006, 1, 0), (-73.79962630000003, 45.60390580000006, 6, 5), (-73.78468490000003, 45.60390580000006, 2, 2), (-73.76974350000003, 45.60390580000006, 1, 0), (-73.75480210000003, 45.60390580000006, 0, 0), (-73.73986070000004, 45.60390580000006, 311, 151), (-73.72491930000004, 45.60390580000006, 1, 2), (-73.70997790000004, 45.60390580000006, 48, 222), (-73.69503650000004, 45.60390580000006, 1, 0), (-73.68009510000005, 45.60390580000006, 21, 6), (-73.66515370000005, 45.60390580000006, 6, 5), (-73.65021230000005, 45.60390580000006, 2, 0), (-73.63527090000005, 45.60390580000006, 11, 33), (-73.62032950000005, 45.60390580000006, 76, 66), (-73.60538810000006, 45.60390580000006, 9, 11), (-73.59044670000006, 45.60390580000006, 0, 2), (-73.57550530000006, 45.60390580000006, 8, 1), (-73.56056390000006, 45.60390580000006, 3, 7), (-73.54562250000006, 45.60390580000006, 4, 0), (-73.53068110000007, 45.60390580000006, 12, 4), (-73.51573970000007, 45.60390580000006, 73, 49), (-73.50079830000007, 45.60390580000006, 0, 0), (-73.48585690000007, 45.60390580000006, 0, 0), (-73.47091550000007, 45.60390580000006, 1, 0), (-73.45597410000008, 45.60390580000006, 65, 25), (-73.44103270000008, 45.60390580000006, 0, 0), (-73.42609130000008, 45.60390580000006, 0, 0), (-73.41114990000008, 45.60390580000006, 0, 0), (-73.39620850000009, 45.60390580000006, 0, 0), (-73.38126710000009, 45.60390580000006, 0, 0), (-73.36632570000009, 45.60390580000006, 0, 0), (-73.35138430000009, 45.60390580000006, 4, 1), (-73.3364429000001, 45.60390580000006, 0, 0), (-73.3215015000001, 45.60390580000006, 0, 0), (-73.3065601000001, 45.60390580000006, 0, 0), (-73.2916187000001, 45.60390580000006, 0, 0), (-74.0237473, 45.61409452000006, 0, 0), (-74.0088059, 45.61409452000006, 0, 0), (-73.9938645, 45.61409452000006, 0, 0), (-73.9789231, 45.61409452000006, 0, 0), (-73.9639817, 45.61409452000006, 0, 0), (-73.94904030000001, 45.61409452000006, 0, 0), (-73.93409890000001, 45.61409452000006, 0, 0), (-73.91915750000001, 45.61409452000006, 0, 0), (-73.90421610000001, 45.61409452000006, 0, 0), (-73.88927470000002, 45.61409452000006, 0, 0), (-73.87433330000002, 45.61409452000006, 0, 0), (-73.85939190000002, 45.61409452000006, 0, 0), (-73.84445050000002, 45.61409452000006, 16, 62), (-73.82950910000002, 45.61409452000006, 6, 1), (-73.81456770000003, 45.61409452000006, 0, 0), (-73.79962630000003, 45.61409452000006, 0, 0), (-73.78468490000003, 45.61409452000006, 52, 21), (-73.76974350000003, 45.61409452000006, 0, 0), (-73.75480210000003, 45.61409452000006, 1, 1), (-73.73986070000004, 45.61409452000006, 1, 2), (-73.72491930000004, 45.61409452000006, 0, 0), (-73.70997790000004, 45.61409452000006, 0, 2), (-73.69503650000004, 45.61409452000006, 1, 0), (-73.68009510000005, 45.61409452000006, 9, 6), (-73.66515370000005, 45.61409452000006, 48, 10), (-73.65021230000005, 45.61409452000006, 11, 5), (-73.63527090000005, 45.61409452000006, 2, 0), (-73.62032950000005, 45.61409452000006, 14, 8), (-73.60538810000006, 45.61409452000006, 39, 23), (-73.59044670000006, 45.61409452000006, 9, 17), (-73.57550530000006, 45.61409452000006, 1, 4), (-73.56056390000006, 45.61409452000006, 8, 2), (-73.54562250000006, 45.61409452000006, 1, 1), (-73.53068110000007, 45.61409452000006, 4, 0), (-73.51573970000007, 45.61409452000006, 4, 6), (-73.50079830000007, 45.61409452000006, 1, 1), (-73.48585690000007, 45.61409452000006, 0, 0), (-73.47091550000007, 45.61409452000006, 22, 2), (-73.45597410000008, 45.61409452000006, 3, 1), (-73.44103270000008, 45.61409452000006, 1, 1), (-73.42609130000008, 45.61409452000006, 0, 0), (-73.41114990000008, 45.61409452000006, 0, 0), (-73.39620850000009, 45.61409452000006, 0, 0), (-73.38126710000009, 45.61409452000006, 0, 0), (-73.36632570000009, 45.61409452000006, 0, 0), (-73.35138430000009, 45.61409452000006, 0, 0), (-73.3364429000001, 45.61409452000006, 0, 0), (-73.3215015000001, 45.61409452000006, 0, 0), (-73.3065601000001, 45.61409452000006, 0, 0), (-73.2916187000001, 45.61409452000006, 0, 0), (-74.0237473, 45.62428324000006, 0, 0), (-74.0088059, 45.62428324000006, 0, 0), (-73.9938645,

45.62428324000006, 0, 0), (-73.9789231, 45.62428324000006, 0, 0), (-73.9639817, 45.62428324000006, 0, 0), (-73.94904030000001, 45.62428324000006, 0, 0), (-73.93409890000001, 45.62428324000006, 0, 0), (-73.91915750000001, 45.62428324000006, 0, 0), (-73.90421610000001, 45.62428324000006, 0, 0), (-73.88927470000002, 45.62428324000006, 0, 0), (-73.87433330000002, 45.62428324000006, 0, 0), (-73.85939190000002, 45.62428324000006, 0, 0), (-73.84445050000002, 45.62428324000006, 32, 13), (-73.82950910000002, 45.62428324000006, 2, 0), (-73.81456770000003, 45.62428324000006, 0, 1), (-73.79962630000003, 45.62428324000006, 3, 2), (-73.78468490000003, 45.62428324000006, 0, 0), (-73.76974350000003, 45.62428324000006, 1, 0), (-73.75480210000003, 45.62428324000006, 2, 3), (-73.73986070000004, 45.62428324000006, 2, 0), (-73.72491930000004, 45.62428324000006, 0, 0), (-73.70997790000004, 45.62428324000006, 0, 0), (-73.69503650000004, 45.62428324000006, 0, 0), (-73.68009510000005, 45.62428324000006, 0, 0), (-73.66515370000005, 45.62428324000006, 0, 0), (-73.65021230000005, 45.62428324000006, 0, 0), (-73.63527090000005, 45.62428324000006, 0, 0), (-73.62032950000005, 45.62428324000006, 14, 2), (-73.60538810000006, 45.62428324000006, 2, 0), (-73.59044670000006, 45.62428324000006, 6, 44), (-73.57550530000006, 45.62428324000006, 0, 0), (-73.56056390000006, 45.62428324000006, 3, 1), (-73.54562250000006, 45.62428324000006, 0, 4), (-73.53068110000007, 45.62428324000006, 0, 0), (-73.51573970000007, 45.62428324000006, 0, 0), (-73.50079830000007, 45.62428324000006, 0, 0), (-73.48585690000007, 45.62428324000006, 0, 0), (-73.47091550000007, 45.62428324000006, 0, 0), (-73.45597410000008, 45.62428324000006, 0, 0), (-73.44103270000008, 45.62428324000006, 0, 0), (-73.42609130000008, 45.62428324000006, 0, 0), (-73.41114990000008, 45.62428324000006, 0, 0), (-73.39620850000009, 45.62428324000006, 0, 0), (-73.38126710000009, 45.62428324000006, 0, 0), (-73.36632570000009, 45.62428324000006, 0, 0), (-73.35138430000009, 45.62428324000006, 0, 0), (-73.3364429000001, 45.62428324000006, 0, 0), (-73.3215015000001, 45.62428324000006, 0, 0), (-73.3065601000001, 45.62428324000006, 0, 0), (-73.2916187000001, 45.62428324000006, 0, 0), (-74.0237473, 45.63447196000006, 0, 0), (-74.0088059, 45.63447196000006, 0, 0), (-73.9938645, 45.63447196000006, 0, 0), (-73.9789231, 45.63447196000006, 0, 0), (-73.9639817, 45.63447196000006, 0, 0), (-73.94904030000001, 45.63447196000006, 0, 0), (-73.93409890000001, 45.63447196000006, 0, 0), (-73.91915750000001, 45.63447196000006, 0, 0), (-73.90421610000001, 45.63447196000006, 0, 0), (-73.88927470000002, 45.63447196000006, 0, 0), (-73.87433330000002, 45.63447196000006, 0, 1), (-73.85939190000002, 45.63447196000006, 5, 9), (-73.84445050000002, 45.63447196000006, 13, 8), (-73.82950910000002, 45.63447196000006, 1, 1), (-73.81456770000003, 45.63447196000006, 10, 9), (-73.79962630000003, 45.63447196000006, 0, 0), (-73.78468490000003, 45.63447196000006, 16, 43), (-73.76974350000003, 45.63447196000006, 1, 0), (-73.75480210000003, 45.63447196000006, 47, 25), (-73.73986070000004, 45.63447196000006, 0, 0), (-73.72491930000004, 45.63447196000006, 0, 0), (-73.70997790000004, 45.63447196000006, 0, 0), (-73.69503650000004, 45.63447196000006, 0, 0), (-73.68009510000005, 45.63447196000006, 0, 0), (-73.66515370000005, 45.63447196000006, 1, 0), (-73.65021230000005, 45.63447196000006, 0, 0), (-73.63527090000005, 45.63447196000006, 0, 0), (-73.62032950000005, 45.63447196000006, 0, 0), (-73.60538810000006, 45.63447196000006, 1, 0), (-73.59044670000006, 45.63447196000006, 12, 4), (-73.57550530000006, 45.63447196000006, 0, 0), (-73.56056390000006, 45.63447196000006, 0, 0), (-73.54562250000006, 45.63447196000006, 1, 0), (-73.53068110000007, 45.63447196000006, 0, 0), (-73.51573970000007, 45.63447196000006, 0, 1), (-73.50079830000007, 45.63447196000006, 6, 23), (-73.48585690000007, 45.63447196000006, 4, 1), (-73.47091550000007, 45.63447196000006, 0, 0), (-73.45597410000008, 45.63447196000006, 0, 0), (-73.44103270000008, 45.63447196000006, 0, 0), (-73.42609130000008, 45.63447196000006, 0, 0), (-73.41114990000008, 45.63447196000006, 0, 0), (-73.39620850000009, 45.63447196000006,

0, 0), (-73.38126710000009, 45.63447196000006, 3, 1), (-73.36632570000009, 45.63447196000006, 0, 0), (-73.35138430000009, 45.63447196000006, 0, 0), (-73.3364429000001, 45.63447196000006, 0, 0), (-73.3215015000001, 45.63447196000006, 0, 0), (-73.3065601000001, 45.63447196000006, 0, 1), (-73.2916187000001, 45.63447196000006, 0, 0), (-74.0237473, 45.644660680000065, 0, 0), (-74.0088059, 45.644660680000065, 0, 0), (-73.9938645, 45.644660680000065, 0, 0), (-73.9789231, 45.644660680000065, 0, 0), (-73.9639817, 45.644660680000065, 0, 0), (-73.94904030000001, 45.644660680000065, 1, 0), (-73.93409890000001, 45.644660680000065, 0, 0), (-73.91915750000001, 45.644660680000065, 0, 0), (-73.90421610000001, 45.644660680000065, 0, 0), (-73.88927470000002, 45.644660680000065, 0, 0), (-73.87433330000002, 45.644660680000065, 0, 0), (-73.85939190000002, 45.644660680000065, 1, 1), (-73.84445050000002, 45.644660680000065, 25, 14), (-73.82950910000002, 45.644660680000065, 2, 1), (-73.81456770000003, 45.644660680000065, 0, 0), (-73.79962630000003, 45.644660680000065, 1, 0), (-73.78468490000003, 45.644660680000065, 1, 0), (-73.76974350000003, 45.644660680000065, 0, 0), (-73.75480210000003, 45.644660680000065, 1, 0), (-73.73986070000004, 45.644660680000065, 0, 1), (-73.72491930000004, 45.644660680000065, 1, 0), (-73.70997790000004, 45.644660680000065, 0, 0), (-73.69503650000004, 45.644660680000065, 0, 0), (-73.68009510000005, 45.644660680000065, 0, 0), (-73.66515370000005, 45.644660680000065, 0, 0), (-73.65021230000005, 45.644660680000065, 0, 0), (-73.63527090000005, 45.644660680000065, 0, 0), (-73.62032950000005, 45.644660680000065, 0, 0), (-73.60538810000006, 45.644660680000065, 0, 0), (-73.59044670000006, 45.644660680000065, 0, 0), (-73.57550530000006, 45.644660680000065, 0, 0), (-73.56056390000006, 45.644660680000065, 0, 0), (-73.54562250000006, 45.644660680000065, 0, 0), (-73.53068110000007, 45.644660680000065, 0, 0), (-73.51573970000007, 45.644660680000065, 2, 0), (-73.50079830000007, 45.644660680000065, 4, 7), (-73.48585690000007, 45.644660680000065, 4, 0), (-73.47091550000007, 45.644660680000065, 0, 0), (-73.45597410000008, 45.644660680000065, 0, 0), (-73.44103270000008, 45.644660680000065, 2, 0), (-73.42609130000008, 45.644660680000065, 0, 0), (-73.41114990000008, 45.644660680000065, 0, 0), (-73.39620850000009, 45.644660680000065, 0, 0), (-73.38126710000009, 45.644660680000065, 0, 0), (-73.36632570000009, 45.644660680000065, 0, 0), (-73.35138430000009, 45.644660680000065, 0, 0), (-73.3364429000001, 45.644660680000065, 0, 0), (-73.3215015000001, 45.644660680000065, 0, 0), (-73.3065601000001, 45.644660680000065, 0, 0), (-73.2916187000001, 45.644660680000065, 0, 0), (-74.0237473, 45.65484940000007, 0, 0), (-74.0088059, 45.65484940000007, 0, 0), (-73.9938645, 45.65484940000007, 0, 0), (-73.9789231, 45.65484940000007, 0, 0), (-73.9639817, 45.65484940000007, 0, 0), (-73.94904030000001, 45.65484940000007, 0, 0), (-73.93409890000001, 45.65484940000007, 0, 0), (-73.91915750000001, 45.65484940000007, 0, 0), (-73.90421610000001, 45.65484940000007, 3, 0), (-73.88927470000002, 45.65484940000007, 0, 0), (-73.87433330000002, 45.65484940000007, 0, 0), (-73.85939190000002, 45.65484940000007, 1, 1), (-73.84445050000002, 45.65484940000007, 0, 0), (-73.82950910000002, 45.65484940000007, 0, 0), (-73.81456770000003, 45.65484940000007, 0, 0), (-73.79962630000003, 45.65484940000007, 3, 1), (-73.78468490000003, 45.65484940000007, 3, 0), (-73.76974350000003, 45.65484940000007, 2, 2), (-73.75480210000003, 45.65484940000007, 0, 0), (-73.73986070000004, 45.65484940000007, 0, 0), (-73.72491930000004, 45.65484940000007, 0, 0), (-73.70997790000004, 45.65484940000007, 0, 0), (-73.69503650000004, 45.65484940000007, 0, 0), (-73.68009510000005, 45.65484940000007, 0, 0), (-73.66515370000005, 45.65484940000007, 0, 0), (-73.65021230000005, 45.65484940000007, 0, 0), (-73.63527090000005, 45.65484940000007, 0, 0), (-73.62032950000005, 45.65484940000007, 0, 0), (-73.60538810000006, 45.65484940000007, 2, 0), (-73.59044670000006, 45.65484940000007, 0, 0), (-73.57550530000006, 45.65484940000007, 2, 0), (-73.56056390000006, 45.65484940000007, 2, 4), (-73.54562250000006,

45.65484940000007, 0, 0), (-73.53068110000007, 45.65484940000007, 0, 2), (-73.51573970000007, 45.65484940000007, 25, 34), (-73.50079830000007, 45.65484940000007, 1, 1), (-73.48585690000007, 45.65484940000007, 4, 2), (-73.47091550000007, 45.65484940000007, 0, 0), (-73.45597410000008, 45.65484940000007, 0, 0), (-73.44103270000008, 45.65484940000007, 1, 1), (-73.42609130000008, 45.65484940000007, 0, 0), (-73.41114990000008, 45.65484940000007, 0, 0), (-73.39620850000009, 45.65484940000007, 0, 0), (-73.38126710000009, 45.65484940000007, 0, 0), (-73.36632570000009, 45.65484940000007, 0, 0), (-73.35138430000009, 45.65484940000007, 0, 0), (-73.3364429000001, 45.65484940000007, 0, 0), (-73.3215015000001, 45.65484940000007, 0, 0), (-73.3065601000001, 45.65484940000007, 4, 0), (-73.2916187000001, 45.65484940000007, 0, 0), (-74.0237473, 45.66503812000007, 0, 0), (-74.0088059, 45.66503812000007, 3, 3), (-73.9938645, 45.66503812000007, 0, 0), (-73.9789231, 45.66503812000007, 0, 0), (-73.9639817, 45.66503812000007, 0, 0), (-73.94904030000001, 45.66503812000007, 0, 0), (-73.93409890000001, 45.66503812000007, 1, 1), (-73.91915750000001, 45.66503812000007, 0, 1), (-73.90421610000001, 45.66503812000007, 0, 0), (-73.88927470000002, 45.66503812000007, 3, 1), (-73.87433330000002, 45.66503812000007, 76, 353), (-73.85939190000002, 45.66503812000007, 1, 0), (-73.84445050000002, 45.66503812000007, 0, 0), (-73.82950910000002, 45.66503812000007, 0, 0), (-73.81456770000003, 45.66503812000007, 8, 4), (-73.79962630000003, 45.66503812000007, 0, 0), (-73.78468490000003, 45.66503812000007, 0, 0), (-73.76974350000003, 45.66503812000007, 0, 0), (-73.75480210000003, 45.66503812000007, 1, 1), (-73.73986070000004, 45.66503812000007, 0, 0), (-73.72491930000004, 45.66503812000007, 0, 0), (-73.70997790000004, 45.66503812000007, 0, 2), (-73.69503650000004, 45.66503812000007, 0, 0), (-73.68009510000005, 45.66503812000007, 0, 0), (-73.66515370000005, 45.66503812000007, 0, 0), (-73.65021230000005, 45.66503812000007, 0, 0), (-73.63527090000005, 45.66503812000007, 2, 0), (-73.62032950000005, 45.66503812000007, 0, 0), (-73.60538810000006, 45.66503812000007, 0, 0), (-73.59044670000006, 45.66503812000007, 1, 0), (-73.57550530000006, 45.66503812000007, 0, 1), (-73.56056390000006, 45.66503812000007, 23, 2), (-73.54562250000006, 45.66503812000007, 3, 0), (-73.53068110000007, 45.66503812000007, 0, 0), (-73.51573970000007, 45.66503812000007, 0, 0), (-73.50079830000007, 45.66503812000007, 6, 8), (-73.48585690000007, 45.66503812000007, 0, 0), (-73.47091550000007, 45.66503812000007, 0, 0), (-73.45597410000008, 45.66503812000007, 0, 0), (-73.44103270000008, 45.66503812000007, 0, 0), (-73.42609130000008, 45.66503812000007, 0, 0), (-73.41114990000008, 45.66503812000007, 0, 0), (-73.39620850000009, 45.66503812000007, 0, 0), (-73.38126710000009, 45.66503812000007, 0, 0), (-73.36632570000009, 45.66503812000007, 0, 0), (-73.35138430000009, 45.66503812000007, 0, 0), (-73.3364429000001, 45.66503812000007, 0, 0), (-73.3215015000001, 45.66503812000007, 0, 0), (-73.3065601000001, 45.66503812000007, 0, 0), (-73.2916187000001, 45.66503812000007, 0, 0), (-74.0237473, 45.67522684000007, 0, 0), (-74.0088059, 45.67522684000007, 0, 0), (-73.9938645, 45.67522684000007, 0, 0), (-73.9789231, 45.67522684000007, 2, 1), (-73.9639817, 45.67522684000007, 0, 0), (-73.94904030000001, 45.67522684000007, 0, 0), (-73.93409890000001, 45.67522684000007, 1, 0), (-73.91915750000001, 45.67522684000007, 1, 1), (-73.90421610000001, 45.67522684000007, 0, 0), (-73.88927470000002, 45.67522684000007, 0, 0), (-73.87433330000002, 45.67522684000007, 3, 3), (-73.85939190000002, 45.67522684000007, 0, 0), (-73.84445050000002, 45.67522684000007, 0, 0), (-73.82950910000002, 45.67522684000007, 0, 0), (-73.81456770000003, 45.67522684000007, 0, 0), (-73.79962630000003, 45.67522684000007, 0, 0), (-73.78468490000003, 45.67522684000007, 2, 0), (-73.76974350000003, 45.67522684000007, 0, 0), (-73.75480210000003, 45.67522684000007, 0, 0), (-73.73986070000004, 45.67522684000007, 1, 0), (-73.72491930000004, 45.67522684000007, 0, 0), (-73.70997790000004, 45.67522684000007, 0, 0), (-73.69503650000004, 45.67522684000007, 0, 0), (-73.68009510000005, 45.67522684000007,

0, 0), (-73.66515370000005, 45.67522684000007, 0, 0), (-73.65021230000005, 45.67522684000007, 0, 0), (-73.63527090000005, 45.67522684000007, 0, 0), (-73.62032950000005, 45.67522684000007, 0, 0), (-73.60538810000006, 45.67522684000007, 0, 0), (-73.59044670000006, 45.67522684000007, 0, 0), (-73.57550530000006, 45.67522684000007, 0, 2), (-73.56056390000006, 45.67522684000007, 0, 0), (-73.54562250000006, 45.67522684000007, 0, 0), (-73.53068110000007, 45.67522684000007, 0, 0), (-73.51573970000007, 45.67522684000007, 0, 0), (-73.50079830000007, 45.67522684000007, 29, 9), (-73.48585690000007, 45.67522684000007, 1, 0), (-73.47091550000007, 45.67522684000007, 0, 0), (-73.45597410000008, 45.67522684000007, 0, 0), (-73.44103270000008, 45.67522684000007, 1, 0), (-73.42609130000008, 45.67522684000007, 0, 1), (-73.41114990000008, 45.67522684000007, 0, 0), (-73.39620850000009, 45.67522684000007, 0, 0), (-73.38126710000009, 45.67522684000007, 0, 0), (-73.36632570000009, 45.67522684000007, 0, 0), (-73.35138430000009, 45.67522684000007, 0, 0), (-73.3364429000001, 45.67522684000007, 0, 0), (-73.3215015000001, 45.67522684000007, 0, 0), (-73.3065601000001, 45.67522684000007, 0, 0), (-73.2916187000001, 45.67522684000007, 0, 0), (-74.0237473, 45.685415560000074, 1, 0), (-74.0088059, 45.685415560000074, 0, 0), (-73.9938645, 45.685415560000074, 0, 0), (-73.9789231, 45.685415560000074, 0, 0), (-73.9639817, 45.685415560000074, 0, 0), (-73.94904030000001, 45.685415560000074, 0, 0), (-73.93409890000001, 45.685415560000074, 0, 0), (-73.91915750000001, 45.685415560000074, 0, 1), (-73.90421610000001, 45.685415560000074, 0, 0), (-73.88927470000002, 45.685415560000074, 1, 1), (-73.87433330000002, 45.685415560000074, 42, 17), (-73.85939190000002, 45.685415560000074, 0, 0), (-73.84445050000002, 45.685415560000074, 0, 0), (-73.82950910000002, 45.685415560000074, 0, 0), (-73.81456770000003, 45.685415560000074, 1, 0), (-73.79962630000003, 45.685415560000074, 0, 0), (-73.78468490000003, 45.685415560000074, 0, 0), (-73.76974350000003, 45.685415560000074, 0, 0), (-73.75480210000003, 45.685415560000074, 0, 0), (-73.73986070000004, 45.685415560000074, 0, 0), (-73.72491930000004, 45.685415560000074, 0, 0), (-73.70997790000004, 45.685415560000074, 0, 0), (-73.69503650000004, 45.685415560000074, 0, 0), (-73.68009510000005, 45.685415560000074, 0, 0), (-73.66515370000005, 45.685415560000074, 0, 0), (-73.65021230000005, 45.685415560000074, 2, 1), (-73.63527090000005, 45.685415560000074, 0, 0), (-73.62032950000005, 45.685415560000074, 0, 0), (-73.60538810000006, 45.685415560000074, 0, 0), (-73.59044670000006, 45.685415560000074, 0, 0), (-73.57550530000006, 45.685415560000074, 0, 0), (-73.56056390000006, 45.685415560000074, 0, 0), (-73.54562250000006, 45.685415560000074, 0, 0), (-73.53068110000007, 45.685415560000074, 0, 0), (-73.51573970000007, 45.685415560000074, 4, 2), (-73.50079830000007, 45.685415560000074, 3, 0), (-73.48585690000007, 45.685415560000074, 0, 0), (-73.47091550000007, 45.685415560000074, 0, 0), (-73.45597410000008, 45.685415560000074, 0, 0), (-73.44103270000008, 45.685415560000074, 14, 7), (-73.42609130000008, 45.685415560000074, 5, 1), (-73.41114990000008, 45.685415560000074, 0, 0), (-73.39620850000009, 45.685415560000074, 0, 0), (-73.38126710000009, 45.685415560000074, 0, 0), (-73.36632570000009, 45.685415560000074, 0, 0), (-73.35138430000009, 45.685415560000074, 0, 0), (-73.3364429000001, 45.685415560000074, 0, 0), (-73.3215015000001, 45.685415560000074, 0, 0), (-73.3065601000001, 45.685415560000074, 0, 0), (-73.2916187000001, 45.685415560000074, 0, 0), (-74.0237473, 45.695604280000076, 0, 0), (-74.0088059, 45.695604280000076, 0, 0), (-73.9938645, 45.695604280000076, 0, 0), (-73.9789231, 45.695604280000076, 0, 0), (-73.9639817, 45.695604280000076, 0, 0), (-73.94904030000001, 45.695604280000076, 0, 0), (-73.93409890000001, 45.695604280000076, 0, 0), (-73.91915750000001, 45.695604280000076, 0, 0), (-73.90421610000001, 45.695604280000076, 0, 0), (-73.88927470000002, 45.695604280000076, 0, 0), (-73.87433330000002, 45.695604280000076, 0, 0), (-73.85939190000002, 45.695604280000076, 0, 0), (-73.84445050000002, 45.695604280000076,

0, 0), (-73.82950910000002, 45.695604280000076, 0, 0), (-73.81456770000003, 45.695604280000076, 0, 0), (-73.79962630000003, 45.695604280000076, 0, 0), (-73.78468490000003, 45.695604280000076, 0, 0), (-73.76974350000003, 45.695604280000076, 0, 0), (-73.75480210000003, 45.695604280000076, 0, 0), (-73.73986070000004, 45.695604280000076, 0, 0), (-73.72491930000004, 45.695604280000076, 0, 0), (-73.70997790000004, 45.695604280000076, 3, 11), (-73.69503650000004, 45.695604280000076, 0, 1), (-73.68009510000005, 45.695604280000076, 0, 0), (-73.66515370000005, 45.695604280000076, 0, 0), (-73.65021230000005, 45.695604280000076, 1, 2), (-73.63527090000005, 45.695604280000076, 38, 35), (-73.62032950000005, 45.695604280000076, 1, 0), (-73.60538810000006, 45.695604280000076, 0, 0), (-73.59044670000006, 45.695604280000076, 0, 0), (-73.57550530000006, 45.695604280000076, 0, 0), (-73.56056390000006, 45.695604280000076, 0, 0), (-73.54562250000006, 45.695604280000076, 0, 0), (-73.53068110000007, 45.695604280000076, 0, 0), (-73.51573970000007, 45.695604280000076, 0, 0), (-73.50079830000007, 45.695604280000076, 0, 0), (-73.48585690000007, 45.695604280000076, 1, 0), (-73.47091550000007, 45.695604280000076, 0, 0), (-73.45597410000008, 45.695604280000076, 0, 0), (-73.44103270000008, 45.695604280000076, 0, 0), (-73.42609130000008, 45.695604280000076, 0, 0), (-73.41114990000008, 45.695604280000076, 0, 0), (-73.39620850000009, 45.695604280000076, 0, 0), (-73.38126710000009, 45.695604280000076, 0, 0), (-73.36632570000009, 45.695604280000076, 0, 0), (-73.35138430000009, 45.695604280000076, 0, 0), (-73.3364429000001, 45.695604280000076, 0, 0), (-73.3215015000001, 45.695604280000076, 0, 0), (-73.3065601000001, 45.695604280000076, 0, 0), (-73.2916187000001, 45.695604280000076, 0, 0), (-74.0237473, 45.70579300000008, 0, 0), (-74.0088059, 45.70579300000008, 0, 0), (-73.9938645, 45.70579300000008, 0, 0), (-73.9789231, 45.70579300000008, 0, 0), (-73.9639817, 45.70579300000008, 0, 0), (-73.94904030000001, 45.70579300000008, 0, 0), (-73.93409890000001, 45.70579300000008, 0, 0), (-73.91915750000001, 45.70579300000008, 0, 0), (-73.90421610000001, 45.70579300000008, 0, 0), (-73.88927470000002, 45.70579300000008, 0, 0), (-73.87433330000002, 45.70579300000008, 0, 0), (-73.85939190000002, 45.70579300000008, 0, 0), (-73.84445050000002, 45.70579300000008, 0, 0), (-73.82950910000002, 45.70579300000008, 0, 0), (-73.81456770000003, 45.70579300000008, 0, 0), (-73.79962630000003, 45.70579300000008, 0, 0), (-73.78468490000003, 45.70579300000008, 0, 0), (-73.76974350000003, 45.70579300000008, 0, 0), (-73.75480210000003, 45.70579300000008, 0, 0), (-73.73986070000004, 45.70579300000008, 0, 0), (-73.72491930000004, 45.70579300000008, 0, 0), (-73.70997790000004, 45.70579300000008, 0, 0), (-73.69503650000004, 45.70579300000008, 0, 0), (-73.68009510000005, 45.70579300000008, 0, 0), (-73.66515370000005, 45.70579300000008, 5, 0), (-73.65021230000005, 45.70579300000008, 78, 27), (-73.63527090000005, 45.70579300000008, 0, 0), (-73.62032950000005, 45.70579300000008, 0, 0), (-73.60538810000006, 45.70579300000008, 0, 0), (-73.59044670000006, 45.70579300000008, 0, 0), (-73.57550530000006, 45.70579300000008, 0, 0), (-73.56056390000006, 45.70579300000008, 0, 0), (-73.54562250000006, 45.70579300000008, 0, 0), (-73.53068110000007, 45.70579300000008, 0, 0), (-73.51573970000007, 45.70579300000008, 0, 0), (-73.50079830000007, 45.70579300000008, 0, 0), (-73.48585690000007, 45.70579300000008, 0, 0), (-73.47091550000007, 45.70579300000008, 0, 1), (-73.45597410000008, 45.70579300000008, 0, 0), (-73.44103270000008, 45.70579300000008, 0, 0), (-73.42609130000008, 45.70579300000008, 0, 0), (-73.41114990000008, 45.70579300000008, 0, 0), (-73.39620850000009, 45.70579300000008, 0, 0), (-73.38126710000009, 45.70579300000008, 0, 0), (-73.36632570000009, 45.70579300000008, 0, 0), (-73.35138430000009, 45.70579300000008, 0, 0), (-73.3364429000001, 45.70579300000008, 0, 0), (-73.3215015000001, 45.70579300000008, 0, 0), (-73.3065601000001, 45.70579300000008, 0, 0), (-73.2916187000001, 45.70579300000008, 0, 0), (-74.0237473, 45.71598172000008, 0, 0), (-74.0088059, 45.71598172000008, 0, 0),

(-73.9938645, 45.71598172000008, 0, 0), (-73.9789231, 45.71598172000008, 0, 0), (-73.9639817, 45.71598172000008, 0, 0), (-73.94904030000001, 45.71598172000008, 0, 0), (-73.93409890000001, 45.71598172000008, 0, 0), (-73.91915750000001, 45.71598172000008, 0, 0), (-73.90421610000001, 45.71598172000008, 0, 0), (-73.88927470000002, 45.71598172000008, 0, 0), (-73.87433330000002, 45.71598172000008, 0, 0), (-73.85939190000002, 45.71598172000008, 0, 0), (-73.84445050000002, 45.71598172000008, 0, 0), (-73.82950910000002, 45.71598172000008, 0, 0), (-73.81456770000003, 45.71598172000008, 0, 0), (-73.79962630000003, 45.71598172000008, 0, 0), (-73.78468490000003, 45.71598172000008, 0, 0), (-73.76974350000003, 45.71598172000008, 0, 0), (-73.75480210000003, 45.71598172000008, 0, 0), (-73.73986070000004, 45.71598172000008, 0, 0), (-73.72491930000004, 45.71598172000008, 0, 0), (-73.70997790000004, 45.71598172000008, 9, 2), (-73.69503650000004, 45.71598172000008, 0, 0), (-73.68009510000005, 45.71598172000008, 0, 0), (-73.66515370000005, 45.71598172000008, 1, 0), (-73.65021230000005, 45.71598172000008, 1, 1), (-73.63527090000005, 45.71598172000008, 0, 0), (-73.62032950000005, 45.71598172000008, 0, 0), (-73.60538810000006, 45.71598172000008, 0, 0), (-73.59044670000006, 45.71598172000008, 0, 0), (-73.57550530000006, 45.71598172000008, 0, 0), (-73.56056390000006, 45.71598172000008, 0, 0), (-73.54562250000006, 45.71598172000008, 13, 28), (-73.53068110000007, 45.71598172000008, 0, 0), (-73.51573970000007, 45.71598172000008, 1, 7), (-73.50079830000007, 45.71598172000008, 0, 1), (-73.48585690000007, 45.71598172000008, 1, 1), (-73.47091550000007, 45.71598172000008, 0, 1), (-73.45597410000008, 45.71598172000008, 0, 0), (-73.44103270000008, 45.71598172000008, 0, 0), (-73.42609130000008, 45.71598172000008, 0, 0), (-73.41114990000008, 45.71598172000008, 0, 0), (-73.39620850000009, 45.71598172000008, 0, 0), (-73.38126710000009, 45.71598172000008, 0, 0), (-73.36632570000009, 45.71598172000008, 0, 0), (-73.35138430000009, 45.71598172000008, 0, 0), (-73.3364429000001, 45.71598172000008, 0, 0), (-73.3215015000001, 45.71598172000008, 0, 0), (-73.3065601000001, 45.71598172000008, 0, 0), (-73.2916187000001, 45.71598172000008, 0, 0), (-74.0237473, 45.72617044000008, 0, 0), (-74.0088059, 45.72617044000008, 0, 0), (-73.9938645, 45.72617044000008, 0, 0), (-73.9789231, 45.72617044000008, 0, 0), (-73.9639817, 45.72617044000008, 0, 0), (-73.94904030000001, 45.72617044000008, 0, 0), (-73.93409890000001, 45.72617044000008, 0, 0), (-73.91915750000001, 45.72617044000008, 0, 0), (-73.90421610000001, 45.72617044000008, 0, 0), (-73.88927470000002, 45.72617044000008, 0, 0), (-73.87433330000002, 45.72617044000008, 0, 0), (-73.85939190000002, 45.72617044000008, 0, 0), (-73.84445050000002, 45.72617044000008, 0, 0), (-73.82950910000002, 45.72617044000008, 1, 3), (-73.81456770000003, 45.72617044000008, 0, 0), (-73.79962630000003, 45.72617044000008, 0, 0), (-73.78468490000003, 45.72617044000008, 0, 0), (-73.76974350000003, 45.72617044000008, 0, 0), (-73.75480210000003, 45.72617044000008, 0, 0), (-73.73986070000004, 45.72617044000008, 0, 0), (-73.72491930000004, 45.72617044000008, 0, 0), (-73.70997790000004, 45.72617044000008, 0, 0), (-73.69503650000004, 45.72617044000008, 1, 1), (-73.68009510000005, 45.72617044000008, 0, 0), (-73.66515370000005, 45.72617044000008, 0, 0), (-73.65021230000005, 45.72617044000008, 0, 0), (-73.63527090000005, 45.72617044000008, 0, 0), (-73.62032950000005, 45.72617044000008, 0, 0), (-73.60538810000006, 45.72617044000008, 0, 0), (-73.59044670000006, 45.72617044000008, 0, 0), (-73.57550530000006, 45.72617044000008, 0, 0), (-73.56056390000006, 45.72617044000008, 0, 0), (-73.54562250000006, 45.72617044000008, 0, 0), (-73.53068110000007, 45.72617044000008, 0, 0), (-73.51573970000007, 45.72617044000008, 0, 0), (-73.50079830000007, 45.72617044000008, 0, 0), (-73.48585690000007, 45.72617044000008, 1, 0), (-73.47091550000007, 45.72617044000008, 1, 0), (-73.45597410000008, 45.72617044000008, 0, 0), (-73.44103270000008, 45.72617044000008, 0, 0), (-73.42609130000008, 45.72617044000008, 0, 0), (-73.41114990000008, 45.72617044000008, 0, 0), (-73.39620850000009, 45.72617044000008, 0,

0), (-73.38126710000009, 45.72617044000008, 0, 0), (-73.36632570000009, 45.72617044000008, 0, 0), (-73.35138430000009, 45.72617044000008, 0, 0), (-73.3364429000001, 45.72617044000008, 0, 0), (-73.3215015000001, 45.72617044000008, 0, 0), (-73.3065601000001, 45.72617044000008, 0, 0), (-73.2916187000001, 45.72617044000008, 0, 0), (-74.0237473, 45.736359160000085, 0, 0), (-74.0088059, 45.736359160000085, 0, 0), (-73.9938645, 45.736359160000085, 0, 0), (-73.9789231, 45.736359160000085, 0, 0), (-73.9639817, 45.736359160000085, 0, 0), (-73.94904030000001, 45.736359160000085, 0, 0), (-73.93409890000001, 45.736359160000085, 0, 0), (-73.91915750000001, 45.736359160000085, 0, 0), (-73.90421610000001, 45.736359160000085, 0, 0), (-73.88927470000002, 45.736359160000085, 0, 0), (-73.87433330000002, 45.736359160000085, 0, 0), (-73.85939190000002, 45.736359160000085, 0, 0), (-73.84445050000002, 45.736359160000085, 0, 0), (-73.82950910000002, 45.736359160000085, 0, 0), (-73.81456770000003, 45.736359160000085, 0, 0), (-73.79962630000003, 45.736359160000085, 0, 0), (-73.78468490000003, 45.736359160000085, 0, 0), (-73.76974350000003, 45.736359160000085, 0, 0), (-73.75480210000003, 45.736359160000085, 0, 0), (-73.73986070000004, 45.736359160000085, 0, 0), (-73.72491930000004, 45.736359160000085, 3, 2), (-73.70997790000004, 45.736359160000085, 0, 0), (-73.69503650000004, 45.736359160000085, 0, 0), (-73.68009510000005, 45.736359160000085, 1, 1), (-73.66515370000005, 45.736359160000085, 0, 0), (-73.65021230000005, 45.736359160000085, 0, 39), (-73.63527090000005, 45.736359160000085, 0, 0), (-73.62032950000005, 45.736359160000085, 0, 0), (-73.60538810000006, 45.736359160000085, 0, 1), (-73.59044670000006, 45.736359160000085, 0, 0), (-73.57550530000006, 45.736359160000085, 0, 0), (-73.56056390000006, 45.736359160000085, 0, 0), (-73.54562250000006, 45.736359160000085, 0, 0), (-73.53068110000007, 45.736359160000085, 0, 0), (-73.51573970000007, 45.736359160000085, 0, 0), (-73.50079830000007, 45.736359160000085, 0, 0), (-73.48585690000007, 45.736359160000085, 0, 0), (-73.47091550000007, 45.736359160000085, 0, 0), (-73.45597410000008, 45.736359160000085, 27, 18), (-73.44103270000008, 45.736359160000085, 3, 1), (-73.42609130000008, 45.736359160000085, 0, 0), (-73.41114990000008, 45.736359160000085, 0, 0), (-73.39620850000009, 45.736359160000085, 0, 0), (-73.38126710000009, 45.736359160000085, 0, 0), (-73.36632570000009, 45.736359160000085, 0, 0), (-73.35138430000009, 45.736359160000085, 0, 0), (-73.3364429000001, 45.736359160000085, 0, 0), (-73.3215015000001, 45.736359160000085, 0, 0), (-73.3065601000001, 45.736359160000085, 0, 0), (-73.2916187000001, 45.736359160000085, 0, 0), (-74.0237473, 45.74654788000009, 0, 0), (-74.0088059, 45.74654788000009, 0, 0), (-73.9938645, 45.74654788000009, 1, 3), (-73.9789231, 45.74654788000009, 0, 0), (-73.9639817, 45.74654788000009, 0, 0), (-73.94904030000001, 45.74654788000009, 0, 0), (-73.93409890000001, 45.74654788000009, 0, 0), (-73.91915750000001, 45.74654788000009, 0, 0), (-73.90421610000001, 45.74654788000009, 0, 0), (-73.88927470000002, 45.74654788000009, 0, 0), (-73.87433330000002, 45.74654788000009, 0, 0), (-73.85939190000002, 45.74654788000009, 0, 0), (-73.84445050000002, 45.74654788000009, 0, 0), (-73.82950910000002, 45.74654788000009, 0, 0), (-73.81456770000003, 45.74654788000009, 0, 0), (-73.79962630000003, 45.74654788000009, 0, 0), (-73.78468490000003, 45.74654788000009, 0, 0), (-73.76974350000003, 45.74654788000009, 0, 0), (-73.75480210000003, 45.74654788000009, 0, 0), (-73.73986070000004, 45.74654788000009, 0, 0), (-73.72491930000004, 45.74654788000009, 5, 4), (-73.70997790000004, 45.74654788000009, 0, 0), (-73.69503650000004, 45.74654788000009, 0, 0), (-73.68009510000005, 45.74654788000009, 0, 0), (-73.66515370000005, 45.74654788000009, 0, 0), (-73.65021230000005, 45.74654788000009, 0, 0), (-73.63527090000005, 45.74654788000009, 0, 0), (-73.62032950000005, 45.74654788000009, 2, 1), (-73.60538810000006, 45.74654788000009, 12, 8), (-73.59044670000006, 45.74654788000009, 0, 0), (-73.57550530000006, 45.74654788000009, 0, 0), (-73.56056390000006, 45.74654788000009, 0, 0), (-73.54562250000006,

45.74654788000009, 0, 0), (-73.53068110000007, 45.74654788000009, 0, 0), (-73.51573970000007, 45.74654788000009, 0, 0), (-73.50079830000007, 45.74654788000009, 0, 0), (-73.48585690000007, 45.74654788000009, 0, 0), (-73.47091550000007, 45.74654788000009, 1, 0), (-73.45597410000008, 45.74654788000009, 3, 2), (-73.44103270000008, 45.74654788000009, 2, 1), (-73.42609130000008, 45.74654788000009, 0, 0), (-73.41114990000008, 45.74654788000009, 0, 0), (-73.39620850000009, 45.74654788000009, 0, 0), (-73.38126710000009, 45.74654788000009, 0, 0), (-73.36632570000009, 45.74654788000009, 0, 0), (-73.35138430000009, 45.74654788000009, 0, 0), (-73.3364429000001, 45.74654788000009, 0, 0), (-73.3215015000001, 45.74654788000009, 0, 0), (-73.3065601000001, 45.74654788000009, 0, 0), (-73.2916187000001, 45.74654788000009, 0, 0), (-74.0237473, 45.75673660000009, 0, 1), (-74.0088059, 45.75673660000009, 0, 0), (-73.9938645, 45.75673660000009, 0, 0), (-73.9789231, 45.75673660000009, 0, 0), (-73.9639817, 45.75673660000009, 0, 0), (-73.94904030000001, 45.75673660000009, 0, 0), (-73.93409890000001, 45.75673660000009, 0, 0), (-73.91915750000001, 45.75673660000009, 1, 0), (-73.90421610000001, 45.75673660000009, 0, 0), (-73.88927470000002, 45.75673660000009, 0, 0), (-73.87433330000002, 45.75673660000009, 0, 0), (-73.85939190000002, 45.75673660000009, 0, 0), (-73.84445050000002, 45.75673660000009, 0, 0), (-73.82950910000002, 45.75673660000009, 0, 0), (-73.81456770000003, 45.75673660000009, 0, 0), (-73.79962630000003, 45.75673660000009, 0, 0), (-73.78468490000003, 45.75673660000009, 0, 0), (-73.76974350000003, 45.75673660000009, 0, 0), (-73.75480210000003, 45.75673660000009, 0, 0), (-73.73986070000004, 45.75673660000009, 0, 0), (-73.72491930000004, 45.75673660000009, 0, 0), (-73.70997790000004, 45.75673660000009, 0, 0), (-73.69503650000004, 45.75673660000009, 0, 0), (-73.68009510000005, 45.75673660000009, 0, 0), (-73.66515370000005, 45.75673660000009, 0, 0), (-73.65021230000005, 45.75673660000009, 0, 0), (-73.63527090000005, 45.75673660000009, 0, 0), (-73.62032950000005, 45.75673660000009, 0, 0), (-73.60538810000006, 45.75673660000009, 0, 0), (-73.59044670000006, 45.75673660000009, 0, 0), (-73.57550530000006, 45.75673660000009, 0, 0), (-73.56056390000006, 45.75673660000009, 0, 0), (-73.54562250000006, 45.75673660000009, 0, 0), (-73.53068110000007, 45.75673660000009, 0, 0), (-73.51573970000007, 45.75673660000009, 1, 0), (-73.50079830000007, 45.75673660000009, 1, 1), (-73.48585690000007, 45.75673660000009, 0, 0), (-73.47091550000007, 45.75673660000009, 1, 1), (-73.45597410000008, 45.75673660000009, 0, 0), (-73.44103270000008, 45.75673660000009, 3, 21), (-73.42609130000008, 45.75673660000009, 0, 0), (-73.41114990000008, 45.75673660000009, 0, 0), (-73.39620850000009, 45.75673660000009, 0, 0), (-73.38126710000009, 45.75673660000009, 0, 0), (-73.36632570000009, 45.75673660000009, 0, 0), (-73.35138430000009, 45.75673660000009, 0, 0), (-73.3364429000001, 45.75673660000009, 0, 0), (-73.3215015000001, 45.75673660000009, 0, 0), (-73.3065601000001, 45.75673660000009, 0, 0), (-73.2916187000001, 45.75673660000009, 0, 0), (-74.0237473, 45.76692532000009, 1, 0), (-74.0088059, 45.76692532000009, 0, 0), (-73.9938645, 45.76692532000009, 9, 3), (-73.9789231, 45.76692532000009, 0, 2), (-73.9639817, 45.76692532000009, 0, 0), (-73.94904030000001, 45.76692532000009, 0, 0), (-73.93409890000001, 45.76692532000009, 0, 0), (-73.91915750000001, 45.76692532000009, 0, 0), (-73.90421610000001, 45.76692532000009, 0, 0), (-73.88927470000002, 45.76692532000009, 0, 0), (-73.87433330000002, 45.76692532000009, 0, 0), (-73.85939190000002, 45.76692532000009, 0, 0), (-73.84445050000002, 45.76692532000009, 0, 0), (-73.82950910000002, 45.76692532000009, 0, 0), (-73.81456770000003, 45.76692532000009, 0, 0), (-73.79962630000003, 45.76692532000009, 0, 0), (-73.78468490000003, 45.76692532000009, 0, 0), (-73.76974350000003, 45.76692532000009, 0, 0), (-73.75480210000003, 45.76692532000009, 0, 0), (-73.73986070000004, 45.76692532000009, 1, 0), (-73.72491930000004, 45.76692532000009, 0, 0), (-73.70997790000004, 45.76692532000009, 0, 0), (-73.69503650000004, 45.76692532000009, 0, 0), (-73.68009510000005, 45.76692532000009,

0, 0), (-73.66515370000005, 45.76692532000009, 0, 0), (-73.65021230000005, 45.76692532000009, 0, 0), (-73.63527090000005, 45.76692532000009, 0, 0), (-73.62032950000005, 45.76692532000009, 0, 0), (-73.60538810000006, 45.76692532000009, 0, 0), (-73.59044670000006, 45.76692532000009, 0, 0), (-73.57550530000006, 45.76692532000009, 0, 0), (-73.56056390000006, 45.76692532000009, 0, 0), (-73.54562250000006, 45.76692532000009, 0, 0), (-73.53068110000007, 45.76692532000009, 0, 0), (-73.51573970000007, 45.76692532000009, 0, 0), (-73.50079830000007, 45.76692532000009, 0, 0), (-73.48585690000007, 45.76692532000009, 2, 0), (-73.47091550000007, 45.76692532000009, 1, 0), (-73.45597410000008, 45.76692532000009, 0, 0), (-73.44103270000008, 45.76692532000009, 1, 0), (-73.42609130000008, 45.76692532000009, 0, 0), (-73.41114990000008, 45.76692532000009, 0, 0), (-73.39620850000009, 45.76692532000009, 0, 0), (-73.38126710000009, 45.76692532000009, 0, 0), (-73.36632570000009, 45.76692532000009, 0, 0), (-73.35138430000009, 45.76692532000009, 12, 48), (-73.3364429000001, 45.76692532000009, 0, 0), (-73.3215015000001, 45.76692532000009, 0, 0), (-73.3065601000001, 45.76692532000009, 0, 0), (-73.2916187000001, 45.76692532000009, 0, 0), (-74.0237473, 45.77711404000009, 0, 0), (-74.0088059, 45.77711404000009, 17, 77), (-73.9938645, 45.77711404000009, 15, 7), (-73.9789231, 45.77711404000009, 0, 0), (-73.9639817, 45.77711404000009, 0, 0), (-73.94904030000001, 45.77711404000009, 0, 0), (-73.93409890000001, 45.77711404000009, 0, 0), (-73.91915750000001, 45.77711404000009, 0, 0), (-73.90421610000001, 45.77711404000009, 0, 0), (-73.88927470000002, 45.77711404000009, 0, 0), (-73.87433330000002, 45.77711404000009, 0, 0), (-73.85939190000002, 45.77711404000009, 0, 0), (-73.84445050000002, 45.77711404000009, 0, 0), (-73.82950910000002, 45.77711404000009, 0, 0), (-73.81456770000003, 45.77711404000009, 0, 0), (-73.79962630000003, 45.77711404000009, 0, 0), (-73.78468490000003, 45.77711404000009, 0, 0), (-73.76974350000003, 45.77711404000009, 0, 0), (-73.75480210000003, 45.77711404000009, 0, 0), (-73.73986070000004, 45.77711404000009, 2, 1), (-73.72491930000004, 45.77711404000009, 0, 0), (-73.70997790000004, 45.77711404000009, 0, 0), (-73.69503650000004, 45.77711404000009, 0, 0), (-73.68009510000005, 45.77711404000009, 0, 0), (-73.66515370000005, 45.77711404000009, 0, 0), (-73.65021230000005, 45.77711404000009, 0, 0), (-73.63527090000005, 45.77711404000009, 3, 9), (-73.62032950000005, 45.77711404000009, 0, 0), (-73.60538810000006, 45.77711404000009, 0, 0), (-73.59044670000006, 45.77711404000009, 0, 0), (-73.57550530000006, 45.77711404000009, 0, 0), (-73.56056390000006, 45.77711404000009, 0, 0), (-73.54562250000006, 45.77711404000009, 0, 0), (-73.53068110000007, 45.77711404000009, 0, 0), (-73.51573970000007, 45.77711404000009, 0, 0), (-73.50079830000007, 45.77711404000009, 0, 0), (-73.48585690000007, 45.77711404000009, 0, 0), (-73.47091550000007, 45.77711404000009, 0, 0), (-73.45597410000008, 45.77711404000009, 0, 0), (-73.44103270000008, 45.77711404000009, 0, 0), (-73.42609130000008, 45.77711404000009, 0, 0), (-73.41114990000008, 45.77711404000009, 0, 0), (-73.39620850000009, 45.77711404000009, 0, 0), (-73.38126710000009, 45.77711404000009, 0, 0), (-73.36632570000009, 45.77711404000009, 29, 159), (-73.35138430000009, 45.77711404000009, 1, 1), (-73.3364429000001, 45.77711404000009, 0, 0), (-73.3215015000001, 45.77711404000009, 0, 0), (-73.3065601000001, 45.77711404000009, 0, 0), (-73.2916187000001, 45.77711404000009, 0, 0), (-74.0237473, 45.787302760000095, 0, 0), (-74.0088059, 45.787302760000095, 0, 0), (-73.9938645, 45.787302760000095, 2, 1), (-73.9789231, 45.787302760000095, 0, 0), (-73.9639817, 45.787302760000095, 0, 0), (-73.94904030000001, 45.787302760000095, 0, 0), (-73.93409890000001, 45.787302760000095, 0, 0), (-73.91915750000001, 45.787302760000095, 0, 0), (-73.90421610000001, 45.787302760000095, 0, 0), (-73.88927470000002, 45.787302760000095, 0, 0), (-73.87433330000002, 45.787302760000095, 0, 0), (-73.85939190000002, 45.787302760000095, 0, 0), (-73.84445050000002, 45.787302760000095, 0, 0), (-73.82950910000002, 45.787302760000095, 0, 0), (-73.81456770000003,

45.787302760000095, 0, 0), (-73.79962630000003, 45.787302760000095, 0, 0), (-73.78468490000003, 45.787302760000095, 0, 0), (-73.76974350000003, 45.787302760000095, 0, 0), (-73.75480210000003, 45.787302760000095, 0, 0), (-73.73986070000004, 45.787302760000095, 0, 0), (-73.72491930000004, 45.787302760000095, 0, 0), (-73.70997790000004, 45.787302760000095, 0, 0), (-73.69503650000004, 45.787302760000095, 0, 0), (-73.68009510000005, 45.787302760000095, 0, 0), (-73.66515370000005, 45.787302760000095, 0, 0), (-73.65021230000005, 45.787302760000095, 0, 0), (-73.63527090000005, 45.787302760000095, 0, 0), (-73.62032950000005, 45.787302760000095, 0, 0), (-73.60538810000006, 45.787302760000095, 0, 0), (-73.59044670000006, 45.787302760000095, 0, 0), (-73.57550530000006, 45.787302760000095, 0, 0), (-73.56056390000006, 45.787302760000095, 0, 0), (-73.54562250000006, 45.787302760000095, 0, 0), (-73.53068110000007, 45.787302760000095, 0, 0), (-73.51573970000007, 45.787302760000095, 0, 0), (-73.50079830000007, 45.787302760000095, 2, 1), (-73.48585690000007, 45.787302760000095, 0, 0), (-73.47091550000007, 45.787302760000095, 0, 0), (-73.45597410000008, 45.787302760000095, 0, 0), (-73.44103270000008, 45.787302760000095, 0, 0), (-73.42609130000008, 45.787302760000095, 0, 0), (-73.41114990000008, 45.787302760000095, 0, 0), (-73.39620850000009, 45.787302760000095, 0, 0), (-73.38126710000009, 45.787302760000095, 0, 0), (-73.36632570000009, 45.787302760000095, 0, 0), (-73.35138430000009, 45.787302760000095, 0, 0), (-73.3364429000001, 45.787302760000095, 0, 0), (-73.3215015000001, 45.787302760000095, 0, 0), (-73.3065601000001, 45.787302760000095, 0, 0), (-73.2916187000001, 45.787302760000095, 0, 0), (-74.0237473, 45.7974914800001, 0, 0), (-74.0088059, 45.7974914800001, 0, 0), (-73.9938645, 45.7974914800001, 0, 0), (-73.9789231, 45.7974914800001, 0, 0), (-73.9639817, 45.7974914800001, 0, 0), (-73.94904030000001, 45.7974914800001, 0, 0), (-73.93409890000001, 45.7974914800001, 0, 0), (-73.91915750000001, 45.7974914800001, 0, 0), (-73.90421610000001, 45.7974914800001, 0, 0), (-73.88927470000002, 45.7974914800001, 0, 0), (-73.87433330000002, 45.7974914800001, 0, 0), (-73.85939190000002, 45.7974914800001, 0, 0), (-73.84445050000002, 45.7974914800001, 0, 0), (-73.82950910000002, 45.7974914800001, 0, 0), (-73.81456770000003, 45.7974914800001, 0, 0), (-73.79962630000003, 45.7974914800001, 0, 0), (-73.78468490000003, 45.7974914800001, 0, 0), (-73.76974350000003, 45.7974914800001, 0, 0), (-73.75480210000003, 45.7974914800001, 0, 0), (-73.73986070000004, 45.7974914800001, 0, 0), (-73.72491930000004, 45.7974914800001, 0, 0), (-73.70997790000004, 45.7974914800001, 0, 0), (-73.69503650000004, 45.7974914800001, 0, 0), (-73.68009510000005, 45.7974914800001, 0, 0), (-73.66515370000005, 45.7974914800001, 0, 0), (-73.65021230000005, 45.7974914800001, 0, 0), (-73.63527090000005, 45.7974914800001, 0, 0), (-73.62032950000005, 45.7974914800001, 0, 0), (-73.60538810000006, 45.7974914800001, 0, 0), (-73.59044670000006, 45.7974914800001, 0, 0), (-73.57550530000006, 45.7974914800001, 0, 0), (-73.56056390000006, 45.7974914800001, 0, 0), (-73.54562250000006, 45.7974914800001, 0, 0), (-73.53068110000007, 45.7974914800001, 0, 0), (-73.51573970000007, 45.7974914800001, 0, 0), (-73.50079830000007, 45.7974914800001, 0, 0), (-73.48585690000007, 45.7974914800001, 0, 0), (-73.47091550000007, 45.7974914800001, 0, 0), (-73.45597410000008, 45.7974914800001, 1, 1), (-73.44103270000008, 45.7974914800001, 0, 0), (-73.42609130000008, 45.7974914800001, 0, 0), (-73.41114990000008, 45.7974914800001, 0, 0), (-73.39620850000009, 45.7974914800001, 0, 0), (-73.38126710000009, 45.7974914800001, 0, 0), (-73.36632570000009, 45.7974914800001, 0, 0), (-73.35138430000009, 45.7974914800001, 0, 0), (-73.3364429000001, 45.7974914800001, 0, 0), (-73.3215015000001, 45.7974914800001, 0, 0), (-73.3065601000001, 45.7974914800001, 0, 0), (-73.2916187000001, 45.7974914800001, 0, 0), (-74.0237473, 45.8076802000001, 0, 0), (-74.0088059, 45.8076802000001, 0, 0), (-73.9938645, 45.8076802000001, 0, 0), (-73.9789231, 45.8076802000001, 0, 0), (-73.9639817, 45.8076802000001, 0, 0), (-73.94904030000001, 45.8076802000001, 0, 0), (-73.93409890000001, 45.8076802000001, 0, 0), (-73.91915750000001, 45.8076802000001, 0, 0), (-73.90421610000001, 45.8076802000001, 0, 0), (-73.88927470000002, 45.8076802000001, 0, 0), (-73.87433330000002,

45.8076802000001, 0, 0), (-73.85939190000002, 45.8076802000001, 0, 0), (-73.84445050000002, 45.8076802000001, 0, 0), (-73.82950910000002, 45.8076802000001, 0, 0), (-73.81456770000003, 45.8076802000001, 0, 0), (-73.79962630000003, 45.8076802000001, 0, 0), (-73.78468490000003, 45.8076802000001, 0, 0), (-73.76974350000003, 45.8076802000001, 0, 0), (-73.75480210000003, 45.8076802000001, 0, 0), (-73.73986070000004, 45.8076802000001, 0, 0), (-73.72491930000004, 45.8076802000001, 0, 0), (-73.70997790000004, 45.8076802000001, 0, 0), (-73.69503650000004, 45.8076802000001, 0, 0), (-73.68009510000005, 45.8076802000001, 0, 0), (-73.66515370000005, 45.8076802000001, 0, 0), (-73.65021230000005, 45.8076802000001, 0, 0), (-73.63527090000005, 45.8076802000001, 0, 0), (-73.62032950000005, 45.8076802000001, 0, 0), (-73.60538810000006, 45.8076802000001, 0, 0), (-73.59044670000006, 45.8076802000001, 0, 0), (-73.57550530000006, 45.8076802000001, 0, 0), (-73.56056390000006, 45.8076802000001, 0, 0), (-73.54562250000006, 45.8076802000001, 0, 0), (-73.53068110000007, 45.8076802000001, 0, 0), (-73.51573970000007, 45.8076802000001, 0, 0), (-73.50079830000007, 45.8076802000001, 0, 0), (-73.48585690000007, 45.8076802000001, 0, 0), (-73.47091550000007, 45.8076802000001, 0, 0), (-73.45597410000008, 45.8076802000001, 0, 0), (-73.44103270000008, 45.8076802000001, 0, 0), (-73.42609130000008, 45.8076802000001, 0, 0), (-73.41114990000008, 45.8076802000001, 0, 0), (-73.39620850000009, 45.8076802000001, 0, 0), (-73.38126710000009, 45.8076802000001, 0, 0), (-73.36632570000009, 45.8076802000001, 0, 0), (-73.35138430000009, 45.8076802000001, 0, 0), (-73.3364429000001, 45.8076802000001, 0, 0), (-73.3215015000001, 45.8076802000001, 0, 0), (-73.3065601000001, 45.8076802000001, 0, 0), (-73.2916187000001, 45.8076802000001, 0, 0), (-74.0237473, 45.8178689200001, 0, 0), (-74.0088059, 45.8178689200001, 0, 0), (-73.9938645, 45.8178689200001, 0, 0), (-73.9789231, 45.8178689200001, 0, 0), (-73.9639817, 45.8178689200001, 0, 0), (-73.94904030000001, 45.8178689200001, 0, 0), (-73.93409890000001, 45.8178689200001, 0, 10), (-73.91915750000001, 45.8178689200001, 0, 0), (-73.90421610000001, 45.8178689200001, 1, 1), (-73.88927470000002, 45.8178689200001, 0, 0), (-73.87433330000002, 45.8178689200001, 0, 0), (-73.85939190000002, 45.8178689200001, 0, 0), (-73.84445050000002, 45.8178689200001, 0, 0), (-73.82950910000002, 45.8178689200001, 0, 0), (-73.81456770000003, 45.8178689200001, 0, 0), (-73.79962630000003, 45.8178689200001, 0, 0), (-73.78468490000003, 45.8178689200001, 0, 0), (-73.76974350000003, 45.8178689200001, 0, 0), (-73.75480210000003, 45.8178689200001, 0, 0), (-73.73986070000004, 45.8178689200001, 0, 0), (-73.72491930000004, 45.8178689200001, 0, 0), (-73.70997790000004, 45.8178689200001, 0, 0), (-73.69503650000004, 45.8178689200001, 0, 0), (-73.68009510000005, 45.8178689200001, 0, 0), (-73.66515370000005, 45.8178689200001, 0, 0), (-73.65021230000005, 45.8178689200001, 0, 0), (-73.63527090000005, 45.8178689200001, 0, 0), (-73.62032950000005, 45.8178689200001, 0, 0), (-73.60538810000006, 45.8178689200001, 1, 0), (-73.59044670000006, 45.8178689200001, 0, 0), (-73.57550530000006, 45.8178689200001, 0, 0), (-73.56056390000006, 45.8178689200001, 0, 0), (-73.54562250000006, 45.8178689200001, 0, 0), (-73.53068110000007, 45.8178689200001, 0, 0), (-73.51573970000007, 45.8178689200001, 0, 0), (-73.50079830000007, 45.8178689200001, 0, 0), (-73.48585690000007, 45.8178689200001, 0, 0), (-73.47091550000007, 45.8178689200001, 0, 0), (-73.45597410000008, 45.8178689200001, 0, 0), (-73.44103270000008, 45.8178689200001, 1, 0), (-73.42609130000008, 45.8178689200001, 1, 1), (-73.41114990000008, 45.8178689200001, 0, 0), (-73.39620850000009, 45.8178689200001, 0, 0), (-73.38126710000009, 45.8178689200001, 0, 0), (-73.36632570000009, 45.8178689200001, 0, 0), (-73.35138430000009, 45.8178689200001, 0, 0), (-73.3364429000001, 45.8178689200001, 0, 0), (-73.3215015000001, 45.8178689200001, 0, 0), (-73.3065601000001, 45.8178689200001, 0, 0), (-73.2916187000001, 45.8178689200001, 0, 0), (-74.0237473, 45.828057640000104, 0, 0), (-74.0088059, 45.828057640000104, 0, 0), (-73.9938645, 45.828057640000104, 0, 0), (-73.9789231, 45.828057640000104, 0, 0), (-73.9639817, 45.828057640000104, 0, 0), (-73.94904030000001, 45.828057640000104, 0, 0), (-73.93409890000001, 45.828057640000104, 0, 0), (-73.91915750000001, 45.828057640000104, 0, 0), (-73.90421610000001, 45.828057640000104, 0, 0), (-73.88927470000002, 45.828057640000104, 0, 0), (-73.87433330000002, 45.828057640000104, 0, 0),

(-73.85939190000002, 45.828057640000104, 0, 0), (-73.84445050000002, 45.828057640000104, 0, 0), (-73.82950910000002, 45.828057640000104, 0, 0), (-73.81456770000003, 45.828057640000104, 0, 0), (-73.79962630000003, 45.828057640000104, 0, 0), (-73.78468490000003, 45.828057640000104, 0, 0), (-73.76974350000003, 45.828057640000104, 0, 0), (-73.75480210000003, 45.828057640000104, 0, 0), (-73.73986070000004, 45.828057640000104, 0, 0), (-73.72491930000004, 45.828057640000104, 0, 0), (-73.70997790000004, 45.828057640000104, 0, 0), (-73.69503650000004, 45.828057640000104, 0, 0), (-73.68009510000005, 45.828057640000104, 0, 0), (-73.66515370000005, 45.828057640000104, 0, 0), (-73.65021230000005, 45.828057640000104, 0, 0), (-73.63527090000005, 45.828057640000104, 0, 0), (-73.62032950000005, 45.828057640000104, 0, 0), (-73.60538810000006, 45.828057640000104, 0, 0), (-73.59044670000006, 45.828057640000104, 0, 0), (-73.57550530000006, 45.828057640000104, 0, 0), (-73.56056390000006, 45.828057640000104, 0, 0), (-73.54562250000006, 45.828057640000104, 0, 0), (-73.53068110000007, 45.828057640000104, 0, 0), (-73.51573970000007, 45.828057640000104, 0, 0), (-73.50079830000007, 45.828057640000104, 0, 0), (-73.48585690000007, 45.828057640000104, 0, 0), (-73.47091550000007, 45.828057640000104, 0, 0), (-73.45597410000008, 45.828057640000104, 0, 0), (-73.44103270000008, 45.828057640000104, 1, 3), (-73.42609130000008, 45.828057640000104, 8, 0), (-73.41114990000008, 45.828057640000104, 0, 0), (-73.39620850000009, 45.828057640000104, 0, 0), (-73.38126710000009, 45.828057640000104, 0, 0), (-73.36632570000009, 45.828057640000104, 0, 0), (-73.35138430000009, 45.828057640000104, 0, 0), (-73.3364429000001, 45.828057640000104, 0, 0), (-73.3215015000001, 45.828057640000104, 0, 0), (-73.3065601000001, 45.828057640000104, 0, 0), (-73.2916187000001, 45.828057640000104, 0, 0)]
