# Supplementary material for: Probing sociodemographic influence on code-switching and language choice in Quebec with geolocation of tweets
Source: Front Psychol. 2023 May 2;14:1137038. doi: 10.3389/fpsyg.2023.1137038 (PMC10187760; doi:10.3389/fpsyg.2023.1137038)
Supplement: Supplementary file 4 [file Data_Sheet_4.pdf]

Data_+/-CS-French per bin in GM, (longitude, latitude, +CS-French, -CS-French)
[(-74.0237473, 45.32881036, 0, 0), (-74.0088059, 45.32881036, 0, 0), (-73.9938645, 45.32881036, 0, 0), (-73.9789231, 45.32881036, 0, 0), (-73.9639817, 45.32881036, 0, 3), (-73.94904030000001, 45.32881036, 0, 0), (-73.93409890000001, 45.32881036, 0, 0), (-73.91915750000001, 45.32881036, 0, 0), (-73.90421610000001, 45.32881036, 0, 0), (-73.88927470000002, 45.32881036, 0, 0), (-73.87433330000002, 45.32881036, 0, 0), (-73.85939190000002, 45.32881036, 0, 0), (-73.84445050000002, 45.32881036, 0, 0), (-73.82950910000002, 45.32881036, 0, 0), (-73.81456770000003, 45.32881036, 0, 2), (-73.79962630000003, 45.32881036, 0, 0), (-73.78468490000003, 45.32881036, 0, 0), (-73.76974350000003, 45.32881036, 0, 0), (-73.75480210000003, 45.32881036, 0, 0), (-73.73986070000004, 45.32881036, 0, 0), (-73.72491930000004, 45.32881036, 0, 0), (-73.70997790000004, 45.32881036, 0, 0), (-73.69503650000004, 45.32881036, 0, 0), (-73.68009510000005, 45.32881036, 0, 0), (-73.66515370000005, 45.32881036, 0, 0), (-73.65021230000005, 45.32881036, 0, 0), (-73.63527090000005, 45.32881036, 0, 0), (-73.62032950000005, 45.32881036, 0, 0), (-73.60538810000006, 45.32881036, 0, 0), (-73.59044670000006, 45.32881036, 0, 0), (-73.57550530000006, 45.32881036, 0, 0), (-73.56056390000006, 45.32881036, 0, 0), (-73.54562250000006, 45.32881036, 0, 0), (-73.53068110000007, 45.32881036, 0, 0), (-73.51573970000007, 45.32881036, 0, 0), (-73.50079830000007, 45.32881036, 0, 0), (-73.48585690000007, 45.32881036, 0, 0), (-73.47091550000007, 45.32881036, 0, 0), (-73.45597410000008, 45.32881036, 0, 0), (-73.44103270000008, 45.32881036, 0, 0), (-73.42609130000008, 45.32881036, 0, 0), (-73.41114990000008, 45.32881036, 0, 0), (-73.39620850000009, 45.32881036, 0, 0), (-73.38126710000009, 45.32881036, 0, 0), (-73.36632570000009, 45.32881036, 0, 0), (-73.35138430000009, 45.32881036, 0, 0), (-73.3364429000001, 45.32881036, 0, 0), (-73.3215015000001, 45.32881036, 0, 0), (-73.3065601000001, 45.32881036, 0, 0), (-73.2916187000001, 45.32881036, 0, 9), (-74.0237473, 45.33899908, 0, 0), (-74.0088059, 45.33899908, 0, 0), (-73.9938645, 45.33899908, 0, 0), (-73.9789231, 45.33899908, 0, 2), (-73.9639817, 45.33899908, 0, 1), (-73.94904030000001, 45.33899908, 0, 0), (-73.93409890000001, 45.33899908, 0, 0), (-73.91915750000001, 45.33899908, 0, 0), (-73.90421610000001, 45.33899908, 0, 0), (-73.88927470000002, 45.33899908, 0, 0), (-73.87433330000002, 45.33899908, 0, 0), (-73.85939190000002, 45.33899908, 0, 0), (-73.84445050000002, 45.33899908, 0, 0), (-73.82950910000002, 45.33899908, 0, 0), (-73.81456770000003, 45.33899908, 0, 0), (-73.79962630000003, 45.33899908, 0, 0), (-73.78468490000003, 45.33899908, 0, 0), (-73.76974350000003, 45.33899908, 0, 2), (-73.75480210000003, 45.33899908, 0, 0), (-73.73986070000004, 45.33899908, 0, 0), (-73.72491930000004, 45.33899908, 0, 2), (-73.70997790000004, 45.33899908, 0, 0), (-73.69503650000004, 45.33899908, 0, 0), (-73.68009510000005, 45.33899908, 0, 0), (-73.66515370000005, 45.33899908, 0, 0), (-73.65021230000005, 45.33899908, 0, 0), (-73.63527090000005, 45.33899908, 0, 0), (-73.62032950000005, 45.33899908, 0, 0), (-73.60538810000006, 45.33899908, 0, 0), (-73.59044670000006, 45.33899908, 0, 0), (-73.57550530000006, 45.33899908, 0, 0), (-73.56056390000006, 45.33899908, 0, 0), (-73.54562250000006, 45.33899908, 0, 0), (-73.53068110000007, 45.33899908, 0, 0), (-73.51573970000007, 45.33899908, 0, 1), (-73.50079830000007, 45.33899908, 0, 0), (-73.48585690000007, 45.33899908, 0, 0), (-73.47091550000007, 45.33899908, 0, 0), (-73.45597410000008, 45.33899908, 0, 0), (-73.44103270000008, 45.33899908, 0, 0), (-73.42609130000008, 45.33899908, 0, 0), (-73.41114990000008, 45.33899908, 0, 0), (-73.39620850000009, 45.33899908, 0, 0), (-73.38126710000009, 45.33899908, 0, 0), (-73.36632570000009, 45.33899908, 0, 0), (-73.35138430000009, 45.33899908, 0, 0), (-73.3364429000001, 45.33899908, 0, 0), (-73.3215015000001, 45.33899908, 0, 0), (-73.3065601000001, 45.33899908, 0, 0), (-73.2916187000001, 45.33899908, 0, 0), (-74.0237473, 45.3491878, 0, 0), (-74.0088059, 45.3491878, 0, 0), (-73.9938645, 45.3491878, 0, 0), (-73.9789231, 45.3491878, 0, 0), (-73.9639817, 45.3491878, 0, 0), (-73.94904030000001,

45.3491878, 0, 0), (-73.93409890000001, 45.3491878, 0, 0), (-73.91915750000001, 45.3491878, 0, 0), (-73.90421610000001, 45.3491878, 0, 11), (-73.88927470000002, 45.3491878, 0, 0), (-73.87433330000002, 45.3491878, 0, 0), (-73.85939190000002, 45.3491878, 0, 0), (-73.84445050000002, 45.3491878, 0, 0), (-73.82950910000002, 45.3491878, 0, 0), (-73.81456770000003, 45.3491878, 0, 0), (-73.79962630000003, 45.3491878, 0, 0), (-73.78468490000003, 45.3491878, 0, 0), (-73.76974350000003, 45.3491878, 0, 0), (-73.75480210000003, 45.3491878, 0, 0), (-73.73986070000004, 45.3491878, 0, 1), (-73.72491930000004, 45.3491878, 0, 2), (-73.70997790000004, 45.3491878, 0, 1), (-73.69503650000004, 45.3491878, 0, 0), (-73.68009510000005, 45.3491878, 0, 0), (-73.66515370000005, 45.3491878, 0, 0), (-73.65021230000005, 45.3491878, 0, 0), (-73.63527090000005, 45.3491878, 0, 0), (-73.62032950000005, 45.3491878, 0, 0), (-73.60538810000006, 45.3491878, 0, 0), (-73.59044670000006, 45.3491878, 0, 0), (-73.57550530000006, 45.3491878, 0, 0), (-73.56056390000006, 45.3491878, 0, 0), (-73.54562250000006, 45.3491878, 0, 0), (-73.53068110000007, 45.3491878, 0, 0), (-73.51573970000007, 45.3491878, 0, 0), (-73.50079830000007, 45.3491878, 0, 0), (-73.48585690000007, 45.3491878, 0, 0), (-73.47091550000007, 45.3491878, 0, 1), (-73.45597410000008, 45.3491878, 0, 0), (-73.44103270000008, 45.3491878, 0, 0), (-73.42609130000008, 45.3491878, 0, 0), (-73.41114990000008, 45.3491878, 0, 0), (-73.39620850000009, 45.3491878, 0, 0), (-73.38126710000009, 45.3491878, 0, 0), (-73.36632570000009, 45.3491878, 0, 0), (-73.35138430000009, 45.3491878, 0, 0), (-73.3364429000001, 45.3491878, 0, 0), (-73.3215015000001, 45.3491878, 0, 0), (-73.3065601000001, 45.3491878, 0, 0), (-73.2916187000001, 45.3491878, 0, 0), (-74.0237473, 45.359376520000005, 0, 0), (-74.0088059, 45.359376520000005, 0, 0), (-73.9938645, 45.359376520000005, 0, 0), (-73.9789231, 45.359376520000005, 0, 0), (-73.9639817, 45.359376520000005, 0, 0), (-73.94904030000001, 45.359376520000005, 0, 0), (-73.93409890000001, 45.359376520000005, 0, 0), (-73.91915750000001, 45.359376520000005, 0, 11), (-73.90421610000001, 45.359376520000005, 0, 1), (-73.88927470000002, 45.359376520000005, 0, 0), (-73.87433330000002, 45.359376520000005, 0, 0), (-73.85939190000002, 45.359376520000005, 0, 0), (-73.84445050000002, 45.359376520000005, 0, 0), (-73.82950910000002, 45.359376520000005, 0, 0), (-73.81456770000003, 45.359376520000005, 0, 0), (-73.79962630000003, 45.359376520000005, 0, 0), (-73.78468490000003, 45.359376520000005, 0, 0), (-73.76974350000003, 45.359376520000005, 0, 0), (-73.75480210000003, 45.359376520000005, 2, 46), (-73.73986070000004, 45.359376520000005, 0, 0), (-73.72491930000004, 45.359376520000005, 0, 6), (-73.70997790000004, 45.359376520000005, 0, 8), (-73.69503650000004, 45.359376520000005, 0, 0), (-73.68009510000005, 45.359376520000005, 0, 0), (-73.66515370000005, 45.359376520000005, 0, 0), (-73.65021230000005, 45.359376520000005, 0, 0), (-73.63527090000005, 45.359376520000005, 0, 0), (-73.62032950000005, 45.359376520000005, 0, 0), (-73.60538810000006, 45.359376520000005, 0, 0), (-73.59044670000006, 45.359376520000005, 0, 0), (-73.57550530000006, 45.359376520000005, 0, 0), (-73.56056390000006, 45.359376520000005, 0, 0), (-73.54562250000006, 45.359376520000005, 0, 0), (-73.53068110000007, 45.359376520000005, 0, 0), (-73.51573970000007, 45.359376520000005, 0, 0), (-73.50079830000007, 45.359376520000005, 0, 0), (-73.48585690000007, 45.359376520000005, 0, 0), (-73.47091550000007, 45.359376520000005, 0, 0), (-73.45597410000008, 45.359376520000005, 0, 0), (-73.44103270000008, 45.359376520000005, 0, 0), (-73.42609130000008, 45.359376520000005, 0, 0), (-73.41114990000008, 45.359376520000005, 0, 0), (-73.39620850000009, 45.359376520000005, 0, 0), (-73.38126710000009, 45.359376520000005, 0, 0), (-73.36632570000009, 45.359376520000005, 0, 0), (-73.35138430000009, 45.359376520000005, 0, 0), (-73.3364429000001, 45.359376520000005, 0, 0), (-73.3215015000001, 45.359376520000005, 0, 0), (-73.3065601000001, 45.359376520000005, 0, 1), (-73.2916187000001, 45.359376520000005,

0, 0), (-74.0237473, 45.3695652400001, 0, 0), (-74.0088059, 45.3695652400001, 0, 0), (-73.9938645, 45.3695652400001, 0, 0), (-73.9789231, 45.3695652400001, 0, 69), (-73.9639817, 45.3695652400001, 0, 0), (-73.94904030000001, 45.3695652400001, 0, 0), (-73.93409890000001, 45.3695652400001, 1, 2), (-73.91915750000001, 45.3695652400001, 0, 0), (-73.90421610000001, 45.3695652400001, 0, 0), (-73.88927470000002, 45.3695652400001, 0, 0), (-73.87433330000002, 45.3695652400001, 0, 1), (-73.85939190000002, 45.3695652400001, 0, 4), (-73.84445050000002, 45.3695652400001, 1, 2), (-73.82950910000002, 45.3695652400001, 0, 0), (-73.81456770000003, 45.3695652400001, 0, 0), (-73.79962630000003, 45.3695652400001, 0, 0), (-73.78468490000003, 45.3695652400001, 0, 0), (-73.76974350000003, 45.3695652400001, 0, 0), (-73.75480210000003, 45.3695652400001, 0, 0), (-73.73986070000004, 45.3695652400001, 0, 0), (-73.72491930000004, 45.3695652400001, 0, 0), (-73.70997790000004, 45.3695652400001, 0, 1), (-73.69503650000004, 45.3695652400001, 0, 0), (-73.68009510000005, 45.3695652400001, 0, 0), (-73.66515370000005, 45.3695652400001, 0, 0), (-73.65021230000005, 45.3695652400001, 0, 1), (-73.63527090000005, 45.3695652400001, 0, 0), (-73.62032950000005, 45.3695652400001, 0, 0), (-73.60538810000006, 45.3695652400001, 0, 0), (-73.59044670000006, 45.3695652400001, 0, 0), (-73.57550530000006, 45.3695652400001, 2, 17), (-73.56056390000006, 45.3695652400001, 1, 7), (-73.54562250000006, 45.3695652400001, 0, 85), (-73.53068110000007, 45.3695652400001, 0, 2), (-73.51573970000007, 45.3695652400001, 0, 3), (-73.50079830000007, 45.3695652400001, 0, 1), (-73.48585690000007, 45.3695652400001, 0, 0), (-73.47091550000007, 45.3695652400001, 0, 0), (-73.45597410000008, 45.3695652400001, 0, 0), (-73.44103270000008, 45.3695652400001, 0, 0), (-73.42609130000008, 45.3695652400001, 0, 0), (-73.41114990000008, 45.3695652400001, 0, 0), (-73.39620850000009, 45.3695652400001, 0, 0), (-73.38126710000009, 45.3695652400001, 0, 0), (-73.36632570000009, 45.3695652400001, 0, 0), (-73.35138430000009, 45.3695652400001, 0, 0), (-73.3364429000001, 45.3695652400001, 0, 0), (-73.3215015000001, 45.3695652400001, 0, 0), (-73.3065601000001, 45.3695652400001, 0, 0), (-73.2916187000001, 45.3695652400001, 0, 0), (-74.0237473, 45.3797539600001, 0, 29), (-74.0088059, 45.3797539600001, 0, 34), (-73.9938645, 45.3797539600001, 0, 5), (-73.9789231, 45.3797539600001, 0, 0), (-73.9639817, 45.3797539600001, 0, 0), (-73.94904030000001, 45.3797539600001, 0, 7), (-73.93409890000001, 45.3797539600001, 0, 0), (-73.91915750000001, 45.3797539600001, 0, 0), (-73.90421610000001, 45.3797539600001, 0, 0), (-73.88927470000002, 45.3797539600001, 0, 0), (-73.87433330000002, 45.3797539600001, 0, 0), (-73.85939190000002, 45.3797539600001, 0, 0), (-73.84445050000002, 45.3797539600001, 0, 0), (-73.82950910000002, 45.3797539600001, 0, 0), (-73.81456770000003, 45.3797539600001, 0, 0), (-73.79962630000003, 45.3797539600001, 0, 0), (-73.78468490000003, 45.3797539600001, 0, 0), (-73.76974350000003, 45.3797539600001, 1, 1), (-73.75480210000003, 45.3797539600001, 0, 14), (-73.73986070000004, 45.3797539600001, 0, 0), (-73.72491930000004, 45.3797539600001, 0, 0), (-73.70997790000004, 45.3797539600001, 1, 9), (-73.69503650000004, 45.3797539600001, 0, 0), (-73.68009510000005, 45.3797539600001, 0, 0), (-73.66515370000005, 45.3797539600001, 0, 0), (-73.65021230000005, 45.3797539600001, 0, 0), (-73.63527090000005, 45.3797539600001, 0, 0), (-73.62032950000005, 45.3797539600001, 0, 0), (-73.60538810000006, 45.3797539600001, 0, 1), (-73.59044670000006, 45.3797539600001, 0, 0), (-73.57550530000006, 45.3797539600001, 0, 0), (-73.56056390000006, 45.3797539600001, 0, 19), (-73.54562250000006, 45.3797539600001, 0, 5), (-73.53068110000007, 45.3797539600001, 0, 3), (-73.51573970000007, 45.3797539600001, 3, 157), (-73.50079830000007, 45.3797539600001, 0, 2), (-73.48585690000007, 45.3797539600001, 0, 2), (-73.47091550000007, 45.3797539600001, 0, 0), (-73.45597410000008, 45.3797539600001, 0, 0), (-73.44103270000008, 45.3797539600001, 0, 0), (-73.42609130000008, 45.3797539600001, 0,

0), (-73.41114990000008, 45.37975396000001, 0, 0), (-73.39620850000009, 45.37975396000001, 0, 0), (-73.38126710000009, 45.37975396000001, 0, 0), (-73.36632570000009, 45.37975396000001, 0, 0), (-73.35138430000009, 45.37975396000001, 0, 0), (-73.3364429000001, 45.37975396000001, 0, 0), (-73.3215015000001, 45.37975396000001, 0, 0), (-73.3065601000001, 45.37975396000001, 0, 0), (-73.2916187000001, 45.37975396000001, 0, 0), (-74.0237473, 45.38994268000001, 0, 17), (-74.0088059, 45.38994268000001, 0, 6), (-73.9938645, 45.38994268000001, 0, 1), (-73.9789231, 45.38994268000001, 0, 6), (-73.9639817, 45.38994268000001, 0, 25), (-73.94904030000001, 45.38994268000001, 0, 11), (-73.93409890000001, 45.38994268000001, 0, 0), (-73.91915750000001, 45.38994268000001, 0, 0), (-73.90421610000001, 45.38994268000001, 0, 3), (-73.88927470000002, 45.38994268000001, 0, 0), (-73.87433330000002, 45.38994268000001, 0, 0), (-73.85939190000002, 45.38994268000001, 0, 0), (-73.84445050000002, 45.38994268000001, 0, 0), (-73.82950910000002, 45.38994268000001, 0, 0), (-73.81456770000003, 45.38994268000001, 0, 0), (-73.79962630000003, 45.38994268000001, 0, 0), (-73.78468490000003, 45.38994268000001, 0, 0), (-73.76974350000003, 45.38994268000001, 0, 0), (-73.75480210000003, 45.38994268000001, 0, 3), (-73.73986070000004, 45.38994268000001, 0, 0), (-73.72491930000004, 45.38994268000001, 0, 0), (-73.70997790000004, 45.38994268000001, 0, 0), (-73.69503650000004, 45.38994268000001, 0, 2), (-73.68009510000005, 45.38994268000001, 0, 0), (-73.66515370000005, 45.38994268000001, 0, 1), (-73.65021230000005, 45.38994268000001, 0, 0), (-73.63527090000005, 45.38994268000001, 0, 0), (-73.62032950000005, 45.38994268000001, 0, 0), (-73.60538810000006, 45.38994268000001, 0, 0), (-73.59044670000006, 45.38994268000001, 0, 1), (-73.57550530000006, 45.38994268000001, 0, 0), (-73.56056390000006, 45.38994268000001, 0, 2), (-73.54562250000006, 45.38994268000001, 0, 32), (-73.53068110000007, 45.38994268000001, 0, 0), (-73.51573970000007, 45.38994268000001, 0, 1), (-73.50079830000007, 45.38994268000001, 0, 0), (-73.48585690000007, 45.38994268000001, 0, 0), (-73.47091550000007, 45.38994268000001, 0, 0), (-73.45597410000008, 45.38994268000001, 0, 0), (-73.44103270000008, 45.38994268000001, 0, 0), (-73.42609130000008, 45.38994268000001, 0, 0), (-73.41114990000008, 45.38994268000001, 0, 0), (-73.39620850000009, 45.38994268000001, 0, 0), (-73.38126710000009, 45.38994268000001, 0, 0), (-73.36632570000009, 45.38994268000001, 0, 0), (-73.35138430000009, 45.38994268000001, 0, 0), (-73.3364429000001, 45.38994268000001, 0, 0), (-73.3215015000001, 45.38994268000001, 0, 0), (-73.3065601000001, 45.38994268000001, 0, 0), (-73.2916187000001, 45.38994268000001, 0, 0), (-74.0237473, 45.400131400000014, 0, 9), (-74.0088059, 45.400131400000014, 0, 0), (-73.9938645, 45.400131400000014, 0, 0), (-73.9789231, 45.400131400000014, 0, 0), (-73.9639817, 45.400131400000014, 1, 44), (-73.94904030000001, 45.400131400000014, 2, 117), (-73.93409890000001, 45.400131400000014, 0, 7), (-73.91915750000001, 45.400131400000014, 0, 5), (-73.90421610000001, 45.400131400000014, 0, 0), (-73.88927470000002, 45.400131400000014, 0, 0), (-73.87433330000002, 45.400131400000014, 0, 0), (-73.85939190000002, 45.400131400000014, 0, 0), (-73.84445050000002, 45.400131400000014, 0, 0), (-73.82950910000002, 45.400131400000014, 0, 0), (-73.81456770000003, 45.400131400000014, 0, 4), (-73.79962630000003, 45.400131400000014, 0, 0), (-73.78468490000003, 45.400131400000014, 0, 0), (-73.76974350000003, 45.400131400000014, 0, 0), (-73.75480210000003, 45.400131400000014, 0, 0), (-73.73986070000004, 45.400131400000014, 0, 0), (-73.72491930000004, 45.400131400000014, 0, 0), (-73.70997790000004, 45.400131400000014, 0, 0), (-73.69503650000004, 45.400131400000014, 0, 3), (-73.68009510000005, 45.400131400000014, 1, 18), (-73.66515370000005, 45.400131400000014, 0, 0), (-73.65021230000005, 45.400131400000014, 0, 0), (-73.63527090000005, 45.400131400000014, 0, 1), (-73.62032950000005, 45.400131400000014, 0, 1), (-73.60538810000006, 45.400131400000014, 0, 0), (-73.59044670000006, 45.400131400000014, 0, 6), (-73.57550530000006, 45.400131400000014, 0, 0),

(-73.56056390000006, 45.400131400000014, 0, 171), (-73.54562250000006, 45.400131400000014, 0, 1), (-73.53068110000007, 45.400131400000014, 0, 2), (-73.51573970000007, 45.400131400000014, 1, 72), (-73.50079830000007, 45.400131400000014, 0, 55), (-73.48585690000007, 45.400131400000014, 0, 2), (-73.47091550000007, 45.400131400000014, 0, 1), (-73.45597410000008, 45.400131400000014, 0, 0), (-73.44103270000008, 45.400131400000014, 0, 0), (-73.42609130000008, 45.400131400000014, 0, 0), (-73.41114990000008, 45.400131400000014, 0, 0), (-73.39620850000009, 45.400131400000014, 0, 0), (-73.38126710000009, 45.400131400000014, 0, 0), (-73.36632570000009, 45.400131400000014, 0, 0), (-73.35138430000009, 45.400131400000014, 0, 0), (-73.3364429000001, 45.400131400000014, 0, 0), (-73.3215015000001, 45.400131400000014, 0, 0), (-73.3065601000001, 45.400131400000014, 0, 0), (-73.2916187000001, 45.400131400000014, 0, 0), (-74.0237473, 45.410320120000016, 0, 3), (-74.0088059, 45.410320120000016, 0, 1), (-73.9938645, 45.410320120000016, 0, 0), (-73.9789231, 45.410320120000016, 0, 0), (-73.9639817, 45.410320120000016, 1, 8), (-73.94904030000001, 45.410320120000016, 1, 51), (-73.93409890000001, 45.410320120000016, 1, 17), (-73.91915750000001, 45.410320120000016, 0, 14), (-73.90421610000001, 45.410320120000016, 0, 1), (-73.88927470000002, 45.410320120000016, 0, 1), (-73.87433330000002, 45.410320120000016, 0, 0), (-73.85939190000002, 45.410320120000016, 0, 0), (-73.84445050000002, 45.410320120000016, 0, 0), (-73.82950910000002, 45.410320120000016, 0, 0), (-73.81456770000003, 45.410320120000016, 0, 0), (-73.79962630000003, 45.410320120000016, 0, 0), (-73.78468490000003, 45.410320120000016, 0, 0), (-73.76974350000003, 45.410320120000016, 0, 0), (-73.75480210000003, 45.410320120000016, 0, 0), (-73.73986070000004, 45.410320120000016, 0, 0), (-73.72491930000004, 45.410320120000016, 0, 0), (-73.70997790000004, 45.410320120000016, 0, 6), (-73.69503650000004, 45.410320120000016, 0, 1), (-73.68009510000005, 45.410320120000016, 0, 13), (-73.66515370000005, 45.410320120000016, 0, 7), (-73.65021230000005, 45.410320120000016, 0, 0), (-73.63527090000005, 45.410320120000016, 0, 0), (-73.62032950000005, 45.410320120000016, 0, 0), (-73.60538810000006, 45.410320120000016, 0, 0), (-73.59044670000006, 45.410320120000016, 0, 0), (-73.57550530000006, 45.410320120000016, 0, 1), (-73.56056390000006, 45.410320120000016, 0, 0), (-73.54562250000006, 45.410320120000016, 0, 0), (-73.53068110000007, 45.410320120000016, 0, 0), (-73.51573970000007, 45.410320120000016, 0, 0), (-73.50079830000007, 45.410320120000016, 0, 1), (-73.48585690000007, 45.410320120000016, 0, 0), (-73.47091550000007, 45.410320120000016, 0, 2), (-73.45597410000008, 45.410320120000016, 0, 0), (-73.44103270000008, 45.410320120000016, 0, 0), (-73.42609130000008, 45.410320120000016, 0, 0), (-73.41114990000008, 45.410320120000016, 0, 0), (-73.39620850000009, 45.410320120000016, 0, 0), (-73.38126710000009, 45.410320120000016, 0, 0), (-73.36632570000009, 45.410320120000016, 0, 0), (-73.35138430000009, 45.410320120000016, 0, 0), (-73.3364429000001, 45.410320120000016, 0, 0), (-73.3215015000001, 45.410320120000016, 0, 0), (-73.3065601000001, 45.410320120000016, 0, 1), (-73.2916187000001, 45.410320120000016, 0, 1), (-74.0237473, 45.42050884000002, 0, 10), (-74.0088059, 45.42050884000002, 0, 5), (-73.9938645, 45.42050884000002, 0, 0), (-73.9789231, 45.42050884000002, 0, 0), (-73.9639817, 45.42050884000002, 0, 0), (-73.94904030000001, 45.42050884000002, 0, 0), (-73.93409890000001, 45.42050884000002, 0, 12), (-73.91915750000001, 45.42050884000002, 1, 84), (-73.90421610000001, 45.42050884000002, 0, 0), (-73.88927470000002, 45.42050884000002, 1, 62), (-73.87433330000002, 45.42050884000002, 2, 291), (-73.85939190000002, 45.42050884000002, 1, 77), (-73.84445050000002, 45.42050884000002, 0, 0), (-73.82950910000002, 45.42050884000002, 0, 1), (-73.81456770000003, 45.42050884000002, 0, 0), (-73.79962630000003, 45.42050884000002, 0, 0), (-73.78468490000003, 45.42050884000002, 0, 0), (-73.76974350000003, 45.42050884000002, 0, 0), (-73.75480210000003, 45.42050884000002, 0, 0),

(-73.73986070000004, 45.42050884000002, 0, 0), (-73.72491930000004, 45.42050884000002, 0, 0), (-73.70997790000004, 45.42050884000002, 0, 0), (-73.69503650000004, 45.42050884000002, 0, 0), (-73.68009510000005, 45.42050884000002, 0, 0), (-73.66515370000005, 45.42050884000002, 0, 0), (-73.65021230000005, 45.42050884000002, 3, 209), (-73.63527090000005, 45.42050884000002, 0, 37), (-73.62032950000005, 45.42050884000002, 0, 1), (-73.60538810000006, 45.42050884000002, 0, 5), (-73.59044670000006, 45.42050884000002, 0, 0), (-73.57550530000006, 45.42050884000002, 0, 0), (-73.56056390000006, 45.42050884000002, 0, 0), (-73.54562250000006, 45.42050884000002, 0, 0), (-73.53068110000007, 45.42050884000002, 0, 0), (-73.51573970000007, 45.42050884000002, 0, 0), (-73.50079830000007, 45.42050884000002, 0, 8), (-73.48585690000007, 45.42050884000002, 1, 73), (-73.47091550000007, 45.42050884000002, 0, 3), (-73.45597410000008, 45.42050884000002, 0, 0), (-73.44103270000008, 45.42050884000002, 0, 0), (-73.42609130000008, 45.42050884000002, 0, 0), (-73.41114990000008, 45.42050884000002, 0, 0), (-73.39620850000009, 45.42050884000002, 0, 0), (-73.38126710000009, 45.42050884000002, 0, 0), (-73.36632570000009, 45.42050884000002, 0, 0), (-73.35138430000009, 45.42050884000002, 0, 0), (-73.3364429000001, 45.42050884000002, 0, 0), (-73.3215015000001, 45.42050884000002, 0, 0), (-73.3065601000001, 45.42050884000002, 0, 0), (-73.2916187000001, 45.42050884000002, 0, 0), (-74.0237473, 45.43069756000002, 0, 0), (-74.0088059, 45.43069756000002, 0, 0), (-73.9938645, 45.43069756000002, 0, 0), (-73.9789231, 45.43069756000002, 0, 0), (-73.9639817, 45.43069756000002, 0, 1), (-73.94904030000001, 45.43069756000002, 0, 1), (-73.93409890000001, 45.43069756000002, 0, 5), (-73.91915750000001, 45.43069756000002, 0, 2), (-73.90421610000001, 45.43069756000002, 0, 0), (-73.88927470000002, 45.43069756000002, 0, 2), (-73.87433330000002, 45.43069756000002, 1, 1479), (-73.85939190000002, 45.43069756000002, 0, 9), (-73.84445050000002, 45.43069756000002, 0, 21), (-73.82950910000002, 45.43069756000002, 3, 87), (-73.81456770000003, 45.43069756000002, 0, 6), (-73.79962630000003, 45.43069756000002, 0, 0), (-73.78468490000003, 45.43069756000002, 0, 0), (-73.76974350000003, 45.43069756000002, 0, 0), (-73.75480210000003, 45.43069756000002, 0, 85), (-73.73986070000004, 45.43069756000002, 0, 0), (-73.72491930000004, 45.43069756000002, 0, 0), (-73.70997790000004, 45.43069756000002, 0, 1), (-73.69503650000004, 45.43069756000002, 0, 15), (-73.68009510000005, 45.43069756000002, 3, 51), (-73.66515370000005, 45.43069756000002, 0, 43), (-73.65021230000005, 45.43069756000002, 1, 11), (-73.63527090000005, 45.43069756000002, 10, 150), (-73.62032950000005, 45.43069756000002, 2, 86), (-73.60538810000006, 45.43069756000002, 0, 17), (-73.59044670000006, 45.43069756000002, 0, 102), (-73.57550530000006, 45.43069756000002, 0, 1), (-73.56056390000006, 45.43069756000002, 0, 0), (-73.54562250000006, 45.43069756000002, 0, 0), (-73.53068110000007, 45.43069756000002, 0, 0), (-73.51573970000007, 45.43069756000002, 0, 0), (-73.50079830000007, 45.43069756000002, 0, 0), (-73.48585690000007, 45.43069756000002, 0, 1), (-73.47091550000007, 45.43069756000002, 0, 0), (-73.45597410000008, 45.43069756000002, 0, 315), (-73.44103270000008, 45.43069756000002, 0, 5), (-73.42609130000008, 45.43069756000002, 0, 0), (-73.41114990000008, 45.43069756000002, 0, 0), (-73.39620850000009, 45.43069756000002, 0, 0), (-73.38126710000009, 45.43069756000002, 0, 0), (-73.36632570000009, 45.43069756000002, 0, 0), (-73.35138430000009, 45.43069756000002, 0, 0), (-73.3364429000001, 45.43069756000002, 0, 0), (-73.3215015000001, 45.43069756000002, 1, 2), (-73.3065601000001, 45.43069756000002, 0, 9), (-73.2916187000001, 45.43069756000002, 0, 10), (-74.0237473, 45.44088628000002, 0, 0), (-74.0088059, 45.44088628000002, 0, 0), (-73.9938645, 45.44088628000002, 0, 0), (-73.9789231, 45.44088628000002, 0, 0), (-73.9639817, 45.44088628000002, 0, 0), (-73.94904030000001, 45.44088628000002, 0, 0), (-73.93409890000001, 45.44088628000002, 0, 0), (-73.91915750000001, 45.44088628000002, 0, 0), (-73.90421610000001, 45.44088628000002, 0, 0), (-73.88927470000002, 45.44088628000002, 0, 16), (-73.87433330000002,

45.44088628000002, 0, 1), (-73.85939190000002, 45.44088628000002, 0, 2), (-73.84445050000002, 45.44088628000002, 0, 0), (-73.82950910000002, 45.44088628000002, 0, 4), (-73.81456770000003, 45.44088628000002, 7, 44), (-73.79962630000003, 45.44088628000002, 0, 7), (-73.78468490000003, 45.44088628000002, 0, 2), (-73.76974350000003, 45.44088628000002, 0, 53), (-73.75480210000003, 45.44088628000002, 1, 18), (-73.73986070000004, 45.44088628000002, 0, 37), (-73.72491930000004, 45.44088628000002, 1, 534), (-73.70997790000004, 45.44088628000002, 0, 0), (-73.69503650000004, 45.44088628000002, 3, 1047), (-73.68009510000005, 45.44088628000002, 1, 30), (-73.66515370000005, 45.44088628000002, 0, 13), (-73.65021230000005, 45.44088628000002, 0, 7), (-73.63527090000005, 45.44088628000002, 0, 117), (-73.62032950000005, 45.44088628000002, 0, 15), (-73.60538810000006, 45.44088628000002, 0, 23), (-73.59044670000006, 45.44088628000002, 0, 2), (-73.57550530000006, 45.44088628000002, 0, 1), (-73.56056390000006, 45.44088628000002, 0, 0), (-73.54562250000006, 45.44088628000002, 0, 0), (-73.53068110000007, 45.44088628000002, 0, 0), (-73.51573970000007, 45.44088628000002, 0, 23), (-73.50079830000007, 45.44088628000002, 0, 0), (-73.48585690000007, 45.44088628000002, 0, 0), (-73.47091550000007, 45.44088628000002, 0, 4), (-73.45597410000008, 45.44088628000002, 0, 3), (-73.44103270000008, 45.44088628000002, 1, 154), (-73.42609130000008, 45.44088628000002, 0, 9), (-73.41114990000008, 45.44088628000002, 0, 0), (-73.39620850000009, 45.44088628000002, 0, 0), (-73.38126710000009, 45.44088628000002, 0, 0), (-73.36632570000009, 45.44088628000002, 0, 0), (-73.35138430000009, 45.44088628000002, 0, 0), (-73.3364429000001, 45.44088628000002, 0, 0), (-73.3215015000001, 45.44088628000002, 0, 0), (-73.3065601000001, 45.44088628000002, 0, 0), (-73.2916187000001, 45.44088628000002, 3, 153), (-74.0237473, 45.451075000000024, 1, 2), (-74.0088059, 45.451075000000024, 0, 0), (-73.9938645, 45.451075000000024, 0, 3), (-73.9789231, 45.451075000000024, 0, 0), (-73.9639817, 45.451075000000024, 0, 0), (-73.94904030000001, 45.451075000000024, 0, 0), (-73.93409890000001, 45.451075000000024, 0, 1), (-73.91915750000001, 45.451075000000024, 0, 0), (-73.90421610000001, 45.451075000000024, 0, 0), (-73.88927470000002, 45.451075000000024, 0, 5), (-73.87433330000002, 45.451075000000024, 0, 0), (-73.85939190000002, 45.451075000000024, 3, 549), (-73.84445050000002, 45.451075000000024, 0, 42), (-73.82950910000002, 45.451075000000024, 1, 7), (-73.81456770000003, 45.451075000000024, 4, 289), (-73.79962630000003, 45.451075000000024, 0, 9), (-73.78468490000003, 45.451075000000024, 4, 16), (-73.76974350000003, 45.451075000000024, 0, 4), (-73.75480210000003, 45.451075000000024, 7, 1203), (-73.73986070000004, 45.451075000000024, 1, 21), (-73.72491930000004, 45.451075000000024, 0, 1), (-73.70997790000004, 45.451075000000024, 0, 28), (-73.69503650000004, 45.451075000000024, 1, 32), (-73.68009510000005, 45.451075000000024, 3, 157), (-73.66515370000005, 45.451075000000024, 0, 3), (-73.65021230000005, 45.451075000000024, 2, 86), (-73.63527090000005, 45.451075000000024, 0, 8), (-73.62032950000005, 45.451075000000024, 3, 23), (-73.60538810000006, 45.451075000000024, 1, 125), (-73.59044670000006, 45.451075000000024, 2, 142), (-73.57550530000006, 45.451075000000024, 6, 178), (-73.56056390000006, 45.451075000000024, 2, 70), (-73.54562250000006, 45.451075000000024, 1, 26), (-73.53068110000007, 45.451075000000024, 0, 0), (-73.51573970000007, 45.451075000000024, 0, 0), (-73.50079830000007, 45.451075000000024, 0, 1), (-73.48585690000007, 45.451075000000024, 0, 34), (-73.47091550000007, 45.451075000000024, 3, 169), (-73.45597410000008, 45.451075000000024, 41, 1897), (-73.44103270000008, 45.451075000000024, 2, 248), (-73.42609130000008, 45.451075000000024, 0, 1), (-73.41114990000008, 45.451075000000024, 0, 0), (-73.39620850000009, 45.451075000000024, 0, 0), (-73.38126710000009, 45.451075000000024, 0, 9), (-73.36632570000009, 45.451075000000024, 0, 0), (-73.35138430000009, 45.451075000000024, 0, 0), (-73.3364429000001, 45.451075000000024, 0,

17), (-73.3215015000001, 45.451075000000024, 0, 0), (-73.3065601000001, 45.451075000000024, 7, 86), (-73.2916187000001, 45.451075000000024, 2, 7), (-74.0237473, 45.46126372000003, 0, 0), (-74.0088059, 45.46126372000003, 0, 0), (-73.9938645, 45.46126372000003, 0, 0), (-73.9789231, 45.46126372000003, 0, 0), (-73.9639817, 45.46126372000003, 0, 0), (-73.94904030000001, 45.46126372000003, 0, 1), (-73.93409890000001, 45.46126372000003, 0, 1), (-73.91915750000001, 45.46126372000003, 0, 0), (-73.90421610000001, 45.46126372000003, 0, 0), (-73.88927470000002, 45.46126372000003, 0, 6), (-73.87433330000002, 45.46126372000003, 0, 9), (-73.85939190000002, 45.46126372000003, 0, 22), (-73.84445050000002, 45.46126372000003, 0, 1), (-73.82950910000002, 45.46126372000003, 3, 196), (-73.81456770000003, 45.46126372000003, 0, 10), (-73.79962630000003, 45.46126372000003, 0, 3), (-73.78468490000003, 45.46126372000003, 0, 2), (-73.76974350000003, 45.46126372000003, 0, 2), (-73.75480210000003, 45.46126372000003, 2, 176), (-73.73986070000004, 45.46126372000003, 1, 34), (-73.72491930000004, 45.46126372000003, 0, 7), (-73.70997790000004, 45.46126372000003, 0, 289), (-73.69503650000004, 45.46126372000003, 0, 0), (-73.68009510000005, 45.46126372000003, 0, 2), (-73.66515370000005, 45.46126372000003, 0, 43), (-73.65021230000005, 45.46126372000003, 0, 7), (-73.63527090000005, 45.46126372000003, 2, 134), (-73.62032950000005, 45.46126372000003, 2, 297), (-73.60538810000006, 45.46126372000003, 0, 18), (-73.59044670000006, 45.46126372000003, 5, 72), (-73.57550530000006, 45.46126372000003, 0, 49), (-73.56056390000006, 45.46126372000003, 8, 210), (-73.54562250000006, 45.46126372000003, 4, 1373), (-73.53068110000007, 45.46126372000003, 0, 0), (-73.51573970000007, 45.46126372000003, 0, 0), (-73.50079830000007, 45.46126372000003, 0, 1), (-73.48585690000007, 45.46126372000003, 0, 3), (-73.47091550000007, 45.46126372000003, 2, 23), (-73.45597410000008, 45.46126372000003, 0, 0), (-73.44103270000008, 45.46126372000003, 0, 1), (-73.42609130000008, 45.46126372000003, 0, 6), (-73.41114990000008, 45.46126372000003, 0, 0), (-73.39620850000009, 45.46126372000003, 0, 0), (-73.38126710000009, 45.46126372000003, 0, 0), (-73.36632570000009, 45.46126372000003, 0, 0), (-73.35138430000009, 45.46126372000003, 1, 4), (-73.3364429000001, 45.46126372000003, 0, 9), (-73.3215015000001, 45.46126372000003, 0, 0), (-73.3065601000001, 45.46126372000003, 0, 6), (-73.2916187000001, 45.46126372000003, 0, 4), (-74.0237473, 45.47145244000003, 0, 0), (-74.0088059, 45.47145244000003, 0, 0), (-73.9938645, 45.47145244000003, 0, 0), (-73.9789231, 45.47145244000003, 0, 0), (-73.9639817, 45.47145244000003, 0, 0), (-73.94904030000001, 45.47145244000003, 0, 0), (-73.93409890000001, 45.47145244000003, 0, 6), (-73.91915750000001, 45.47145244000003, 0, 0), (-73.90421610000001, 45.47145244000003, 0, 0), (-73.88927470000002, 45.47145244000003, 1, 169), (-73.87433330000002, 45.47145244000003, 0, 16), (-73.85939190000002, 45.47145244000003, 0, 12), (-73.84445050000002, 45.47145244000003, 0, 3), (-73.82950910000002, 45.47145244000003, 1, 302), (-73.81456770000003, 45.47145244000003, 0, 8), (-73.79962630000003, 45.47145244000003, 70, 1198), (-73.78468490000003, 45.47145244000003, 0, 200), (-73.76974350000003, 45.47145244000003, 0, 4), (-73.75480210000003, 45.47145244000003, 0, 0), (-73.73986070000004, 45.47145244000003, 0, 7), (-73.72491930000004, 45.47145244000003, 0, 2), (-73.70997790000004, 45.47145244000003, 0, 8), (-73.69503650000004, 45.47145244000003, 0, 0), (-73.68009510000005, 45.47145244000003, 0, 51), (-73.66515370000005, 45.47145244000003, 1, 135), (-73.65021230000005, 45.47145244000003, 6, 331), (-73.63527090000005, 45.47145244000003, 0, 30), (-73.62032950000005, 45.47145244000003, 11, 599), (-73.60538810000006, 45.47145244000003, 7, 164), (-73.59044670000006, 45.47145244000003, 8, 381), (-73.57550530000006, 45.47145244000003, 2, 181), (-73.56056390000006, 45.47145244000003, 2, 50), (-73.54562250000006, 45.47145244000003, 0, 21), (-73.53068110000007, 45.47145244000003, 0, 4), (-73.51573970000007, 45.47145244000003, 0, 12), (-73.50079830000007, 45.47145244000003, 0,

1), (-73.48585690000007, 45.47145244000003, 0, 6), (-73.47091550000007, 45.47145244000003, 0, 92), (-73.45597410000008, 45.47145244000003, 0, 0), (-73.44103270000008, 45.47145244000003, 0, 7), (-73.42609130000008, 45.47145244000003, 0, 2), (-73.41114990000008, 45.47145244000003, 0, 0), (-73.39620850000009, 45.47145244000003, 0, 0), (-73.38126710000009, 45.47145244000003, 0, 0), (-73.36632570000009, 45.47145244000003, 0, 0), (-73.35138430000009, 45.47145244000003, 0, 0), (-73.3364429000001, 45.47145244000003, 0, 0), (-73.3215015000001, 45.47145244000003, 0, 0), (-73.3065601000001, 45.47145244000003, 0, 0), (-73.2916187000001, 45.47145244000003, 0, 0), (-74.0237473, 45.48164116000003, 0, 3), (-74.0088059, 45.48164116000003, 0, 0), (-73.9938645, 45.48164116000003, 0, 0), (-73.9789231, 45.48164116000003, 0, 0), (-73.9639817, 45.48164116000003, 0, 0), (-73.94904030000001, 45.48164116000003, 0, 0), (-73.93409890000001, 45.48164116000003, 0, 0), (-73.91915750000001, 45.48164116000003, 0, 5), (-73.90421610000001, 45.48164116000003, 0, 21), (-73.88927470000002, 45.48164116000003, 0, 0), (-73.87433330000002, 45.48164116000003, 2, 39), (-73.85939190000002, 45.48164116000003, 0, 10), (-73.84445050000002, 45.48164116000003, 0, 8), (-73.82950910000002, 45.48164116000003, 0, 0), (-73.81456770000003, 45.48164116000003, 0, 51), (-73.79962630000003, 45.48164116000003, 6, 702), (-73.78468490000003, 45.48164116000003, 13, 289), (-73.76974350000003, 45.48164116000003, 0, 1), (-73.75480210000003, 45.48164116000003, 0, 0), (-73.73986070000004, 45.48164116000003, 0, 0), (-73.72491930000004, 45.48164116000003, 1, 0), (-73.70997790000004, 45.48164116000003, 1, 176), (-73.69503650000004, 45.48164116000003, 0, 41), (-73.68009510000005, 45.48164116000003, 0, 0), (-73.66515370000005, 45.48164116000003, 0, 4), (-73.65021230000005, 45.48164116000003, 1, 21), (-73.63527090000005, 45.48164116000003, 2, 285), (-73.62032950000005, 45.48164116000003, 5, 457), (-73.60538810000006, 45.48164116000003, 13, 864), (-73.59044670000006, 45.48164116000003, 20, 977), (-73.57550530000006, 45.48164116000003, 13, 777), (-73.56056390000006, 45.48164116000003, 7, 648), (-73.54562250000006, 45.48164116000003, 1, 26), (-73.53068110000007, 45.48164116000003, 0, 0), (-73.51573970000007, 45.48164116000003, 0, 0), (-73.50079830000007, 45.48164116000003, 0, 2), (-73.48585690000007, 45.48164116000003, 2, 50), (-73.47091550000007, 45.48164116000003, 0, 4), (-73.45597410000008, 45.48164116000003, 1, 8), (-73.44103270000008, 45.48164116000003, 0, 1), (-73.42609130000008, 45.48164116000003, 0, 1), (-73.41114990000008, 45.48164116000003, 0, 4), (-73.39620850000009, 45.48164116000003, 0, 12), (-73.38126710000009, 45.48164116000003, 33, 185), (-73.36632570000009, 45.48164116000003, 0, 0), (-73.35138430000009, 45.48164116000003, 0, 0), (-73.3364429000001, 45.48164116000003, 0, 0), (-73.3215015000001, 45.48164116000003, 0, 0), (-73.3065601000001, 45.48164116000003, 0, 0), (-73.2916187000001, 45.48164116000003, 0, 0), (-74.0237473, 45.49182988000003, 1, 4), (-74.0088059, 45.49182988000003, 0, 0), (-73.9938645, 45.49182988000003, 0, 0), (-73.9789231, 45.49182988000003, 0, 0), (-73.9639817, 45.49182988000003, 0, 0), (-73.94904030000001, 45.49182988000003, 0, 0), (-73.93409890000001, 45.49182988000003, 0, 0), (-73.91915750000001, 45.49182988000003, 0, 0), (-73.90421610000001, 45.49182988000003, 0, 5), (-73.88927470000002, 45.49182988000003, 0, 0), (-73.87433330000002, 45.49182988000003, 0, 10), (-73.85939190000002, 45.49182988000003, 1, 4), (-73.84445050000002, 45.49182988000003, 1, 57), (-73.82950910000002, 45.49182988000003, 0, 7), (-73.81456770000003, 45.49182988000003, 7, 1012), (-73.79962630000003, 45.49182988000003, 0, 19), (-73.78468490000003, 45.49182988000003, 0, 1), (-73.76974350000003, 45.49182988000003, 0, 3), (-73.75480210000003, 45.49182988000003, 2, 19), (-73.73986070000004, 45.49182988000003, 0, 28), (-73.72491930000004, 45.49182988000003, 1, 11), (-73.70997790000004, 45.49182988000003, 1, 53), (-73.69503650000004, 45.49182988000003, 0, 18), (-73.68009510000005, 45.49182988000003, 2, 36), (-73.66515370000005, 45.49182988000003, 3, 506), (-73.65021230000005, 45.49182988000003,

26, 283), (-73.63527090000005, 45.49182988000003, 0, 123), (-73.62032950000005, 45.49182988000003, 6, 414), (-73.60538810000006, 45.49182988000003, 5, 137), (-73.59044670000006, 45.49182988000003, 7, 390), (-73.57550530000006, 45.49182988000003, 40, 2287), (-73.56056390000006, 45.49182988000003, 12, 444), (-73.54562250000006, 45.49182988000003, 1, 12), (-73.53068110000007, 45.49182988000003, 0, 10), (-73.51573970000007, 45.49182988000003, 0, 1), (-73.50079830000007, 45.49182988000003, 1, 75), (-73.48585690000007, 45.49182988000003, 0, 7), (-73.47091550000007, 45.49182988000003, 0, 31), (-73.45597410000008, 45.49182988000003, 0, 11), (-73.44103270000008, 45.49182988000003, 0, 1), (-73.42609130000008, 45.49182988000003, 0, 1), (-73.41114990000008, 45.49182988000003, 0, 23), (-73.39620850000009, 45.49182988000003, 0, 7), (-73.38126710000009, 45.49182988000003, 0, 51), (-73.36632570000009, 45.49182988000003, 0, 0), (-73.35138430000009, 45.49182988000003, 0, 1), (-73.3364429000001, 45.49182988000003, 0, 0), (-73.3215015000001, 45.49182988000003, 0, 0), (-73.3065601000001, 45.49182988000003, 0, 0), (-73.2916187000001, 45.49182988000003, 0, 0), (-74.0237473, 45.502018600000035, 0, 0), (-74.0088059, 45.502018600000035, 0, 0), (-73.9938645, 45.502018600000035, 0, 0), (-73.9789231, 45.502018600000035, 0, 1), (-73.9639817, 45.502018600000035, 1, 34), (-73.94904030000001, 45.502018600000035, 0, 0), (-73.93409890000001, 45.502018600000035, 0, 0), (-73.91915750000001, 45.502018600000035, 0, 0), (-73.90421610000001, 45.502018600000035, 0, 9), (-73.88927470000002, 45.502018600000035, 0, 0), (-73.87433330000002, 45.502018600000035, 0, 0), (-73.85939190000002, 45.502018600000035, 0, 0), (-73.84445050000002, 45.502018600000035, 0, 7), (-73.82950910000002, 45.502018600000035, 0, 41), (-73.81456770000003, 45.502018600000035, 0, 7), (-73.79962630000003, 45.502018600000035, 0, 7), (-73.78468490000003, 45.502018600000035, 0, 14), (-73.76974350000003, 45.502018600000035, 0, 12), (-73.75480210000003, 45.502018600000035, 18, 587), (-73.73986070000004, 45.502018600000035, 0, 1), (-73.72491930000004, 45.502018600000035, 1, 28), (-73.70997790000004, 45.502018600000035, 2, 80), (-73.69503650000004, 45.502018600000035, 0, 16), (-73.68009510000005, 45.502018600000035, 0, 14), (-73.66515370000005, 45.502018600000035, 1, 59), (-73.65021230000005, 45.502018600000035, 28, 1351), (-73.63527090000005, 45.502018600000035, 1, 73), (-73.62032950000005, 45.502018600000035, 2, 140), (-73.60538810000006, 45.502018600000035, 5, 142), (-73.59044670000006, 45.502018600000035, 60, 3132), (-73.57550530000006, 45.502018600000035, 508, 22890), (-73.56056390000006, 45.502018600000035, 626, 26716), (-73.54562250000006, 45.502018600000035, 48, 425), (-73.53068110000007, 45.502018600000035, 4, 158), (-73.51573970000007, 45.502018600000035, 8, 161), (-73.50079830000007, 45.502018600000035, 0, 3), (-73.48585690000007, 45.502018600000035, 0, 0), (-73.47091550000007, 45.502018600000035, 0, 1), (-73.45597410000008, 45.502018600000035, 0, 0), (-73.44103270000008, 45.502018600000035, 0, 81), (-73.42609130000008, 45.502018600000035, 0, 29), (-73.41114990000008, 45.502018600000035, 17, 606), (-73.39620850000009, 45.502018600000035, 0, 0), (-73.38126710000009, 45.502018600000035, 1, 86), (-73.36632570000009, 45.502018600000035, 0, 0), (-73.35138430000009, 45.502018600000035, 0, 0), (-73.3364429000001, 45.502018600000035, 0, 0), (-73.3215015000001, 45.502018600000035, 0, 0), (-73.3065601000001, 45.502018600000035, 0, 0), (-73.2916187000001, 45.502018600000035, 0, 0), (-74.0237473, 45.51220732000004, 0, 0), (-74.0088059, 45.51220732000004, 0, 0), (-73.9938645, 45.51220732000004, 0, 0), (-73.9789231, 45.51220732000004, 0, 0), (-73.9639817, 45.51220732000004, 0, 0), (-73.94904030000001, 45.51220732000004, 0, 0), (-73.93409890000001, 45.51220732000004, 0, 0), (-73.91915750000001, 45.51220732000004, 0, 0), (-73.90421610000001, 45.51220732000004, 0, 0), (-73.88927470000002, 45.51220732000004, 0, 2), (-73.87433330000002, 45.51220732000004, 0, 0), (-73.85939190000002, 45.51220732000004, 0, 0), (-73.84445050000002, 45.51220732000004, 0, 0), (-73.82950910000002, 45.51220732000004, 0, 0),

(-73.81456770000003, 45.51220732000004, 0, 27), (-73.79962630000003, 45.51220732000004, 0, 1), (-73.78468490000003, 45.51220732000004, 0, 1), (-73.76974350000003, 45.51220732000004, 0, 0), (-73.75480210000003, 45.51220732000004, 0, 1), (-73.73986070000004, 45.51220732000004, 0, 7), (-73.72491930000004, 45.51220732000004, 1, 14), (-73.70997790000004, 45.51220732000004, 2, 56), (-73.69503650000004, 45.51220732000004, 3, 197), (-73.68009510000005, 45.51220732000004, 8, 106), (-73.66515370000005, 45.51220732000004, 5, 188), (-73.65021230000005, 45.51220732000004, 10, 324), (-73.63527090000005, 45.51220732000004, 5, 44), (-73.62032950000005, 45.51220732000004, 5, 1285), (-73.60538810000006, 45.51220732000004, 4, 56), (-73.59044670000006, 45.51220732000004, 25, 2854), (-73.57550530000006, 45.51220732000004, 35, 1335), (-73.56056390000006, 45.51220732000004, 97, 3692), (-73.54562250000006, 45.51220732000004, 10, 442), (-73.53068110000007, 45.51220732000004, 17, 219), (-73.51573970000007, 45.51220732000004, 0, 42), (-73.50079830000007, 45.51220732000004, 0, 2), (-73.48585690000007, 45.51220732000004, 0, 2), (-73.47091550000007, 45.51220732000004, 0, 0), (-73.45597410000008, 45.51220732000004, 0, 5), (-73.44103270000008, 45.51220732000004, 0, 3), (-73.42609130000008, 45.51220732000004, 1, 7), (-73.41114990000008, 45.51220732000004, 0, 1), (-73.39620850000009, 45.51220732000004, 0, 1), (-73.38126710000009, 45.51220732000004, 1, 5), (-73.36632570000009, 45.51220732000004, 0, 0), (-73.35138430000009, 45.51220732000004, 0, 0), (-73.3364429000001, 45.51220732000004, 0, 0), (-73.3215015000001, 45.51220732000004, 0, 0), (-73.3065601000001, 45.51220732000004, 0, 0), (-73.2916187000001, 45.51220732000004, 0, 0), (-74.0237473, 45.52239604000004, 0, 0), (-74.0088059, 45.52239604000004, 0, 0), (-73.9938645, 45.52239604000004, 0, 1), (-73.9789231, 45.52239604000004, 0, 0), (-73.9639817, 45.52239604000004, 0, 0), (-73.94904030000001, 45.52239604000004, 0, 1), (-73.93409890000001, 45.52239604000004, 0, 0), (-73.91915750000001, 45.52239604000004, 0, 0), (-73.90421610000001, 45.52239604000004, 0, 0), (-73.88927470000002, 45.52239604000004, 0, 0), (-73.87433330000002, 45.52239604000004, 0, 1), (-73.85939190000002, 45.52239604000004, 0, 3), (-73.84445050000002, 45.52239604000004, 1, 4), (-73.82950910000002, 45.52239604000004, 0, 0), (-73.81456770000003, 45.52239604000004, 0, 0), (-73.79962630000003, 45.52239604000004, 0, 5), (-73.78468490000003, 45.52239604000004, 0, 12), (-73.76974350000003, 45.52239604000004, 0, 3), (-73.75480210000003, 45.52239604000004, 0, 1), (-73.73986070000004, 45.52239604000004, 0, 16), (-73.72491930000004, 45.52239604000004, 0, 11), (-73.70997790000004, 45.52239604000004, 2, 30), (-73.69503650000004, 45.52239604000004, 0, 37), (-73.68009510000005, 45.52239604000004, 0, 66), (-73.66515370000005, 45.52239604000004, 0, 20), (-73.65021230000005, 45.52239604000004, 0, 6), (-73.63527090000005, 45.52239604000004, 0, 2), (-73.62032950000005, 45.52239604000004, 5, 166), (-73.60538810000006, 45.52239604000004, 16, 858), (-73.59044670000006, 45.52239604000004, 83, 1306), (-73.57550530000006, 45.52239604000004, 42, 1437), (-73.56056390000006, 45.52239604000004, 24, 1049), (-73.54562250000006, 45.52239604000004, 7, 315), (-73.53068110000007, 45.52239604000004, 9, 65), (-73.51573970000007, 45.52239604000004, 0, 13), (-73.50079830000007, 45.52239604000004, 2, 10), (-73.48585690000007, 45.52239604000004, 0, 0), (-73.47091550000007, 45.52239604000004, 3, 162), (-73.45597410000008, 45.52239604000004, 0, 5), (-73.44103270000008, 45.52239604000004, 0, 0), (-73.42609130000008, 45.52239604000004, 0, 1), (-73.41114990000008, 45.52239604000004, 0, 0), (-73.39620850000009, 45.52239604000004, 0, 2), (-73.38126710000009, 45.52239604000004, 0, 0), (-73.36632570000009, 45.52239604000004, 0, 6), (-73.35138430000009, 45.52239604000004, 2, 4), (-73.3364429000001, 45.52239604000004, 3, 173), (-73.3215015000001, 45.52239604000004, 0, 0), (-73.3065601000001, 45.52239604000004, 0, 0), (-73.2916187000001, 45.52239604000004, 0, 4), (-74.0237473, 45.53258476000004, 0, 0), (-74.0088059, 45.53258476000004, 0, 0), (-73.9938645, 45.53258476000004, 0, 5), (-73.9789231,

45.53258476000004, 0, 0), (-73.9639817, 45.53258476000004, 0, 0), (-73.94904030000001, 45.53258476000004, 0, 0), (-73.93409890000001, 45.53258476000004, 0, 5), (-73.91915750000001, 45.53258476000004, 0, 0), (-73.90421610000001, 45.53258476000004, 0, 0), (-73.88927470000002, 45.53258476000004, 0, 9), (-73.87433330000002, 45.53258476000004, 0, 2), (-73.85939190000002, 45.53258476000004, 0, 0), (-73.84445050000002, 45.53258476000004, 0, 0), (-73.82950910000002, 45.53258476000004, 0, 1), (-73.81456770000003, 45.53258476000004, 0, 8), (-73.79962630000003, 45.53258476000004, 0, 5), (-73.78468490000003, 45.53258476000004, 0, 20), (-73.76974350000003, 45.53258476000004, 1, 41), (-73.75480210000003, 45.53258476000004, 0, 5), (-73.73986070000004, 45.53258476000004, 0, 29), (-73.72491930000004, 45.53258476000004, 0, 7), (-73.70997790000004, 45.53258476000004, 22, 27), (-73.69503650000004, 45.53258476000004, 0, 2), (-73.68009510000005, 45.53258476000004, 0, 12), (-73.66515370000005, 45.53258476000004, 5, 19), (-73.65021230000005, 45.53258476000004, 2, 329), (-73.63527090000005, 45.53258476000004, 0, 37), (-73.62032950000005, 45.53258476000004, 21, 765), (-73.60538810000006, 45.53258476000004, 47, 1355), (-73.59044670000006, 45.53258476000004, 7, 93), (-73.57550530000006, 45.53258476000004, 5, 99), (-73.56056390000006, 45.53258476000004, 3, 74), (-73.54562250000006, 45.53258476000004, 1, 40), (-73.53068110000007, 45.53258476000004, 0, 0), (-73.51573970000007, 45.53258476000004, 71, 1025), (-73.50079830000007, 45.53258476000004, 0, 1), (-73.48585690000007, 45.53258476000004, 0, 22), (-73.47091550000007, 45.53258476000004, 0, 1), (-73.45597410000008, 45.53258476000004, 0, 1), (-73.44103270000008, 45.53258476000004, 0, 12), (-73.42609130000008, 45.53258476000004, 0, 2), (-73.41114990000008, 45.53258476000004, 0, 0), (-73.39620850000009, 45.53258476000004, 0, 0), (-73.38126710000009, 45.53258476000004, 0, 0), (-73.36632570000009, 45.53258476000004, 0, 0), (-73.35138430000009, 45.53258476000004, 7, 211), (-73.3364429000001, 45.53258476000004, 0, 5), (-73.3215015000001, 45.53258476000004, 0, 0), (-73.3065601000001, 45.53258476000004, 0, 0), (-73.2916187000001, 45.53258476000004, 0, 0), (-74.0237473, 45.542773480000044, 0, 0), (-74.0088059, 45.542773480000044, 0, 3), (-73.9938645, 45.542773480000044, 0, 0), (-73.9789231, 45.542773480000044, 0, 0), (-73.9639817, 45.542773480000044, 0, 0), (-73.94904030000001, 45.542773480000044, 0, 0), (-73.93409890000001, 45.542773480000044, 0, 0), (-73.91915750000001, 45.542773480000044, 0, 3), (-73.90421610000001, 45.542773480000044, 0, 0), (-73.88927470000002, 45.542773480000044, 0, 6), (-73.87433330000002, 45.542773480000044, 0, 11), (-73.85939190000002, 45.542773480000044, 0, 5), (-73.84445050000002, 45.542773480000044, 0, 0), (-73.82950910000002, 45.542773480000044, 0, 0), (-73.81456770000003, 45.542773480000044, 0, 1), (-73.79962630000003, 45.542773480000044, 0, 0), (-73.78468490000003, 45.542773480000044, 0, 5), (-73.76974350000003, 45.542773480000044, 0, 12), (-73.75480210000003, 45.542773480000044, 3, 23), (-73.73986070000004, 45.542773480000044, 0, 0), (-73.72491930000004, 45.542773480000044, 1, 4), (-73.70997790000004, 45.542773480000044, 0, 1), (-73.69503650000004, 45.542773480000044, 0, 5), (-73.68009510000005, 45.542773480000044, 0, 3), (-73.66515370000005, 45.542773480000044, 1, 65), (-73.65021230000005, 45.542773480000044, 4, 34), (-73.63527090000005, 45.542773480000044, 7, 2457), (-73.62032950000005, 45.542773480000044, 1, 84), (-73.60538810000006, 45.542773480000044, 15, 569), (-73.59044670000006, 45.542773480000044, 4, 81), (-73.57550530000006, 45.542773480000044, 5, 37), (-73.56056390000006, 45.542773480000044, 0, 34), (-73.54562250000006, 45.542773480000044, 1, 180), (-73.53068110000007, 45.542773480000044, 1, 5), (-73.51573970000007, 45.542773480000044, 0, 4), (-73.50079830000007, 45.542773480000044, 0, 1), (-73.48585690000007, 45.542773480000044, 0, 6), (-73.47091550000007, 45.542773480000044, 1, 37), (-73.45597410000008, 45.542773480000044, 0, 1), (-73.44103270000008, 45.542773480000044, 0, 0),

(-73.42609130000008, 45.542773480000044, 0, 1), (-73.41114990000008, 45.542773480000044, 0, 0), (-73.39620850000009, 45.542773480000044, 0, 0), (-73.38126710000009, 45.542773480000044, 0, 0), (-73.36632570000009, 45.542773480000044, 1, 0), (-73.35138430000009, 45.542773480000044, 0, 0), (-73.3364429000001, 45.542773480000044, 1, 30), (-73.3215015000001, 45.542773480000044, 0, 0), (-73.3065601000001, 45.542773480000044, 0, 0), (-73.2916187000001, 45.542773480000044, 0, 0), (-74.0237473, 45.552962200000046, 0, 7), (-74.0088059, 45.552962200000046, 0, 0), (-73.9938645, 45.552962200000046, 0, 0), (-73.9789231, 45.552962200000046, 0, 0), (-73.9639817, 45.552962200000046, 0, 0), (-73.94904030000001, 45.552962200000046, 0, 0), (-73.93409890000001, 45.552962200000046, 0, 0), (-73.91915750000001, 45.552962200000046, 0, 0), (-73.90421610000001, 45.552962200000046, 0, 2), (-73.88927470000002, 45.552962200000046, 0, 1), (-73.87433330000002, 45.552962200000046, 0, 5), (-73.85939190000002, 45.552962200000046, 0, 0), (-73.84445050000002, 45.552962200000046, 0, 118), (-73.82950910000002, 45.552962200000046, 0, 0), (-73.81456770000003, 45.552962200000046, 0, 1), (-73.79962630000003, 45.552962200000046, 0, 0), (-73.78468490000003, 45.552962200000046, 0, 46), (-73.76974350000003, 45.552962200000046, 1, 15), (-73.75480210000003, 45.552962200000046, 1, 16), (-73.73986070000004, 45.552962200000046, 4, 32), (-73.72491930000004, 45.552962200000046, 1, 144), (-73.70997790000004, 45.552962200000046, 0, 1), (-73.69503650000004, 45.552962200000046, 4, 1147), (-73.68009510000005, 45.552962200000046, 0, 0), (-73.66515370000005, 45.552962200000046, 1, 23), (-73.65021230000005, 45.552962200000046, 0, 12), (-73.63527090000005, 45.552962200000046, 1, 23), (-73.62032950000005, 45.552962200000046, 0, 14), (-73.60538810000006, 45.552962200000046, 11, 373), (-73.59044670000006, 45.552962200000046, 0, 13), (-73.57550530000006, 45.552962200000046, 1, 34), (-73.56056390000006, 45.552962200000046, 2, 110), (-73.54562250000006, 45.552962200000046, 5, 192), (-73.53068110000007, 45.552962200000046, 0, 29), (-73.51573970000007, 45.552962200000046, 0, 0), (-73.50079830000007, 45.552962200000046, 0, 1), (-73.48585690000007, 45.552962200000046, 1, 9), (-73.47091550000007, 45.552962200000046, 0, 0), (-73.45597410000008, 45.552962200000046, 0, 2), (-73.44103270000008, 45.552962200000046, 0, 0), (-73.42609130000008, 45.552962200000046, 0, 2), (-73.41114990000008, 45.552962200000046, 0, 0), (-73.39620850000009, 45.552962200000046, 0, 0), (-73.38126710000009, 45.552962200000046, 0, 0), (-73.36632570000009, 45.552962200000046, 0, 0), (-73.35138430000009, 45.552962200000046, 0, 1), (-73.3364429000001, 45.552962200000046, 0, 0), (-73.3215015000001, 45.552962200000046, 0, 13), (-73.3065601000001, 45.552962200000046, 0, 0), (-73.2916187000001, 45.552962200000046, 0, 0), (-74.0237473, 45.56315092000005, 0, 0), (-74.0088059, 45.56315092000005, 6, 26), (-73.9938645, 45.56315092000005, 0, 0), (-73.9789231, 45.56315092000005, 0, 2), (-73.9639817, 45.56315092000005, 0, 0), (-73.94904030000001, 45.56315092000005, 0, 0), (-73.93409890000001, 45.56315092000005, 0, 0), (-73.91915750000001, 45.56315092000005, 0, 7), (-73.90421610000001, 45.56315092000005, 0, 42), (-73.88927470000002, 45.56315092000005, 8, 136), (-73.87433330000002, 45.56315092000005, 0, 0), (-73.85939190000002, 45.56315092000005, 0, 0), (-73.84445050000002, 45.56315092000005, 0, 3), (-73.82950910000002, 45.56315092000005, 0, 0), (-73.81456770000003, 45.56315092000005, 0, 3), (-73.79962630000003, 45.56315092000005, 0, 2), (-73.78468490000003, 45.56315092000005, 0, 34), (-73.76974350000003, 45.56315092000005, 0, 8), (-73.75480210000003, 45.56315092000005, 24, 216), (-73.73986070000004, 45.56315092000005, 9, 100), (-73.72491930000004, 45.56315092000005, 2, 82), (-73.70997790000004, 45.56315092000005, 0, 6), (-73.69503650000004, 45.56315092000005, 0, 9), (-73.68009510000005, 45.56315092000005, 0, 9), (-73.66515370000005, 45.56315092000005, 45, 2013), (-73.65021230000005, 45.56315092000005, 0, 16), (-73.63527090000005, 45.56315092000005, 1, 38),

(-73.62032950000005, 45.56315092000005, 1, 45), (-73.60538810000006, 45.56315092000005, 1, 43), (-73.59044670000006, 45.56315092000005, 0, 8), (-73.57550530000006, 45.56315092000005, 0, 89), (-73.56056390000006, 45.56315092000005, 0, 19), (-73.54562250000006, 45.56315092000005, 2, 101), (-73.53068110000007, 45.56315092000005, 0, 9), (-73.51573970000007, 45.56315092000005, 0, 3), (-73.50079830000007, 45.56315092000005, 0, 19), (-73.48585690000007, 45.56315092000005, 0, 1), (-73.47091550000007, 45.56315092000005, 0, 0), (-73.45597410000008, 45.56315092000005, 0, 3), (-73.44103270000008, 45.56315092000005, 0, 1), (-73.42609130000008, 45.56315092000005, 0, 81), (-73.41114990000008, 45.56315092000005, 0, 1), (-73.39620850000009, 45.56315092000005, 0, 0), (-73.38126710000009, 45.56315092000005, 0, 0), (-73.36632570000009, 45.56315092000005, 0, 0), (-73.35138430000009, 45.56315092000005, 0, 0), (-73.3364429000001, 45.56315092000005, 0, 10), (-73.3215015000001, 45.56315092000005, 0, 0), (-73.3065601000001, 45.56315092000005, 0, 0), (-73.2916187000001, 45.56315092000005, 16, 19), (-74.0237473, 45.57333964000005, 0, 0), (-74.0088059, 45.57333964000005, 0, 0), (-73.9938645, 45.57333964000005, 0, 0), (-73.9789231, 45.57333964000005, 0, 0), (-73.9639817, 45.57333964000005, 1, 25), (-73.94904030000001, 45.57333964000005, 0, 0), (-73.93409890000001, 45.57333964000005, 0, 0), (-73.91915750000001, 45.57333964000005, 0, 1), (-73.90421610000001, 45.57333964000005, 1, 4), (-73.88927470000002, 45.57333964000005, 0, 2), (-73.87433330000002, 45.57333964000005, 0, 0), (-73.85939190000002, 45.57333964000005, 0, 1), (-73.84445050000002, 45.57333964000005, 0, 0), (-73.82950910000002, 45.57333964000005, 0, 1), (-73.81456770000003, 45.57333964000005, 2, 12), (-73.79962630000003, 45.57333964000005, 0, 0), (-73.78468490000003, 45.57333964000005, 7, 961), (-73.76974350000003, 45.57333964000005, 0, 5), (-73.75480210000003, 45.57333964000005, 3, 227), (-73.73986070000004, 45.57333964000005, 0, 2), (-73.72491930000004, 45.57333964000005, 0, 32), (-73.70997790000004, 45.57333964000005, 17, 1332), (-73.69503650000004, 45.57333964000005, 0, 5), (-73.68009510000005, 45.57333964000005, 0, 5), (-73.66515370000005, 45.57333964000005, 11, 25), (-73.65021230000005, 45.57333964000005, 0, 4), (-73.63527090000005, 45.57333964000005, 1, 25), (-73.62032950000005, 45.57333964000005, 1, 5), (-73.60538810000006, 45.57333964000005, 3, 305), (-73.59044670000006, 45.57333964000005, 2, 92), (-73.57550530000006, 45.57333964000005, 0, 5), (-73.56056390000006, 45.57333964000005, 0, 11), (-73.54562250000006, 45.57333964000005, 0, 15), (-73.53068110000007, 45.57333964000005, 3, 105), (-73.51573970000007, 45.57333964000005, 0, 0), (-73.50079830000007, 45.57333964000005, 0, 0), (-73.48585690000007, 45.57333964000005, 1, 3), (-73.47091550000007, 45.57333964000005, 0, 12), (-73.45597410000008, 45.57333964000005, 0, 6), (-73.44103270000008, 45.57333964000005, 1, 30), (-73.42609130000008, 45.57333964000005, 0, 1), (-73.41114990000008, 45.57333964000005, 0, 35), (-73.39620850000009, 45.57333964000005, 8, 10), (-73.38126710000009, 45.57333964000005, 0, 1), (-73.36632570000009, 45.57333964000005, 0, 0), (-73.35138430000009, 45.57333964000005, 0, 0), (-73.3364429000001, 45.57333964000005, 0, 0), (-73.3215015000001, 45.57333964000005, 0, 0), (-73.3065601000001, 45.57333964000005, 0, 0), (-73.2916187000001, 45.57333964000005, 0, 0), (-74.0237473, 45.58352836000005, 0, 0), (-74.0088059, 45.58352836000005, 0, 0), (-73.9938645, 45.58352836000005, 0, 0), (-73.9789231, 45.58352836000005, 0, 4), (-73.9639817, 45.58352836000005, 0, 0), (-73.94904030000001, 45.58352836000005, 0, 0), (-73.93409890000001, 45.58352836000005, 0, 0), (-73.91915750000001, 45.58352836000005, 0, 0), (-73.90421610000001, 45.58352836000005, 0, 0), (-73.88927470000002, 45.58352836000005, 0, 0), (-73.87433330000002, 45.58352836000005, 0, 8), (-73.85939190000002, 45.58352836000005, 0, 0), (-73.84445050000002, 45.58352836000005, 0, 0), (-73.82950910000002, 45.58352836000005, 0, 0), (-73.81456770000003, 45.58352836000005, 0, 0), (-73.79962630000003, 45.58352836000005, 0, 1), (-73.78468490000003, 45.58352836000005, 0, 2), (-73.76974350000003, 45.58352836000005,

0, 2), (-73.75480210000003, 45.58352836000005, 0, 8), (-73.73986070000004, 45.58352836000005, 0, 4), (-73.72491930000004, 45.58352836000005, 0, 0), (-73.70997790000004, 45.58352836000005, 1, 9), (-73.69503650000004, 45.58352836000005, 0, 1), (-73.68009510000005, 45.58352836000005, 0, 0), (-73.66515370000005, 45.58352836000005, 0, 0), (-73.65021230000005, 45.58352836000005, 1, 18), (-73.63527090000005, 45.58352836000005, 1, 44), (-73.62032950000005, 45.58352836000005, 0, 0), (-73.60538810000006, 45.58352836000005, 4, 128), (-73.59044670000006, 45.58352836000005, 3, 51), (-73.57550530000006, 45.58352836000005, 0, 4), (-73.56056390000006, 45.58352836000005, 0, 9), (-73.54562250000006, 45.58352836000005, 0, 63), (-73.53068110000007, 45.58352836000005, 0, 6), (-73.51573970000007, 45.58352836000005, 0, 0), (-73.50079830000007, 45.58352836000005, 0, 0), (-73.48585690000007, 45.58352836000005, 0, 0), (-73.47091550000007, 45.58352836000005, 0, 0), (-73.45597410000008, 45.58352836000005, 0, 1), (-73.44103270000008, 45.58352836000005, 0, 6), (-73.42609130000008, 45.58352836000005, 0, 0), (-73.41114990000008, 45.58352836000005, 0, 0), (-73.39620850000009, 45.58352836000005, 0, 0), (-73.38126710000009, 45.58352836000005, 0, 0), (-73.36632570000009, 45.58352836000005, 0, 0), (-73.35138430000009, 45.58352836000005, 0, 0), (-73.3364429000001, 45.58352836000005, 0, 1), (-73.3215015000001, 45.58352836000005, 0, 1), (-73.3065601000001, 45.58352836000005, 0, 0), (-73.2916187000001, 45.58352836000005, 0, 0), (-74.0237473, 45.593717080000054, 0, 0), (-74.0088059, 45.593717080000054, 0, 0), (-73.9938645, 45.593717080000054, 0, 0), (-73.9789231, 45.593717080000054, 0, 0), (-73.9639817, 45.593717080000054, 0, 0), (-73.94904030000001, 45.593717080000054, 0, 1), (-73.93409890000001, 45.593717080000054, 0, 1), (-73.91915750000001, 45.593717080000054, 0, 0), (-73.90421610000001, 45.593717080000054, 0, 0), (-73.88927470000002, 45.593717080000054, 0, 0), (-73.87433330000002, 45.593717080000054, 0, 0), (-73.85939190000002, 45.593717080000054, 0, 0), (-73.84445050000002, 45.593717080000054, 0, 0), (-73.82950910000002, 45.593717080000054, 0, 0), (-73.81456770000003, 45.593717080000054, 0, 0), (-73.79962630000003, 45.593717080000054, 1, 1), (-73.78468490000003, 45.593717080000054, 0, 14), (-73.76974350000003, 45.593717080000054, 0, 1), (-73.75480210000003, 45.593717080000054, 0, 1), (-73.73986070000004, 45.593717080000054, 0, 2), (-73.72491930000004, 45.593717080000054, 0, 0), (-73.70997790000004, 45.593717080000054, 0, 0), (-73.69503650000004, 45.593717080000054, 1, 21), (-73.68009510000005, 45.593717080000054, 0, 1), (-73.66515370000005, 45.593717080000054, 0, 13), (-73.65021230000005, 45.593717080000054, 0, 4), (-73.63527090000005, 45.593717080000054, 0, 6), (-73.62032950000005, 45.593717080000054, 0, 1), (-73.60538810000006, 45.593717080000054, 0, 2), (-73.59044670000006, 45.593717080000054, 0, 12), (-73.57550530000006, 45.593717080000054, 1, 85), (-73.56056390000006, 45.593717080000054, 0, 2), (-73.54562250000006, 45.593717080000054, 1, 19), (-73.53068110000007, 45.593717080000054, 0, 16), (-73.51573970000007, 45.593717080000054, 0, 9), (-73.50079830000007, 45.593717080000054, 0, 0), (-73.48585690000007, 45.593717080000054, 0, 0), (-73.47091550000007, 45.593717080000054, 0, 1), (-73.45597410000008, 45.593717080000054, 0, 0), (-73.44103270000008, 45.593717080000054, 26, 363), (-73.42609130000008, 45.593717080000054, 0, 2), (-73.41114990000008, 45.593717080000054, 0, 0), (-73.39620850000009, 45.593717080000054, 0, 0), (-73.38126710000009, 45.593717080000054, 0, 0), (-73.36632570000009, 45.593717080000054, 0, 0), (-73.35138430000009, 45.593717080000054, 0, 0), (-73.3364429000001, 45.593717080000054, 0, 42), (-73.3215015000001, 45.593717080000054, 0, 6), (-73.3065601000001, 45.593717080000054, 0, 0), (-73.2916187000001, 45.593717080000054, 0, 0), (-74.0237473, 45.60390580000006, 0, 0), (-74.0088059, 45.60390580000006, 0, 0), (-73.9938645, 45.60390580000006, 0, 0), (-73.9789231, 45.60390580000006, 0, 0), (-73.9639817, 45.60390580000006, 0, 0), (-73.94904030000001, 45.60390580000006, 0, 0), (-73.93409890000001, 45.60390580000006, 0, 0),

(-73.91915750000001, 45.60390580000006, 0, 0), (-73.90421610000001, 45.60390580000006, 0, 0), (-73.88927470000002, 45.60390580000006, 0, 0), (-73.87433330000002, 45.60390580000006, 0, 0), (-73.85939190000002, 45.60390580000006, 0, 0), (-73.84445050000002, 45.60390580000006, 0, 0), (-73.82950910000002, 45.60390580000006, 0, 0), (-73.81456770000003, 45.60390580000006, 0, 0), (-73.79962630000003, 45.60390580000006, 0, 3), (-73.78468490000003, 45.60390580000006, 0, 1), (-73.76974350000003, 45.60390580000006, 0, 0), (-73.75480210000003, 45.60390580000006, 0, 0), (-73.73986070000004, 45.60390580000006, 32, 1160), (-73.72491930000004, 45.60390580000006, 2, 16), (-73.70997790000004, 45.60390580000006, 69, 1286), (-73.69503650000004, 45.60390580000006, 0, 0), (-73.68009510000005, 45.60390580000006, 0, 1), (-73.66515370000005, 45.60390580000006, 0, 12), (-73.65021230000005, 45.60390580000006, 0, 2), (-73.63527090000005, 45.60390580000006, 1, 32), (-73.62032950000005, 45.60390580000006, 4, 33), (-73.60538810000006, 45.60390580000006, 1, 23), (-73.59044670000006, 45.60390580000006, 1, 10), (-73.57550530000006, 45.60390580000006, 1, 7), (-73.56056390000006, 45.60390580000006, 0, 267), (-73.54562250000006, 45.60390580000006, 0, 4), (-73.53068110000007, 45.60390580000006, 0, 15), (-73.51573970000007, 45.60390580000006, 11, 21), (-73.50079830000007, 45.60390580000006, 0, 0), (-73.48585690000007, 45.60390580000006, 0, 0), (-73.47091550000007, 45.60390580000006, 0, 3), (-73.45597410000008, 45.60390580000006, 3, 22), (-73.44103270000008, 45.60390580000006, 0, 0), (-73.42609130000008, 45.60390580000006, 0, 0), (-73.41114990000008, 45.60390580000006, 0, 0), (-73.39620850000009, 45.60390580000006, 0, 0), (-73.38126710000009, 45.60390580000006, 0, 0), (-73.36632570000009, 45.60390580000006, 0, 0), (-73.35138430000009, 45.60390580000006, 1, 2), (-73.3364429000001, 45.60390580000006, 0, 0), (-73.3215015000001, 45.60390580000006, 0, 0), (-73.3065601000001, 45.60390580000006, 0, 2), (-73.2916187000001, 45.60390580000006, 0, 0), (-74.0237473, 45.61409452000006, 0, 0), (-74.0088059, 45.61409452000006, 0, 0), (-73.9938645, 45.61409452000006, 0, 1), (-73.9789231, 45.61409452000006, 0, 0), (-73.9639817, 45.61409452000006, 0, 0), (-73.94904030000001, 45.61409452000006, 0, 0), (-73.93409890000001, 45.61409452000006, 0, 0), (-73.91915750000001, 45.61409452000006, 0, 0), (-73.90421610000001, 45.61409452000006, 0, 0), (-73.88927470000002, 45.61409452000006, 0, 0), (-73.87433330000002, 45.61409452000006, 0, 0), (-73.85939190000002, 45.61409452000006, 0, 1), (-73.84445050000002, 45.61409452000006, 5, 122), (-73.82950910000002, 45.61409452000006, 0, 45), (-73.81456770000003, 45.61409452000006, 0, 0), (-73.79962630000003, 45.61409452000006, 0, 0), (-73.78468490000003, 45.61409452000006, 1, 98), (-73.76974350000003, 45.61409452000006, 0, 0), (-73.75480210000003, 45.61409452000006, 0, 2), (-73.73986070000004, 45.61409452000006, 0, 32), (-73.72491930000004, 45.61409452000006, 0, 3), (-73.70997790000004, 45.61409452000006, 0, 0), (-73.69503650000004, 45.61409452000006, 0, 11), (-73.68009510000005, 45.61409452000006, 0, 5), (-73.66515370000005, 45.61409452000006, 0, 0), (-73.65021230000005, 45.61409452000006, 0, 3), (-73.63527090000005, 45.61409452000006, 0, 1), (-73.62032950000005, 45.61409452000006, 0, 5), (-73.60538810000006, 45.61409452000006, 0, 76), (-73.59044670000006, 45.61409452000006, 20, 135), (-73.57550530000006, 45.61409452000006, 0, 318), (-73.56056390000006, 45.61409452000006, 5, 37), (-73.54562250000006, 45.61409452000006, 0, 2), (-73.53068110000007, 45.61409452000006, 0, 0), (-73.51573970000007, 45.61409452000006, 0, 490), (-73.50079830000007, 45.61409452000006, 0, 1), (-73.48585690000007, 45.61409452000006, 0, 0), (-73.47091550000007, 45.61409452000006, 1, 2), (-73.45597410000008, 45.61409452000006, 1, 0), (-73.44103270000008, 45.61409452000006, 0, 3), (-73.42609130000008, 45.61409452000006, 0, 0), (-73.41114990000008, 45.61409452000006, 0, 0), (-73.39620850000009, 45.61409452000006, 0, 0), (-73.38126710000009, 45.61409452000006, 0, 0), (-73.36632570000009, 45.61409452000006, 0, 0), (-73.35138430000009, 45.61409452000006, 0,

0), (-73.3364429000001, 45.61409452000006, 0, 0), (-73.3215015000001, 45.61409452000006, 0, 0), (-73.3065601000001, 45.61409452000006, 0, 0), (-73.2916187000001, 45.61409452000006, 0, 0), (-74.0237473, 45.62428324000006, 0, 0), (-74.0088059, 45.62428324000006, 0, 0), (-73.9938645, 45.62428324000006, 0, 0), (-73.9789231, 45.62428324000006, 0, 0), (-73.9639817, 45.62428324000006, 0, 0), (-73.94904030000001, 45.62428324000006, 0, 0), (-73.93409890000001, 45.62428324000006, 0, 0), (-73.91915750000001, 45.62428324000006, 0, 0), (-73.90421610000001, 45.62428324000006, 0, 0), (-73.88927470000002, 45.62428324000006, 0, 0), (-73.87433330000002, 45.62428324000006, 0, 0), (-73.85939190000002, 45.62428324000006, 0, 77), (-73.84445050000002, 45.62428324000006, 1, 16), (-73.82950910000002, 45.62428324000006, 0, 0), (-73.81456770000003, 45.62428324000006, 0, 1), (-73.79962630000003, 45.62428324000006, 0, 29), (-73.78468490000003, 45.62428324000006, 0, 0), (-73.76974350000003, 45.62428324000006, 1, 1), (-73.75480210000003, 45.62428324000006, 0, 1), (-73.73986070000004, 45.62428324000006, 0, 0), (-73.72491930000004, 45.62428324000006, 0, 1), (-73.70997790000004, 45.62428324000006, 0, 0), (-73.69503650000004, 45.62428324000006, 0, 1), (-73.68009510000005, 45.62428324000006, 0, 3), (-73.66515370000005, 45.62428324000006, 0, 0), (-73.65021230000005, 45.62428324000006, 0, 0), (-73.63527090000005, 45.62428324000006, 0, 1), (-73.62032950000005, 45.62428324000006, 0, 1), (-73.60538810000006, 45.62428324000006, 0, 50), (-73.59044670000006, 45.62428324000006, 0, 109), (-73.57550530000006, 45.62428324000006, 0, 3), (-73.56056390000006, 45.62428324000006, 0, 4), (-73.54562250000006, 45.62428324000006, 0, 190), (-73.53068110000007, 45.62428324000006, 0, 0), (-73.51573970000007, 45.62428324000006, 0, 0), (-73.50079830000007, 45.62428324000006, 0, 0), (-73.48585690000007, 45.62428324000006, 0, 0), (-73.47091550000007, 45.62428324000006, 0, 0), (-73.45597410000008, 45.62428324000006, 0, 0), (-73.44103270000008, 45.62428324000006, 1, 32), (-73.42609130000008, 45.62428324000006, 0, 0), (-73.41114990000008, 45.62428324000006, 0, 0), (-73.39620850000009, 45.62428324000006, 0, 0), (-73.38126710000009, 45.62428324000006, 0, 0), (-73.36632570000009, 45.62428324000006, 0, 0), (-73.35138430000009, 45.62428324000006, 0, 0), (-73.3364429000001, 45.62428324000006, 0, 0), (-73.3215015000001, 45.62428324000006, 0, 0), (-73.3065601000001, 45.62428324000006, 0, 0), (-73.2916187000001, 45.62428324000006, 0, 0), (-74.0237473, 45.63447196000006, 0, 0), (-74.0088059, 45.63447196000006, 0, 0), (-73.9938645, 45.63447196000006, 0, 0), (-73.9789231, 45.63447196000006, 0, 0), (-73.9639817, 45.63447196000006, 0, 0), (-73.94904030000001, 45.63447196000006, 0, 0), (-73.93409890000001, 45.63447196000006, 0, 0), (-73.91915750000001, 45.63447196000006, 0, 0), (-73.90421610000001, 45.63447196000006, 0, 1), (-73.88927470000002, 45.63447196000006, 0, 0), (-73.87433330000002, 45.63447196000006, 0, 0), (-73.85939190000002, 45.63447196000006, 0, 33), (-73.84445050000002, 45.63447196000006, 0, 5), (-73.82950910000002, 45.63447196000006, 0, 1), (-73.81456770000003, 45.63447196000006, 1, 9), (-73.79962630000003, 45.63447196000006, 0, 3), (-73.78468490000003, 45.63447196000006, 20, 135), (-73.76974350000003, 45.63447196000006, 0, 0), (-73.75480210000003, 45.63447196000006, 0, 1), (-73.73986070000004, 45.63447196000006, 0, 0), (-73.72491930000004, 45.63447196000006, 0, 0), (-73.70997790000004, 45.63447196000006, 0, 0), (-73.69503650000004, 45.63447196000006, 0, 0), (-73.68009510000005, 45.63447196000006, 0, 0), (-73.66515370000005, 45.63447196000006, 0, 0), (-73.65021230000005, 45.63447196000006, 0, 4), (-73.63527090000005, 45.63447196000006, 0, 0), (-73.62032950000005, 45.63447196000006, 0, 1), (-73.60538810000006, 45.63447196000006, 0, 1), (-73.59044670000006, 45.63447196000006, 0, 28), (-73.57550530000006, 45.63447196000006, 0, 0), (-73.56056390000006, 45.63447196000006, 0, 1), (-73.54562250000006, 45.63447196000006, 0, 140), (-73.53068110000007, 45.63447196000006, 0, 1), (-73.51573970000007, 45.63447196000006, 0, 2), (-73.50079830000007, 45.63447196000006, 13, 391), (-73.48585690000007, 45.63447196000006, 0, 5),

(-73.47091550000007, 45.63447196000006, 0, 0), (-73.45597410000008, 45.63447196000006, 0, 0), (-73.44103270000008, 45.63447196000006, 0, 0), (-73.42609130000008, 45.63447196000006, 0, 0), (-73.41114990000008, 45.63447196000006, 0, 0), (-73.39620850000009, 45.63447196000006, 0, 0), (-73.38126710000009, 45.63447196000006, 0, 1), (-73.36632570000009, 45.63447196000006, 0, 0), (-73.35138430000009, 45.63447196000006, 0, 0), (-73.3364429000001, 45.63447196000006, 0, 0), (-73.3215015000001, 45.63447196000006, 0, 0), (-73.3065601000001, 45.63447196000006, 0, 0), (-73.2916187000001, 45.63447196000006, 0, 0), (-74.0237473, 45.644660680000065, 0, 0), (-74.0088059, 45.644660680000065, 0, 0), (-73.9938645, 45.644660680000065, 0, 0), (-73.9789231, 45.644660680000065, 0, 0), (-73.9639817, 45.644660680000065, 0, 0), (-73.94904030000001, 45.644660680000065, 0, 0), (-73.93409890000001, 45.644660680000065, 0, 0), (-73.91915750000001, 45.644660680000065, 0, 0), (-73.90421610000001, 45.644660680000065, 0, 1), (-73.88927470000002, 45.644660680000065, 0, 0), (-73.87433330000002, 45.644660680000065, 0, 11), (-73.85939190000002, 45.644660680000065, 0, 7), (-73.84445050000002, 45.644660680000065, 1, 47), (-73.82950910000002, 45.644660680000065, 0, 1), (-73.81456770000003, 45.644660680000065, 0, 0), (-73.79962630000003, 45.644660680000065, 0, 0), (-73.78468490000003, 45.644660680000065, 0, 2), (-73.76974350000003, 45.644660680000065, 0, 2), (-73.75480210000003, 45.644660680000065, 0, 2), (-73.73986070000004, 45.644660680000065, 0, 0), (-73.72491930000004, 45.644660680000065, 0, 1), (-73.70997790000004, 45.644660680000065, 0, 0), (-73.69503650000004, 45.644660680000065, 0, 0), (-73.68009510000005, 45.644660680000065, 0, 0), (-73.66515370000005, 45.644660680000065, 0, 0), (-73.65021230000005, 45.644660680000065, 0, 0), (-73.63527090000005, 45.644660680000065, 0, 0), (-73.62032950000005, 45.644660680000065, 0, 0), (-73.60538810000006, 45.644660680000065, 0, 0), (-73.59044670000006, 45.644660680000065, 0, 3), (-73.57550530000006, 45.644660680000065, 0, 12), (-73.56056390000006, 45.644660680000065, 0, 0), (-73.54562250000006, 45.644660680000065, 0, 0), (-73.53068110000007, 45.644660680000065, 0, 0), (-73.51573970000007, 45.644660680000065, 0, 3), (-73.50079830000007, 45.644660680000065, 4, 18), (-73.48585690000007, 45.644660680000065, 0, 2), (-73.47091550000007, 45.644660680000065, 0, 0), (-73.45597410000008, 45.644660680000065, 0, 0), (-73.44103270000008, 45.644660680000065, 0, 0), (-73.42609130000008, 45.644660680000065, 0, 0), (-73.41114990000008, 45.644660680000065, 0, 0), (-73.39620850000009, 45.644660680000065, 0, 0), (-73.38126710000009, 45.644660680000065, 0, 0), (-73.36632570000009, 45.644660680000065, 0, 0), (-73.35138430000009, 45.644660680000065, 0, 0), (-73.3364429000001, 45.644660680000065, 0, 0), (-73.3215015000001, 45.644660680000065, 0, 0), (-73.3065601000001, 45.644660680000065, 0, 0), (-73.2916187000001, 45.644660680000065, 0, 0), (-74.0237473, 45.65484940000007, 0, 0), (-74.0088059, 45.65484940000007, 0, 0), (-73.9938645, 45.65484940000007, 0, 0), (-73.9789231, 45.65484940000007, 0, 0), (-73.9639817, 45.65484940000007, 0, 0), (-73.94904030000001, 45.65484940000007, 0, 0), (-73.93409890000001, 45.65484940000007, 0, 0), (-73.91915750000001, 45.65484940000007, 0, 0), (-73.90421610000001, 45.65484940000007, 0, 2), (-73.88927470000002, 45.65484940000007, 0, 0), (-73.87433330000002, 45.65484940000007, 0, 0), (-73.85939190000002, 45.65484940000007, 0, 11), (-73.84445050000002, 45.65484940000007, 0, 0), (-73.82950910000002, 45.65484940000007, 0, 0), (-73.81456770000003, 45.65484940000007, 0, 1), (-73.79962630000003, 45.65484940000007, 0, 12), (-73.78468490000003, 45.65484940000007, 1, 1), (-73.76974350000003, 45.65484940000007, 2, 10), (-73.75480210000003, 45.65484940000007, 0, 0), (-73.73986070000004, 45.65484940000007, 0, 0), (-73.72491930000004, 45.65484940000007, 0, 0), (-73.70997790000004, 45.65484940000007, 0, 0), (-73.69503650000004, 45.65484940000007, 0, 0), (-73.68009510000005, 45.65484940000007, 0, 0), (-73.66515370000005, 45.65484940000007, 0, 0), (-73.65021230000005, 45.65484940000007, 0, 0), (-73.63527090000005, 45.65484940000007, 0,

0), (-73.62032950000005, 45.65484940000007, 0, 0), (-73.60538810000006, 45.65484940000007, 0, 1), (-73.59044670000006, 45.65484940000007, 0, 0), (-73.57550530000006, 45.65484940000007, 0, 23), (-73.56056390000006, 45.65484940000007, 0, 5), (-73.54562250000006, 45.65484940000007, 0, 1), (-73.53068110000007, 45.65484940000007, 0, 141), (-73.51573970000007, 45.65484940000007, 3, 40), (-73.50079830000007, 45.65484940000007, 1, 76), (-73.48585690000007, 45.65484940000007, 0, 0), (-73.47091550000007, 45.65484940000007, 0, 0), (-73.45597410000008, 45.65484940000007, 0, 0), (-73.44103270000008, 45.65484940000007, 0, 0), (-73.42609130000008, 45.65484940000007, 0, 0), (-73.41114990000008, 45.65484940000007, 0, 0), (-73.39620850000009, 45.65484940000007, 0, 0), (-73.38126710000009, 45.65484940000007, 0, 0), (-73.36632570000009, 45.65484940000007, 0, 0), (-73.35138430000009, 45.65484940000007, 0, 0), (-73.3364429000001, 45.65484940000007, 0, 0), (-73.3215015000001, 45.65484940000007, 0, 0), (-73.3065601000001, 45.65484940000007, 0, 1), (-73.2916187000001, 45.65484940000007, 0, 0), (-74.0237473, 45.66503812000007, 0, 0), (-74.0088059, 45.66503812000007, 0, 18), (-73.9938645, 45.66503812000007, 0, 0), (-73.9789231, 45.66503812000007, 0, 0), (-73.9639817, 45.66503812000007, 0, 0), (-73.94904030000001, 45.66503812000007, 0, 0), (-73.93409890000001, 45.66503812000007, 0, 0), (-73.91915750000001, 45.66503812000007, 0, 0), (-73.90421610000001, 45.66503812000007, 0, 0), (-73.88927470000002, 45.66503812000007, 0, 0), (-73.87433330000002, 45.66503812000007, 54, 1113), (-73.85939190000002, 45.66503812000007, 76, 353), (-73.84445050000002, 45.66503812000007, 0, 0), (-73.82950910000002, 45.66503812000007, 0, 0), (-73.81456770000003, 45.66503812000007, 0, 0), (-73.79962630000003, 45.66503812000007, 0, 0), (-73.78468490000003, 45.66503812000007, 1, 1), (-73.76974350000003, 45.66503812000007, 0, 0), (-73.75480210000003, 45.66503812000007, 0, 9), (-73.73986070000004, 45.66503812000007, 0, 0), (-73.72491930000004, 45.66503812000007, 0, 0), (-73.70997790000004, 45.66503812000007, 0, 1), (-73.69503650000004, 45.66503812000007, 0, 0), (-73.68009510000005, 45.66503812000007, 0, 0), (-73.66515370000005, 45.66503812000007, 0, 0), (-73.65021230000005, 45.66503812000007, 0, 0), (-73.63527090000005, 45.66503812000007, 0, 0), (-73.62032950000005, 45.66503812000007, 0, 0), (-73.60538810000006, 45.66503812000007, 0, 0), (-73.59044670000006, 45.66503812000007, 0, 2), (-73.57550530000006, 45.66503812000007, 0, 0), (-73.56056390000006, 45.66503812000007, 2, 100), (-73.54562250000006, 45.66503812000007, 0, 3), (-73.53068110000007, 45.66503812000007, 0, 0), (-73.51573970000007, 45.66503812000007, 0, 1), (-73.50079830000007, 45.66503812000007, 0, 4), (-73.48585690000007, 45.66503812000007, 0, 0), (-73.47091550000007, 45.66503812000007, 0, 0), (-73.45597410000008, 45.66503812000007, 0, 0), (-73.44103270000008, 45.66503812000007, 0, 0), (-73.42609130000008, 45.66503812000007, 0, 0), (-73.41114990000008, 45.66503812000007, 0, 0), (-73.39620850000009, 45.66503812000007, 0, 0), (-73.38126710000009, 45.66503812000007, 0, 0), (-73.36632570000009, 45.66503812000007, 0, 0), (-73.35138430000009, 45.66503812000007, 0, 0), (-73.3364429000001, 45.66503812000007, 0, 0), (-73.3215015000001, 45.66503812000007, 0, 0), (-73.3065601000001, 45.66503812000007, 0, 0), (-73.2916187000001, 45.66503812000007, 0, 0), (-74.0237473, 45.67522684000007, 0, 0), (-74.0088059, 45.67522684000007, 0, 0), (-73.9938645, 45.67522684000007, 0, 0), (-73.9789231, 45.67522684000007, 0, 0), (-73.9639817, 45.67522684000007, 0, 0), (-73.94904030000001, 45.67522684000007, 0, 0), (-73.93409890000001, 45.67522684000007, 0, 0), (-73.91915750000001, 45.67522684000007, 0, 10), (-73.90421610000001, 45.67522684000007, 0, 9), (-73.88927470000002, 45.67522684000007, 0, 0), (-73.87433330000002, 45.67522684000007, 2, 1), (-73.85939190000002, 45.67522684000007, 0, 0), (-73.84445050000002, 45.67522684000007, 0, 0), (-73.82950910000002, 45.67522684000007, 0, 0), (-73.81456770000003, 45.67522684000007, 0, 0), (-73.79962630000003, 45.67522684000007, 0, 0), (-73.78468490000003, 45.67522684000007, 0, 0), (-73.76974350000003, 45.67522684000007, 0, 5),

(-73.75480210000003, 45.67522684000007, 0, 0), (-73.73986070000004, 45.67522684000007, 0, 0), (-73.72491930000004, 45.67522684000007, 0, 0), (-73.70997790000004, 45.67522684000007, 0, 0), (-73.69503650000004, 45.67522684000007, 0, 0), (-73.68009510000005, 45.67522684000007, 0, 0), (-73.66515370000005, 45.67522684000007, 0, 0), (-73.65021230000005, 45.67522684000007, 0, 0), (-73.63527090000005, 45.67522684000007, 0, 0), (-73.62032950000005, 45.67522684000007, 0, 0), (-73.60538810000006, 45.67522684000007, 0, 1), (-73.59044670000006, 45.67522684000007, 0, 0), (-73.57550530000006, 45.67522684000007, 0, 0), (-73.56056390000006, 45.67522684000007, 0, 0), (-73.54562250000006, 45.67522684000007, 0, 0), (-73.53068110000007, 45.67522684000007, 0, 0), (-73.51573970000007, 45.67522684000007, 0, 0), (-73.50079830000007, 45.67522684000007, 0, 2), (-73.48585690000007, 45.67522684000007, 0, 0), (-73.47091550000007, 45.67522684000007, 0, 0), (-73.45597410000008, 45.67522684000007, 0, 0), (-73.44103270000008, 45.67522684000007, 0, 0), (-73.42609130000008, 45.67522684000007, 0, 1), (-73.41114990000008, 45.67522684000007, 0, 0), (-73.39620850000009, 45.67522684000007, 0, 0), (-73.38126710000009, 45.67522684000007, 0, 0), (-73.36632570000009, 45.67522684000007, 0, 0), (-73.35138430000009, 45.67522684000007, 0, 0), (-73.3364429000001, 45.67522684000007, 0, 0), (-73.3215015000001, 45.67522684000007, 0, 0), (-73.3065601000001, 45.67522684000007, 0, 0), (-73.2916187000001, 45.67522684000007, 0, 0), (-74.0237473, 45.685415560000074, 0, 22), (-74.0088059, 45.685415560000074, 0, 0), (-73.9938645, 45.685415560000074, 0, 0), (-73.9789231, 45.685415560000074, 0, 0), (-73.9639817, 45.685415560000074, 0, 0), (-73.94904030000001, 45.685415560000074, 0, 0), (-73.93409890000001, 45.685415560000074, 0, 0), (-73.91915750000001, 45.685415560000074, 0, 0), (-73.90421610000001, 45.685415560000074, 0, 1), (-73.88927470000002, 45.685415560000074, 0, 0), (-73.87433330000002, 45.685415560000074, 8, 21), (-73.85939190000002, 45.685415560000074, 0, 0), (-73.84445050000002, 45.685415560000074, 0, 0), (-73.82950910000002, 45.685415560000074, 0, 0), (-73.81456770000003, 45.685415560000074, 0, 0), (-73.79962630000003, 45.685415560000074, 0, 0), (-73.78468490000003, 45.685415560000074, 0, 0), (-73.76974350000003, 45.685415560000074, 0, 0), (-73.75480210000003, 45.685415560000074, 0, 0), (-73.73986070000004, 45.685415560000074, 0, 0), (-73.72491930000004, 45.685415560000074, 0, 0), (-73.70997790000004, 45.685415560000074, 0, 0), (-73.69503650000004, 45.685415560000074, 0, 0), (-73.68009510000005, 45.685415560000074, 0, 0), (-73.66515370000005, 45.685415560000074, 0, 0), (-73.65021230000005, 45.685415560000074, 0, 0), (-73.63527090000005, 45.685415560000074, 0, 0), (-73.62032950000005, 45.685415560000074, 0, 0), (-73.60538810000006, 45.685415560000074, 0, 0), (-73.59044670000006, 45.685415560000074, 0, 0), (-73.57550530000006, 45.685415560000074, 0, 0), (-73.56056390000006, 45.685415560000074, 0, 0), (-73.54562250000006, 45.685415560000074, 0, 0), (-73.53068110000007, 45.685415560000074, 0, 0), (-73.51573970000007, 45.685415560000074, 0, 1), (-73.50079830000007, 45.685415560000074, 0, 1), (-73.48585690000007, 45.685415560000074, 0, 0), (-73.47091550000007, 45.685415560000074, 0, 0), (-73.45597410000008, 45.685415560000074, 0, 0), (-73.44103270000008, 45.685415560000074, 4, 19), (-73.42609130000008, 45.685415560000074, 0, 7), (-73.41114990000008, 45.685415560000074, 0, 0), (-73.39620850000009, 45.685415560000074, 0, 0), (-73.38126710000009, 45.685415560000074, 0, 0), (-73.36632570000009, 45.685415560000074, 0, 0), (-73.35138430000009, 45.685415560000074, 0, 0), (-73.3364429000001, 45.685415560000074, 0, 0), (-73.3215015000001, 45.685415560000074, 0, 0), (-73.3065601000001, 45.685415560000074, 0, 0), (-73.2916187000001, 45.685415560000074, 0, 0), (-74.0237473, 45.695604280000076, 0, 2), (-74.0088059, 45.695604280000076, 0, 0), (-73.9938645, 45.695604280000076, 0, 0), (-73.9789231, 45.695604280000076, 0, 0), (-73.9639817, 45.695604280000076, 0, 0), (-73.94904030000001, 45.695604280000076, 0, 0), (-73.93409890000001, 45.695604280000076, 0, 0), (-73.91915750000001, 45.695604280000076,

0, 0), (-73.90421610000001, 45.695604280000076, 0, 0), (-73.88927470000002, 45.695604280000076, 0, 0), (-73.87433330000002, 45.695604280000076, 0, 0), (-73.85939190000002, 45.695604280000076, 0, 0), (-73.84445050000002, 45.695604280000076, 0, 0), (-73.82950910000002, 45.695604280000076, 0, 0), (-73.81456770000003, 45.695604280000076, 0, 6), (-73.79962630000003, 45.695604280000076, 0, 0), (-73.78468490000003, 45.695604280000076, 0, 0), (-73.76974350000003, 45.695604280000076, 0, 0), (-73.75480210000003, 45.695604280000076, 0, 0), (-73.73986070000004, 45.695604280000076, 0, 0), (-73.72491930000004, 45.695604280000076, 0, 0), (-73.70997790000004, 45.695604280000076, 0, 5), (-73.69503650000004, 45.695604280000076, 0, 0), (-73.68009510000005, 45.695604280000076, 0, 0), (-73.66515370000005, 45.695604280000076, 0, 0), (-73.65021230000005, 45.695604280000076, 0, 2), (-73.63527090000005, 45.695604280000076, 1, 65), (-73.62032950000005, 45.695604280000076, 0, 0), (-73.60538810000006, 45.695604280000076, 0, 137), (-73.59044670000006, 45.695604280000076, 0, 0), (-73.57550530000006, 45.695604280000076, 0, 0), (-73.56056390000006, 45.695604280000076, 0, 0), (-73.54562250000006, 45.695604280000076, 0, 0), (-73.53068110000007, 45.695604280000076, 0, 0), (-73.51573970000007, 45.695604280000076, 0, 1), (-73.50079830000007, 45.695604280000076, 0, 0), (-73.48585690000007, 45.695604280000076, 0, 1), (-73.47091550000007, 45.695604280000076, 0, 0), (-73.45597410000008, 45.695604280000076, 0, 0), (-73.44103270000008, 45.695604280000076, 0, 0), (-73.42609130000008, 45.695604280000076, 0, 0), (-73.41114990000008, 45.695604280000076, 0, 0), (-73.39620850000009, 45.695604280000076, 0, 0), (-73.38126710000009, 45.695604280000076, 0, 0), (-73.36632570000009, 45.695604280000076, 0, 0), (-73.35138430000009, 45.695604280000076, 0, 0), (-73.3364429000001, 45.695604280000076, 0, 0), (-73.3215015000001, 45.695604280000076, 0, 0), (-73.3065601000001, 45.695604280000076, 0, 0), (-73.2916187000001, 45.695604280000076, 0, 0), (-74.0237473, 45.70579300000008, 0, 0), (-74.0088059, 45.70579300000008, 0, 0), (-73.9938645, 45.70579300000008, 0, 0), (-73.9789231, 45.70579300000008, 0, 0), (-73.9639817, 45.70579300000008, 0, 0), (-73.94904030000001, 45.70579300000008, 0, 0), (-73.93409890000001, 45.70579300000008, 0, 0), (-73.91915750000001, 45.70579300000008, 0, 0), (-73.90421610000001, 45.70579300000008, 0, 0), (-73.88927470000002, 45.70579300000008, 0, 0), (-73.87433330000002, 45.70579300000008, 0, 0), (-73.85939190000002, 45.70579300000008, 0, 0), (-73.84445050000002, 45.70579300000008, 0, 0), (-73.82950910000002, 45.70579300000008, 0, 0), (-73.81456770000003, 45.70579300000008, 0, 0), (-73.79962630000003, 45.70579300000008, 0, 0), (-73.78468490000003, 45.70579300000008, 0, 0), (-73.76974350000003, 45.70579300000008, 0, 0), (-73.75480210000003, 45.70579300000008, 0, 0), (-73.73986070000004, 45.70579300000008, 0, 0), (-73.72491930000004, 45.70579300000008, 0, 0), (-73.70997790000004, 45.70579300000008, 0, 0), (-73.69503650000004, 45.70579300000008, 0, 0), (-73.68009510000005, 45.70579300000008, 0, 0), (-73.66515370000005, 45.70579300000008, 0, 0), (-73.65021230000005, 45.70579300000008, 1, 12), (-73.63527090000005, 45.70579300000008, 0, 2), (-73.62032950000005, 45.70579300000008, 0, 1), (-73.60538810000006, 45.70579300000008, 0, 0), (-73.59044670000006, 45.70579300000008, 0, 0), (-73.57550530000006, 45.70579300000008, 0, 0), (-73.56056390000006, 45.70579300000008, 0, 0), (-73.54562250000006, 45.70579300000008, 0, 0), (-73.53068110000007, 45.70579300000008, 0, 0), (-73.51573970000007, 45.70579300000008, 0, 5), (-73.50079830000007, 45.70579300000008, 0, 0), (-73.48585690000007, 45.70579300000008, 0, 0), (-73.47091550000007, 45.70579300000008, 0, 2), (-73.45597410000008, 45.70579300000008, 0, 0), (-73.44103270000008, 45.70579300000008, 0, 0), (-73.42609130000008, 45.70579300000008, 0, 0), (-73.41114990000008, 45.70579300000008, 0, 0), (-73.39620850000009, 45.70579300000008, 0, 0), (-73.38126710000009, 45.70579300000008, 0, 0), (-73.36632570000009, 45.70579300000008, 0, 0), (-73.35138430000009, 45.70579300000008, 0, 0), (-73.3364429000001,

45.70579300000008, 0, 0), (-73.3215015000001, 45.70579300000008, 0, 0), (-73.3065601000001,
45.70579300000008, 0, 0), (-73.2916187000001, 45.70579300000008, 0, 0), (-74.0237473,
45.71598172000008, 0, 0), (-74.0088059, 45.71598172000008, 0, 0), (-73.9938645,
45.71598172000008, 0, 0), (-73.9789231, 45.71598172000008, 0, 0), (-73.9639817,
45.71598172000008, 0, 0), (-73.94904030000001, 45.71598172000008, 0, 0),
(-73.93409890000001, 45.71598172000008, 0, 0), (-73.91915750000001, 45.71598172000008, 0,
0), (-73.90421610000001, 45.71598172000008, 0, 0), (-73.88927470000002, 45.71598172000008,
0, 0), (-73.87433330000002, 45.71598172000008, 0, 0), (-73.85939190000002,
45.71598172000008, 0, 0), (-73.84445050000002, 45.71598172000008, 0, 0),
(-73.82950910000002, 45.71598172000008, 0, 0), (-73.81456770000003, 45.71598172000008, 0,
0), (-73.79962630000003, 45.71598172000008, 0, 0), (-73.78468490000003, 45.71598172000008,
0, 0), (-73.76974350000003, 45.71598172000008, 0, 0), (-73.75480210000003,
45.71598172000008, 0, 0), (-73.73986070000004, 45.71598172000008, 0, 0),
(-73.72491930000004, 45.71598172000008, 0, 0), (-73.70997790000004, 45.71598172000008, 0,
14), (-73.69503650000004, 45.71598172000008, 0, 6), (-73.68009510000005,
45.71598172000008, 0, 0), (-73.66515370000005, 45.71598172000008, 0, 0),
(-73.65021230000005, 45.71598172000008, 0, 0), (-73.63527090000005, 45.71598172000008, 0,
0), (-73.62032950000005, 45.71598172000008, 0, 0), (-73.60538810000006, 45.71598172000008,
0, 2), (-73.59044670000006, 45.71598172000008, 0, 0), (-73.57550530000006,
45.71598172000008, 0, 0), (-73.56056390000006, 45.71598172000008, 0, 0),
(-73.54562250000006, 45.71598172000008, 13, 71), (-73.53068110000007, 45.71598172000008,
0, 0), (-73.51573970000007, 45.71598172000008, 0, 80), (-73.50079830000007,
45.71598172000008, 0, 10), (-73.48585690000007, 45.71598172000008, 0, 5),
(-73.47091550000007, 45.71598172000008, 0, 1), (-73.45597410000008, 45.71598172000008, 0,
0), (-73.44103270000008, 45.71598172000008, 0, 0), (-73.42609130000008, 45.71598172000008,
0, 0), (-73.41114990000008, 45.71598172000008, 0, 0), (-73.39620850000009,
45.71598172000008, 0, 0), (-73.38126710000009, 45.71598172000008, 0, 0),
(-73.36632570000009, 45.71598172000008, 0, 0), (-73.35138430000009, 45.71598172000008, 0,
0), (-73.3364429000001, 45.71598172000008, 0, 0), (-73.3215015000001, 45.71598172000008, 0,
0), (-73.3065601000001, 45.71598172000008, 0, 0), (-73.2916187000001, 45.71598172000008, 0,
0), (-74.0237473, 45.72617044000008, 0, 0), (-74.0088059, 45.72617044000008, 0, 0),
(-73.9938645, 45.72617044000008, 0, 0), (-73.9789231, 45.72617044000008, 0, 0), (-73.9639817,
45.72617044000008, 0, 0), (-73.94904030000001, 45.72617044000008, 0, 0),
(-73.93409890000001, 45.72617044000008, 0, 0), (-73.91915750000001, 45.72617044000008, 0,
0), (-73.90421610000001, 45.72617044000008, 0, 0), (-73.88927470000002, 45.72617044000008,
0, 0), (-73.87433330000002, 45.72617044000008, 0, 0), (-73.85939190000002,
45.72617044000008, 0, 0), (-73.84445050000002, 45.72617044000008, 0, 0),
(-73.82950910000002, 45.72617044000008, 0, 2), (-73.81456770000003, 45.72617044000008, 0,
0), (-73.79962630000003, 45.72617044000008, 0, 0), (-73.78468490000003, 45.72617044000008,
0, 0), (-73.76974350000003, 45.72617044000008, 0, 0), (-73.75480210000003,
45.72617044000008, 0, 0), (-73.73986070000004, 45.72617044000008, 0, 0),
(-73.72491930000004, 45.72617044000008, 0, 0), (-73.70997790000004, 45.72617044000008, 0,
0), (-73.69503650000004, 45.72617044000008, 0, 1), (-73.68009510000005, 45.72617044000008,
0, 1), (-73.66515370000005, 45.72617044000008, 0, 0), (-73.65021230000005,
45.72617044000008, 0, 0), (-73.63527090000005, 45.72617044000008, 0, 1),
(-73.62032950000005, 45.72617044000008, 0, 54), (-73.60538810000006, 45.72617044000008, 0,
0), (-73.59044670000006, 45.72617044000008, 0, 0), (-73.57550530000006, 45.72617044000008,
0, 0), (-73.56056390000006, 45.72617044000008, 0, 0), (-73.54562250000006,
45.72617044000008, 0, 0), (-73.53068110000007, 45.72617044000008, 0, 0),
(-73.51573970000007, 45.72617044000008, 0, 1), (-73.50079830000007, 45.72617044000008, 0,
0), (-73.48585690000007, 45.72617044000008, 0, 0), (-73.47091550000007, 45.72617044000008,

0, 0), (-73.45597410000008, 45.72617044000008, 0, 2), (-73.44103270000008, 45.72617044000008, 0, 0), (-73.42609130000008, 45.72617044000008, 0, 0), (-73.41114990000008, 45.72617044000008, 0, 0), (-73.39620850000009, 45.72617044000008, 0, 0), (-73.38126710000009, 45.72617044000008, 0, 0), (-73.36632570000009, 45.72617044000008, 0, 0), (-73.35138430000009, 45.72617044000008, 0, 0), (-73.3364429000001, 45.72617044000008, 0, 0), (-73.3215015000001, 45.72617044000008, 0, 0), (-73.3065601000001, 45.72617044000008, 0, 0), (-73.2916187000001, 45.72617044000008, 0, 0), (-74.0237473, 45.736359160000085, 0, 0), (-74.0088059, 45.736359160000085, 0, 0), (-73.9938645, 45.736359160000085, 0, 0), (-73.9789231, 45.736359160000085, 0, 0), (-73.9639817, 45.736359160000085, 0, 0), (-73.94904030000001, 45.736359160000085, 0, 0), (-73.93409890000001, 45.736359160000085, 0, 0), (-73.91915750000001, 45.736359160000085, 0, 0), (-73.90421610000001, 45.736359160000085, 0, 0), (-73.88927470000002, 45.736359160000085, 0, 0), (-73.87433330000002, 45.736359160000085, 0, 0), (-73.85939190000002, 45.736359160000085, 0, 0), (-73.84445050000002, 45.736359160000085, 0, 0), (-73.82950910000002, 45.736359160000085, 0, 0), (-73.81456770000003, 45.736359160000085, 0, 0), (-73.79962630000003, 45.736359160000085, 0, 0), (-73.78468490000003, 45.736359160000085, 0, 0), (-73.76974350000003, 45.736359160000085, 0, 0), (-73.75480210000003, 45.736359160000085, 0, 0), (-73.73986070000004, 45.736359160000085, 0, 0), (-73.72491930000004, 45.736359160000085, 0, 3), (-73.70997790000004, 45.736359160000085, 0, 0), (-73.69503650000004, 45.736359160000085, 0, 0), (-73.68009510000005, 45.736359160000085, 0, 1), (-73.66515370000005, 45.736359160000085, 0, 0), (-73.65021230000005, 45.736359160000085, 0, 224), (-73.63527090000005, 45.736359160000085, 0, 0), (-73.62032950000005, 45.736359160000085, 0, 26), (-73.60538810000006, 45.736359160000085, 0, 0), (-73.59044670000006, 45.736359160000085, 0, 0), (-73.57550530000006, 45.736359160000085, 0, 0), (-73.56056390000006, 45.736359160000085, 0, 0), (-73.54562250000006, 45.736359160000085, 0, 0), (-73.53068110000007, 45.736359160000085, 0, 0), (-73.51573970000007, 45.736359160000085, 0, 0), (-73.50079830000007, 45.736359160000085, 0, 0), (-73.48585690000007, 45.736359160000085, 0, 0), (-73.47091550000007, 45.736359160000085, 0, 0), (-73.45597410000008, 45.736359160000085, 1, 51), (-73.44103270000008, 45.736359160000085, 0, 1), (-73.42609130000008, 45.736359160000085, 0, 0), (-73.41114990000008, 45.736359160000085, 0, 0), (-73.39620850000009, 45.736359160000085, 0, 0), (-73.38126710000009, 45.736359160000085, 0, 0), (-73.36632570000009, 45.736359160000085, 0, 0), (-73.35138430000009, 45.736359160000085, 0, 0), (-73.3364429000001, 45.736359160000085, 0, 0), (-73.3215015000001, 45.736359160000085, 0, 0), (-73.3065601000001, 45.736359160000085, 0, 0), (-73.2916187000001, 45.736359160000085, 0, 0), (-74.0237473, 45.74654788000009, 0, 0), (-74.0088059, 45.74654788000009, 0, 0), (-73.9938645, 45.74654788000009, 0, 0), (-73.9789231, 45.74654788000009, 0, 0), (-73.9639817, 45.74654788000009, 0, 0), (-73.94904030000001, 45.74654788000009, 0, 0), (-73.93409890000001, 45.74654788000009, 0, 0), (-73.91915750000001, 45.74654788000009, 0, 0), (-73.90421610000001, 45.74654788000009, 0, 0), (-73.88927470000002, 45.74654788000009, 0, 0), (-73.87433330000002, 45.74654788000009, 0, 0), (-73.85939190000002, 45.74654788000009, 0, 0), (-73.84445050000002, 45.74654788000009, 0, 0), (-73.82950910000002, 45.74654788000009, 0, 0), (-73.81456770000003, 45.74654788000009, 0, 0), (-73.79962630000003, 45.74654788000009, 0, 0), (-73.78468490000003, 45.74654788000009, 0, 0), (-73.76974350000003, 45.74654788000009, 0, 0), (-73.75480210000003, 45.74654788000009, 0, 0), (-73.73986070000004, 45.74654788000009, 0, 0), (-73.72491930000004, 45.74654788000009, 0, 7), (-73.70997790000004, 45.74654788000009, 0, 0), (-73.69503650000004, 45.74654788000009, 0, 0), (-73.68009510000005, 45.74654788000009, 0, 0), (-73.66515370000005, 45.74654788000009, 0, 0), (-73.65021230000005, 45.74654788000009, 0, 0), (-73.63527090000005, 45.74654788000009, 0, 0),

(-73.62032950000005, 45.74654788000009, 0, 1), (-73.60538810000006, 45.74654788000009, 0, 11), (-73.59044670000006, 45.74654788000009, 0, 0), (-73.57550530000006, 45.74654788000009, 0, 1), (-73.56056390000006, 45.74654788000009, 0, 0), (-73.54562250000006, 45.74654788000009, 0, 0), (-73.53068110000007, 45.74654788000009, 0, 0), (-73.51573970000007, 45.74654788000009, 0, 0), (-73.50079830000007, 45.74654788000009, 0, 0), (-73.48585690000007, 45.74654788000009, 0, 0), (-73.47091550000007, 45.74654788000009, 0, 2), (-73.45597410000008, 45.74654788000009, 0, 10), (-73.44103270000008, 45.74654788000009, 0, 1), (-73.42609130000008, 45.74654788000009, 0, 0), (-73.41114990000008, 45.74654788000009, 0, 0), (-73.39620850000009, 45.74654788000009, 0, 0), (-73.38126710000009, 45.74654788000009, 0, 0), (-73.36632570000009, 45.74654788000009, 0, 0), (-73.35138430000009, 45.74654788000009, 0, 0), (-73.3364429000001, 45.74654788000009, 0, 0), (-73.3215015000001, 45.74654788000009, 0, 0), (-73.3065601000001, 45.74654788000009, 0, 0), (-73.2916187000001, 45.74654788000009, 0, 0), (-74.0237473, 45.75673660000009, 0, 45), (-74.0088059, 45.75673660000009, 0, 19), (-73.9938645, 45.75673660000009, 0, 0), (-73.9789231, 45.75673660000009, 0, 0), (-73.9639817, 45.75673660000009, 0, 0), (-73.94904030000001, 45.75673660000009, 0, 0), (-73.93409890000001, 45.75673660000009, 0, 0), (-73.91915750000001, 45.75673660000009, 0, 0), (-73.90421610000001, 45.75673660000009, 0, 0), (-73.88927470000002, 45.75673660000009, 0, 0), (-73.87433330000002, 45.75673660000009, 0, 0), (-73.85939190000002, 45.75673660000009, 0, 0), (-73.84445050000002, 45.75673660000009, 0, 0), (-73.82950910000002, 45.75673660000009, 0, 0), (-73.81456770000003, 45.75673660000009, 0, 0), (-73.79962630000003, 45.75673660000009, 0, 0), (-73.78468490000003, 45.75673660000009, 0, 0), (-73.76974350000003, 45.75673660000009, 0, 0), (-73.75480210000003, 45.75673660000009, 0, 0), (-73.73986070000004, 45.75673660000009, 0, 0), (-73.72491930000004, 45.75673660000009, 0, 0), (-73.70997790000004, 45.75673660000009, 0, 0), (-73.69503650000004, 45.75673660000009, 0, 0), (-73.68009510000005, 45.75673660000009, 0, 0), (-73.66515370000005, 45.75673660000009, 0, 0), (-73.65021230000005, 45.75673660000009, 0, 0), (-73.63527090000005, 45.75673660000009, 0, 0), (-73.62032950000005, 45.75673660000009, 0, 0), (-73.60538810000006, 45.75673660000009, 0, 0), (-73.59044670000006, 45.75673660000009, 0, 0), (-73.57550530000006, 45.75673660000009, 0, 0), (-73.56056390000006, 45.75673660000009, 0, 0), (-73.54562250000006, 45.75673660000009, 0, 0), (-73.53068110000007, 45.75673660000009, 0, 0), (-73.51573970000007, 45.75673660000009, 0, 0), (-73.50079830000007, 45.75673660000009, 0, 0), (-73.48585690000007, 45.75673660000009, 0, 3), (-73.47091550000007, 45.75673660000009, 0, 15), (-73.45597410000008, 45.75673660000009, 0, 0), (-73.44103270000008, 45.75673660000009, 6, 113), (-73.42609130000008, 45.75673660000009, 0, 0), (-73.41114990000008, 45.75673660000009, 0, 0), (-73.39620850000009, 45.75673660000009, 0, 0), (-73.38126710000009, 45.75673660000009, 0, 0), (-73.36632570000009, 45.75673660000009, 0, 0), (-73.35138430000009, 45.75673660000009, 0, 0), (-73.3364429000001, 45.75673660000009, 0, 0), (-73.3215015000001, 45.75673660000009, 0, 0), (-73.3065601000001, 45.75673660000009, 0, 0), (-73.2916187000001, 45.75673660000009, 0, 0), (-74.0237473, 45.76692532000009, 0, 1), (-74.0088059, 45.76692532000009, 0, 1), (-73.9938645, 45.76692532000009, 0, 2), (-73.9789231, 45.76692532000009, 0, 6), (-73.9639817, 45.76692532000009, 0, 0), (-73.94904030000001, 45.76692532000009, 0, 0), (-73.93409890000001, 45.76692532000009, 0, 0), (-73.91915750000001, 45.76692532000009, 0, 0), (-73.90421610000001, 45.76692532000009, 0, 0), (-73.88927470000002, 45.76692532000009, 0, 0), (-73.87433330000002, 45.76692532000009, 0, 0), (-73.85939190000002, 45.76692532000009, 0, 0), (-73.84445050000002, 45.76692532000009, 0, 0), (-73.82950910000002, 45.76692532000009, 0, 0), (-73.81456770000003, 45.76692532000009, 0, 1), (-73.79962630000003, 45.76692532000009, 0, 1), (-73.78468490000003, 45.76692532000009, 0, 0), (-73.76974350000003, 45.76692532000009, 0, 0), (-73.75480210000003,

45.76692532000009, 0, 0), (-73.73986070000004, 45.76692532000009, 0, 0), (-73.72491930000004, 45.76692532000009, 0, 0), (-73.70997790000004, 45.76692532000009, 0, 0), (-73.69503650000004, 45.76692532000009, 0, 0), (-73.68009510000005, 45.76692532000009, 0, 0), (-73.66515370000005, 45.76692532000009, 0, 1), (-73.65021230000005, 45.76692532000009, 0, 0), (-73.63527090000005, 45.76692532000009, 0, 0), (-73.62032950000005, 45.76692532000009, 0, 0), (-73.60538810000006, 45.76692532000009, 0, 0), (-73.59044670000006, 45.76692532000009, 0, 0), (-73.57550530000006, 45.76692532000009, 0, 0), (-73.56056390000006, 45.76692532000009, 0, 0), (-73.54562250000006, 45.76692532000009, 0, 0), (-73.53068110000007, 45.76692532000009, 0, 0), (-73.51573970000007, 45.76692532000009, 0, 0), (-73.50079830000007, 45.76692532000009, 0, 0), (-73.48585690000007, 45.76692532000009, 0, 0), (-73.47091550000007, 45.76692532000009, 0, 0), (-73.45597410000008, 45.76692532000009, 0, 0), (-73.44103270000008, 45.76692532000009, 0, 0), (-73.42609130000008, 45.76692532000009, 0, 0), (-73.41114990000008, 45.76692532000009, 0, 0), (-73.39620850000009, 45.76692532000009, 0, 0), (-73.38126710000009, 45.76692532000009, 0, 0), (-73.36632570000009, 45.76692532000009, 0, 0), (-73.35138430000009, 45.76692532000009, 0, 3), (-73.3364429000001, 45.76692532000009, 0, 0), (-73.3215015000001, 45.76692532000009, 0, 0), (-73.3065601000001, 45.76692532000009, 0, 0), (-73.2916187000001, 45.76692532000009, 0, 0), (-74.0237473, 45.77711404000009, 0, 2), (-74.0088059, 45.77711404000009, 5, 123), (-73.9938645, 45.77711404000009, 0, 8), (-73.9789231, 45.77711404000009, 0, 0), (-73.9639817, 45.77711404000009, 0, 0), (-73.94904030000001, 45.77711404000009, 0, 0), (-73.93409890000001, 45.77711404000009, 0, 0), (-73.91915750000001, 45.77711404000009, 0, 0), (-73.90421610000001, 45.77711404000009, 0, 0), (-73.88927470000002, 45.77711404000009, 0, 0), (-73.87433330000002, 45.77711404000009, 0, 0), (-73.85939190000002, 45.77711404000009, 0, 0), (-73.84445050000002, 45.77711404000009, 0, 0), (-73.82950910000002, 45.77711404000009, 0, 0), (-73.81456770000003, 45.77711404000009, 0, 0), (-73.79962630000003, 45.77711404000009, 0, 0), (-73.78468490000003, 45.77711404000009, 0, 0), (-73.76974350000003, 45.77711404000009, 0, 0), (-73.75480210000003, 45.77711404000009, 0, 0), (-73.73986070000004, 45.77711404000009, 0, 0), (-73.72491930000004, 45.77711404000009, 0, 0), (-73.70997790000004, 45.77711404000009, 0, 0), (-73.69503650000004, 45.77711404000009, 0, 0), (-73.68009510000005, 45.77711404000009, 0, 0), (-73.66515370000005, 45.77711404000009, 0, 0), (-73.65021230000005, 45.77711404000009, 0, 0), (-73.63527090000005, 45.77711404000009, 1, 52), (-73.62032950000005, 45.77711404000009, 0, 0), (-73.60538810000006, 45.77711404000009, 0, 0), (-73.59044670000006, 45.77711404000009, 0, 0), (-73.57550530000006, 45.77711404000009, 0, 0), (-73.56056390000006, 45.77711404000009, 0, 0), (-73.54562250000006, 45.77711404000009, 0, 0), (-73.53068110000007, 45.77711404000009, 0, 0), (-73.51573970000007, 45.77711404000009, 0, 0), (-73.50079830000007, 45.77711404000009, 0, 0), (-73.48585690000007, 45.77711404000009, 0, 0), (-73.47091550000007, 45.77711404000009, 0, 0), (-73.45597410000008, 45.77711404000009, 0, 0), (-73.44103270000008, 45.77711404000009, 0, 0), (-73.42609130000008, 45.77711404000009, 0, 0), (-73.41114990000008, 45.77711404000009, 0, 0), (-73.39620850000009, 45.77711404000009, 0, 0), (-73.38126710000009, 45.77711404000009, 0, 0), (-73.36632570000009, 45.77711404000009, 0, 7), (-73.35138430000009, 45.77711404000009, 0, 2), (-73.3364429000001, 45.77711404000009, 0, 0), (-73.3215015000001, 45.77711404000009, 0, 0), (-73.3065601000001, 45.77711404000009, 0, 0), (-73.2916187000001, 45.77711404000009, 0, 0), (-74.0237473, 45.787302760000095, 0, 0), (-74.0088059, 45.787302760000095, 0, 0), (-73.9938645, 45.787302760000095, 1, 3), (-73.9789231, 45.787302760000095, 0, 1), (-73.9639817, 45.787302760000095, 0, 0), (-73.94904030000001, 45.787302760000095, 0, 0), (-73.93409890000001, 45.787302760000095, 0, 1), (-73.91915750000001, 45.787302760000095, 0, 0), (-73.90421610000001, 45.787302760000095, 0, 0), (-73.88927470000002, 45.787302760000095, 0, 0),

(-73.87433330000002, 45.787302760000095, 0, 0), (-73.85939190000002, 45.787302760000095, 0, 0), (-73.84445050000002, 45.787302760000095, 0, 0), (-73.82950910000002, 45.787302760000095, 0, 0), (-73.81456770000003, 45.787302760000095, 0, 0), (-73.79962630000003, 45.787302760000095, 0, 0), (-73.78468490000003, 45.787302760000095, 0, 0), (-73.76974350000003, 45.787302760000095, 0, 0), (-73.75480210000003, 45.787302760000095, 0, 0), (-73.73986070000004, 45.787302760000095, 0, 0), (-73.72491930000004, 45.787302760000095, 0, 0), (-73.70997790000004, 45.787302760000095, 0, 0), (-73.69503650000004, 45.787302760000095, 0, 0), (-73.68009510000005, 45.787302760000095, 0, 0), (-73.66515370000005, 45.787302760000095, 0, 0), (-73.65021230000005, 45.787302760000095, 0, 0), (-73.63527090000005, 45.787302760000095, 0, 0), (-73.62032950000005, 45.787302760000095, 0, 0), (-73.60538810000006, 45.787302760000095, 0, 0), (-73.59044670000006, 45.787302760000095, 0, 0), (-73.57550530000006, 45.787302760000095, 0, 0), (-73.56056390000006, 45.787302760000095, 0, 0), (-73.54562250000006, 45.787302760000095, 0, 0), (-73.53068110000007, 45.787302760000095, 0, 0), (-73.51573970000007, 45.787302760000095, 0, 0), (-73.50079830000007, 45.787302760000095, 0, 0), (-73.48585690000007, 45.787302760000095, 0, 0), (-73.47091550000007, 45.787302760000095, 0, 0), (-73.45597410000008, 45.787302760000095, 0, 0), (-73.44103270000008, 45.787302760000095, 0, 0), (-73.42609130000008, 45.787302760000095, 0, 0), (-73.41114990000008, 45.787302760000095, 0, 0), (-73.39620850000009, 45.787302760000095, 0, 0), (-73.38126710000009, 45.787302760000095, 0, 0), (-73.36632570000009, 45.787302760000095, 0, 0), (-73.35138430000009, 45.787302760000095, 0, 0), (-73.3364429000001, 45.787302760000095, 0, 0), (-73.3215015000001, 45.787302760000095, 0, 0), (-73.3065601000001, 45.787302760000095, 0, 0), (-73.2916187000001, 45.787302760000095, 0, 0), (-74.0237473, 45.7974914800001, 0, 9), (-74.0088059, 45.7974914800001, 0, 0), (-73.9938645, 45.7974914800001, 0, 0), (-73.9789231, 45.7974914800001, 0, 0), (-73.9639817, 45.7974914800001, 0, 0), (-73.94904030000001, 45.7974914800001, 0, 0), (-73.93409890000001, 45.7974914800001, 0, 0), (-73.91915750000001, 45.7974914800001, 0, 0), (-73.90421610000001, 45.7974914800001, 0, 0), (-73.88927470000002, 45.7974914800001, 0, 0), (-73.87433330000002, 45.7974914800001, 0, 0), (-73.85939190000002, 45.7974914800001, 0, 0), (-73.84445050000002, 45.7974914800001, 0, 0), (-73.82950910000002, 45.7974914800001, 0, 0), (-73.81456770000003, 45.7974914800001, 0, 0), (-73.79962630000003, 45.7974914800001, 0, 0), (-73.78468490000003, 45.7974914800001, 0, 0), (-73.76974350000003, 45.7974914800001, 0, 0), (-73.75480210000003, 45.7974914800001, 0, 0), (-73.73986070000004, 45.7974914800001, 0, 0), (-73.72491930000004, 45.7974914800001, 0, 0), (-73.70997790000004, 45.7974914800001, 0, 0), (-73.69503650000004, 45.7974914800001, 0, 0), (-73.68009510000005, 45.7974914800001, 0, 0), (-73.66515370000005, 45.7974914800001, 0, 0), (-73.65021230000005, 45.7974914800001, 0, 0), (-73.63527090000005, 45.7974914800001, 0, 0), (-73.62032950000005, 45.7974914800001, 0, 0), (-73.60538810000006, 45.7974914800001, 0, 0), (-73.59044670000006, 45.7974914800001, 0, 0), (-73.57550530000006, 45.7974914800001, 0, 0), (-73.56056390000006, 45.7974914800001, 0, 0), (-73.54562250000006, 45.7974914800001, 0, 0), (-73.53068110000007, 45.7974914800001, 0, 0), (-73.51573970000007, 45.7974914800001, 0, 0), (-73.50079830000007, 45.7974914800001, 0, 0), (-73.48585690000007, 45.7974914800001, 0, 0), (-73.47091550000007, 45.7974914800001, 0, 0), (-73.45597410000008, 45.7974914800001, 0, 0), (-73.44103270000008, 45.7974914800001, 0, 0), (-73.42609130000008, 45.7974914800001, 0, 0), (-73.41114990000008, 45.7974914800001, 0, 0), (-73.39620850000009, 45.7974914800001, 0, 0), (-73.38126710000009, 45.7974914800001, 0, 0), (-73.36632570000009, 45.7974914800001, 0, 0), (-73.35138430000009, 45.7974914800001, 0, 0), (-73.3364429000001, 45.7974914800001, 0, 1), (-73.3215015000001, 45.7974914800001, 0, 0), (-73.3065601000001, 45.7974914800001, 0, 0), (-73.2916187000001, 45.7974914800001, 0, 0), (-74.0237473, 45.8076802000001, 0, 2), (-74.0088059, 45.8076802000001, 0, 0), (-73.9938645, 45.8076802000001, 0, 0), (-73.9789231, 45.8076802000001, 0, 0), (-73.9639817, 45.8076802000001, 0, 0), (-73.94904030000001,

45.8076802000001, 0, 0), (-73.93409890000001, 45.8076802000001, 0, 0), (-73.91915750000001, 45.8076802000001, 0, 0), (-73.90421610000001, 45.8076802000001, 0, 0), (-73.88927470000002, 45.8076802000001, 0, 0), (-73.87433330000002, 45.8076802000001, 0, 0), (-73.85939190000002, 45.8076802000001, 0, 0), (-73.84445050000002, 45.8076802000001, 0, 0), (-73.82950910000002, 45.8076802000001, 0, 0), (-73.81456770000003, 45.8076802000001, 0, 0), (-73.79962630000003, 45.8076802000001, 0, 0), (-73.78468490000003, 45.8076802000001, 0, 0), (-73.76974350000003, 45.8076802000001, 0, 0), (-73.75480210000003, 45.8076802000001, 0, 0), (-73.73986070000004, 45.8076802000001, 0, 0), (-73.72491930000004, 45.8076802000001, 0, 0), (-73.70997790000004, 45.8076802000001, 0, 0), (-73.69503650000004, 45.8076802000001, 0, 0), (-73.68009510000005, 45.8076802000001, 0, 0), (-73.66515370000005, 45.8076802000001, 0, 0), (-73.65021230000005, 45.8076802000001, 0, 0), (-73.63527090000005, 45.8076802000001, 0, 0), (-73.62032950000005, 45.8076802000001, 0, 0), (-73.60538810000006, 45.8076802000001, 0, 0), (-73.59044670000006, 45.8076802000001, 0, 0), (-73.57550530000006, 45.8076802000001, 0, 0), (-73.56056390000006, 45.8076802000001, 0, 0), (-73.54562250000006, 45.8076802000001, 0, 0), (-73.53068110000007, 45.8076802000001, 0, 0), (-73.51573970000007, 45.8076802000001, 0, 0), (-73.50079830000007, 45.8076802000001, 0, 0), (-73.48585690000007, 45.8076802000001, 0, 0), (-73.47091550000007, 45.8076802000001, 0, 0), (-73.45597410000008, 45.8076802000001, 0, 0), (-73.44103270000008, 45.8076802000001, 0, 0), (-73.42609130000008, 45.8076802000001, 0, 0), (-73.41114990000008, 45.8076802000001, 0, 0), (-73.39620850000009, 45.8076802000001, 0, 0), (-73.38126710000009, 45.8076802000001, 0, 0), (-73.36632570000009, 45.8076802000001, 0, 0), (-73.35138430000009, 45.8076802000001, 0, 0), (-73.3364429000001, 45.8076802000001, 0, 0), (-73.3215015000001, 45.8076802000001, 0, 0), (-73.3065601000001, 45.8076802000001, 0, 0), (-73.2916187000001, 45.8076802000001, 0, 0), (-74.0237473, 45.8178689200001, 0, 0), (-74.0088059, 45.8178689200001, 0, 0), (-73.9938645, 45.8178689200001, 0, 0), (-73.9789231, 45.8178689200001, 0, 0), (-73.9639817, 45.8178689200001, 0, 0), (-73.94904030000001, 45.8178689200001, 0, 0), (-73.93409890000001, 45.8178689200001, 0, 486), (-73.91915750000001, 45.8178689200001, 0, 0), (-73.90421610000001, 45.8178689200001, 0, 2), (-73.88927470000002, 45.8178689200001, 0, 0), (-73.87433330000002, 45.8178689200001, 0, 0), (-73.85939190000002, 45.8178689200001, 0, 0), (-73.84445050000002, 45.8178689200001, 0, 0), (-73.82950910000002, 45.8178689200001, 0, 0), (-73.81456770000003, 45.8178689200001, 0, 0), (-73.79962630000003, 45.8178689200001, 0, 0), (-73.78468490000003, 45.8178689200001, 0, 0), (-73.76974350000003, 45.8178689200001, 0, 0), (-73.75480210000003, 45.8178689200001, 0, 0), (-73.73986070000004, 45.8178689200001, 0, 0), (-73.72491930000004, 45.8178689200001, 0, 0), (-73.70997790000004, 45.8178689200001, 0, 0), (-73.69503650000004, 45.8178689200001, 0, 0), (-73.68009510000005, 45.8178689200001, 0, 0), (-73.66515370000005, 45.8178689200001, 0, 0), (-73.65021230000005, 45.8178689200001, 0, 0), (-73.63527090000005, 45.8178689200001, 0, 0), (-73.62032950000005, 45.8178689200001, 0, 0), (-73.60538810000006, 45.8178689200001, 0, 0), (-73.59044670000006, 45.8178689200001, 0, 0), (-73.57550530000006, 45.8178689200001, 0, 0), (-73.56056390000006, 45.8178689200001, 0, 0), (-73.54562250000006, 45.8178689200001, 0, 0), (-73.53068110000007, 45.8178689200001, 0, 0), (-73.51573970000007, 45.8178689200001, 0, 0), (-73.50079830000007, 45.8178689200001, 0, 0), (-73.48585690000007, 45.8178689200001, 0, 0), (-73.47091550000007, 45.8178689200001, 0, 0), (-73.45597410000008, 45.8178689200001, 0, 0), (-73.44103270000008, 45.8178689200001, 0, 0), (-73.42609130000008, 45.8178689200001, 0, 0), (-73.41114990000008, 45.8178689200001, 0, 0), (-73.39620850000009, 45.8178689200001, 0, 0), (-73.38126710000009, 45.8178689200001, 0, 0), (-73.36632570000009, 45.8178689200001, 0, 0), (-73.35138430000009, 45.8178689200001, 0, 0), (-73.3364429000001, 45.8178689200001, 0, 0), (-73.3215015000001, 45.8178689200001, 0, 0), (-73.3065601000001, 45.8178689200001, 0, 0), (-73.2916187000001, 45.8178689200001, 0, 0), (-74.0237473, 45.828057640000104, 0, 0), (-74.0088059, 45.828057640000104, 0, 0), (-73.9938645, 45.828057640000104, 0, 0), (-73.9789231, 45.828057640000104, 0, 0), (-73.9639817, 45.828057640000104, 0, 0), (-73.94904030000001, 45.828057640000104, 0, 0), (-73.93409890000001, 45.828057640000104,

0, 0), (-73.91915750000001, 45.828057640000104, 0, 0), (-73.90421610000001, 45.828057640000104, 0, 0), (-73.88927470000002, 45.828057640000104, 0, 0), (-73.87433330000002, 45.828057640000104, 0, 0), (-73.85939190000002, 45.828057640000104, 0, 0), (-73.84445050000002, 45.828057640000104, 0, 0), (-73.82950910000002, 45.828057640000104, 0, 0), (-73.81456770000003, 45.828057640000104, 0, 0), (-73.79962630000003, 45.828057640000104, 0, 0), (-73.78468490000003, 45.828057640000104, 0, 0), (-73.76974350000003, 45.828057640000104, 0, 0), (-73.75480210000003, 45.828057640000104, 0, 0), (-73.73986070000004, 45.828057640000104, 0, 0), (-73.72491930000004, 45.828057640000104, 0, 0), (-73.70997790000004, 45.828057640000104, 0, 0), (-73.69503650000004, 45.828057640000104, 0, 0), (-73.68009510000005, 45.828057640000104, 0, 0), (-73.66515370000005, 45.828057640000104, 0, 0), (-73.65021230000005, 45.828057640000104, 0, 0), (-73.63527090000005, 45.828057640000104, 0, 0), (-73.62032950000005, 45.828057640000104, 0, 0), (-73.60538810000006, 45.828057640000104, 0, 1), (-73.59044670000006, 45.828057640000104, 0, 0), (-73.57550530000006, 45.828057640000104, 0, 0), (-73.56056390000006, 45.828057640000104, 0, 0), (-73.54562250000006, 45.828057640000104, 0, 0), (-73.53068110000007, 45.828057640000104, 0, 0), (-73.51573970000007, 45.828057640000104, 0, 0), (-73.50079830000007, 45.828057640000104, 0, 0), (-73.48585690000007, 45.828057640000104, 0, 0), (-73.47091550000007, 45.828057640000104, 0, 0), (-73.45597410000008, 45.828057640000104, 0, 0), (-73.44103270000008, 45.828057640000104, 0, 1), (-73.42609130000008, 45.828057640000104, 0, 0), (-73.41114990000008, 45.828057640000104, 0, 0), (-73.39620850000009, 45.828057640000104, 0, 0), (-73.38126710000009, 45.828057640000104, 0, 0), (-73.36632570000009, 45.828057640000104, 0, 1), (-73.35138430000009, 45.828057640000104, 0, 0), (-73.3364429000001, 45.828057640000104, 0, 0), (-73.3215015000001, 45.828057640000104, 0, 0), (-73.3065601000001, 45.828057640000104, 0, 0), (-73.2916187000001, 45.828057640000104, 0, 0)]
